# Type 2 diabetes attributable to ambient particulate matter pollution: a global burden study from 1990 to 2019

**DOI:** 10.3389/fpubh.2024.1371253

**Published:** 2024-05-20

**Authors:** Yuyi Sha, Shuai Wang

**Affiliations:** ^1^Department of Intensive Care Medicine, Ningbo No.2 Hospital, Ningbo, Zhejiang, China; ^2^Department of Rehabilitation Medicine, Ningbo No.2 Hospital, Ningbo, Zhejiang, China

**Keywords:** type 2 diabetes, ambient particulate matter pollution, EAPC, socio-demographic index, adult

## Abstract

**Background:**

This study assesses the changes over time and geographical locations in the disease burden of type 2 diabetes (T2D) attributed to ambient particulate matter pollution (APMP) from 1990 to 2019 in 204 countries and regions with different socio-demographic indexes (SDI).

**Methods:**

The Global Burden of Diseases Study 2019 (GBD2019) database was used to analyze the global burden of T2D attributed to APMP. This study evaluated both the age-standardized death rate (ASDR) and disability-adjusted life years (DALYs) related to T2D, comparing data from 1990 to 2019. Estimated Annual Percentage Changes (EAPCs) were also utilized to investigate the trends over the 30-year study period.

**Results:**

The global age-standardized DALY rate and ASDR exhibited an increasing trend, with an EAPC of 2.21 (95% CI: 2.15 to 2.27) and 1.50 (95% CI: 1.43 to 1.58), respectively. This rise was most notable among older adult populations, men, regions in Africa and Asia, as well as low-middle SDI regions. In 2019, the ASDR for T2D caused by APMP was recorded at 2.47 per 100,000 population, while the DALY rate stood at 108.98 per 100,000 population. Males and countries with middle SDI levels displayed significantly high age-standardized death and DALY rates, particularly noticeable in Southern Sub-Saharan Africa. Conversely, regions with high SDI levels like High-income North America demonstrated decreasing trends.

**Conclusion:**

This study reveals a significant increase in T2D worldwide as a result of APMP from 1990 to 2019, with a particular emphasis on its impact on men, the older adult, and regions with low to middle SDI levels. These results underscore the urgent necessity for implementing policies aimed at addressing air pollution in order to reduce the prevalence of T2D, especially in the areas most heavily affected.

## 1 Introduction

The rapid acceleration of global economic development and urbanization has significantly impacted lifestyles, leading to an increase in health conditions, particularly T2D (1). Exposure to APMP, a common form of air pollution prevalent in urban areas, is a key factor contributing to this rise (2). APMP is composed of a variety of particles with different sizes and chemical compositions, posing substantial risks to public health (3). The correlation between APMP and diseases like T2D is now a major global concern (4). This study aims to evaluate the global burden of T2D attributed to APMP through a detailed analysis spanning 29 years from 1990 to 2019.

In 2019, T2D accounted for 2.6% (1.47 million) of global deaths (5), with 13.4% of these deaths attributed to APMP (6). Studies examining the relationship between APMP and T2D have generated conflicting results, contrasting with the consistent negative associations identified between APMP and cardiovascular and respiratory diseases (7–11). Nevertheless, multiple epidemiological studies have established a connection between APMP exposure and an elevated risk of T2D. For instance, Li et al. illustrated this link, which was further supported by an Italian study indicating a correlation between higher annual average APMP levels and an increase in diabetes-related hospital admissions (12, 13). Similarly, Lao et al. noted a relationship between prolonged exposure to high levels of APMP and T2D in Asia (14). Despite these discoveries, there remains a lack of comprehensive global assessments on the burden of T2D attributed to APMP, particularly in diverse socio-economic contexts. This research gap underscores the critical need for more extensive studies to fully comprehend the specific effects of APMP on T2D in various populations and environmental conditions.

This study addresses a research gap by utilizing data from the GBD2019 to estimate the spatiotemporal trends of the burden of T2D attributed to APMP. The focus is on identifying regions most severely affected and providing insights for policymaking and strategies to mitigate air pollution-related disease burden. The analysis incorporates the SDI developed by the Institute for Health Metrics and Evaluation (IHME) in 2015 (15), increasingly used in recent studies to assess disease burdens considering socio-economic levels (16–18). The study presents a comprehensive 30-year trend analysis (1990–2019) of T2D burden due to APMP across 204 countries and regions, categorized by SDIs. The findings offer valuable insights for policymaking and actions to reduce air pollution and its health impacts.

## 2 Methods

### 2.1 Data source

Data on the global burden of Type 2 Diabetes attributed to Ambient Particulate Matter Pollution (APMP) was sourced from the Global Burden of Disease Study 2019 (19). The GBD studies offer thorough evaluations of the health consequences resulting from 329 diseases across 204 countries and territories, grouped into 21 regions based on epidemiological similarities and geographical proximity. GBD 2019 utilized a triangulated approach for PM2.5 data collection that included ground-based measurements, satellite estimates, and conversions from PM10 measurements: Over 10,000 ground monitors across 116 countries provided direct readings of PM10 and PM2.5 concentrations from 2010 to 2017, offering reliable data essential for calibrating other sources. In areas lacking ground monitors, satellite-derived PM2.5 estimates were used. These estimates are based on aerosol optical depth (AOD) measurements from updated satellite products, processed through the GEOS-Chem chemical transport model to correlate satellite observations with ground-level PM2.5. For regions without direct PM2.5 monitoring, PM2.5 levels were estimated using conversion factors that consider local, national, and regional data. To quantify the burden of T2D attributable to APMP, the GBD 2019 applied the population-attributable fraction (PAF) method. This method calculates the proportion of disease DALYs or mortality that can be attributed to a specific risk factor, considering age, sex, location, and year. The PAF for T2D due to APMP was determined by integrating relative risk estimates for T2D associated with APMP exposure levels, derived from systematic reviews of epidemiological studies (19).

The burden of T2D related to APMP was then estimated by applying these PAFs to the total number of T2D deaths and DALYs, providing a comprehensive view of how APMP contributes to T2D morbidity and mortality globally. This approach allowed for the assessment of T2D burden attributable to APMP across different demographics and geographies, reflecting the varying levels of pollution exposure and disease risk.

The SDI for each country is determined by various factors including economic growth, fertility rate, and educational attainment. These countries and territories are categorized into five SDI levels: high (greater than 0.81), high-middle (0.70–0.81), middle (0.61–0.69), low-middle (0.46–0.60), and low (less than 0.46). Our analysis focused on data from 1990 to 2019, specifically looking at the number of deaths, DALYs, and age-standardized rates (ASRs) related to particulate matter pollution. This data was further dissected by age, sex, geographical location, and year to offer a comprehensive understanding of the global patterns and effects of Air Pollution-Related Mortality on T2D. The study utilized publicly available data and did not require ethical approval.

### 2.2 Defining APMP and clinical diagnostic criteria for T2D

The APMP measures the mass concentration of particles with an aerodynamic diameter less than 2.5 μm per cubic meter of air (20). Outdoor PM2.5 pollution, similar to APMP, is predominantly caused by exposure to PM2.5 particles in outdoor air environments. The clinical diagnosis of T2D relies on standardized criteria, essential for accurately identifying T2D within the context of the GBD study. T2D is classified under the ICD-10 codes E11 in the International Statistical Classification of Diseases and Related Health Problems.

### 2.3 Statistical analyses

To address the heterogeneity in the burden of T2D resulting from variations in age structure, quantified by death rates and DALYs, we utilized age-standardized DALY rates and ASDRs in our analysis. This approach effectively neutralizes the influence of age structure differences, enabling a more precise estimation of the T2D burden attributable to APMP. To examine the temporal trends in these age-standardized rates between 1990 and 2019, we employed metrics such as percentage change, annual percentage change (APCs), and EAPCs. At the national, regional, and global levels, a linear regression model was utilized by fitting the natural logarithm of the ASRs to the model: ln (ASR) = α + βX + ε, where ‘X’ represents the calendar year. The EAPC and its corresponding 95% confidence interval (CI) were calculated as 100 × (exp(β) – 1). Trends were classified as stable if the EAPC’s 95% CI included 0 (*p* ≥ 0.05), and as increasing (EAPC and its 95% CI > 0) or decreasing (EAPC and its 95% CI < 0) otherwise. This methodological framework ensures a comprehensive and systematic analysis of the global T2D burden attributable to APMP, while integrating epidemiological and socio-demographic factors. For further details on these estimates, please refer to the GBD Compare visualization tool^1^ and the GBD Results Tool.^2^

## 3 Results

### 3.1 2019 global trends and burden of T2D attributable to APMP: a temporal analysis

**Figure 1:**
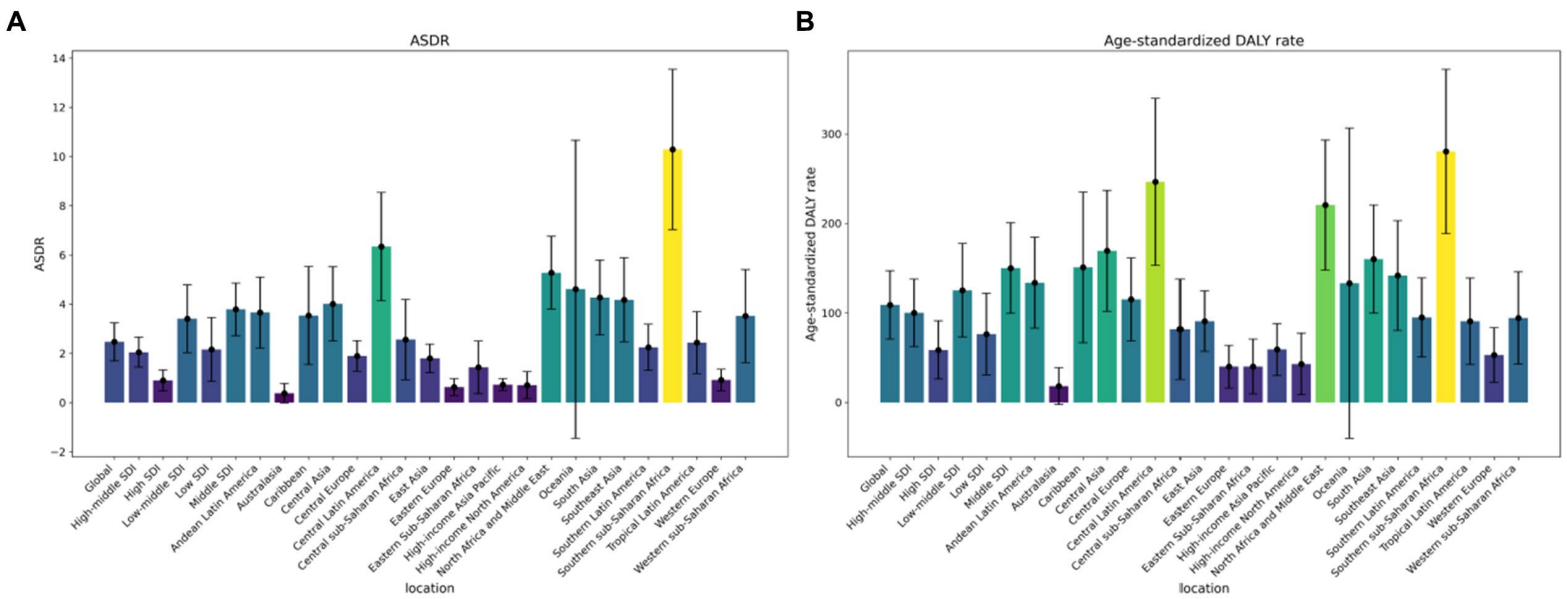
Change of type 2 diabetes mellitus rates attributed to ambient particulate matter pollution in from 1990 to 2019 by regions: **(A)** Deaths number, **(B)** DALY number. DALY, disability-adjusted life-year.

In 2019, the burden of attributable APMP led to an estimated 196.8 thousand deaths globally, with a range of 136.3 to 258.4 thousand. This number is significantly higher, at 3.53 times, than the figure recorded in 1990. Moreover, APMP also contributed to approximately 9.0 million DALYs associated with T2D, with a range of 6.1 to 12.2 million, on a global scale. This represents a 3.88-fold increase compared to the recorded number in 1990 (refer to Supplementary Table 1 and Figure 1 for detailed data).

**Figure 2:**
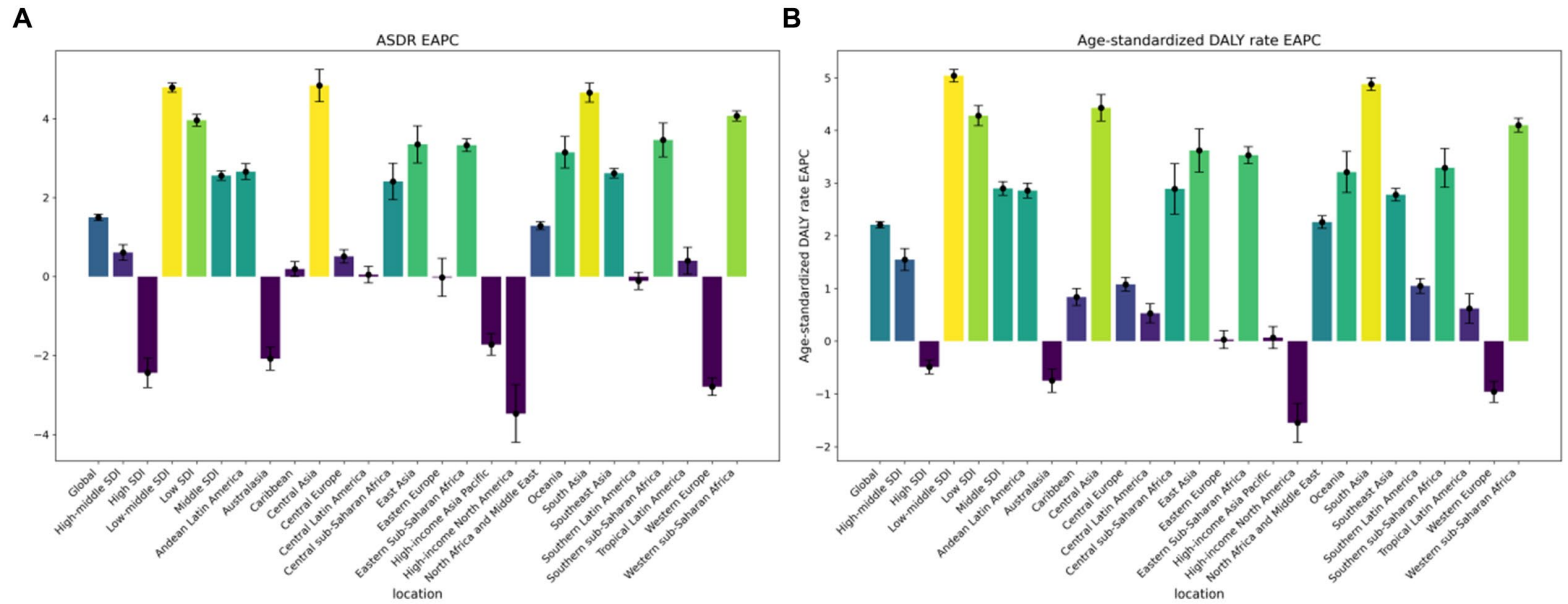
The ASRs of global burden of type 2 diabetes mellitus attributed to ambient particulate matter pollution by region: **(A)**. The EAPC of ASDR from 1990-2019. **(B)**. The EAPC of age-standardized DALY rate from 1990-2019. ASRs, age-standardized rates; ASDR, age-standardized Death rate; DALY, disability adjusted life-year.

**Figure 3:**
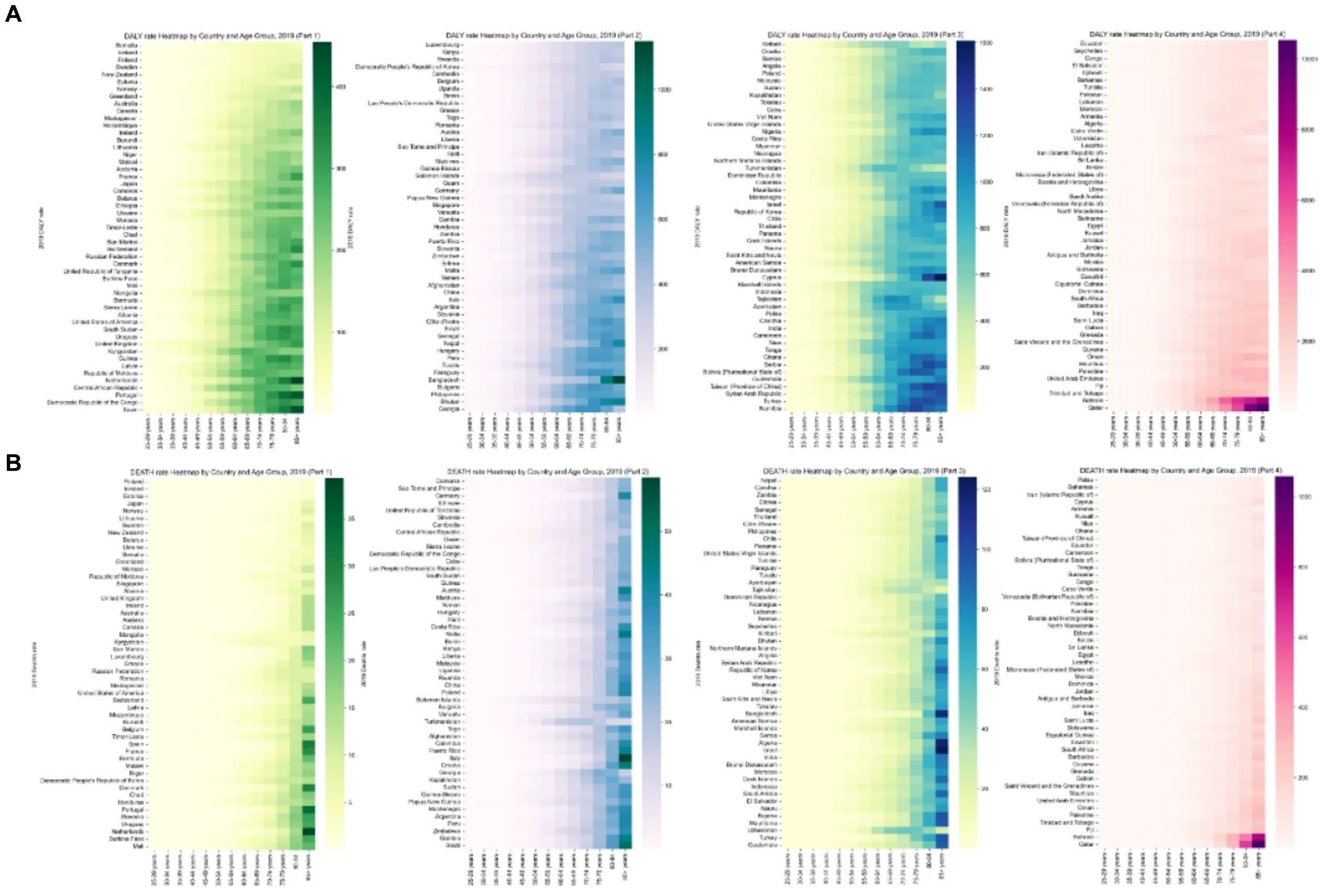
Trends in EAPCs of type 2 diabetes mellitus attributed to ambient particulate matter pollution by region: **(A)**. The EAPC of age-standardized DALY rate from 1990-2019. **(B)**. The EAPC of ASDR from 1990-2019. EAPCs, estimated annual percentage changes; DALY, disability-adjusted life-year.

Since 1990, there has been a notable rise in the number of deaths and DALYs linked to T2D due to exposure to APMP (Figure 2). The ASDR and DALY rates related to APMP have also shown similar upward trends. Globally, in 2019, the ASDR and age-standardized DALY rates for T2D attributed to APMP were 2.47 and 108.98, respectively (Table 1). Between 1990 and 2019, the global age-standardized DALY rate and ASDR for T2D linked to APMP have increased, with EAPCs of 2.21 (2.15, 2.27) for the DALY rate and 1.50 (1.43, 1.58) for the ASDR, respectively (Supplementary Table 3). Regionally, High-income North America saw the most significant decrease in the age-standardized DALY rate (EAPC = −1.55). Conversely, South Asia experienced the largest rise in this rate (EAPC = 4.88). In terms of the ASDR, the largest decrease was also observed in High-income North America (EAPC = −3.47), while Central Asia reported a notable increase (EAPC = 4.84) (Table 1, Supplementary Tables 3, 4, and Figure 3).

**Table 1:** The ASDR and age-standardized DALY rate of type 2 diabetes burden attributed to ambient partical matter pollution from 1990 to 2019 in different regions and countries.

	ASDR (per 100000) No. (95%UI)			Age–standardized DALY rate (per 100000) No. (95%UI)		
Location	1990	2019	EAPC	1990	2019	EAPC
Global	1.57 (1.04, 2.21)	2.47 (1.71, 3.24)	1.50 (1.43, 1.58)	58.36 (36.59, 83.10)	108.98 (74.06, 147.23)	2.21 (2.15, 2.27)
Sex	–	–	–	–	–	–
Male	1.62 (1.06, 2.28)	2.76 (1.94, 3.66)	1.81 (1.71, 1.92)	62.55 (39.08, 89.38)	122.21 (84.12, 165.91)	2.37 (2.29, 2.44)
Female	1.52 (1.01, 2.14)	2.22 (1.54, 2.91)	1.22 (1.16, 1.28)	54.42 (34.32, 77.87)	96.89 (65.30, 132.54)	2.04 (1.99, 2.09)
Sociodemographic index	–	–	–	–	–	–
High–middle SDI	1.72 (1.16, 2.37)	2.05 (1.46, 2.66)	0.61 (0.41, 0.81)	67.22 (43.63, 95.71)	100.16 (68.29, 137.91)	1.55 (1.34, 1.76)
High SDI	1.57 (0.93, 2.31)	0.90 (0.56, 1.33)	−2.44 (−2.80, −2.07)	64.16 (36.52, 97.13)	58.63 (33.80, 91.13)	−0.49 (−0.61, −0.36)
Low–middle SDI	0.94 (0.41, 1.75)	3.41 (2.18, 4.79)	4.79 (4.68, 4.91)	31.66 (13.48, 58.69)	125.41 (79.54, 177.60)	5.04 (4.93, 5.16)
Low SDI	0.77 (0.22, 1.69)	2.16 (1.12, 3.46)	3.96 (3.81, 4.11)	24.36 (7.19, 53.98)	76.33 (40.76, 121.87)	4.28 (4.09, 4.47)
Middle SDI	1.83 (1.14, 2.71)	3.79 (2.70, 4.86)	2.56 (2.43, 2.68)	65.15 (38.66, 97.65)	150.14 (103.58, 200.72)	2.90 (2.77, 3.03)
Region	–	–	–	–	–	–
Andean Latin America	1.78 (0.76, 3.18)	3.66 (2.36, 5.10)	2.66 (2.46, 2.86)	60.28 (25.99, 104.51)	133.84 (85.15, 184.64)	2.86 (2.72, 3.00)
Australasia	0.59 (0.05, 1.44)	0.38 (0.09, 0.78)	−2.08 (−2.36, −1.79)	19.71 (1.82, 48.04)	18.31 (4.08, 38.78)	−0.75 (−0.96, −0.53)
Caribbean	3.16 (1.26, 5.26)	3.54 (1.96, 5.53)	0.19 (0.00, 0.38)	113.77 (44.48, 197.75)	150.91 (81.57, 235.09)	0.84 (0.67, 1.00)
Central Asia	0.93 (0.43, 1.52)	4.01 (2.69, 5.52)	4.84 (4.43, 5.25)	47.13 (20.67, 81.71)	169.40 (110.80, 237.02)	4.43 (4.17, 4.68)
Central Europe	1.75 (0.97, 2.52)	1.90 (1.27, 2.52)	0.51 (0.35, 0.68)	86.74 (45.74, 131.04)	115.15 (72.83, 161.56)	1.08 (0.95, 1.21)
Central Latin America	5.51 (2.95, 8.35)	6.34 (4.33, 8.54)	0.05 (−0.16, 0.26)	192.70 (102.55, 297.19)	246.53 (161.84, 339.83)	0.53 (0.35, 0.71)
Central sub–Saharan Africa	1.20 (0.33, 2.79)	2.56 (1.28, 4.19)	2.41 (1.94, 2.87)	33.93 (9.52, 78.45)	81.80 (41.84, 137.88)	2.89 (2.42, 3.37)
East Asia	0.72 (0.33, 1.25)	1.80 (1.28, 2.38)	3.35 (2.89, 3.82)	34.21 (15.20, 61.18)	90.83 (60.49, 124.49)	3.62 (3.22, 4.03)
Eastern Europe	0.58 (0.28, 0.91)	0.63 (0.33, 0.97)	−0.02 (−0.50, 0.46)	39.64 (18.13, 65.90)	39.97 (20.21, 63.77)	0.03 (−0.15, 0.20)
Eastern Sub–Saharan Africa	0.61 (0.18, 1.45)	1.43 (0.70, 2.50)	3.33 (3.17, 3.49)	16.31 (4.70, 38.67)	40.18 (19.83, 70.89)	3.53 (3.37, 3.69)
High–income Asia Pacific	1.06 (0.44, 1.70)	0.73 (0.49, 0.97)	−1.72 (−1.99, −1.45)	51.23 (19.44, 84.09)	59.19 (35.83, 88.06)	0.07 (−0.15, 0.28)
High–income North America	1.52 (0.56, 2.67)	0.71 (0.32, 1.26)	−3.47 (−4.20, −2.74)	68.28 (23.85, 121.39)	43.06 (18.72, 77.52)	−1.55 (−1.93, −1.18)
North Africa and Middle East	3.79 (2.65, 5.06)	5.28 (3.80, 6.76)	1.29 (1.18, 1.39)	121.82 (83.54, 162.60)	220.55 (156.88, 293.20)	2.26 (2.15, 2.38)
Oceania	1.77 (0.47, 4.79)	4.61 (1.34, 10.67)	3.15 (2.76, 3.55)	50.99 (13.84, 140.82)	133.27 (39.87, 306.72)	3.21 (2.83, 3.60)
South Asia	1.22 (0.48, 2.32)	4.27 (2.85, 5.79)	4.66 (4.42, 4.90)	41.80 (17.40, 79.64)	160.25 (106.33, 220.58)	4.88 (4.76, 5.00)
Southeast Asia	1.90 (0.89, 3.32)	4.18 (2.71, 5.89)	2.62 (2.50, 2.74)	60.56 (27.77, 106.85)	141.80 (90.88, 203.20)	2.78 (2.67, 2.90)
Southern Latin America	2.05 (0.74, 3.60)	2.25 (1.43, 3.19)	−0.11 (−0.32, 0.11)	64.25 (24.49, 113.46)	95.10 (58.78, 139.32)	1.05 (0.91, 1.19)
Southern sub–Saharan Africa	4.42 (2.89, 6.16)	10.29 (7.09, 13.55)	3.46 (3.03, 3.89)	124.34 (80.18, 172.02)	280.44 (189.36, 372.20)	3.29 (2.92, 3.66)
Tropical Latin America	2.11 (0.87, 3.75)	2.44 (1.47, 3.70)	0.40 (0.06, 0.74)	75.10 (31.35, 132.36)	90.72 (52.41, 138.96)	0.62 (0.35, 0.90)
Western Europe	2.00 (0.98, 3.07)	0.92 (0.56, 1.36)	−2.79 (−3.00, −2.57)	69.06 (32.73, 109.82)	53.11 (29.90, 83.57)	−0.96 (−1.15, −0.76)
Western sub–Saharan Africa	1.17 (0.43, 2.35)	3.52 (2.02, 5.41)	4.07 (3.94, 4.20)	30.78 (11.07, 63.52)	94.45 (54.13, 145.88)	4.10 (3.98, 4.23)
Countries	–	–	–	–	–	–
Afghanistan	0.60 (0.11, 1.66)	2.32 (0.79, 4.65)	5.61 (4.78, 6.45)	21.57 (3.88, 58.09)	92.54 (34.00, 176.43)	5.86 (5.08, 6.65)
Albania	0.30 (0.14, 0.54)	0.43 (0.26, 0.67)	1.27 (0.76, 1.79)	19.44 (8.35, 35.09)	43.95 (24.83, 67.23)	3.20 (2.95, 3.45)
Algeria	2.78 (1.58, 4.32)	3.88 (2.48, 5.55)	1.44 (1.32, 1.57)	104.36 (62.00, 156.48)	201.86 (129.10, 286.18)	2.35 (2.28, 2.42)
American Samoa	2.89 (0.72, 8.13)	4.24 (1.54, 9.55)	0.93 (0.65, 1.20)	96.90 (23.71, 256.60)	159.88 (57.06, 358.56)	1.33 (1.10, 1.57)
Andorra	0.92 (0.29, 1.65)	0.43 (0.20, 0.73)	−2.37 (−2.58, −2.16)	35.86 (11.71, 65.00)	29.89 (13.19, 51.46)	−0.44 (−0.67, −0.21)
Angola	0.77 (0.18, 1.95)	3.72 (1.82, 6.37)	5.69 (5.26, 6.13)	23.56 (5.55, 59.21)	117.01 (56.51, 200.69)	5.86 (5.41, 6.32)
Antigua and Barbuda	7.73 (2.05, 13.59)	8.71 (3.58, 13.75)	−0.05 (−0.26, 0.15)	231.89 (61.72, 409.84)	283.26 (112.80, 461.93)	0.31 (0.16, 0.47)
Argentina	2.22 (0.76, 4.00)	2.17 (1.24, 3.25)	−0.62 (−0.87, −0.37)	67.32 (22.93, 121.09)	87.05 (49.86, 131.29)	0.44 (0.26, 0.63)
Armenia	2.49 (1.18, 3.82)	5.83 (3.95, 7.98)	2.47 (1.75, 3.19)	94.05 (45.14, 147.42)	216.39 (143.89, 293.77)	2.74 (2.22, 3.26)
Australia	0.62 (0.06, 1.46)	0.40 (0.10, 0.82)	−1.94 (−2.18, −1.69)	20.25 (1.90, 48.26)	19.18 (4.19, 40.18)	−0.60 (−0.80, −0.40)
Austria	2.06 (1.09, 3.04)	1.23 (0.75, 1.79)	−1.32 (−1.90, −0.73)	63.96 (33.20, 97.75)	54.51 (31.18, 84.47)	−0.35 (−0.78, 0.08)
Azerbaijan	1.04 (0.42, 1.82)	4.03 (2.36, 5.89)	4.46 (3.93, 5.01)	48.54 (19.37, 85.95)	169.38 (97.89, 249.46)	4.30 (3.92, 4.68)
Bahamas	6.26 (1.55, 11.18)	5.10 (1.60, 9.04)	−1.25 (−1.52, −0.98)	199.96 (49.38, 364.08)	206.07 (60.57, 370.47)	−0.29 (−0.48, −0.10)
Bahrain	17.14 (11.93, 22.49)	30.34 (20.94, 41.59)	2.32 (1.76, 2.89)	455.17 (317.89, 602.73)	770.18 (533.12, 1046.92)	1.90 (1.50, 2.30)
Bangladesh	0.89 (0.23, 2.14)	3.15 (1.81, 4.79)	4.09 (3.58, 4.61)	24.63 (6.43, 57.56)	90.83 (52.86, 137.70)	4.45 (4.21, 4.69)
Barbados	11.44 (4.13, 17.90)	10.47 (5.05, 15.71)	−0.82 (−1.00, −0.63)	312.14 (112.36, 499.79)	318.00 (147.73, 488.60)	−0.37 (−0.52, −0.21)
Belarus	0.73 (0.40, 1.06)	0.32 (0.19, 0.48)	−4.09 (−4.56, −3.61)	51.43 (27.87, 80.47)	40.53 (22.92, 63.81)	−1.17 (−1.47, −0.87)
Belgium	1.86 (0.96, 2.80)	0.72 (0.43, 1.04)	−3.44 (−3.72, −3.16)	67.24 (33.49, 105.45)	53.02 (29.44, 82.87)	−0.95 (−1.18, −0.73)
Belize	2.81 (0.54, 6.46)	7.51 (2.56, 12.34)	2.99 (2.21, 3.78)	81.90 (15.61, 184.90)	244.66 (86.09, 409.10)	3.27 (2.61, 3.92)
Benin	0.61 (0.15, 1.49)	1.76 (0.70, 3.34)	3.67 (3.37, 3.96)	17.62 (4.40, 43.09)	54.43 (21.63, 100.82)	3.92 (3.63, 4.21)
Bermuda	2.28 (0.44, 5.44)	0.87 (0.13, 1.85)	−3.97 (−4.24, −3.69)	68.81 (13.14, 163.08)	40.21 (6.50, 84.83)	−2.51 (−2.80, −2.22)
Bhutan	0.39 (0.08, 0.99)	3.15 (1.64, 5.09)	8.76 (8.32, 9.21)	13.07 (2.82, 33.03)	99.97 (52.72, 158.45)	8.50 (8.07, 8.93)
Bolivia (Plurinational State of)	3.47 (1.25, 6.59)	5.84 (3.27, 8.99)	1.56 (1.28, 1.84)	101.93 (38.95, 190.39)	172.58 (100.74, 257.86)	1.59 (1.32, 1.86)
Bosnia and Herzegovina	1.26 (0.60, 2.16)	6.03 (3.69, 8.62)	7.06 (6.05, 8.08)	54.56 (25.50, 94.21)	211.79 (135.40, 296.68)	5.89 (5.16, 6.62)
Botswana	2.59 (1.13, 4.64)	11.46 (6.19, 17.99)	5.08 (4.35, 5.83)	67.93 (30.37, 121.09)	300.19 (169.74, 467.40)	5.13 (4.43, 5.84)
Brazil	2.14 (0.89, 3.80)	2.42 (1.47, 3.69)	0.31 (−0.03, 0.65)	76.26 (31.83, 134.26)	90.46 (52.19, 139.05)	0.56 (0.29, 0.84)
Brunei Darussalam	6.28 (1.79, 13.37)	4.27 (1.12, 8.33)	−0.54 (−1.06, −0.01)	171.12 (46.92, 364.42)	137.28 (35.37, 269.23)	−0.14 (−0.65, 0.38)
Bulgaria	2.26 (1.22, 3.33)	2.06 (1.31, 2.87)	−1.22 (−1.70, −0.75)	95.30 (49.24, 146.39)	109.03 (66.81, 157.64)	−0.04 (−0.33, 0.25)
Burkina Faso	0.63 (0.11, 1.80)	1.26 (0.35, 2.89)	2.71 (2.55, 2.87)	16.92 (2.97, 49.06)	38.11 (10.91, 85.92)	3.13 (2.98, 3.29)
Burundi	0.80 (0.17, 2.07)	0.91 (0.26, 2.09)	0.23 (−0.02, 0.47)	21.80 (4.64, 57.54)	26.40 (7.47, 60.76)	0.41 (0.14, 0.68)
Cabo Verde	0.39 (0.14, 0.79)	5.63 (3.61, 7.84)	8.84 (7.88, 9.80)	18.91 (6.70, 38.30)	187.02 (120.92, 258.72)	7.99 (7.37, 8.61)
Cambodia	0.60 (0.15, 1.50)	1.49 (0.64, 2.64)	3.55 (3.33, 3.77)	17.60 (4.36, 44.16)	54.68 (24.48, 97.22)	4.33 (4.13, 4.52)
Cameroon	2.01 (0.73, 4.02)	5.73 (2.87, 9.22)	3.65 (3.42, 3.88)	51.97 (19.41, 104.34)	156.04 (81.85, 246.86)	3.81 (3.56, 4.05)
Canada	1.03 (0.26, 2.07)	0.46 (0.19, 0.85)	−3.81 (−4.64, −2.97)	27.78 (6.42, 55.44)	20.20 (7.75, 39.07)	−1.95 (−2.42, −1.47)
Central African Republic	1.09 (0.22, 2.81)	1.68 (0.44, 3.74)	1.56 (1.41, 1.71)	33.35 (6.99, 83.37)	56.00 (15.05, 125.24)	1.85 (1.70, 2.01)
Chad	0.43 (0.07, 1.29)	1.17 (0.32, 2.64)	3.66 (3.43, 3.89)	12.84 (2.24, 37.64)	36.29 (9.96, 80.42)	3.82 (3.61, 4.03)
Chile	1.82 (0.82, 2.99)	2.72 (1.87, 3.60)	1.29 (0.99, 1.59)	67.85 (29.07, 115.36)	124.48 (79.24, 173.60)	2.10 (1.86, 2.34)
China	0.65 (0.29, 1.16)	1.74 (1.22, 2.32)	3.71 (3.26, 4.15)	32.64 (14.22, 59.13)	89.70 (59.56, 123.38)	3.82 (3.40, 4.23)
Colombia	1.91 (0.87, 3.32)	1.96 (1.24, 2.86)	−0.89 (−1.43, −0.35)	90.68 (40.08, 162.56)	133.83 (84.28, 195.94)	0.78 (0.44, 1.13)
Comoros	0.51 (0.15, 1.16)	1.24 (0.55, 2.32)	2.96 (2.82, 3.09)	13.04 (3.89, 29.75)	33.78 (14.73, 63.87)	3.16 (3.02, 3.30)
Congo	2.49 (0.79, 5.42)	6.46 (3.27, 10.07)	3.21 (2.93, 3.50)	71.80 (22.51, 152.23)	194.89 (102.99, 302.88)	3.40 (3.13, 3.68)
Cook Islands	4.20 (1.22, 11.03)	4.72 (0.89, 11.48)	0.02 (−0.26, 0.31)	111.25 (33.02, 290.90)	142.11 (26.48, 346.97)	0.53 (0.29, 0.76)
Costa Rica	1.16 (0.46, 2.13)	1.61 (0.97, 2.32)	0.36 (−0.12, 0.83)	61.69 (24.33, 116.01)	127.47 (75.44, 192.91)	2.28 (2.03, 2.53)
Croatia	1.92 (1.01, 2.80)	1.75 (1.11, 2.42)	−0.81 (−1.14, −0.49)	91.67 (45.83, 138.41)	108.11 (66.06, 155.79)	0.26 (0.08, 0.43)
Cuba	2.90 (1.10, 4.84)	1.46 (0.74, 2.32)	−2.56 (−3.27, −1.84)	134.63 (48.66, 234.89)	127.76 (60.20, 212.85)	−0.30 (−0.64, 0.05)
Cyprus	9.44 (4.02, 15.65)	3.86 (2.36, 5.54)	−3.53 (−3.87, −3.19)	189.20 (78.93, 311.52)	113.96 (68.57, 170.35)	−2.28 (−2.60, −1.97)
Czechia	2.23 (1.27, 3.09)	2.15 (1.36, 3.01)	1.79 (0.88, 2.71)	122.69 (66.58, 185.53)	151.50 (90.34, 224.34)	1.18 (0.87, 1.50)
Côte d’Ivoire	1.22 (0.33, 2.73)	2.96 (1.28, 5.18)	2.81 (2.33, 3.30)	33.41 (9.11, 74.32)	87.34 (37.99, 154.71)	3.08 (2.63, 3.53)
Democratic People’s Republic of Korea	0.65 (0.24, 1.39)	1.21 (0.68, 1.96)	2.35 (2.21, 2.48)	25.64 (9.33, 53.81)	60.23 (32.95, 95.99)	3.13 (2.97, 3.29)
Democratic Republic of the Congo	1.11 (0.27, 2.71)	1.54 (0.57, 3.00)	0.64 (−0.09, 1.38)	31.07 (7.81, 78.55)	52.09 (18.87, 102.78)	1.35 (0.64, 2.07)
Denmark	1.38 (0.61, 2.21)	0.94 (0.50, 1.51)	−1.04 (−1.78, −0.30)	44.87 (19.29, 72.38)	35.94 (18.61, 58.41)	−0.65 (−1.14, −0.17)
Djibouti	1.46 (0.43, 3.27)	6.77 (3.75, 10.22)	6.01 (5.38, 6.65)	39.80 (11.53, 89.65)	185.43 (102.29, 279.68)	6.02 (5.38, 6.66)
Dominica	5.11 (1.64, 10.15)	8.78 (3.73, 14.28)	1.39 (1.00, 1.78)	148.59 (45.64, 300.57)	311.90 (129.23, 510.48)	2.14 (1.77, 2.50)
Dominican Republic	0.84 (0.22, 1.99)	3.84 (1.55, 6.87)	6.27 (5.81, 6.74)	27.87 (7.03, 67.37)	139.81 (56.84, 244.28)	6.43 (6.05, 6.81)
Ecuador	2.01 (0.78, 3.63)	5.38 (3.24, 7.88)	3.72 (3.26, 4.19)	68.82 (27.45, 125.23)	180.64 (110.33, 256.81)	3.57 (3.14, 4.01)
Egypt	4.83 (3.39, 6.33)	8.12 (5.29, 11.36)	2.03 (1.88, 2.18)	148.38 (103.31, 199.03)	293.51 (202.60, 393.29)	2.57 (2.45, 2.68)
El Salvador	0.86 (0.29, 1.71)	5.19 (2.95, 8.02)	6.52 (6.02, 7.01)	38.04 (13.26, 77.58)	214.04 (124.13, 327.64)	6.30 (5.88, 6.72)
Equatorial Guinea	0.93 (0.18, 2.64)	10.44 (6.16, 15.72)	10.35 (9.76, 10.95)	28.65 (5.33, 79.63)	294.69 (166.61, 427.48)	9.99 (9.43, 10.56)
Eritrea	0.92 (0.20, 2.36)	2.91 (1.11, 5.52)	3.68 (3.24, 4.12)	27.07 (6.21, 69.71)	83.41 (32.97, 158.24)	3.60 (3.21, 4.00)
Estonia	0.23 (0.08, 0.46)	0.17 (0.06, 0.34)	−0.95 (−2.30, 0.42)	20.55 (6.91, 41.59)	16.03 (5.25, 31.99)	−0.58 (−1.56, 0.40)
Eswatini	3.17 (1.28, 5.85)	12.41 (6.34, 20.24)	5.00 (4.35, 5.66)	79.98 (33.11, 149.67)	310.07 (161.29, 499.35)	4.97 (4.32, 5.62)
Ethiopia	0.63 (0.14, 1.71)	1.29 (0.58, 2.40)	2.70 (2.30, 3.11)	17.31 (3.72, 47.45)	34.01 (15.18, 62.98)	2.40 (2.02, 2.78)
Fiji	4.04 (0.85, 12.74)	18.21 (4.56, 41.19)	4.86 (3.87, 5.85)	111.26 (23.71, 346.04)	486.81 (125.47, 1096.97)	4.85 (3.93, 5.78)
Finland	0.45 (0.05, 1.00)	0.09 (0.02, 0.22)	−5.75 (−6.15, −5.34)	26.43 (3.07, 62.02)	13.61 (2.32, 33.25)	−2.31 (−2.89, −1.72)
France	1.20 (0.57, 1.87)	0.75 (0.44, 1.12)	−1.54 (−2.25, −0.82)	34.74 (16.24, 55.29)	29.01 (16.62, 45.15)	−0.57 (−0.98, −0.16)
Gabon	4.03 (1.45, 8.08)	13.16 (8.28, 18.52)	4.04 (3.53, 4.56)	113.59 (42.32, 219.73)	379.37 (235.78, 538.68)	4.16 (3.66, 4.66)
Gambia	0.69 (0.16, 1.73)	2.38 (1.02, 4.43)	4.26 (4.06, 4.45)	19.37 (4.58, 48.19)	68.31 (29.22, 122.85)	4.38 (4.21, 4.54)
Georgia	0.92 (0.40, 1.60)	2.46 (1.48, 3.58)	4.43 (3.84, 5.02)	45.01 (18.82, 80.90)	121.40 (70.47, 183.58)	4.11 (3.73, 4.49)
Germany	2.57 (1.31, 3.85)	1.05 (0.64, 1.57)	−3.32 (−3.58, −3.06)	92.89 (45.61, 144.16)	65.19 (36.58, 102.63)	−1.48 (−1.73, −1.24)
Ghana	1.55 (0.63, 2.92)	5.66 (3.27, 8.45)	5.08 (4.76, 5.41)	44.51 (18.37, 85.49)	169.55 (93.92, 249.66)	5.34 (5.01, 5.66)
Greece	1.20 (0.62, 1.84)	0.66 (0.41, 0.92)	−1.28 (−1.72, −0.83)	54.50 (26.72, 87.34)	59.45 (33.91, 91.74)	0.49 (0.22, 0.76)
Greenland	0.69 (0.12, 1.98)	0.38 (0.03, 1.11)	−2.77 (−3.12, −2.42)	19.65 (3.43, 55.06)	19.41 (1.49, 57.13)	−0.53 (−0.89, −0.18)
Grenada	5.23 (1.31, 10.82)	11.78 (5.20, 17.98)	2.27 (1.68, 2.86)	157.48 (37.81, 330.17)	379.29 (163.75, 591.55)	2.49 (2.01, 2.97)
Guam	2.75 (0.41, 6.99)	1.58 (0.61, 2.87)	−2.08 (−2.86, −1.29)	83.64 (12.36, 213.50)	78.77 (30.91, 140.75)	−0.19 (−0.90, 0.53)
Guatemala	0.67 (0.19, 1.46)	5.57 (2.96, 9.18)	6.44 (5.69, 7.19)	30.61 (8.88, 68.64)	201.32 (107.51, 325.23)	6.14 (5.62, 6.65)
Guinea	0.61 (0.14, 1.65)	1.57 (0.56, 3.35)	3.56 (3.35, 3.78)	16.60 (3.77, 45.12)	45.68 (15.86, 93.99)	3.77 (3.59, 3.94)
Guinea–Bissau	1.02 (0.21, 2.55)	2.32 (0.85, 4.67)	2.89 (2.72, 3.07)	28.59 (6.01, 73.11)	67.23 (24.64, 137.48)	3.02 (2.86, 3.17)
Guyana	7.65 (1.59, 15.04)	13.54 (5.27, 22.27)	1.71 (1.06, 2.37)	241.21 (52.37, 464.06)	449.35 (181.84, 724.88)	1.96 (1.38, 2.55)
Haiti	1.14 (0.27, 3.01)	1.91 (0.67, 4.31)	2.05 (1.90, 2.19)	34.63 (8.39, 90.88)	68.17 (23.67, 149.30)	2.59 (2.45, 2.74)
Honduras	0.37 (0.11, 0.86)	1.29 (0.64, 2.18)	4.73 (4.35, 5.12)	25.12 (7.19, 61.30)	80.53 (40.09, 138.59)	4.35 (4.01, 4.69)
Hungary	1.64 (0.86, 2.47)	1.60 (1.05, 2.24)	0.11 (−0.29, 0.52)	82.81 (41.32, 132.31)	102.34 (61.11, 148.06)	0.77 (0.43, 1.11)
Iceland	0.24 (0.02, 0.64)	0.12 (0.02, 0.26)	−2.72 (−3.12, −2.31)	12.04 (0.97, 32.34)	11.05 (1.88, 26.63)	−0.24 (−0.53, 0.06)
India	1.34 (0.58, 2.45)	4.25 (2.83, 5.72)	4.33 (4.10, 4.56)	45.50 (20.25, 83.40)	165.86 (109.74, 229.54)	4.68 (4.54, 4.81)
Indonesia	1.82 (0.78, 3.31)	5.43 (3.40, 7.94)	3.74 (3.58, 3.90)	58.29 (25.96, 106.05)	168.79 (105.59, 242.80)	3.53 (3.37, 3.69)
Iran (Islamic Republic of)	2.52 (1.80, 3.28)	4.90 (3.50, 6.25)	2.65 (2.45, 2.86)	100.94 (70.57, 138.53)	212.37 (151.53, 287.80)	2.93 (2.80, 3.05)
Iraq	8.25 (5.14, 11.79)	10.48 (7.17, 13.95)	0.80 (0.66, 0.95)	257.22 (159.36, 361.01)	369.95 (256.75, 489.76)	1.24 (1.05, 1.43)
Ireland	1.11 (0.32, 2.00)	0.38 (0.16, 0.65)	−3.93 (−4.32, −3.53)	30.70 (8.83, 57.29)	23.75 (9.83, 43.71)	−1.01 (−1.23, −0.78)
Israel	2.99 (1.66, 4.41)	3.47 (2.30, 4.63)	−0.61 (−1.68, 0.48)	89.78 (49.63, 134.52)	112.98 (74.35, 157.20)	0.04 (−0.70, 0.78)
Italy	3.27 (1.84, 4.75)	1.61 (1.03, 2.24)	−2.39 (−2.53, −2.25)	99.92 (56.21, 146.28)	79.15 (48.50, 117.64)	−0.59 (−0.93, −0.25)
Jamaica	3.12 (1.15, 6.17)	9.24 (5.20, 13.95)	3.40 (2.65, 4.16)	88.41 (32.54, 173.73)	275.56 (157.36, 421.53)	3.58 (2.87, 4.31)
Japan	0.69 (0.21, 1.20)	0.24 (0.14, 0.36)	−3.67 (−4.25, −3.08)	37.48 (10.86, 66.83)	38.65 (21.26, 61.78)	−0.23 (−0.65, 0.20)
Jordan	12.81 (8.70, 17.26)	8.55 (6.05, 11.42)	−1.72 (−2.16, −1.28)	324.76 (225.05, 436.78)	273.75 (190.80, 367.91)	−0.88 (−1.24, −0.53)
Kazakhstan	0.65 (0.27, 1.13)	2.27 (1.38, 3.33)	3.21 (2.34, 4.09)	45.03 (18.25, 82.23)	125.35 (71.81, 190.37)	3.35 (2.96, 3.74)
Kenya	0.66 (0.27, 1.28)	1.81 (0.90, 2.97)	4.07 (3.74, 4.39)	17.92 (7.46, 34.39)	52.69 (27.09, 87.16)	4.35 (4.00, 4.71)
Kiribati	2.07 (0.46, 5.75)	4.59 (1.19, 11.61)	1.57 (0.88, 2.26)	63.06 (14.09, 179.73)	139.36 (36.73, 349.09)	1.61 (0.95, 2.27)
Kuwait	6.78 (4.96, 8.68)	4.43 (3.11, 5.86)	−1.70 (−2.41, −0.98)	251.87 (175.11, 337.61)	270.94 (178.21, 384.24)	0.00 (−0.45, 0.45)
Kyrgyzstan	0.41 (0.15, 0.77)	0.81 (0.45, 1.23)	1.22 (0.70, 1.75)	22.86 (8.31, 45.35)	54.88 (30.57, 86.16)	2.37 (2.04, 2.70)
Lao People’s Democratic Republic	0.69 (0.17, 1.77)	1.60 (0.74, 2.85)	2.79 (2.59, 2.99)	22.52 (5.70, 56.18)	58.48 (26.13, 102.46)	3.26 (3.07, 3.44)
Latvia	0.66 (0.34, 1.03)	0.82 (0.47, 1.28)	1.00 (0.21, 1.80)	45.23 (21.89, 72.92)	52.07 (29.00, 79.84)	0.74 (0.16, 1.32)
Lebanon	3.29 (2.09, 4.69)	3.32 (2.06, 4.77)	0.15 (−0.03, 0.33)	128.61 (78.75, 184.73)	205.72 (131.65, 290.78)	1.69 (1.54, 1.84)
Lesotho	1.81 (0.70, 3.60)	8.59 (4.23, 14.46)	6.31 (5.96, 6.67)	47.75 (18.06, 93.87)	219.80 (109.98, 363.42)	6.18 (5.84, 6.53)
Liberia	0.80 (0.22, 1.93)	1.80 (0.72, 3.53)	3.93 (3.51, 4.35)	23.82 (6.75, 59.10)	58.92 (24.17, 118.23)	4.34 (3.88, 4.80)
Libya	2.19 (1.23, 3.27)	4.07 (2.67, 5.95)	2.27 (1.99, 2.55)	106.01 (60.76, 153.50)	251.70 (161.53, 357.36)	2.86 (2.53, 3.19)
Lithuania	0.47 (0.21, 0.77)	0.34 (0.19, 0.55)	−1.05 (−1.54, −0.56)	36.42 (16.15, 61.72)	31.37 (16.40, 52.26)	−0.26 (−0.75, 0.24)
Luxembourg	1.44 (0.62, 2.35)	0.52 (0.29, 0.82)	−3.39 (−3.74, −3.05)	42.09 (16.81, 71.24)	50.00 (25.52, 83.07)	0.88 (0.55, 1.21)
Madagascar	0.36 (0.10, 0.81)	0.76 (0.32, 1.50)	2.91 (2.73, 3.08)	10.07 (2.89, 22.32)	22.79 (9.21, 44.80)	3.22 (3.06, 3.39)
Malawi	0.50 (0.11, 1.25)	0.98 (0.37, 1.94)	2.92 (2.68, 3.16)	13.52 (3.06, 34.74)	29.96 (11.39, 59.37)	3.34 (3.09, 3.59)
Malaysia	4.55 (2.39, 6.69)	1.94 (1.17, 2.92)	−3.70 (−4.27, −3.13)	163.02 (81.77, 236.65)	119.19 (71.69, 176.17)	−1.23 (−1.45, −1.02)
Maldives	1.03 (0.28, 2.41)	1.47 (0.78, 2.36)	0.68 (0.28, 1.08)	30.97 (8.38, 74.32)	60.33 (30.10, 99.69)	1.93 (1.51, 2.36)
Mali	0.56 (0.10, 1.58)	1.27 (0.37, 2.96)	3.15 (3.02, 3.28)	15.59 (2.87, 44.64)	37.45 (10.74, 84.94)	3.34 (3.24, 3.44)
Malta	3.27 (1.39, 5.34)	1.43 (0.83, 2.08)	−2.63 (−2.98, −2.28)	97.60 (42.08, 164.51)	75.97 (42.50, 118.29)	−0.71 (−1.01, −0.40)
Marshall Islands	1.53 (0.28, 4.76)	4.66 (1.17, 11.42)	3.51 (2.97, 4.05)	52.23 (9.58, 161.96)	173.72 (45.32, 430.95)	3.80 (3.26, 4.34)
Mauritania	1.87 (0.67, 3.99)	4.42 (2.34, 7.10)	2.71 (2.51, 2.90)	47.89 (17.08, 100.22)	114.35 (61.15, 184.23)	2.71 (2.53, 2.89)
Mauritius	4.44 (1.97, 7.05)	14.28 (7.03, 22.23)	6.03 (4.91, 7.16)	155.40 (68.77, 246.04)	430.04 (208.00, 661.33)	4.96 (4.10, 5.84)
Mexico	8.62 (4.79, 12.83)	9.18 (6.23, 12.64)	−0.24 (−0.50, 0.02)	286.77 (159.15, 429.71)	323.11 (212.55, 448.68)	0.03 (−0.19, 0.26)
Micronesia (Federated States of)	2.14 (0.41, 6.34)	9.03 (2.15, 23.02)	4.93 (4.43, 5.44)	61.88 (11.82, 182.86)	262.32 (60.91, 656.15)	4.99 (4.47, 5.51)
Monaco	0.26 (0.03, 0.57)	0.33 (0.17, 0.52)	1.46 (0.49, 2.45)	17.69 (1.77, 38.89)	37.59 (18.12, 62.05)	3.15 (2.31, 3.99)
Mongolia	0.26 (0.10, 0.49)	0.76 (0.46, 1.11)	3.71 (3.49, 3.92)	13.00 (5.14, 24.68)	46.75 (27.59, 67.67)	4.80 (4.68, 4.93)
Montenegro	1.78 (1.00, 2.61)	2.08 (1.28, 2.91)	0.80 (0.60, 0.99)	92.84 (48.94, 144.28)	128.45 (75.55, 187.65)	1.20 (1.07, 1.34)
Morocco	1.11 (0.49, 1.99)	4.63 (3.11, 6.47)	5.39 (5.19, 5.58)	46.52 (21.23, 78.63)	208.26 (136.96, 283.24)	5.54 (5.30, 5.78)
Mozambique	0.29 (0.05, 0.84)	0.89 (0.30, 1.87)	4.86 (4.50, 5.22)	7.88 (1.40, 22.34)	26.00 (8.90, 54.80)	5.18 (4.82, 5.54)
Myanmar	1.59 (0.48, 3.68)	3.95 (2.17, 6.16)	3.65 (3.36, 3.95)	47.39 (14.51, 111.73)	124.47 (67.73, 193.80)	3.83 (3.53, 4.12)
Namibia	2.62 (1.13, 4.69)	6.76 (3.49, 10.63)	3.13 (2.69, 3.57)	69.05 (29.98, 123.78)	179.05 (92.80, 277.90)	3.19 (2.79, 3.59)
Nauru	2.99 (0.55, 9.92)	5.38 (0.99, 14.75)	0.98 (0.59, 1.38)	83.05 (15.13, 273.97)	153.59 (28.29, 422.67)	1.09 (0.70, 1.48)
Nepal	0.37 (0.08, 0.92)	2.13 (1.12, 3.34)	6.94 (6.52, 7.36)	15.68 (3.49, 39.68)	89.43 (47.00, 142.37)	6.66 (6.41, 6.92)
Netherlands	2.73 (1.36, 4.16)	0.96 (0.58, 1.40)	−3.95 (−4.37, −3.53)	81.43 (39.83, 125.67)	44.79 (25.93, 69.41)	−2.26 (−2.44, −2.08)
New Zealand	0.49 (0.03, 1.27)	0.28 (0.04, 0.62)	−3.15 (−3.73, −2.58)	17.11 (1.14, 43.92)	13.63 (2.15, 31.66)	−1.88 (−2.27, −1.50)
Nicaragua	0.72 (0.21, 1.64)	3.54 (1.74, 5.97)	5.42 (4.89, 5.96)	28.26 (8.19, 66.75)	125.68 (60.17, 212.38)	5.24 (4.90, 5.58)
Niger	0.50 (0.07, 1.59)	0.96 (0.20, 2.66)	2.34 (2.10, 2.59)	12.56 (1.84, 39.21)	26.37 (5.39, 73.27)	2.60 (2.39, 2.80)
Nigeria	1.33 (0.50, 2.82)	4.18 (2.42, 6.39)	4.27 (4.12, 4.41)	33.83 (12.96, 71.00)	104.56 (58.42, 160.25)	4.12 (3.99, 4.25)
Niue	2.56 (0.77, 6.37)	5.76 (1.07, 14.46)	2.39 (1.96, 2.82)	77.79 (24.04, 189.61)	185.55 (33.35, 451.56)	2.60 (2.22, 2.99)
North Macedonia	2.88 (1.67, 4.31)	6.08 (4.05, 8.37)	3.03 (2.46, 3.60)	113.79 (62.19, 170.89)	227.88 (149.45, 309.71)	2.65 (2.28, 3.03)
Northern Mariana Islands	3.49 (1.01, 8.06)	3.75 (1.77, 6.51)	0.39 (0.04, 0.75)	109.72 (30.53, 247.47)	132.29 (60.65, 229.17)	0.68 (0.39, 0.97)
Norway	0.66 (0.18, 1.26)	0.23 (0.07, 0.46)	−3.75 (−4.30, −3.20)	36.19 (9.57, 70.84)	18.14 (5.70, 36.64)	−3.00 (−3.32, −2.69)
Oman	5.32 (2.70, 8.69)	13.41 (9.54, 17.56)	3.12 (2.56, 3.69)	149.77 (77.44, 240.55)	371.16 (264.46, 488.93)	2.83 (2.20, 3.45)
Pakistan	0.99 (0.26, 2.21)	6.35 (3.69, 9.38)	7.23 (6.90, 7.56)	33.96 (9.46, 74.48)	198.63 (118.85, 288.93)	6.91 (6.63, 7.20)
Palau	3.67 (0.02, 10.87)	5.51 (0.02, 15.00)	2.18 (1.41, 2.95)	109.04 (0.69, 321.91)	172.82 (0.52, 461.94)	2.39 (1.62, 3.16)
Palestine	5.43 (2.46, 9.08)	14.43 (10.15, 18.99)	2.94 (2.64, 3.23)	141.02 (65.78, 233.55)	398.78 (280.19, 529.43)	3.18 (2.89, 3.46)
Panama	1.32 (0.49, 2.52)	3.06 (1.67, 4.82)	3.02 (2.46, 3.57)	58.12 (20.87, 114.53)	136.01 (72.30, 216.25)	3.26 (2.84, 3.69)
Papua New Guinea	1.23 (0.20, 4.04)	2.56 (0.57, 6.93)	2.38 (2.25, 2.51)	36.55 (6.05, 120.26)	79.05 (17.47, 221.46)	2.54 (2.40, 2.67)
Paraguay	0.88 (0.33, 1.75)	3.33 (1.65, 5.64)	4.73 (4.06, 5.39)	29.00 (11.11, 59.11)	102.27 (52.86, 170.47)	4.31 (3.80, 4.82)
Peru	1.25 (0.54, 2.20)	2.39 (1.46, 3.56)	2.46 (2.06, 2.86)	45.24 (19.21, 78.05)	101.38 (63.04, 142.77)	2.88 (2.55, 3.22)
Philippines	2.78 (1.42, 4.54)	3.51 (2.17, 5.23)	0.12 (−0.22, 0.46)	77.17 (40.89, 127.36)	113.08 (69.86, 168.43)	0.60 (0.30, 0.90)
Poland	1.91 (1.13, 2.73)	1.79 (1.21, 2.38)	0.01 (−0.31, 0.33)	100.46 (55.90, 149.53)	121.60 (77.28, 170.04)	0.83 (0.50, 1.16)
Portugal	1.83 (0.52, 3.50)	0.96 (0.47, 1.60)	−2.79 (−3.45, −2.12)	60.09 (17.54, 117.89)	45.00 (20.71, 79.43)	−1.28 (−1.81, −0.74)
Puerto Rico	2.43 (0.01, 7.32)	1.90 (0.36, 3.86)	−1.66 (−2.02, −1.31)	80.64 (0.41, 244.18)	78.95 (15.87, 160.53)	−0.78 (−1.11, −0.44)
Qatar	26.96 (18.84, 36.18)	29.68 (20.49, 41.50)	0.78 (0.23, 1.32)	612.07 (431.77, 800.04)	722.09 (498.23, 988.83)	0.82 (0.32, 1.32)
Republic of Korea	3.13 (1.68, 4.52)	3.01 (2.03, 3.92)	−1.03 (−1.90, −0.15)	118.16 (61.53, 173.26)	121.62 (81.02, 168.61)	−0.64 (−1.14, −0.15)
Republic of Moldova	0.61 (0.26, 1.03)	0.54 (0.30, 0.83)	−0.68 (−1.14, −0.21)	41.02 (17.42, 71.67)	55.82 (28.36, 89.79)	1.47 (0.99, 1.95)
Romania	0.75 (0.36, 1.18)	0.79 (0.50, 1.14)	0.19 (−0.07, 0.46)	44.67 (19.64, 74.77)	63.70 (38.05, 93.36)	1.22 (1.06, 1.38)
Russian Federation	0.58 (0.26, 0.94)	0.74 (0.37, 1.18)	0.79 (0.12, 1.46)	36.93 (16.04, 61.84)	38.81 (19.28, 63.72)	0.28 (0.11, 0.45)
Rwanda	1.17 (0.29, 3.00)	1.84 (0.71, 3.54)	1.09 (0.71, 1.47)	31.32 (7.75, 75.95)	50.00 (19.63, 97.64)	1.12 (0.72, 1.52)
Saint Kitts and Nevis	3.68 (0.83, 8.33)	3.93 (1.53, 6.98)	0.02 (−0.23, 0.27)	109.40 (24.64, 249.91)	136.63 (53.10, 242.24)	0.35 (0.11, 0.60)
Saint Lucia	6.90 (2.03, 14.17)	9.97 (4.55, 15.34)	0.03 (−0.42, 0.48)	209.13 (62.51, 424.82)	369.05 (158.26, 582.11)	1.16 (0.83, 1.49)
Saint Vincent and the Grenadines	6.97 (1.75, 14.34)	12.82 (5.37, 20.09)	1.33 (0.88, 1.77)	196.97 (49.08, 404.86)	413.60 (166.85, 667.25)	1.83 (1.40, 2.25)
Samoa	2.34 (0.52, 6.59)	3.83 (0.96, 9.22)	1.26 (0.99, 1.53)	69.72 (15.76, 193.06)	124.13 (30.63, 303.80)	1.54 (1.30, 1.79)
San Marino	0.77 (0.17, 1.47)	0.51 (0.18, 0.93)	−1.53 (−1.77, −1.29)	31.56 (7.05, 61.34)	36.16 (13.17, 65.40)	0.26 (−0.01, 0.53)
Sao Tome and Principe	0.32 (0.11, 0.71)	1.33 (0.63, 2.27)	5.45 (5.21, 5.70)	13.54 (4.47, 30.05)	60.39 (27.16, 106.10)	5.78 (5.54, 6.01)
Saudi Arabia	3.05 (1.57, 5.09)	4.64 (3.29, 6.21)	0.07 (−0.71, 0.85)	109.49 (56.78, 180.22)	251.53 (171.91, 342.30)	1.87 (1.27, 2.47)
Senegal	1.08 (0.30, 2.49)	2.68 (1.15, 5.11)	2.93 (2.60, 3.26)	34.15 (9.85, 78.03)	87.09 (37.89, 158.98)	3.16 (2.87, 3.46)
Serbia	2.46 (1.30, 3.83)	3.69 (2.43, 5.09)	1.77 (1.42, 2.12)	100.06 (52.61, 157.56)	166.21 (106.74, 232.14)	1.90 (1.65, 2.15)
Seychelles	1.63 (0.79, 2.58)	3.43 (1.75, 5.11)	2.20 (1.87, 2.54)	76.09 (36.16, 123.40)	188.11 (96.53, 296.31)	2.80 (2.47, 3.14)
Sierra Leone	0.61 (0.16, 1.45)	1.46 (0.52, 2.99)	3.46 (3.26, 3.66)	15.06 (3.76, 37.68)	38.90 (14.43, 80.45)	3.77 (3.55, 3.99)
Singapore	2.93 (1.07, 4.61)	0.39 (0.23, 0.55)	−7.25 (−8.25, −6.25)	128.25 (44.02, 211.29)	76.38 (40.81, 121.16)	−1.89 (−2.12, −1.66)
Slovakia	2.40 (1.35, 3.35)	1.33 (0.82, 1.88)	−1.95 (−2.05, −1.85)	104.40 (57.68, 154.03)	91.09 (54.93, 132.80)	−0.52 (−0.58, −0.46)
Slovenia	1.74 (0.95, 2.57)	1.05 (0.65, 1.54)	−4.06 (−5.03, −3.08)	83.39 (43.90, 126.76)	77.75 (45.75, 115.83)	−1.36 (−1.81, −0.91)
Solomon Islands	0.64 (0.10, 2.03)	2.62 (0.66, 7.08)	4.70 (4.53, 4.87)	20.88 (3.38, 65.92)	85.95 (21.93, 230.67)	4.77 (4.63, 4.91)
Somalia	0.29 (0.04, 0.92)	0.38 (0.06, 1.14)	1.46 (1.13, 1.78)	8.05 (1.10, 25.71)	11.32 (1.80, 34.19)	1.61 (1.29, 1.93)
South Africa	5.21 (3.43, 7.19)	11.41 (7.86, 14.99)	3.26 (2.82, 3.70)	148.70 (98.19, 203.64)	313.84 (211.13, 414.79)	3.06 (2.69, 3.44)
South Sudan	0.85 (0.23, 1.99)	1.47 (0.56, 2.90)	2.24 (2.10, 2.38)	22.84 (6.35, 52.81)	41.67 (15.81, 81.38)	2.41 (2.28, 2.54)
Spain	1.96 (0.74, 3.45)	0.68 (0.38, 1.05)	−3.77 (−4.13, −3.41)	69.03 (24.84, 126.83)	46.76 (23.88, 77.59)	−1.61 (−2.01, −1.20)
Sri Lanka	1.40 (0.54, 2.69)	6.26 (3.40, 9.85)	6.19 (5.38, 7.01)	48.87 (18.61, 94.02)	206.71 (113.73, 324.69)	5.77 (5.11, 6.44)
Sudan	0.34 (0.09, 0.82)	2.24 (1.18, 3.57)	7.37 (7.02, 7.71)	15.40 (4.28, 36.31)	122.32 (68.54, 189.78)	8.01 (7.77, 8.25)
Suriname	3.67 (1.14, 6.70)	6.59 (3.16, 10.40)	2.11 (1.79, 2.44)	139.42 (41.89, 262.31)	290.84 (138.23, 462.31)	2.74 (2.51, 2.98)
Sweden	0.69 (0.11, 1.43)	0.24 (0.06, 0.53)	−3.60 (−4.17, −3.02)	26.58 (4.56, 57.59)	11.95 (2.62, 27.39)	−2.57 (−3.10, −2.04)
Switzerland	2.04 (0.93, 3.23)	0.56 (0.31, 0.88)	−4.90 (−5.23, −4.57)	65.44 (30.01, 107.89)	35.41 (18.34, 59.52)	−2.23 (−2.44, −2.01)
Syrian Arab Republic	3.39 (2.10, 4.86)	3.38 (2.32, 4.83)	−0.79 (−1.14, −0.43)	133.65 (87.61, 187.56)	180.74 (116.38, 254.09)	0.57 (0.36, 0.77)
Taiwan (Province of China)	4.02 (2.07, 6.34)	4.84 (3.22, 6.80)	−0.52 (−1.34, 0.30)	124.13 (61.78, 201.06)	168.29 (111.77, 234.88)	0.18 (−0.45, 0.81)
Tajikistan	0.72 (0.22, 1.48)	4.51 (2.23, 7.06)	7.09 (6.57, 7.62)	32.20 (10.15, 67.79)	175.05 (89.23, 267.15)	6.35 (5.93, 6.77)
Thailand	2.35 (1.07, 4.02)	3.07 (1.95, 4.50)	0.09 (−0.22, 0.41)	78.37 (36.19, 133.65)	137.31 (89.50, 191.41)	1.13 (0.84, 1.43)
Timor–Leste	0.26 (0.05, 0.76)	0.94 (0.37, 1.87)	5.70 (4.97, 6.43)	8.42 (1.70, 24.46)	36.83 (14.27, 73.27)	6.28 (5.60, 6.96)
Togo	0.83 (0.24, 1.87)	2.03 (0.91, 3.64)	2.97 (2.65, 3.29)	21.95 (6.62, 48.66)	56.26 (24.99, 101.12)	3.13 (2.82, 3.44)
Tokelau	3.69 (0.04, 12.65)	4.21 (0.01, 11.89)	0.00 (−0.25, 0.25)	109.21 (1.24, 363.21)	130.86 (0.36, 372.62)	0.16 (−0.09, 0.41)
Tonga	2.46 (0.57, 6.84)	6.20 (1.64, 14.36)	3.09 (2.71, 3.47)	69.63 (17.02, 198.35)	184.50 (48.54, 422.97)	3.22 (2.89, 3.55)
Trinidad and Tobago	19.31 (4.68, 31.48)	16.77 (7.04, 27.28)	−1.01 (−1.25, −0.77)	573.62 (143.29, 954.47)	529.64 (221.82, 841.80)	−0.81 (−1.04, −0.58)
Tunisia	1.75 (1.03, 2.61)	3.12 (1.97, 4.55)	2.14 (1.89, 2.39)	90.86 (53.32, 133.55)	197.98 (128.09, 280.65)	2.80 (2.49, 3.10)
Turkey	6.11 (4.02, 8.46)	4.51 (2.96, 6.18)	−0.79 (−1.08, −0.49)	172.98 (115.12, 236.99)	172.00 (114.80, 236.98)	0.62 (0.26, 0.98)
Turkmenistan	1.66 (0.73, 2.50)	3.04 (1.80, 4.54)	1.32 (0.89, 1.75)	80.14 (34.86, 123.37)	155.01 (93.03, 225.18)	1.90 (1.62, 2.18)
Tuvalu	1.10 (0.22, 3.41)	3.73 (0.90, 9.91)	3.70 (3.28, 4.13)	32.37 (6.70, 100.25)	114.00 (28.50, 295.28)	3.88 (3.50, 4.25)
Uganda	0.59 (0.12, 1.58)	1.83 (0.77, 3.51)	4.47 (4.25, 4.70)	16.07 (3.42, 43.87)	54.36 (23.32, 102.30)	4.81 (4.58, 5.05)
Ukraine	0.57 (0.28, 0.88)	0.40 (0.22, 0.61)	−2.15 (−2.51, −1.79)	45.13 (20.99, 73.29)	43.51 (22.68, 69.26)	−0.34 (−0.61, −0.06)
United Arab Emirates	17.35 (11.96, 23.85)	12.77 (8.55, 17.81)	−1.00 (−1.73, −0.27)	433.46 (304.93, 580.85)	430.73 (300.60, 588.00)	0.02 (−0.46, 0.50)
United Kingdom	1.18 (0.53, 1.88)	0.37 (0.20, 0.58)	−4.37 (−4.52, −4.22)	54.03 (22.87, 88.21)	51.31 (25.79, 86.05)	−0.04 (−0.23, 0.14)
United Republic of Tanzania	0.52 (0.15, 1.19)	1.36 (0.61, 2.51)	3.78 (3.62, 3.94)	12.68 (3.67, 29.43)	37.12 (16.61, 67.81)	4.31 (4.11, 4.51)
United States of America	1.57 (0.59, 2.73)	0.74 (0.34, 1.31)	−3.43 (−4.16, −2.70)	72.66 (25.71, 128.55)	45.80 (19.99, 82.10)	−1.52 (−1.89, −1.14)
United States Virgin Islands	2.99 (0.36, 7.55)	3.22 (1.40, 5.53)	0.08 (−0.35, 0.51)	94.01 (11.25, 237.80)	122.65 (51.66, 208.24)	0.69 (0.29, 1.09)
Uruguay	1.21 (0.28, 2.60)	1.17 (0.52, 2.00)	−0.16 (−0.39, 0.08)	29.01 (6.83, 62.34)	42.09 (18.52, 72.23)	1.20 (0.77, 1.63)
Uzbekistan	0.99 (0.38, 1.68)	6.67 (4.27, 9.36)	6.42 (5.68, 7.16)	46.57 (18.11, 80.26)	245.07 (157.88, 346.15)	5.69 (5.18, 6.21)
Vanuatu	0.73 (0.15, 2.14)	2.30 (0.60, 5.64)	3.91 (3.74, 4.08)	24.57 (5.03, 69.42)	78.57 (21.00, 190.00)	3.92 (3.77, 4.07)
Venezuela (Bolivarian Republic of)	4.94 (2.22, 7.74)	6.64 (3.98, 9.74)	0.90 (0.75, 1.05)	177.21 (80.19, 281.96)	253.75 (153.31, 367.41)	1.23 (1.10, 1.37)
Viet Nam	1.01 (0.34, 2.13)	3.54 (2.06, 5.24)	5.00 (4.69, 5.31)	27.91 (9.94, 59.55)	109.38 (62.04, 162.63)	5.37 (5.05, 5.70)
Yemen	0.33 (0.07, 0.81)	1.77 (0.84, 3.03)	6.81 (6.49, 7.12)	13.46 (3.00, 33.76)	84.92 (42.64, 139.91)	7.26 (6.95, 7.56)
Zambia	0.98 (0.30, 2.09)	2.71 (1.29, 4.70)	3.57 (3.44, 3.70)	26.24 (8.13, 55.58)	76.85 (36.53, 132.72)	3.79 (3.66, 3.93)
Zimbabwe	1.23 (0.48, 2.26)	2.61 (1.16, 4.57)	2.76 (2.26, 3.26)	36.95 (14.71, 67.46)	81.24 (35.98, 147.20)	2.78 (2.32, 3.24)

### 3.2 Regional and national variations in T2D burden attributable to APMP: a 2019 comparative analysis

In 2019, Southern Sub-Saharan Africa had the highest DALY and death rates related to T2D caused by APMP exposure (Supplementary Table 1). South Asia experienced a significant increase in the age-standardized DALY rate for APMP-attributable T2D since 1990, while Central Asia saw the largest rise in ASDR. Conversely, High-income North America, Western Europe, and Australasia reported notable reductions in age-standardized death and DALY rates of APMP-attributable T2D over the past 30 years (Supplementary Figure S1 and Supplementary Table 4).

Qatar had the highest ASDR and DALY rates related to APMP in 1990 at the national level. However, by 2019, Bahrain emerged as having the highest rates, closely followed by Qatar and Fiji. Finland had the lowest ASDR for T2D attributable to APMP in 2019, while Iceland had the lowest age-standardized DALY rate. Equatorial Guinea experienced the most significant increases in both ASDR and age-standardized DALY rates due to APMP exposure over three decades. On the other hand, Singapore and Norway demonstrated the most substantial decreases in age-standardized death and DALY rates, respectively.

### 3.3 Age- and sex-specific T2D burden attributable to particulate matter pollution

**Figure 4:**
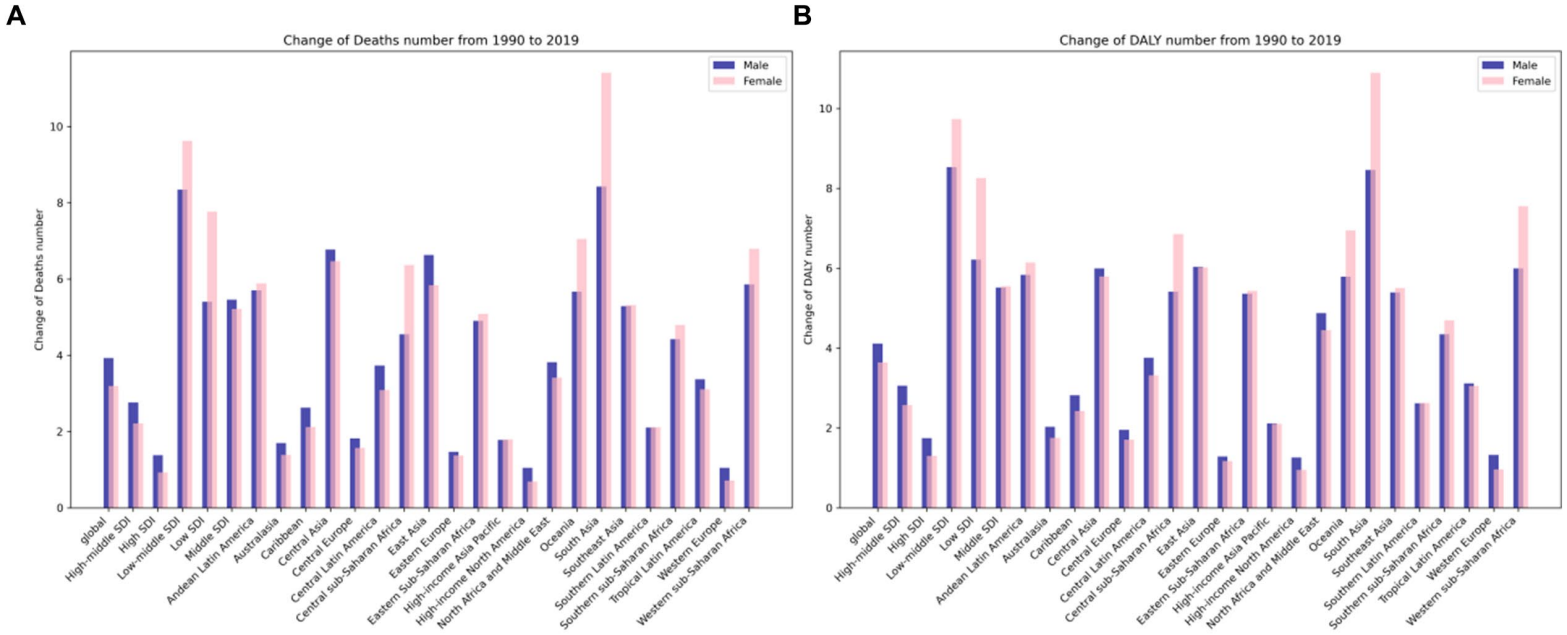
Global heatmap of type 2 diabetes mellitus rates attributed to ambient particulate matter pollution in 2019 by country and age group: **(A)** age-standardized death rate, **(B)** age-standardized DALY rate. DALY, disability-adjusted life-year.

Figure 4 illustrates that the highest burden of APMP is primarily seen in individuals aged 85 and older. There has been a notable increase in this burden across all age groups since 1990. In 2019, the DALY rate for T2D related to APMP showed a significant rise starting at age 55, with the ASDR increasing notably from age 75. Gender analysis for 2019 indicates that males consistently carry a greater burden of T2D linked to APMP across various age groups, as shown in Figure 1. The escalation in T2D burden due to APMP was more rapid in males, with the male ASDR EAPC at 1.81 (95% CI: 1.71 to 1.92), compared to females at 1.22 (95% CI: 1.16 to 1.28). Likewise, the EAPC for the male age-standardized DALY rate was 2.37 (95% CI: 2.29 to 2.44), higher than the female rate of 2.04 (95% CI: 1.99 to 2.09). Death rates from T2D due to APMP increased with age for both genders, peaking in the over-85 age group. A similar pattern was observed for DALY rates, with the highest rates seen in the 85+ age group (Figure 4).

### 3.4 Association of T2D burden attributable to particulate matter pollution and SDI

The relationship between the SDI and the burden of T2D attributed to APMP is complex and varies significantly. As shown in Supplementary Figure S1, the ASDR and the age-standardized DALY rate for T2D caused by APMP initially rise in countries with an SDI below 0.6. However, these rates begin to decrease as the SDI increases, reaching their highest levels in countries with a moderate SDI. Over the past 30 years, only high SDI countries have seen a reduction in the burden of T2D attributed to APMP, while low-to-middle SDI countries have experienced the most significant increases (see Table 1 and Supplementary Table 9).

In 2019, regions with middle SDI had the highest ASDR and DALY rates, while regions with high SDI had the lowest rates (Table 1). High SDI regions experienced the most substantial decline in ASDR from 1990 to 2019, with an EAPC of −2.44 (95% CI: −2.80 to −2.07). Conversely, regions with low-middle SDI saw the largest increase in ASDR, with an EAPC of 4.79 (95% CI: 4.68 to 4.91). Likewise, high SDI regions exhibited the most significant decrease in the EAPC of age-standardized DALY rate, while low-middle SDI regions showed the largest percentage increase (Table 1 and Supplementary Table 9).

Gaussian process regression analysis indicates that predicted rates of ASDR and age-standardized DALY rate for T2D deaths due to APMP vary, with changes in SDI influencing this variation. In regions with an SDI below 0.61, both ASDR and age-standardized DALY rate decrease as SDI increases. However, in the SDI range of 0.61 to 0.69, the rates gradually rise. For regions with an SDI between 0.7 and 0.81, the rates decrease again, while regions with an SDI above 0.81 show an upward trend in these rates (refer to Figures 1, 4). Notably, South Sub-Saharan Africa, North Africa, and the Middle East have higher than expected ASDRs, whereas Western Europe displays lower rates. This pattern in ASDR is consistent with the observed pattern in age-standardized DALY rates.

## 4 Discussion

This study aimed to estimate the global burden of Type 2 Diabetes attributable to APMP over a 30-year period. Over this time period, there was a notable increase in the absolute number of deaths and DALYs associated with APMP. This increase can be attributed to two main factors. Firstly, there was a rise in global population-weighted PM2.5 levels, reaching a peak of 44.2 μg/m^3^ in 2015 (20), particularly between 2010 and 2015. Secondly, the combined effects of population growth and aging further intensified the negative impact of air pollution on health. It is important to highlight that the age-standardized death and DALY rates related to APMP exposure showed diverse trends. Since 1990, these rates have demonstrated a significant increase, likely influenced by factors such as coal burning, urbanization, and the proliferation of vehicles and industrial factories, which vary across countries (21).

Our 30-year global analysis of 204 countries and regions found that the burden of T2D linked to APMP in 2019 remained substantial, especially among men, the older adult, and in regions with middle SDI. The T2D burden attributed to APMP has risen worldwide, with notable increases seen in low-middle and middle SDI regions such as Bhutan, Cabo Verde, and Equatorial Guinea.

The correlation between APMP and T2D in our study is supported by previous research findings. PM2.5, a significant component of APMP, has been demonstrated to trigger systemic inflammation, elevate oxidative stress, and hinder endothelial function, all of which are mechanisms that play a role in the development of T2D (22). Furthermore, animal studies have demonstrated a direct association between PM2.5 exposure and the development of insulin resistance, a key feature of T2D (23). In patients with diabetes mellitus, exposure to ambient particulate matter, notably PM2.5, has been implicated in the exacerbation of systemic oxidative stress and inflammatory responses. These processes play pivotal roles in the pathogenesis of atherosclerosis. Reflecting on the study by Muresan et al. (24), which elucidates the oxidative stress dynamics in pregnancy through elevated markers like malondialdehyde (MDA), a parallel can be drawn to the diabetic context. In diabetes, particulate matter exposure can intensify oxidative stress, leading to vascular endothelial dysfunction and the progression of atherosclerotic changes (25).

Moreover, the study’s insights into homocysteine’s role in vascular pathology provide a valuable perspective. Elevated homocysteine levels, associated with vascular complications, highlight the potential exacerbation of hyperhomocysteinemia through environmental pollutants, further elevating the atherosclerosis risk in diabetic individuals. Thus, the intersection of air pollution and metabolic disturbances in diabetes underscores the imperative to address particulate exposure as a significant risk factor in the atherosclerotic paradigm within this population.

Long-term exposure to air pollution has consistently been linked to higher morbidity and mortality rates of T2D in cohort studies (1, 24). Furthermore, short-term increases in air pollution concentrations have been associated with elevated hospitalization rates for T2D (26). Our study specifically examined deaths related to type 2 diabetes (T2D) attributable to air pollution, revealing a significant increase in such deaths among individuals aged 85 years and older. This trend is likely a result of the cumulative effects of long-term exposure to air pollution, as well as the presence of multiple comorbidities and prevalent age-related diseases in this older adult population.

Our findings indicate a greater burden of T2D resulting from APMP in men compared to women, aligning with previous studies (8). Several potential factors may account for this gender disparity. Men often have prolonged exposure to outdoor environments and higher rates of inhaling particulate matter, potentially increasing their susceptibility to APMP-induced vascular inflammation (27). Furthermore, men commonly exhibit elevated levels of insulin resistance and are more likely to have additional T2D risk factors like smoking (28, 29), potentially contributing to this gender-specific variation. Our previous research has also highlighted sex-specific links between heavy metal exposure (a component of APMP) and metabolic conditions (30), underscoring the importance of further exploring these gender-specific effects.

The burden of T2D caused by APMP varies significantly across different geographical regions. There is a notable difference in age-standardized DALY rates and ASDR between countries with the highest and lowest SDI rankings, with a 15-fold and 27-fold difference, respectively (see Supplementary Table 9). This disparity underscores the presence of social and spatial inequalities in the prevention of type 2 diabetes, healthcare provision, and air pollution control. In countries with high SDI rankings, the burden of type 2 diabetes linked to APMP has been gradually decreasing since the 1990s. This decline can be attributed to increased awareness of health and environmental issues, improved access to medical care, regular physical activity (31), and advancements in diabetes monitoring and management. Conversely, low SDI countries, particularly in Africa, the Middle East, and parts of Asia, continue to grapple with a higher burden of type 2 diabetes, indicating the necessity for comprehensive strategies to combat the disease.

The age-standardized DALY rate and ASDR of T2D burden due to APMP follow the trend of SDI. Developed countries have seen a decrease in T2D burden, while low SDI countries, with higher APMP exposure and limited health awareness, face a heavier burden (32). The rise in T2D burden in these regions from 1990 to 2019 can be partially linked to increased APMP emissions from factors like car ownership, urbanization, and reduced green spaces. In areas with moderate SDI, the adverse health effects of environmental pollution may outweigh the benefits of healthcare and education. Moreover, under-reporting in low SDI countries, due to limited medical resources, could lead to an underestimation of the T2D burden. In high SDI countries, greater health literacy, improved healthcare systems, and better air quality are contributing to the decrease in T2D burden. This disparity highlights the critical need for T2D prevention and air quality control, especially in low SDI countries (33).

It is important to acknowledge the limitations of our study. Our research highlights significant associations between regional T2D burdens and APMP levels, providing crucial insights into geographical areas where high pollution coincides with increased T2D prevalence. However, it is important to recognize that, given the ecological nature of our study, these findings do not establish causality. The study design, based on aggregate data, limits our ability to link individual T2D cases directly to specific levels of APMP exposure, and we cannot rule out the influence of other confounding factors associated with regional differences, such as rapid industrialization, globalization, lifestyle changes, and improved disease diagnosis rates. Therefore, our conclusions should be viewed as identifying potential hypotheses for future research rather than definitive evidence of causality. We suggest that subsequent analytical studies with individual-level data are necessary to explore the complex interplay between air pollution and T2D, control for personal confounders, and ascertain a direct causal relationship. The observed trends underscore the urgency of investigating air pollution’s health impacts, particularly in rapidly industrializing regions of Asia, Africa, and South America, where both pollution levels and chronic disease burdens are escalating. In some low-income countries, there may be misclassification due to a lack of air pollution monitoring and detailed T2D data. Efforts were made to mitigate these effects by using GBD data. However, the impact of various sources of air pollution on T2D, such as vehicle emissions and biomass combustion, could not be evaluated due to data unavailability (34). We acknowledge that despite these efforts, the completeness and precision of the data may still be limited in some regions. Future studies should aim to improve data collection and monitoring techniques, particularly in underrepresented areas, to enable more accurate evaluations of air pollution’s health impacts. Additionally, our secondary analysis of GBD2019 data did not consider other significant diabetes risk factors like occupation, ethnicity, and diet. It is important to note that our analysis focused solely on the burden of T2D attributed to APMP and did not include other pollutants like PM10, NO2, SO2, or the specific complications of T2D.

## 5 Conclusion

This study emphasizes a significant increase in the global burden of T2D linked to APMP from 1990 to 2019. Regions with high burdens included Southern sub-Saharan Africa, Central Latin America, North Africa, and the Middle East. Notable countries with the highest burdens were Trinidad and Tobago, Qatar, and Bahrain, while Bhutan, Sudan, and Equatorial Guinea experienced rapid increases. The rise in disease burden was particularly notable in men, older adult populations, and regions with low to middle SDI levels. These findings underscore the significant health and societal costs associated with exposure to APMP. The research offers valuable insights into regions heavily impacted by T2D due to high air pollution levels, aiding in the development of targeted interventions and policy measures to reduce pollution levels. The results stress the urgent need for further studies to create effective strategies for controlling air pollution and alleviating the increasing burden of T2D, especially among vulnerable populations. Ultimately, addressing APMP as a key environmental risk factor is crucial for reducing the global health impact of T2D, particularly in low to middle SDI regions where the burden is most severe.

## Data availability statement

The raw data supporting the conclusions of this article will be made available by the authors, without undue reservation.

## Author contributions

YS: Formal analysis, Methodology, Writing – original draft. SW: Writing – review & editing, Supervision, Visualization.

## Footnotes

^1^https://vizhub.healthdata.org/gbd-compare/

^2^http://ghdx.healthdata.org/gbd-results-tool
