# Supplementary material for: Type 2 diabetes attributable to ambient particulate matter pollution: a global burden study from 1990 to 2019
Source: Front Public Health. 2024 May 20;12:1371253. doi: 10.3389/fpubh.2024.1371253 (PMC11144887; doi:10.3389/fpubh.2024.1371253)
Supplement: Supplementary file 1 [file Data_Sheet_1.docx]

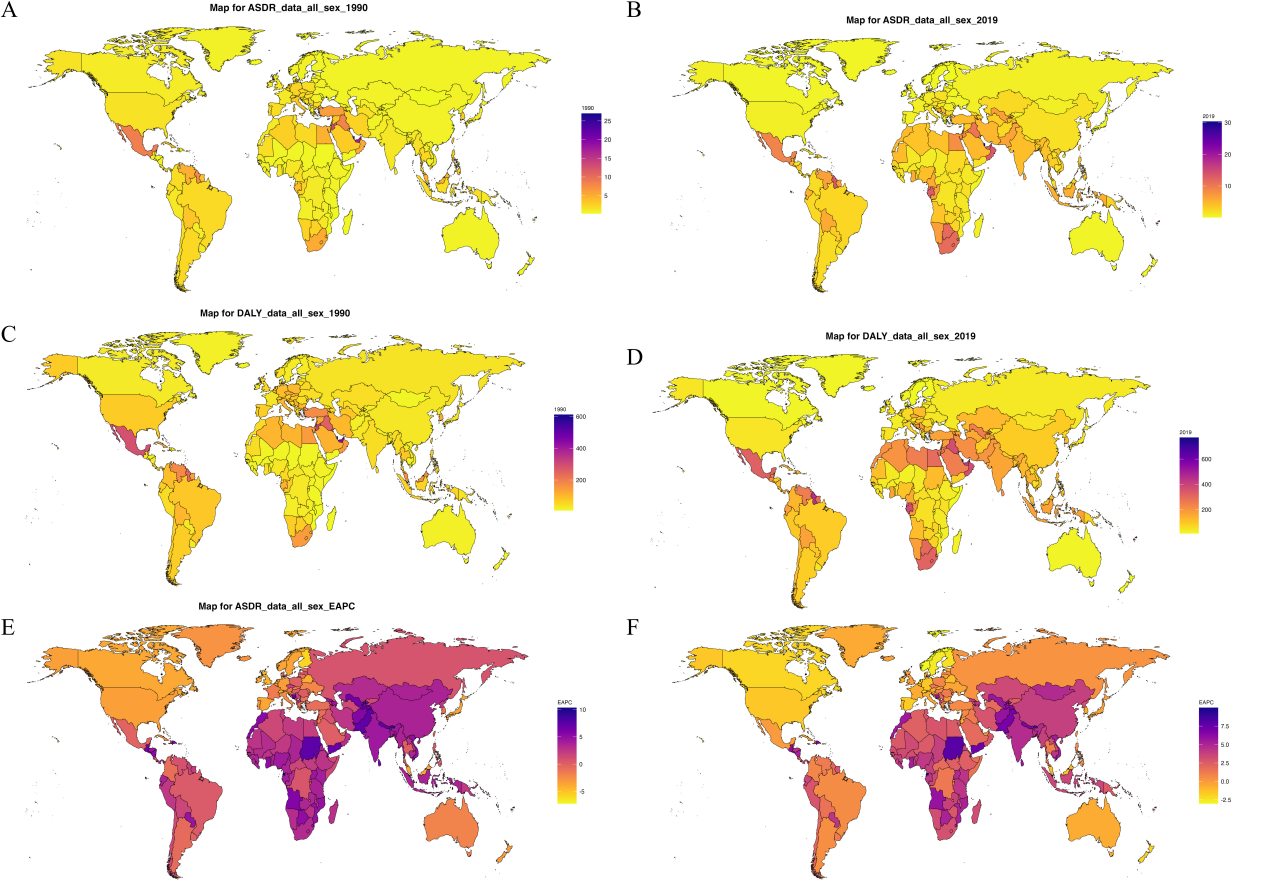


**Figure S1.** Global Disparities in Type 2 Diabetes Mellitus Attributed to Ambient particulate matter pollution from 1990 to 2019 in all Sex. (C) Age-Standardized DALY Rate in 1990, (D) Age-Standardized DALY Rate in 2019, (A) ASDR in 1990, (D) Age-Standardized Death Rate in 2019, (E) EAPCs in ASDR, (F) EAPCs in Age-Standardized DALY Rate. DALY = Disability-Adjusted Life-Year; ASDR = Age-Standardized Death Rate; EAPCs = Estimated Annual Percentage Changes.


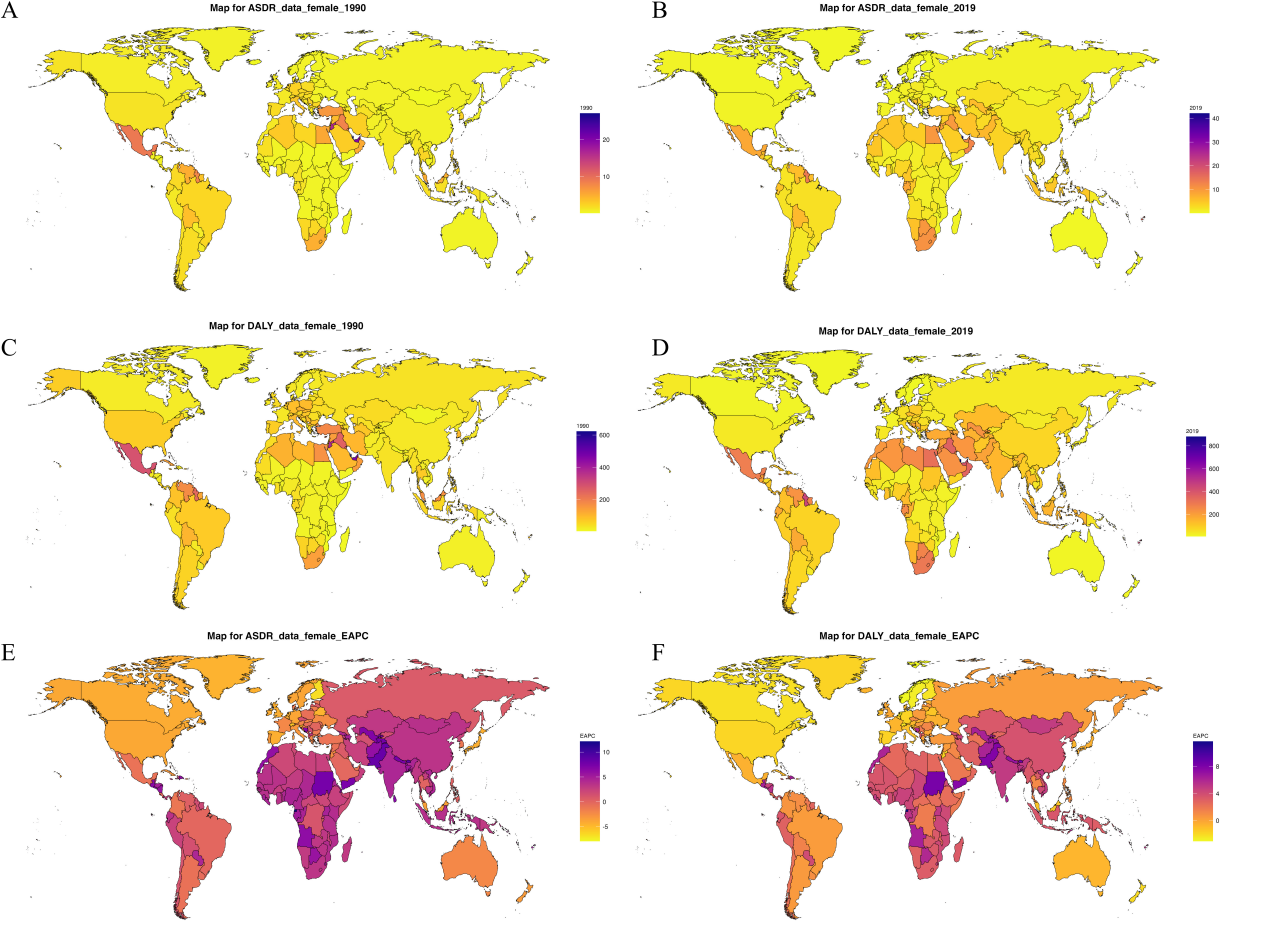


**Figure S2.** Global Disparities in Type 2 Diabetes Mellitus Attributed to Ambient particulate matter pollution from 1990 to 2019 in Female. (C) Age-Standardized DALY Rate in 1990, (D) Age-Standardized DALY Rate in 2019, (A) ASDR in 1990, (D) Age-Standardized Death Rate in 2019, (E) EAPCs in ASDR, (F) EAPCs in Age-Standardized DALY Rate. DALY = Disability-Adjusted Life-Year; ASDR = Age-Standardized Death Rate; EAPCs = Estimated Annual Percentage Changes.


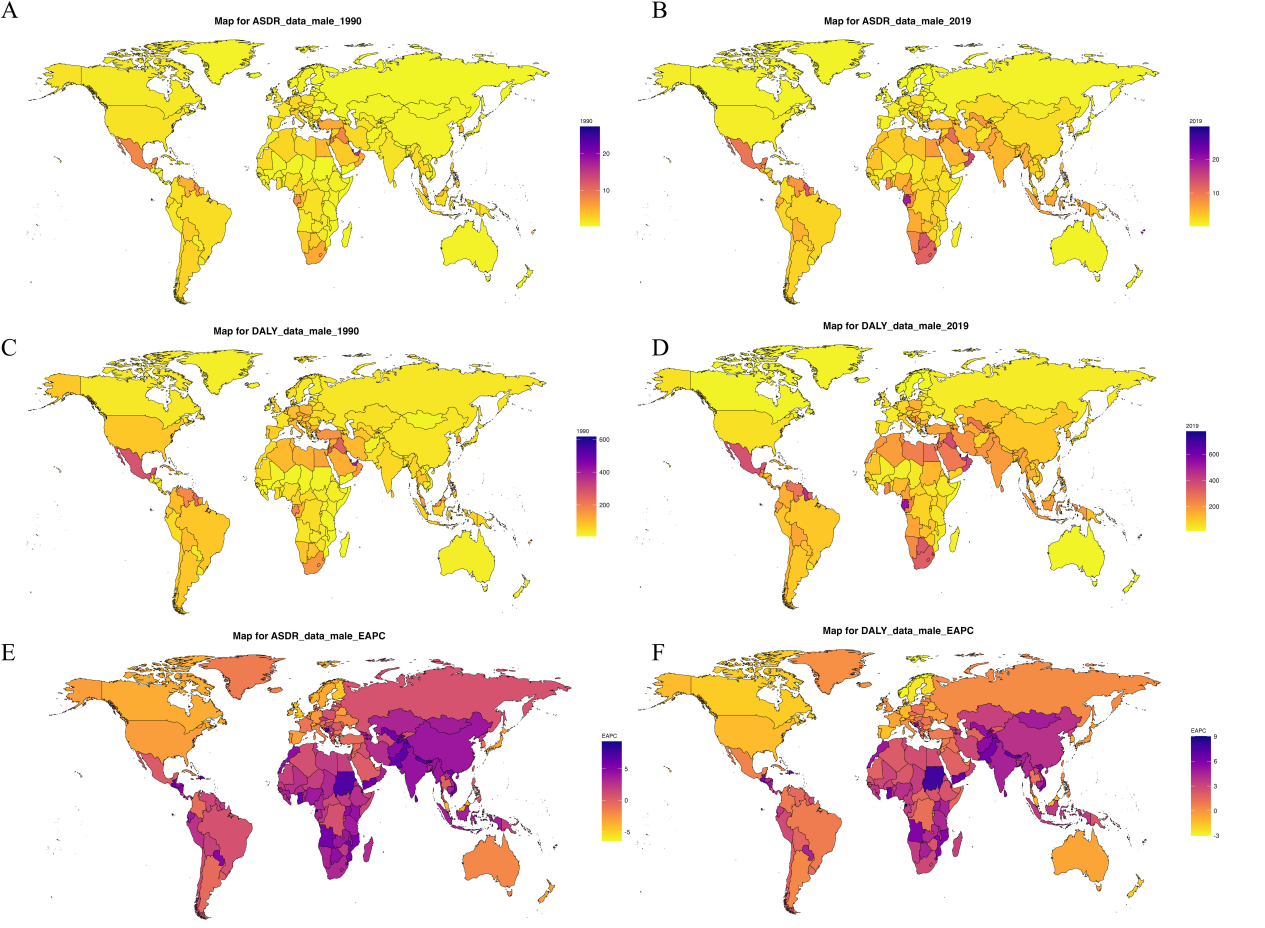


**Figure S3.** Global Disparities in Type 2 Diabetes Mellitus Attributed to Ambient particulate matter pollution from 1990 to 2019 in Male. (C) Age-Standardized DALY Rate in 1990, (D) Age-Standardized DALY Rate in 2019, (A) ASDR in 1990, (D) Age-Standardized Death Rate in 2019, (E) EAPCs in ASDR, (F) EAPCs in Age-Standardized DALY Rate. DALY = Disability-Adjusted Life-Year; ASDR = Age-Standardized Death Rate; EAPCs = Estimated Annual Percentage Changes.


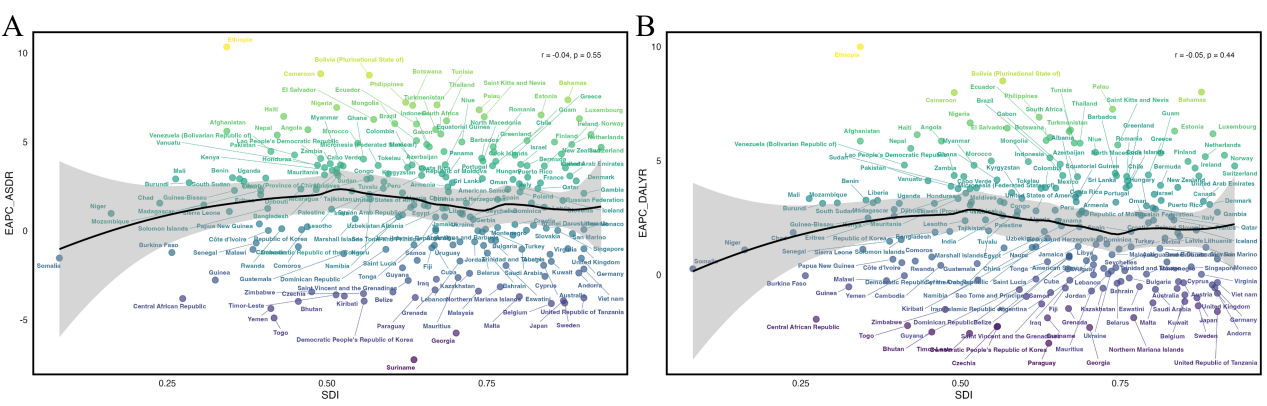


**Figure S4.** EAPCs in Type 2 Diabetes Mellitus Attributed to Ambient particulate matter pollution from 1990 to 2019 for 204 countries and territories by SDI; (A) EAPCs in ASDR, (B) EAPCs in Age-Standardized DALY Rate. Expected values based on the SDI and disease rates in all locations are shown as the black line. Each point shows the observed age-standardized DALY rate for each country in 1990 and 2019. ASDR = age-standardized Death rates. SDI= Socio-demographic Index


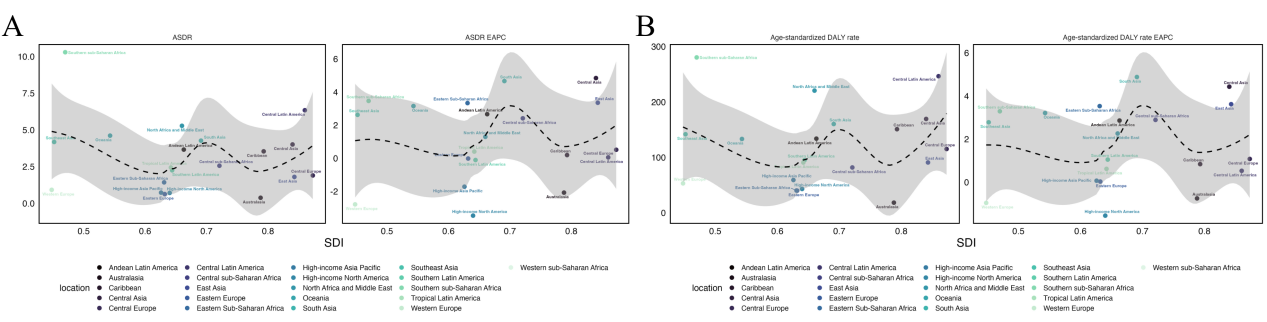


**Figure S5.** EAPCs in Type 2 Diabetes Mellitus Attributed to Ambient particulate matter pollution from 1990 to 2019 for 204 countries and territories by SDI; (A) ASDR in 2019 and its EAPCs in ASDR, (B) Age-Standardized DALY Rate in 2019 and its EAPCs in Age-Standardized DALY Rate. Expected values based on the SDI and disease rates in all locations are shown as the black line. Each point shows the observed age-standardized DALY rate for each country in 1990 and 2019. ASDR = age-standardized Death rates. SDI= Socio-demographic Index
