# Supplementary material for: Type 2 diabetes attributable to ambient particulate matter pollution: a global burden study from 1990 to 2019
Source: Front Public Health. 2024 May 20;12:1371253. doi: 10.3389/fpubh.2024.1371253 (PMC11144887; doi:10.3389/fpubh.2024.1371253)
Supplement: Supplementary file 2 [file Table_1.DOCX]

**Supplementary Table 1. The number and its trend of death and DALY for type 2 diabetes burden attributed to ambient particulate matter pollution in 1990 and 2019 in different regions.**

| **locations** | **Sex** | **deaths number in 1990** | **deaths number in 2019** | **daly number in 1990** | **daly number in 2019** | **Percentage change in deaths number** | **Percentage change in daly number** |
| --- | --- | --- | --- | --- | --- | --- | --- |
| global | both | 55824.18 | 196791.83 | 2330453.77 | 9034002.50 | 3.53 | 3.88 |
| High-middle SDI | both | 16815.55 | 41087.93 | 722235.52 | 2021314.12 | 2.44 | 2.80 |
| High SDI | both | 16586.77 | 18447.91 | 656770.75 | 992576.33 | 1.11 | 1.51 |
| Low-middle SDI | both | 4643.32 | 41241.19 | 194270.97 | 1753192.91 | 8.88 | 9.02 |
| Low SDI | both | 1524.07 | 9457.35 | 59564.68 | 412200.47 | 6.21 | 6.92 |
| Middle SDI | both | 16205.49 | 86419.95 | 695815.39 | 3848943.48 | 5.33 | 5.53 |
| Andean Latin America | both | 343.29 | 1990.39 | 12616.30 | 75519.27 | 5.80 | 5.99 |
| Australasia | both | 136.04 | 209.52 | 4578.31 | 8673.26 | 1.54 | 1.89 |
| Caribbean | both | 789.47 | 1836.70 | 29941.46 | 78178.36 | 2.33 | 2.61 |
| Central Asia | both | 430.09 | 2841.18 | 23074.23 | 135905.69 | 6.61 | 5.89 |
| Central Europe | both | 2545.23 | 4267.88 | 128175.16 | 233554.71 | 1.68 | 1.82 |
| Central Latin America | both | 4306.88 | 14581.77 | 167880.15 | 592994.24 | 3.39 | 3.53 |
| Central sub-Saharan Africa | both | 223.10 | 1124.16 | 7974.32 | 46530.71 | 5.04 | 5.84 |
| East Asia | both | 5487.70 | 34119.95 | 315396.79 | 1901689.56 | 6.22 | 6.03 |
| Eastern Europe | both | 1585.08 | 2225.60 | 109885.34 | 133411.70 | 1.40 | 1.21 |
| Eastern Sub-Saharan Africa | both | 388.88 | 1930.77 | 12573.80 | 67715.19 | 4.96 | 5.39 |
| High-income Asia Pacific | both | 2051.43 | 3670.28 | 104845.03 | 221102.61 | 1.79 | 2.11 |
| High-income North America | both | 5489.37 | 4621.01 | 230631.92 | 253125.16 | 0.84 | 1.10 |
| North Africa and Middle East | both | 5543.22 | 19939.17 | 213899.59 | 996411.77 | 3.60 | 4.66 |
| Oceania | both | 46.59 | 285.88 | 1667.04 | 10305.28 | 6.14 | 6.18 |
| South Asia | both | 5358.24 | 51453.99 | 245217.49 | 2303297.39 | 9.60 | 9.39 |
| Southeast Asia | both | 4456.36 | 23629.39 | 165221.15 | 899571.60 | 5.30 | 5.44 |
| Southern Latin America | both | 910.40 | 1918.20 | 29825.32 | 78247.37 | 2.11 | 2.62 |
| Southern sub-Saharan Africa | both | 1100.74 | 5091.03 | 34912.05 | 158115.63 | 4.63 | 4.53 |
| Tropical Latin America | both | 1759.70 | 5676.10 | 71897.30 | 221762.65 | 3.23 | 3.08 |
| Western Europe | both | 12016.60 | 10048.32 | 393488.49 | 441345.79 | 0.84 | 1.12 |
| Western sub-Saharan Africa | both | 855.76 | 5330.55 | 26752.53 | 176544.59 | 6.23 | 6.60 |
| global | female | 30517.95 | 97443.75 | 1158190.18 | 4213482.07 | 3.19 | 3.64 |
| High-middle SDI | female | 9850.96 | 21828.60 | 384311.12 | 988511.13 | 2.22 | 2.57 |
| High SDI | female | 9804.14 | 9098.18 | 341837.07 | 443742.42 | 0.93 | 1.30 |
| Low-middle SDI | female | 1956.96 | 18828.61 | 79175.14 | 770829.10 | 9.62 | 9.74 |
| Low SDI | female | 515.40 | 4004.11 | 20541.48 | 169618.83 | 7.77 | 8.26 |
| Middle SDI | female | 8362.59 | 43614.47 | 331402.03 | 1838040.24 | 5.22 | 5.55 |
| Andean Latin America | female | 178.94 | 1053.22 | 6182.74 | 37994.88 | 5.89 | 6.15 |
| Australasia | female | 69.71 | 96.77 | 2225.47 | 3895.45 | 1.39 | 1.75 |
| Caribbean | female | 465.58 | 984.96 | 15755.68 | 38217.68 | 2.12 | 2.43 |
| Central Asia | female | 233.79 | 1511.64 | 12353.85 | 71601.10 | 6.47 | 5.80 |
| Central Europe | female | 1476.36 | 2321.06 | 67251.62 | 114644.17 | 1.57 | 1.70 |
| Central Latin America | female | 2320.72 | 7172.10 | 84958.64 | 281691.89 | 3.09 | 3.32 |
| Central sub-Saharan Africa | female | 60.05 | 382.42 | 2322.56 | 15921.89 | 6.37 | 6.86 |
| East Asia | female | 2867.95 | 16741.95 | 144117.08 | 867507.85 | 5.84 | 6.02 |
| Eastern Europe | female | 1110.55 | 1527.97 | 69157.36 | 80920.24 | 1.38 | 1.17 |
| Eastern Sub-Saharan Africa | female | 131.20 | 666.85 | 4339.78 | 23583.12 | 5.08 | 5.43 |
| High-income Asia Pacific | female | 1031.27 | 1851.16 | 46602.81 | 97984.62 | 1.80 | 2.10 |
| High-income North America | female | 3167.58 | 2184.18 | 120403.55 | 114456.99 | 0.69 | 0.95 |
| North Africa and Middle East | female | 3038.70 | 10374.96 | 109428.89 | 487016.60 | 3.41 | 4.45 |
| Oceania | female | 15.68 | 110.59 | 562.84 | 3910.04 | 7.05 | 6.95 |
| South Asia | female | 2116.39 | 24137.72 | 93871.54 | 1022501.03 | 11.41 | 10.89 |
| Southeast Asia | female | 2185.62 | 11612.62 | 77073.68 | 424108.68 | 5.31 | 5.50 |
| Southern Latin America | female | 449.21 | 947.96 | 14582.54 | 38307.24 | 2.11 | 2.63 |
| Southern sub-Saharan Africa | female | 603.86 | 2894.12 | 18374.21 | 86293.90 | 4.79 | 4.70 |
| Tropical Latin America | female | 981.77 | 3050.82 | 36177.21 | 110352.23 | 3.11 | 3.05 |
| Western Europe | female | 7671.32 | 5499.97 | 222102.27 | 214429.50 | 0.72 | 0.97 |
| Western sub-Saharan Africa | female | 341.71 | 2320.73 | 10345.86 | 78142.95 | 6.79 | 7.55 |
| global | male | 25306.24 | 99348.08 | 1172263.59 | 4820520.43 | 3.93 | 4.11 |
| High-middle SDI | male | 6964.59 | 19259.33 | 337924.40 | 1032802.98 | 2.77 | 3.06 |
| High SDI | male | 6782.62 | 9349.73 | 314933.68 | 548833.91 | 1.38 | 1.74 |
| Low-middle SDI | male | 2686.36 | 22412.59 | 115095.83 | 982363.81 | 8.34 | 8.54 |
| Low SDI | male | 1008.67 | 5453.24 | 39023.20 | 242581.64 | 5.41 | 6.22 |
| Middle SDI | male | 7842.90 | 42805.49 | 364413.36 | 2010903.24 | 5.46 | 5.52 |
| Andean Latin America | male | 164.35 | 937.17 | 6433.56 | 37524.39 | 5.70 | 5.83 |
| Australasia | male | 66.34 | 112.75 | 2352.84 | 4777.80 | 1.70 | 2.03 |
| Caribbean | male | 323.90 | 851.74 | 14185.77 | 39960.68 | 2.63 | 2.82 |
| Central Asia | male | 196.30 | 1329.54 | 10720.38 | 64304.59 | 6.77 | 6.00 |
| Central Europe | male | 1068.87 | 1946.82 | 60923.54 | 118910.54 | 1.82 | 1.95 |
| Central Latin America | male | 1986.17 | 7409.67 | 82921.51 | 311302.34 | 3.73 | 3.75 |
| Central sub-Saharan Africa | male | 163.05 | 741.74 | 5651.76 | 30608.82 | 4.55 | 5.42 |
| East Asia | male | 2619.75 | 17378.00 | 171279.71 | 1034181.70 | 6.63 | 6.04 |
| Eastern Europe | male | 474.53 | 697.62 | 40727.98 | 52491.46 | 1.47 | 1.29 |
| Eastern Sub-Saharan Africa | male | 257.68 | 1263.93 | 8234.03 | 44132.07 | 4.91 | 5.36 |
| High-income Asia Pacific | male | 1020.16 | 1819.12 | 58242.22 | 123117.99 | 1.78 | 2.11 |
| High-income North America | male | 2321.79 | 2436.83 | 110228.37 | 138668.16 | 1.05 | 1.26 |
| North Africa and Middle East | male | 2504.52 | 9564.21 | 104470.69 | 509395.16 | 3.82 | 4.88 |
| Oceania | male | 30.91 | 175.29 | 1104.19 | 6395.24 | 5.67 | 5.79 |
| South Asia | male | 3241.85 | 27316.28 | 151345.95 | 1280796.37 | 8.43 | 8.46 |
| Southeast Asia | male | 2270.74 | 12016.76 | 88147.48 | 475462.91 | 5.29 | 5.39 |
| Southern Latin America | male | 461.19 | 970.24 | 15242.78 | 39940.12 | 2.10 | 2.62 |
| Southern sub-Saharan Africa | male | 496.88 | 2196.91 | 16537.84 | 71821.73 | 4.42 | 4.34 |
| Tropical Latin America | male | 777.93 | 2625.28 | 35720.09 | 111410.42 | 3.37 | 3.12 |
| Western Europe | male | 4345.27 | 4548.36 | 171386.22 | 226916.28 | 1.05 | 1.32 |
| Western sub-Saharan Africa | male | 514.05 | 3009.82 | 16406.67 | 98401.63 | 5.86 | 6.00 |

**Supplementary Table 2. The number and its trend of deaths and DALY type 2 diabetes burden attributed to ambient particulate matter pollution in 1990 and 2019, by countries and regions.**

| **Country** | **Region** | **Sex** | **deaths number in 1990** | **deaths number in 2019** | **daly number in 1990** | **daly number in 2019** | **Percentage change in deaths number** | **Percentage change in daly number** |
| --- | --- | --- | --- | --- | --- | --- | --- | --- |
| Afghanistan | North Africa and Middle East | both | 39.83 | 250.11 | 1585.29 | 12892.53 | 6.28 | 8.13 |
| Albania | Central Europe | both | 5.64 | 18.80 | 416.41 | 1820.74 | 3.34 | 4.37 |
| Algeria | North Africa and Middle East | both | 248.86 | 1064.44 | 12720.28 | 69895.60 | 4.28 | 5.49 |
| American Samoa | Oceania | both | 0.57 | 1.87 | 24.09 | 79.98 | 3.26 | 3.32 |
| Andorra | Western Europe | both | 0.41 | 0.66 | 19.77 | 41.21 | 1.63 | 2.08 |
| Angola | Central sub-Saharan Africa | both | 25.92 | 333.31 | 989.52 | 14072.76 | 12.86 | 14.22 |
| Antigua and Barbuda | Caribbean | both | 4.26 | 8.06 | 120.36 | 290.69 | 1.89 | 2.42 |
| Argentina | Southern Latin America | both | 691.98 | 1196.19 | 21856.52 | 46207.39 | 1.73 | 2.11 |
| Armenia | Central Asia | both | 63.93 | 241.35 | 2649.20 | 9015.51 | 3.78 | 3.40 |
| Australia | Australasia | both | 117.01 | 186.62 | 3923.48 | 7671.87 | 1.59 | 1.96 |
| Austria | Western Europe | both | 257.56 | 256.45 | 7507.98 | 9276.14 | 1.00 | 1.24 |
| Azerbaijan | Central Asia | both | 51.54 | 342.75 | 2587.38 | 17320.57 | 6.65 | 6.69 |
| Bahrain | North Africa and Middle East | both | 21.04 | 167.22 | 799.32 | 7477.02 | 7.95 | 9.35 |
| Bangladesh | South Asia | both | 337.29 | 3318.28 | 11701.59 | 117081.44 | 9.84 | 10.01 |
| Barbados | Caribbean | both | 35.25 | 51.75 | 888.16 | 1523.34 | 1.47 | 1.72 |
| Belarus | Eastern Europe | both | 93.60 | 52.18 | 6599.15 | 6118.03 | 0.56 | 0.93 |
| Belgium | Western Europe | both | 294.25 | 196.29 | 10146.79 | 11044.59 | 0.67 | 1.09 |
| Belize | Caribbean | both | 2.52 | 19.40 | 76.95 | 714.78 | 7.69 | 9.29 |
| Benin | Western sub-Saharan Africa | both | 11.01 | 72.14 | 350.93 | 2704.55 | 6.55 | 7.71 |
| Bermuda | Caribbean | both | 1.34 | 1.19 | 43.24 | 49.25 | 0.89 | 1.14 |
| Bhutan | South Asia | both | 0.76 | 15.21 | 33.78 | 568.13 | 20.04 | 16.82 |
| Bolivia (Plurinational State of) | Andean Latin America | both | 103.10 | 474.62 | 3357.69 | 15434.22 | 4.60 | 4.60 |
| Bosnia and Herzegovina | Central Europe | both | 44.73 | 360.49 | 2250.24 | 12530.41 | 8.06 | 5.57 |
| Botswana | Southern sub-Saharan Africa | both | 13.01 | 131.30 | 392.79 | 4148.53 | 10.09 | 10.56 |
| Brazil | Tropical Latin America | both | 1741.58 | 5500.60 | 71245.27 | 216026.90 | 3.16 | 3.03 |
| Brunei Darussalam | High-income Asia Pacific | both | 4.81 | 9.54 | 172.33 | 441.34 | 1.98 | 2.56 |
| Bulgaria | Central Europe | both | 281.76 | 314.21 | 11974.03 | 14781.53 | 1.12 | 1.23 |
| Burkina Faso | Western sub-Saharan Africa | both | 22.76 | 97.19 | 733.78 | 3607.97 | 4.27 | 4.92 |
| Burundi | Eastern Sub-Saharan Africa | both | 16.83 | 34.02 | 524.37 | 1256.87 | 2.02 | 2.40 |
| Cambodia | Southeast Asia | both | 24.31 | 157.89 | 844.31 | 6722.06 | 6.49 | 7.96 |
| Cameroon | Western sub-Saharan Africa | both | 74.88 | 566.09 | 2319.70 | 19005.95 | 7.56 | 8.19 |
| Canada | High-income North America | both | 330.16 | 347.44 | 9019.06 | 13383.47 | 1.05 | 1.48 |
| Cabo Verde | Western sub-Saharan Africa | both | 0.91 | 23.06 | 42.91 | 798.62 | 25.41 | 18.61 |
| Central African Republic | Central sub-Saharan Africa | both | 11.60 | 32.29 | 425.99 | 1373.94 | 2.78 | 3.23 |
| Chad | Western sub-Saharan Africa | both | 10.98 | 55.36 | 361.14 | 2070.11 | 5.04 | 5.73 |
| Chile | Southern Latin America | both | 170.46 | 652.85 | 6829.11 | 29855.19 | 3.83 | 4.37 |
| China | East Asia | both | 4817.47 | 31801.42 | 290843.28 | 1815953.24 | 6.60 | 6.24 |
| Colombia | Central Latin America | both | 306.01 | 1052.83 | 16622.42 | 70662.69 | 3.44 | 4.25 |
| Comoros | Eastern Sub-Saharan Africa | both | 0.99 | 5.36 | 28.92 | 165.56 | 5.41 | 5.72 |
| Congo | Central sub-Saharan Africa | both | 23.33 | 137.59 | 807.86 | 5436.99 | 5.90 | 6.73 |
| Costa Rica | Central Latin America | both | 19.41 | 81.84 | 1107.15 | 6614.07 | 4.22 | 5.97 |
| Côte d'Ivoire | Western sub-Saharan Africa | both | 38.74 | 249.86 | 1379.58 | 9539.41 | 6.45 | 6.91 |
| Croatia | Central Europe | both | 115.81 | 168.04 | 5842.47 | 8907.52 | 1.45 | 1.52 |
| Cuba | Caribbean | both | 292.30 | 287.22 | 13845.16 | 23198.79 | 0.98 | 1.68 |
| Cyprus | Western Europe | both | 62.85 | 68.86 | 1489.14 | 2192.52 | 1.10 | 1.47 |
| Czechia | Central Europe | both | 307.70 | 482.60 | 16677.39 | 29880.35 | 1.57 | 1.79 |
| Democratic Republic of the Congo | Central sub-Saharan Africa | both | 139.88 | 462.22 | 5037.77 | 20034.20 | 3.30 | 3.98 |
| Denmark | Western Europe | both | 114.68 | 117.05 | 3423.04 | 3826.21 | 1.02 | 1.12 |
| Djibouti | Eastern Sub-Saharan Africa | both | 1.59 | 31.04 | 59.09 | 1155.82 | 19.58 | 19.56 |
| Dominica | Caribbean | both | 3.79 | 8.01 | 102.62 | 276.23 | 2.12 | 2.69 |
| Dominican Republic | Caribbean | both | 29.11 | 346.90 | 1101.41 | 13422.26 | 11.92 | 12.19 |
| Ecuador | Andean Latin America | both | 99.43 | 754.18 | 3754.06 | 27455.09 | 7.58 | 7.31 |
| Egypt | North Africa and Middle East | both | 1243.28 | 4619.41 | 45081.41 | 199554.99 | 3.72 | 4.43 |
| El Salvador | Central Latin America | both | 24.75 | 311.78 | 1158.44 | 12523.16 | 12.60 | 10.81 |
| Equatorial Guinea | Central sub-Saharan Africa | both | 1.68 | 40.26 | 60.33 | 1485.68 | 23.93 | 24.63 |
| Eritrea | Eastern Sub-Saharan Africa | both | 7.98 | 63.25 | 301.33 | 2407.26 | 7.93 | 7.99 |
| Estonia | Eastern Europe | both | 4.75 | 4.71 | 411.35 | 368.73 | 0.99 | 0.90 |
| Ethiopia | Eastern Sub-Saharan Africa | both | 111.41 | 447.55 | 3692.33 | 14213.73 | 4.02 | 3.85 |
| Micronesia (Federated States of) | Oceania | both | 0.92 | 5.76 | 31.02 | 205.16 | 6.22 | 6.61 |
| Fiji | Oceania | both | 13.02 | 120.49 | 437.10 | 3834.29 | 9.25 | 8.77 |
| Finland | Western Europe | both | 32.48 | 12.56 | 1828.60 | 1408.36 | 0.39 | 0.77 |
| France | Western Europe | both | 1056.60 | 1278.75 | 28462.86 | 37205.61 | 1.21 | 1.31 |
| Gabon | Central sub-Saharan Africa | both | 20.69 | 118.48 | 652.83 | 4127.13 | 5.73 | 6.32 |
| Georgia | Central Asia | both | 56.42 | 150.92 | 2833.91 | 6915.46 | 2.67 | 2.44 |
| Germany | Western Europe | both | 3426.77 | 2371.67 | 116037.95 | 112395.64 | 0.69 | 0.97 |
| Ghana | Western sub-Saharan Africa | both | 79.95 | 777.22 | 2826.80 | 28063.03 | 9.72 | 9.93 |
| Greece | Western Europe | both | 181.25 | 176.93 | 8207.45 | 12233.42 | 0.98 | 1.49 |
| Greenland | High-income North America | both | 0.20 | 0.24 | 7.08 | 13.84 | 1.16 | 1.95 |
| Grenada | Caribbean | both | 3.96 | 11.97 | 108.37 | 429.17 | 3.02 | 3.96 |
| Guam | Oceania | both | 1.66 | 2.98 | 67.96 | 149.99 | 1.79 | 2.21 |
| Guatemala | Central Latin America | both | 21.85 | 588.17 | 1193.52 | 23338.24 | 26.92 | 19.55 |
| Guinea | Western sub-Saharan Africa | both | 18.04 | 76.66 | 548.35 | 2539.88 | 4.25 | 4.63 |
| Guinea-Bissau | Western sub-Saharan Africa | both | 3.67 | 14.07 | 119.63 | 509.87 | 3.83 | 4.26 |
| Guyana | Caribbean | both | 27.21 | 78.44 | 976.80 | 2974.04 | 2.88 | 3.04 |
| Haiti | Caribbean | both | 34.19 | 118.21 | 1203.97 | 5093.08 | 3.46 | 4.23 |
| Honduras | Central Latin America | both | 7.61 | 73.18 | 560.65 | 5196.61 | 9.62 | 9.27 |
| Hungary | Central Europe | both | 242.64 | 328.36 | 12016.26 | 18407.17 | 1.35 | 1.53 |
| Iceland | Western Europe | both | 0.72 | 0.72 | 33.74 | 57.15 | 0.99 | 1.69 |
| India | South Asia | both | 4492.18 | 42006.17 | 212081.42 | 1934780.66 | 9.35 | 9.12 |
| Indonesia | Southeast Asia | both | 1724.13 | 11146.08 | 64326.41 | 402392.95 | 6.46 | 6.26 |
| Iran (Islamic Republic of) | North Africa and Middle East | both | 483.91 | 3162.04 | 26776.89 | 158843.00 | 6.53 | 5.93 |
| Iraq | North Africa and Middle East | both | 596.88 | 2060.29 | 20974.19 | 90221.93 | 3.45 | 4.30 |
| Ireland | Western Europe | both | 45.24 | 29.49 | 1253.42 | 1717.62 | 0.65 | 1.37 |
| Israel | Western Europe | both | 139.50 | 436.35 | 4315.96 | 12837.03 | 3.13 | 2.97 |
| Italy | Western Europe | both | 3001.27 | 2860.98 | 88226.18 | 104833.56 | 0.95 | 1.19 |
| Jamaica | Caribbean | both | 56.56 | 286.38 | 1550.30 | 8210.51 | 5.06 | 5.30 |
| Japan | High-income Asia Pacific | both | 1131.86 | 1048.16 | 63611.37 | 107112.84 | 0.93 | 1.68 |
| Jordan | North Africa and Middle East | both | 133.12 | 424.96 | 4258.92 | 18249.53 | 3.19 | 4.29 |
| Kazakhstan | Central Asia | both | 79.35 | 357.95 | 5932.30 | 22427.50 | 4.51 | 3.78 |
| Kenya | Eastern Sub-Saharan Africa | both | 46.09 | 334.34 | 1510.04 | 12359.76 | 7.25 | 8.19 |
| Kiribati | Oceania | both | 0.76 | 3.04 | 26.58 | 111.75 | 3.99 | 4.20 |
| Kuwait | North Africa and Middle East | both | 30.36 | 84.92 | 1734.10 | 8462.09 | 2.80 | 4.88 |
| Kyrgyzstan | Central Asia | both | 12.25 | 36.36 | 715.38 | 2771.67 | 2.97 | 3.87 |
| Lao People's Democratic Republic | Southeast Asia | both | 13.28 | 61.47 | 498.88 | 2697.75 | 4.63 | 5.41 |
| Latvia | Eastern Europe | both | 23.58 | 34.27 | 1574.83 | 1827.06 | 1.45 | 1.16 |
| Lebanon | North Africa and Middle East | both | 64.58 | 171.00 | 2949.62 | 10745.67 | 2.65 | 3.64 |
| Lesotho | Southern sub-Saharan Africa | both | 16.28 | 96.44 | 473.24 | 2842.08 | 5.92 | 6.01 |
| Liberia | Western sub-Saharan Africa | both | 7.80 | 30.41 | 263.04 | 1247.62 | 3.90 | 4.74 |
| Libya | North Africa and Middle East | both | 36.60 | 184.90 | 2052.54 | 13755.50 | 5.05 | 6.70 |
| Lithuania | Eastern Europe | both | 21.10 | 20.16 | 1615.09 | 1569.28 | 0.96 | 0.97 |
| Luxembourg | Western Europe | both | 7.75 | 5.81 | 226.98 | 481.67 | 0.75 | 2.12 |
| North Macedonia | Central Europe | both | 49.17 | 181.33 | 2145.07 | 7348.49 | 3.69 | 3.43 |
| Madagascar | Eastern Sub-Saharan Africa | both | 16.00 | 65.51 | 529.77 | 2636.20 | 4.10 | 4.98 |
| Malawi | Eastern Sub-Saharan Africa | both | 15.87 | 60.85 | 528.91 | 2257.56 | 3.83 | 4.27 |
| Malaysia | Southeast Asia | both | 382.15 | 472.85 | 15647.50 | 32961.64 | 1.24 | 2.11 |
| Maldives | Southeast Asia | both | 0.75 | 3.74 | 28.50 | 196.49 | 4.98 | 6.90 |
| Mali | Western sub-Saharan Africa | both | 19.60 | 91.61 | 648.22 | 3240.33 | 4.67 | 5.00 |
| Malta | Western Europe | both | 13.26 | 14.49 | 416.15 | 656.17 | 1.09 | 1.58 |
| Marshall Islands | Oceania | both | 0.23 | 1.43 | 9.37 | 69.56 | 6.27 | 7.43 |
| Mauritania | Western sub-Saharan Africa | both | 16.67 | 79.25 | 478.73 | 2349.29 | 4.75 | 4.91 |
| Mauritius | Southeast Asia | both | 31.66 | 242.38 | 1205.30 | 7653.31 | 7.66 | 6.35 |
| Mexico | Central Latin America | both | 3453.96 | 10345.08 | 128129.83 | 388025.60 | 3.00 | 3.03 |
| Republic of Moldova | Eastern Europe | both | 26.86 | 31.52 | 1865.87 | 3135.78 | 1.17 | 1.68 |
| Mongolia | Central Asia | both | 2.57 | 17.07 | 144.84 | 1287.80 | 6.64 | 8.89 |
| Montenegro | Central Europe | both | 10.50 | 20.30 | 580.70 | 1240.99 | 1.93 | 2.14 |
| Morocco | North Africa and Middle East | both | 132.52 | 1283.32 | 6523.67 | 67230.31 | 9.68 | 10.31 |
| Mozambique | Eastern Sub-Saharan Africa | both | 14.69 | 84.23 | 482.89 | 3034.49 | 5.74 | 6.28 |
| Myanmar | Southeast Asia | both | 343.22 | 1655.38 | 11697.88 | 59351.74 | 4.82 | 5.07 |
| Namibia | Southern sub-Saharan Africa | both | 17.36 | 84.25 | 503.15 | 2488.95 | 4.85 | 4.95 |
| Nepal | South Asia | both | 27.23 | 375.40 | 1547.59 | 20064.80 | 13.79 | 12.97 |
| Netherlands | Western Europe | both | 554.26 | 358.64 | 16071.61 | 14323.55 | 0.65 | 0.89 |
| New Zealand | Australasia | both | 19.03 | 22.90 | 654.83 | 1001.38 | 1.20 | 1.53 |
| Nicaragua | Central Latin America | both | 10.35 | 141.49 | 454.54 | 5792.57 | 13.67 | 12.74 |
| Niger | Western sub-Saharan Africa | both | 11.49 | 59.71 | 353.72 | 2051.73 | 5.20 | 5.80 |
| Nigeria | Western sub-Saharan Africa | both | 487.96 | 2840.52 | 14585.44 | 88010.54 | 5.82 | 6.03 |
| Democratic People's Republic of Korea | East Asia | both | 94.97 | 378.09 | 4378.34 | 19640.64 | 3.98 | 4.49 |
| Northern Mariana Islands | Oceania | both | 0.50 | 1.71 | 23.92 | 74.77 | 3.39 | 3.13 |
| Norway | Western Europe | both | 48.47 | 25.54 | 2280.89 | 1598.40 | 0.53 | 0.70 |
| Oman | North Africa and Middle East | both | 27.91 | 147.48 | 1025.19 | 6709.35 | 5.28 | 6.54 |
| Pakistan | South Asia | both | 500.78 | 5738.92 | 19853.11 | 230802.36 | 11.46 | 11.63 |
| Palestine | North Africa and Middle East | both | 41.50 | 263.15 | 1204.30 | 9217.48 | 6.34 | 7.65 |
| Panama | Central Latin America | both | 18.64 | 127.64 | 879.82 | 5646.24 | 6.85 | 6.42 |
| Papua New Guinea | Oceania | both | 20.76 | 110.40 | 765.15 | 4382.56 | 5.32 | 5.73 |
| Paraguay | Tropical Latin America | both | 18.12 | 175.49 | 652.03 | 5735.76 | 9.69 | 8.80 |
| Peru | Andean Latin America | both | 140.76 | 761.59 | 5504.54 | 32629.96 | 5.41 | 5.93 |
| Philippines | Southeast Asia | both | 669.29 | 2502.30 | 24933.53 | 92839.56 | 3.74 | 3.72 |
| Poland | Central Europe | both | 838.79 | 1308.08 | 44014.44 | 79713.53 | 1.56 | 1.81 |
| Portugal | Western Europe | both | 255.87 | 277.66 | 8420.78 | 10122.66 | 1.09 | 1.20 |
| Puerto Rico | Caribbean | both | 85.90 | 148.25 | 2912.97 | 5109.18 | 1.73 | 1.75 |
| Qatar | North Africa and Middle East | both | 15.03 | 81.49 | 620.25 | 6935.37 | 5.42 | 11.18 |
| Romania | Central Europe | both | 207.45 | 304.35 | 12565.44 | 21886.89 | 1.47 | 1.74 |
| Russian Federation | Eastern Europe | both | 1014.95 | 1782.28 | 66460.21 | 89596.23 | 1.76 | 1.35 |
| Rwanda | Eastern Sub-Saharan Africa | both | 29.84 | 89.26 | 940.24 | 3056.11 | 2.99 | 3.25 |
| Saint Lucia | Caribbean | both | 5.70 | 20.60 | 183.35 | 797.89 | 3.62 | 4.35 |
| Saint Vincent and the Grenadines | Caribbean | both | 4.82 | 16.50 | 140.65 | 560.43 | 3.42 | 3.98 |
| Samoa | Oceania | both | 1.90 | 5.22 | 63.14 | 189.49 | 2.75 | 3.00 |
| Sao Tome and Principe | Central sub-Saharan Africa | both | 0.18 | 1.17 | 8.66 | 64.95 | 6.36 | 7.50 |
| Saudi Arabia | North Africa and Middle East | both | 152.76 | 663.02 | 7031.34 | 57706.61 | 4.34 | 8.21 |
| Senegal | Western sub-Saharan Africa | both | 29.96 | 171.78 | 1102.25 | 6570.52 | 5.73 | 5.96 |
| Serbia | Central Europe | both | 256.00 | 605.23 | 11424.16 | 25774.83 | 2.36 | 2.26 |
| Seychelles | Southeast Asia | both | 0.91 | 3.40 | 42.95 | 211.08 | 3.73 | 4.91 |
| Sierra Leone | Western sub-Saharan Africa | both | 10.57 | 44.55 | 285.76 | 1411.59 | 4.21 | 4.94 |
| Singapore | High-income Asia Pacific | both | 58.29 | 28.45 | 3011.79 | 6172.05 | 0.49 | 2.05 |
| Slovakia | Central Europe | both | 144.02 | 124.71 | 6246.28 | 8161.61 | 0.87 | 1.31 |
| Slovenia | Central Europe | both | 41.03 | 51.37 | 2022.27 | 3100.66 | 1.25 | 1.53 |
| Solomon Islands | Oceania | both | 0.89 | 8.13 | 33.97 | 327.56 | 9.13 | 9.64 |
| Somalia | Eastern Sub-Saharan Africa | both | 6.25 | 20.97 | 219.26 | 802.63 | 3.36 | 3.66 |
| South Africa | Southern sub-Saharan Africa | both | 1003.40 | 4559.74 | 31794.42 | 141008.96 | 4.54 | 4.44 |
| Republic of Korea | High-income Asia Pacific | both | 856.47 | 2584.13 | 38049.54 | 107376.38 | 3.02 | 2.82 |
| South Sudan | Eastern Sub-Saharan Africa | both | 17.68 | 45.49 | 547.66 | 1627.09 | 2.57 | 2.97 |
| Spain | Western Europe | both | 1071.52 | 851.53 | 37731.53 | 41767.98 | 0.79 | 1.11 |
| Sri Lanka | Southeast Asia | both | 125.60 | 1402.94 | 5258.73 | 52365.45 | 11.17 | 9.96 |
| Sudan | North Africa and Middle East | both | 28.62 | 363.44 | 1479.64 | 24377.13 | 12.70 | 16.47 |
| Suriname | Caribbean | both | 9.11 | 38.24 | 374.51 | 1795.02 | 4.20 | 4.79 |
| Eswatini | Southern sub-Saharan Africa | both | 8.04 | 62.13 | 233.77 | 1820.16 | 7.72 | 7.79 |
| Sweden | Western Europe | both | 110.57 | 58.90 | 3756.90 | 2272.51 | 0.53 | 0.60 |
| Switzerland | Western Europe | both | 229.66 | 120.23 | 6757.62 | 5751.56 | 0.52 | 0.85 |
| Syrian Arab Republic | North Africa and Middle East | both | 156.73 | 339.52 | 7356.95 | 22961.16 | 2.17 | 3.12 |
| Taiwan (Province of China) | East Asia | both | 575.25 | 1940.44 | 20175.17 | 66095.68 | 3.37 | 3.28 |
| Tajikistan | Central Asia | both | 20.14 | 212.69 | 950.79 | 10085.83 | 10.56 | 10.61 |
| United Republic of Tanzania | Eastern Sub-Saharan Africa | both | 47.29 | 283.64 | 1384.28 | 9307.16 | 6.00 | 6.72 |
| Thailand | Southeast Asia | both | 764.69 | 3085.01 | 29310.13 | 140302.32 | 4.03 | 4.79 |
| Bahamas | Caribbean | both | 8.92 | 18.35 | 320.03 | 834.03 | 2.06 | 2.61 |
| Gambia | Western sub-Saharan Africa | both | 2.01 | 19.77 | 68.50 | 657.61 | 9.85 | 9.60 |
| Timor-Leste | Southeast Asia | both | 0.62 | 6.73 | 26.39 | 302.34 | 10.78 | 11.46 |
| Togo | Western sub-Saharan Africa | both | 8.55 | 60.06 | 274.50 | 2098.55 | 7.02 | 7.65 |
| Tonga | Oceania | both | 1.26 | 4.79 | 40.08 | 147.45 | 3.79 | 3.68 |
| Trinidad and Tobago | Caribbean | both | 154.66 | 306.98 | 4873.59 | 9939.24 | 1.98 | 2.04 |
| Tunisia | North Africa and Middle East | both | 73.91 | 362.53 | 4651.36 | 25386.57 | 4.91 | 5.46 |
| Turkey | North Africa and Middle East | both | 1957.87 | 3738.33 | 62477.94 | 152404.39 | 1.91 | 2.44 |
| Turkmenistan | Central Asia | both | 31.90 | 122.53 | 1654.65 | 6789.81 | 3.84 | 4.10 |
| Uganda | Eastern Sub-Saharan Africa | both | 32.53 | 213.98 | 1049.30 | 7951.31 | 6.58 | 7.58 |
| Ukraine | Eastern Europe | both | 400.24 | 300.48 | 31358.85 | 30796.58 | 0.75 | 0.98 |
| United Arab Emirates | North Africa and Middle East | both | 40.31 | 285.01 | 1759.11 | 20311.54 | 7.07 | 11.55 |
| United Kingdom | Western Europe | both | 1101.22 | 519.19 | 46524.80 | 54865.14 | 0.47 | 1.18 |
| United States of America | High-income North America | both | 5158.88 | 4273.25 | 221600.51 | 239723.83 | 0.83 | 1.08 |
| Uruguay | Southern Latin America | both | 47.92 | 69.06 | 1138.49 | 2180.85 | 1.44 | 1.92 |
| Uzbekistan | Central Asia | both | 111.99 | 1359.55 | 5605.77 | 59291.54 | 12.14 | 10.58 |
| Vanuatu | Oceania | both | 0.43 | 3.63 | 17.52 | 145.79 | 8.43 | 8.32 |
| Venezuela (Bolivarian Republic of) | Central Latin America | both | 444.31 | 1859.75 | 17773.77 | 75195.06 | 4.19 | 4.23 |
| Viet nam | Southeast Asia | both | 369.81 | 2858.24 | 11181.02 | 100396.40 | 7.73 | 8.98 |
| United States Virgin Islands | Caribbean | both | 2.23 | 5.78 | 82.33 | 218.56 | 2.59 | 2.65 |
| Yemen | North Africa and Middle East | both | 13.88 | 202.33 | 693.44 | 12061.65 | 14.58 | 17.39 |
| Zambia | Eastern Sub-Saharan Africa | both | 23.56 | 149.72 | 766.19 | 5429.50 | 6.35 | 7.09 |
| Zimbabwe | Southern sub-Saharan Africa | both | 42.64 | 157.17 | 1514.69 | 5806.95 | 3.69 | 3.83 |
| Monaco | Western Europe | both | 0.21 | 0.38 | 11.36 | 31.01 | 1.79 | 2.73 |
| San Marino | Western Europe | both | 0.25 | 0.40 | 10.28 | 21.33 | 1.56 | 2.08 |
| Saint Kitts and Nevis | Caribbean | both | 1.35 | 2.28 | 39.25 | 93.60 | 1.69 | 2.38 |
| Cook Islands | Oceania | both | 0.49 | 1.15 | 14.50 | 34.50 | 2.34 | 2.38 |
| Nauru | Oceania | both | 0.10 | 0.19 | 3.70 | 7.96 | 1.95 | 2.15 |
| Niue | Oceania | both | 0.06 | 0.12 | 1.65 | 3.98 | 2.15 | 2.41 |
| Palau | Oceania | both | 0.33 | 1.06 | 11.26 | 39.99 | 3.23 | 3.55 |
| Tokelau | Oceania | both | 0.05 | 0.05 | 1.44 | 1.76 | 1.10 | 1.22 |
| Tuvalu | Oceania | both | 0.07 | 0.35 | 2.34 | 12.01 | 5.03 | 5.14 |
| Afghanistan | North Africa and Middle East | male | 16.82 | 76.36 | 721.57 | 5057.00 | 4.54 | 7.01 |
| Albania | Central Europe | male | 3.06 | 9.74 | 244.63 | 991.79 | 3.19 | 4.05 |
| Algeria | North Africa and Middle East | male | 111.92 | 445.98 | 6028.26 | 32414.62 | 3.98 | 5.38 |
| American Samoa | Oceania | male | 0.36 | 0.98 | 15.21 | 42.55 | 2.73 | 2.80 |
| Andorra | Western Europe | male | 0.27 | 0.40 | 12.43 | 24.00 | 1.47 | 1.93 |
| Angola | Central sub-Saharan Africa | male | 19.21 | 221.70 | 717.70 | 9239.61 | 11.54 | 12.87 |
| Antigua and Barbuda | Caribbean | male | 1.88 | 3.48 | 56.91 | 136.46 | 1.85 | 2.40 |
| Argentina | Southern Latin America | male | 352.72 | 624.42 | 11225.88 | 24101.06 | 1.77 | 2.15 |
| Armenia | Central Asia | male | 24.60 | 96.83 | 1100.47 | 3844.26 | 3.94 | 3.49 |
| Australia | Australasia | male | 56.85 | 100.34 | 2025.94 | 4242.27 | 1.76 | 2.09 |
| Austria | Western Europe | male | 89.94 | 124.10 | 3076.53 | 4818.78 | 1.38 | 1.57 |
| Azerbaijan | Central Asia | male | 24.47 | 156.31 | 1249.23 | 7997.87 | 6.39 | 6.40 |
| Bahrain | North Africa and Middle East | male | 11.96 | 97.39 | 474.38 | 4789.05 | 8.14 | 10.10 |
| Bangladesh | South Asia | male | 225.87 | 1774.79 | 7522.58 | 63592.98 | 7.86 | 8.45 |
| Barbados | Caribbean | male | 12.04 | 20.99 | 330.34 | 668.60 | 1.74 | 2.02 |
| Belarus | Eastern Europe | male | 32.07 | 21.18 | 2547.81 | 2583.16 | 0.66 | 1.01 |
| Belgium | Western Europe | male | 91.72 | 85.21 | 4182.67 | 5525.26 | 0.93 | 1.32 |
| Belize | Caribbean | male | 1.05 | 9.58 | 34.87 | 353.59 | 9.10 | 10.14 |
| Benin | Western sub-Saharan Africa | male | 6.81 | 38.83 | 214.21 | 1504.98 | 5.70 | 7.03 |
| Bermuda | Caribbean | male | 0.63 | 0.63 | 22.27 | 26.69 | 1.01 | 1.20 |
| Bhutan | South Asia | male | 0.40 | 8.44 | 18.93 | 325.09 | 21.03 | 17.18 |
| Bolivia (Plurinational State of) | Andean Latin America | male | 41.81 | 200.84 | 1493.62 | 7256.56 | 4.80 | 4.86 |
| Bosnia and Herzegovina | Central Europe | male | 17.54 | 156.20 | 1088.13 | 6193.59 | 8.91 | 5.69 |
| Botswana | Southern sub-Saharan Africa | male | 6.84 | 63.39 | 213.86 | 2074.54 | 9.27 | 9.70 |
| Brazil | Tropical Latin America | male | 769.53 | 2537.39 | 35391.41 | 108280.65 | 3.30 | 3.06 |
| Brunei Darussalam | High-income Asia Pacific | male | 2.69 | 5.26 | 101.09 | 258.26 | 1.95 | 2.55 |
| Bulgaria | Central Europe | male | 131.74 | 143.86 | 5968.84 | 7279.37 | 1.09 | 1.22 |
| Burkina Faso | Western sub-Saharan Africa | male | 15.28 | 63.39 | 468.74 | 2236.99 | 4.15 | 4.77 |
| Burundi | Eastern Sub-Saharan Africa | male | 10.64 | 22.78 | 328.52 | 842.74 | 2.14 | 2.57 |
| Cambodia | Southeast Asia | male | 12.54 | 79.25 | 444.56 | 3596.31 | 6.32 | 8.09 |
| Cameroon | Western sub-Saharan Africa | male | 45.39 | 311.42 | 1407.28 | 10764.00 | 6.86 | 7.65 |
| Canada | High-income North America | male | 150.82 | 183.13 | 4516.57 | 7425.70 | 1.21 | 1.64 |
| Cabo Verde | Western sub-Saharan Africa | male | 0.46 | 9.33 | 21.96 | 369.94 | 20.50 | 16.85 |
| Central African Republic | Central sub-Saharan Africa | male | 8.61 | 22.35 | 309.91 | 947.99 | 2.59 | 3.06 |
| Chad | Western sub-Saharan Africa | male | 6.30 | 31.50 | 207.96 | 1200.19 | 5.00 | 5.77 |
| Chile | Southern Latin America | male | 86.26 | 313.31 | 3461.65 | 14703.01 | 3.63 | 4.25 |
| China | East Asia | male | 2306.62 | 16244.41 | 158703.89 | 989258.31 | 7.04 | 6.23 |
| Colombia | Central Latin America | male | 135.88 | 462.44 | 8657.65 | 35518.46 | 3.40 | 4.10 |
| Comoros | Eastern Sub-Saharan Africa | male | 0.61 | 3.02 | 17.87 | 97.37 | 4.97 | 5.45 |
| Congo | Central sub-Saharan Africa | male | 16.05 | 79.52 | 550.61 | 3203.04 | 4.95 | 5.82 |
| Costa Rica | Central Latin America | male | 8.58 | 39.23 | 594.24 | 3556.63 | 4.57 | 5.99 |
| Côte d'Ivoire | Western sub-Saharan Africa | male | 27.03 | 147.51 | 949.75 | 5684.15 | 5.46 | 5.98 |
| Croatia | Central Europe | male | 45.83 | 73.54 | 2706.66 | 4453.46 | 1.60 | 1.65 |
| Cuba | Caribbean | male | 106.21 | 119.78 | 6647.09 | 12376.70 | 1.13 | 1.86 |
| Cyprus | Western Europe | male | 24.28 | 33.62 | 667.74 | 1164.63 | 1.38 | 1.74 |
| Czechia | Central Europe | male | 129.06 | 225.81 | 7901.93 | 15648.31 | 1.75 | 1.98 |
| Democratic Republic of the Congo | Central sub-Saharan Africa | male | 102.80 | 319.34 | 3549.96 | 13674.04 | 3.11 | 3.85 |
| Denmark | Western Europe | male | 56.07 | 64.42 | 1788.59 | 2120.78 | 1.15 | 1.19 |
| Djibouti | Eastern Sub-Saharan Africa | male | 1.02 | 20.20 | 38.32 | 750.82 | 19.85 | 19.59 |
| Dominica | Caribbean | male | 1.44 | 3.40 | 45.16 | 144.66 | 2.36 | 3.20 |
| Dominican Republic | Caribbean | male | 15.39 | 185.13 | 610.94 | 7422.69 | 12.03 | 12.15 |
| Ecuador | Andean Latin America | male | 45.57 | 354.34 | 1867.08 | 13408.70 | 7.78 | 7.18 |
| Egypt | North Africa and Middle East | male | 556.98 | 2284.29 | 20486.17 | 102305.71 | 4.10 | 4.99 |
| El Salvador | Central Latin America | male | 12.24 | 132.69 | 639.59 | 5947.14 | 10.84 | 9.30 |
| Equatorial Guinea | Central sub-Saharan Africa | male | 1.23 | 22.72 | 43.01 | 840.41 | 18.49 | 19.54 |
| Eritrea | Eastern Sub-Saharan Africa | male | 4.85 | 34.34 | 185.71 | 1389.47 | 7.08 | 7.48 |
| Estonia | Eastern Europe | male | 1.91 | 1.83 | 180.87 | 162.23 | 0.96 | 0.90 |
| Ethiopia | Eastern Sub-Saharan Africa | male | 77.79 | 302.10 | 2515.12 | 9251.05 | 3.88 | 3.68 |
| Micronesia (Federated States of) | Oceania | male | 0.52 | 3.06 | 18.25 | 114.23 | 5.83 | 6.26 |
| Fiji | Oceania | male | 7.74 | 62.97 | 260.10 | 2053.10 | 8.14 | 7.89 |
| Finland | Western Europe | male | 10.65 | 6.34 | 774.16 | 688.02 | 0.59 | 0.89 |
| France | Western Europe | male | 414.62 | 610.04 | 13545.20 | 19458.33 | 1.47 | 1.44 |
| Gabon | Central sub-Saharan Africa | male | 15.14 | 76.12 | 480.55 | 2703.71 | 5.03 | 5.63 |
| Georgia | Central Asia | male | 28.01 | 74.99 | 1403.31 | 3535.51 | 2.68 | 2.52 |
| Germany | Western Europe | male | 1077.16 | 1064.71 | 46111.53 | 57215.09 | 0.99 | 1.24 |
| Ghana | Western sub-Saharan Africa | male | 40.82 | 481.97 | 1451.48 | 16813.67 | 11.81 | 11.58 |
| Greece | Western Europe | male | 70.55 | 84.74 | 3875.22 | 6479.92 | 1.20 | 1.67 |
| Greenland | High-income North America | male | 0.11 | 0.16 | 4.41 | 9.62 | 1.52 | 2.18 |
| Grenada | Caribbean | male | 1.66 | 5.35 | 49.14 | 216.90 | 3.21 | 4.41 |
| Guam | Oceania | male | 0.66 | 1.38 | 30.98 | 75.95 | 2.09 | 2.45 |
| Guatemala | Central Latin America | male | 11.61 | 272.44 | 669.38 | 11410.96 | 23.46 | 17.05 |
| Guinea | Western sub-Saharan Africa | male | 9.44 | 39.52 | 292.41 | 1365.89 | 4.19 | 4.67 |
| Guinea-Bissau | Western sub-Saharan Africa | male | 2.41 | 7.28 | 76.47 | 270.09 | 3.01 | 3.53 |
| Guyana | Caribbean | male | 12.73 | 35.78 | 474.12 | 1423.93 | 2.81 | 3.00 |
| Haiti | Caribbean | male | 12.40 | 41.31 | 484.26 | 2075.96 | 3.33 | 4.29 |
| Honduras | Central Latin America | male | 4.41 | 39.03 | 343.76 | 2917.95 | 8.86 | 8.49 |
| Hungary | Central Europe | male | 92.59 | 148.62 | 5515.89 | 9352.08 | 1.61 | 1.70 |
| Iceland | Western Europe | male | 0.36 | 0.48 | 17.49 | 32.81 | 1.34 | 1.88 |
| India | South Asia | male | 2673.63 | 22450.85 | 129850.92 | 1081055.70 | 8.40 | 8.33 |
| Indonesia | Southeast Asia | male | 901.25 | 5883.17 | 34176.83 | 217472.19 | 6.53 | 6.36 |
| Iran (Islamic Republic of) | North Africa and Middle East | male | 225.70 | 1455.96 | 13913.92 | 76320.14 | 6.45 | 5.49 |
| Iraq | North Africa and Middle East | male | 287.41 | 1053.78 | 10359.89 | 45353.94 | 3.67 | 4.38 |
| Ireland | Western Europe | male | 21.71 | 16.07 | 641.39 | 953.30 | 0.74 | 1.49 |
| Israel | Western Europe | male | 62.22 | 204.98 | 1969.24 | 6446.35 | 3.29 | 3.27 |
| Italy | Western Europe | male | 1090.16 | 1271.13 | 37860.96 | 52947.22 | 1.17 | 1.40 |
| Jamaica | Caribbean | male | 24.16 | 119.48 | 699.06 | 3764.91 | 4.94 | 5.39 |
| Japan | High-income Asia Pacific | male | 534.77 | 515.13 | 35732.90 | 60619.89 | 0.96 | 1.70 |
| Jordan | North Africa and Middle East | male | 51.10 | 224.08 | 1877.09 | 10375.72 | 4.39 | 5.53 |
| Kazakhstan | Central Asia | male | 27.22 | 132.73 | 2203.91 | 8580.66 | 4.88 | 3.89 |
| Kenya | Eastern Sub-Saharan Africa | male | 30.71 | 227.09 | 1001.45 | 8414.89 | 7.39 | 8.40 |
| Kiribati | Oceania | male | 0.41 | 1.81 | 14.75 | 69.00 | 4.45 | 4.68 |
| Kuwait | North Africa and Middle East | male | 15.60 | 54.04 | 1065.65 | 5242.77 | 3.46 | 4.92 |
| Kyrgyzstan | Central Asia | male | 5.70 | 17.23 | 349.04 | 1336.44 | 3.02 | 3.83 |
| Lao People's Democratic Republic | Southeast Asia | male | 6.78 | 31.17 | 265.37 | 1460.13 | 4.59 | 5.50 |
| Latvia | Eastern Europe | male | 7.98 | 11.73 | 627.28 | 758.09 | 1.47 | 1.21 |
| Lebanon | North Africa and Middle East | male | 37.16 | 102.63 | 1623.55 | 5651.31 | 2.76 | 3.48 |
| Lesotho | Southern sub-Saharan Africa | male | 8.78 | 46.45 | 268.47 | 1461.80 | 5.29 | 5.44 |
| Liberia | Western sub-Saharan Africa | male | 4.76 | 15.87 | 162.07 | 695.26 | 3.34 | 4.29 |
| Libya | North Africa and Middle East | male | 18.38 | 86.59 | 1142.14 | 6955.06 | 4.71 | 6.09 |
| Lithuania | Eastern Europe | male | 8.66 | 8.54 | 725.45 | 725.03 | 0.99 | 1.00 |
| Luxembourg | Western Europe | male | 2.86 | 2.63 | 99.05 | 257.68 | 0.92 | 2.60 |
| North Macedonia | Central Europe | male | 21.20 | 80.14 | 1054.25 | 3652.40 | 3.78 | 3.46 |
| Madagascar | Eastern Sub-Saharan Africa | male | 10.21 | 39.17 | 329.74 | 1590.69 | 3.84 | 4.82 |
| Malawi | Eastern Sub-Saharan Africa | male | 9.78 | 40.73 | 325.30 | 1529.77 | 4.16 | 4.70 |
| Malaysia | Southeast Asia | male | 166.97 | 225.81 | 7372.48 | 16461.07 | 1.35 | 2.23 |
| Maldives | Southeast Asia | male | 0.43 | 2.31 | 16.53 | 123.29 | 5.38 | 7.46 |
| Mali | Western sub-Saharan Africa | male | 10.06 | 43.46 | 336.44 | 1602.44 | 4.32 | 4.76 |
| Malta | Western Europe | male | 5.02 | 6.99 | 179.14 | 319.60 | 1.39 | 1.78 |
| Marshall Islands | Oceania | male | 0.12 | 0.65 | 5.34 | 35.23 | 5.31 | 6.59 |
| Mauritania | Western sub-Saharan Africa | male | 8.56 | 34.50 | 250.48 | 1024.78 | 4.03 | 4.09 |
| Mauritius | Southeast Asia | male | 16.11 | 123.85 | 644.60 | 4077.43 | 7.69 | 6.33 |
| Mexico | Central Latin America | male | 1594.43 | 5373.79 | 62547.48 | 206733.66 | 3.37 | 3.31 |
| Republic of Moldova | Eastern Europe | male | 11.69 | 13.71 | 859.32 | 1408.14 | 1.17 | 1.64 |
| Mongolia | Central Asia | male | 1.60 | 11.09 | 85.11 | 743.75 | 6.95 | 8.74 |
| Montenegro | Central Europe | male | 4.67 | 9.55 | 290.31 | 625.86 | 2.05 | 2.16 |
| Morocco | North Africa and Middle East | male | 62.94 | 548.19 | 3362.78 | 31205.22 | 8.71 | 9.28 |
| Mozambique | Eastern Sub-Saharan Africa | male | 9.54 | 59.31 | 311.66 | 2139.20 | 6.22 | 6.86 |
| Myanmar | Southeast Asia | male | 189.94 | 953.50 | 6373.45 | 33519.81 | 5.02 | 5.26 |
| Namibia | Southern sub-Saharan Africa | male | 9.15 | 43.27 | 269.25 | 1311.67 | 4.73 | 4.87 |
| Nepal | South Asia | male | 17.85 | 211.42 | 1042.78 | 12212.06 | 11.84 | 11.71 |
| Netherlands | Western Europe | male | 201.32 | 161.58 | 7261.38 | 7697.32 | 0.80 | 1.06 |
| New Zealand | Australasia | male | 9.48 | 12.41 | 326.90 | 535.53 | 1.31 | 1.64 |
| Nicaragua | Central Latin America | male | 6.08 | 74.61 | 265.55 | 3157.34 | 12.28 | 11.89 |
| Niger | Western sub-Saharan Africa | male | 7.02 | 29.83 | 216.87 | 1052.20 | 4.25 | 4.85 |
| Nigeria | Western sub-Saharan Africa | male | 298.01 | 1604.65 | 9280.50 | 48082.32 | 5.38 | 5.18 |
| Democratic People's Republic of Korea | East Asia | male | 39.96 | 162.76 | 2139.09 | 9874.19 | 4.07 | 4.62 |
| Northern Mariana Islands | Oceania | male | 0.28 | 0.98 | 14.37 | 42.83 | 3.47 | 2.98 |
| Norway | Western Europe | male | 21.87 | 12.34 | 1146.18 | 846.08 | 0.56 | 0.74 |
| Oman | North Africa and Middle East | male | 14.53 | 82.34 | 584.77 | 4090.37 | 5.67 | 6.99 |
| Pakistan | South Asia | male | 324.10 | 2870.78 | 12910.74 | 123610.54 | 8.86 | 9.57 |
| Palestine | North Africa and Middle East | male | 19.80 | 125.93 | 602.06 | 4842.96 | 6.36 | 8.04 |
| Panama | Central Latin America | male | 8.66 | 60.89 | 478.79 | 2973.89 | 7.03 | 6.21 |
| Papua New Guinea | Oceania | male | 16.03 | 81.81 | 576.38 | 3145.49 | 5.10 | 5.46 |
| Paraguay | Tropical Latin America | male | 8.41 | 87.89 | 328.68 | 3129.78 | 10.46 | 9.52 |
| Peru | Andean Latin America | male | 76.97 | 382.00 | 3072.85 | 16859.13 | 4.96 | 5.49 |
| Philippines | Southeast Asia | male | 395.31 | 1346.65 | 15520.41 | 51019.61 | 3.41 | 3.29 |
| Poland | Central Europe | male | 328.44 | 599.12 | 19887.45 | 40843.86 | 1.82 | 2.05 |
| Portugal | Western Europe | male | 105.47 | 117.38 | 3822.70 | 4714.89 | 1.11 | 1.23 |
| Puerto Rico | Caribbean | male | 38.26 | 70.73 | 1392.13 | 2548.91 | 1.85 | 1.83 |
| Qatar | North Africa and Middle East | male | 8.68 | 55.03 | 408.07 | 5195.04 | 6.34 | 12.73 |
| Romania | Central Europe | male | 100.93 | 146.77 | 6575.83 | 11121.76 | 1.45 | 1.69 |
| Russian Federation | Eastern Europe | male | 266.81 | 509.81 | 23231.43 | 33279.04 | 1.91 | 1.43 |
| Rwanda | Eastern Sub-Saharan Africa | male | 18.41 | 52.05 | 575.56 | 1821.80 | 2.83 | 3.17 |
| Saint Lucia | Caribbean | male | 2.35 | 9.47 | 79.97 | 377.74 | 4.02 | 4.72 |
| Saint Vincent and the Grenadines | Caribbean | male | 1.73 | 7.80 | 57.94 | 284.74 | 4.52 | 4.91 |
| Samoa | Oceania | male | 1.18 | 2.81 | 39.80 | 105.83 | 2.38 | 2.66 |
| Sao Tome and Principe | Central sub-Saharan Africa | male | 0.12 | 0.78 | 5.30 | 39.29 | 6.46 | 7.41 |
| Saudi Arabia | North Africa and Middle East | male | 90.83 | 420.05 | 4451.25 | 36055.57 | 4.62 | 8.10 |
| Senegal | Western sub-Saharan Africa | male | 18.62 | 86.91 | 678.25 | 3480.07 | 4.67 | 5.13 |
| Serbia | Central Europe | male | 117.43 | 273.94 | 5860.33 | 13141.55 | 2.33 | 2.24 |
| Seychelles | Southeast Asia | male | 0.38 | 1.50 | 20.85 | 106.45 | 3.92 | 5.10 |
| Sierra Leone | Western sub-Saharan Africa | male | 6.78 | 21.56 | 182.80 | 710.18 | 3.18 | 3.89 |
| Singapore | High-income Asia Pacific | male | 22.90 | 14.05 | 1444.98 | 3681.21 | 0.61 | 2.55 |
| Slovakia | Central Europe | male | 61.67 | 54.58 | 2947.47 | 3973.53 | 0.89 | 1.35 |
| Slovenia | Central Europe | male | 14.72 | 24.94 | 881.84 | 1632.99 | 1.69 | 1.85 |
| Solomon Islands | Oceania | male | 0.55 | 4.70 | 20.93 | 194.62 | 8.59 | 9.30 |
| Somalia | Eastern Sub-Saharan Africa | male | 4.04 | 12.38 | 140.14 | 486.59 | 3.07 | 3.47 |
| South Africa | Southern sub-Saharan Africa | male | 447.30 | 1940.97 | 14855.20 | 63198.07 | 4.34 | 4.25 |
| Republic of Korea | High-income Asia Pacific | male | 459.79 | 1284.68 | 20963.25 | 58558.63 | 2.79 | 2.79 |
| South Sudan | Eastern Sub-Saharan Africa | male | 12.04 | 30.97 | 377.65 | 1083.79 | 2.57 | 2.87 |
| Spain | Western Europe | male | 378.22 | 343.07 | 16306.74 | 20840.11 | 0.91 | 1.28 |
| Sri Lanka | Southeast Asia | male | 81.64 | 706.94 | 3321.81 | 27302.09 | 8.66 | 8.22 |
| Sudan | North Africa and Middle East | male | 15.55 | 202.49 | 864.66 | 13985.61 | 13.02 | 16.17 |
| Suriname | Caribbean | male | 4.51 | 19.38 | 198.47 | 932.99 | 4.30 | 4.70 |
| Eswatini | Southern sub-Saharan Africa | male | 4.44 | 34.53 | 133.45 | 1057.57 | 7.78 | 7.92 |
| Sweden | Western Europe | male | 50.37 | 30.24 | 1901.09 | 1249.56 | 0.60 | 0.66 |
| Switzerland | Western Europe | male | 86.43 | 55.54 | 3036.94 | 3001.15 | 0.64 | 0.99 |
| Syrian Arab Republic | North Africa and Middle East | male | 69.10 | 159.26 | 3584.67 | 11345.61 | 2.30 | 3.17 |
| Taiwan (Province of China) | East Asia | male | 273.17 | 970.83 | 10436.72 | 35049.21 | 3.55 | 3.36 |
| Tajikistan | Central Asia | male | 11.27 | 116.11 | 530.07 | 5417.16 | 10.30 | 10.22 |
| United Republic of Tanzania | Eastern Sub-Saharan Africa | male | 29.47 | 176.93 | 869.12 | 5854.82 | 6.00 | 6.74 |
| Thailand | Southeast Asia | male | 357.78 | 1379.64 | 14989.19 | 69304.88 | 3.86 | 4.62 |
| Bahamas | Caribbean | male | 3.53 | 8.37 | 140.72 | 405.26 | 2.37 | 2.88 |
| Gambia | Western sub-Saharan Africa | male | 1.28 | 10.41 | 44.55 | 361.84 | 8.13 | 8.12 |
| Timor-Leste | Southeast Asia | male | 0.29 | 3.29 | 13.77 | 166.62 | 11.52 | 12.10 |
| Togo | Western sub-Saharan Africa | male | 4.90 | 31.06 | 158.62 | 1141.97 | 6.34 | 7.20 |
| Tonga | Oceania | male | 0.54 | 2.40 | 17.92 | 77.61 | 4.44 | 4.33 |
| Trinidad and Tobago | Caribbean | male | 71.73 | 158.09 | 2336.49 | 5280.94 | 2.20 | 2.26 |
| Tunisia | North Africa and Middle East | male | 37.45 | 181.66 | 2636.81 | 13687.40 | 4.85 | 5.19 |
| Turkey | North Africa and Middle East | male | 819.76 | 1579.92 | 28669.98 | 71945.69 | 1.93 | 2.51 |
| Turkmenistan | Central Asia | male | 14.59 | 57.22 | 754.53 | 3166.35 | 3.92 | 4.20 |
| Uganda | Eastern Sub-Saharan Africa | male | 22.76 | 140.47 | 720.87 | 5191.60 | 6.17 | 7.20 |
| Ukraine | Eastern Europe | male | 145.42 | 130.82 | 12555.83 | 13575.77 | 0.90 | 1.08 |
| United Arab Emirates | North Africa and Middle East | male | 24.40 | 228.26 | 1165.94 | 16080.74 | 9.36 | 13.79 |
| United Kingdom | Western Europe | male | 480.17 | 247.96 | 22956.77 | 29890.00 | 0.52 | 1.30 |
| United States of America | High-income North America | male | 2170.81 | 2253.50 | 105704.87 | 131230.63 | 1.04 | 1.24 |
| Uruguay | Southern Latin America | male | 22.19 | 32.47 | 554.63 | 1134.05 | 1.46 | 2.04 |
| Uzbekistan | Central Asia | male | 58.84 | 667.02 | 3044.70 | 29682.58 | 11.34 | 9.75 |
| Vanuatu | Oceania | male | 0.26 | 2.01 | 10.97 | 84.60 | 7.66 | 7.71 |
| Venezuela (Bolivarian Republic of) | Central Latin America | male | 204.28 | 954.55 | 8725.08 | 39086.30 | 4.67 | 4.48 |
| Viet nam | Southeast Asia | male | 138.30 | 1263.95 | 4870.45 | 50230.12 | 9.14 | 10.31 |
| United States Virgin Islands | Caribbean | male | 0.85 | 2.93 | 36.24 | 113.60 | 3.44 | 3.13 |
| Yemen | North Africa and Middle East | male | 6.78 | 90.28 | 376.79 | 5978.10 | 13.32 | 15.87 |
| Zambia | Eastern Sub-Saharan Africa | male | 15.61 | 101.37 | 490.96 | 3652.20 | 6.49 | 7.44 |
| Zimbabwe | Southern sub-Saharan Africa | male | 20.38 | 68.31 | 797.60 | 2718.08 | 3.35 | 3.41 |
| Monaco | Western Europe | male | 0.10 | 0.22 | 5.58 | 16.53 | 2.13 | 2.96 |
| San Marino | Western Europe | male | 0.12 | 0.20 | 5.18 | 11.04 | 1.70 | 2.13 |
| Saint Kitts and Nevis | Caribbean | male | 0.55 | 1.21 | 17.08 | 51.75 | 2.20 | 3.03 |
| Cook Islands | Oceania | male | 0.27 | 0.58 | 7.96 | 17.98 | 2.17 | 2.26 |
| Nauru | Oceania | male | 0.06 | 0.09 | 2.17 | 4.06 | 1.69 | 1.87 |
| Niue | Oceania | male | 0.03 | 0.06 | 0.85 | 2.00 | 2.23 | 2.36 |
| Palau | Oceania | male | 0.15 | 0.55 | 5.59 | 21.80 | 3.57 | 3.90 |
| Tokelau | Oceania | male | 0.01 | 0.02 | 0.43 | 0.66 | 1.31 | 1.53 |
| Tuvalu | Oceania | male | 0.03 | 0.15 | 1.09 | 5.63 | 4.87 | 5.17 |
| Afghanistan | North Africa and Middle East | female | 23.01 | 173.76 | 863.71 | 7835.52 | 7.55 | 9.07 |
| Albania | Central Europe | female | 2.58 | 9.06 | 171.78 | 828.94 | 3.51 | 4.83 |
| Algeria | North Africa and Middle East | female | 136.94 | 618.47 | 6692.01 | 37480.97 | 4.52 | 5.60 |
| American Samoa | Oceania | female | 0.22 | 0.89 | 8.89 | 37.43 | 4.15 | 4.21 |
| Andorra | Western Europe | female | 0.13 | 0.26 | 7.34 | 17.21 | 1.94 | 2.35 |
| Angola | Central sub-Saharan Africa | female | 6.71 | 111.61 | 271.82 | 4833.15 | 16.63 | 17.78 |
| Antigua and Barbuda | Caribbean | female | 2.38 | 4.58 | 63.45 | 154.23 | 1.92 | 2.43 |
| Argentina | Southern Latin America | female | 339.26 | 571.77 | 10630.64 | 22106.33 | 1.69 | 2.08 |
| Armenia | Central Asia | female | 39.32 | 144.52 | 1548.73 | 5171.25 | 3.67 | 3.34 |
| Australia | Australasia | female | 60.16 | 86.28 | 1897.53 | 3429.60 | 1.43 | 1.81 |
| Austria | Western Europe | female | 167.62 | 132.35 | 4431.45 | 4457.36 | 0.79 | 1.01 |
| Azerbaijan | Central Asia | female | 27.07 | 186.44 | 1338.15 | 9322.69 | 6.89 | 6.97 |
| Bahrain | North Africa and Middle East | female | 9.08 | 69.83 | 324.94 | 2687.97 | 7.69 | 8.27 |
| Bangladesh | South Asia | female | 111.43 | 1543.49 | 4179.01 | 53488.46 | 13.85 | 12.80 |
| Barbados | Caribbean | female | 23.22 | 30.77 | 557.82 | 854.74 | 1.32 | 1.53 |
| Belarus | Eastern Europe | female | 61.53 | 31.00 | 4051.34 | 3534.88 | 0.50 | 0.87 |
| Belgium | Western Europe | female | 202.53 | 111.09 | 5964.12 | 5519.33 | 0.55 | 0.93 |
| Belize | Caribbean | female | 1.47 | 9.82 | 42.07 | 361.18 | 6.68 | 8.58 |
| Benin | Western sub-Saharan Africa | female | 4.20 | 33.31 | 136.72 | 1199.57 | 7.92 | 8.77 |
| Bermuda | Caribbean | female | 0.71 | 0.56 | 20.97 | 22.56 | 0.78 | 1.08 |
| Bhutan | South Asia | female | 0.36 | 6.77 | 14.85 | 243.04 | 18.93 | 16.37 |
| Bolivia (Plurinational State of) | Andean Latin America | female | 61.28 | 273.78 | 1864.07 | 8177.66 | 4.47 | 4.39 |
| Bosnia and Herzegovina | Central Europe | female | 27.19 | 204.29 | 1162.11 | 6336.82 | 7.51 | 5.45 |
| Botswana | Southern sub-Saharan Africa | female | 6.18 | 67.91 | 178.93 | 2073.99 | 11.00 | 11.59 |
| Brazil | Tropical Latin America | female | 972.06 | 2963.22 | 35853.86 | 107746.25 | 3.05 | 3.01 |
| Brunei Darussalam | High-income Asia Pacific | female | 2.12 | 4.28 | 71.24 | 183.09 | 2.02 | 2.57 |
| Bulgaria | Central Europe | female | 150.02 | 170.36 | 6005.19 | 7502.16 | 1.14 | 1.25 |
| Burkina Faso | Western sub-Saharan Africa | female | 7.48 | 33.80 | 265.04 | 1370.98 | 4.52 | 5.17 |
| Burundi | Eastern Sub-Saharan Africa | female | 6.20 | 11.24 | 195.85 | 414.13 | 1.81 | 2.11 |
| Cambodia | Southeast Asia | female | 11.78 | 78.64 | 399.75 | 3125.75 | 6.68 | 7.82 |
| Cameroon | Western sub-Saharan Africa | female | 29.49 | 254.67 | 912.42 | 8241.95 | 8.64 | 9.03 |
| Canada | High-income North America | female | 179.35 | 164.32 | 4502.49 | 5957.76 | 0.92 | 1.32 |
| Cabo Verde | Western sub-Saharan Africa | female | 0.45 | 13.73 | 20.95 | 428.68 | 30.36 | 20.46 |
| Central African Republic | Central sub-Saharan Africa | female | 2.98 | 9.94 | 116.08 | 425.95 | 3.33 | 3.67 |
| Chad | Western sub-Saharan Africa | female | 4.68 | 23.86 | 153.18 | 869.92 | 5.10 | 5.68 |
| Chile | Southern Latin America | female | 84.20 | 339.55 | 3367.46 | 15152.18 | 4.03 | 4.50 |
| China | East Asia | female | 2510.85 | 15557.01 | 132139.39 | 826694.93 | 6.20 | 6.26 |
| Colombia | Central Latin America | female | 170.13 | 590.39 | 7964.78 | 35144.23 | 3.47 | 4.41 |
| Comoros | Eastern Sub-Saharan Africa | female | 0.38 | 2.34 | 11.05 | 68.20 | 6.11 | 6.17 |
| Congo | Central sub-Saharan Africa | female | 7.28 | 58.08 | 257.25 | 2233.95 | 7.98 | 8.68 |
| Costa Rica | Central Latin America | female | 10.83 | 42.60 | 512.91 | 3057.44 | 3.93 | 5.96 |
| Côte d'Ivoire | Western sub-Saharan Africa | female | 11.71 | 102.35 | 429.84 | 3855.26 | 8.74 | 8.97 |
| Croatia | Central Europe | female | 69.98 | 94.51 | 3135.81 | 4454.06 | 1.35 | 1.42 |
| Cuba | Caribbean | female | 186.09 | 167.44 | 7198.08 | 10822.08 | 0.90 | 1.50 |
| Cyprus | Western Europe | female | 38.57 | 35.24 | 821.40 | 1027.89 | 0.91 | 1.25 |
| Czechia | Central Europe | female | 178.64 | 256.79 | 8775.46 | 14232.04 | 1.44 | 1.62 |
| Democratic Republic of the Congo | Central sub-Saharan Africa | female | 37.08 | 142.89 | 1487.82 | 6360.15 | 3.85 | 4.27 |
| Denmark | Western Europe | female | 58.61 | 52.63 | 1634.46 | 1705.43 | 0.90 | 1.04 |
| Djibouti | Eastern Sub-Saharan Africa | female | 0.57 | 10.85 | 20.77 | 405.00 | 19.11 | 19.50 |
| Dominica | Caribbean | female | 2.34 | 4.61 | 57.46 | 131.56 | 1.97 | 2.29 |
| Dominican Republic | Caribbean | female | 13.72 | 161.77 | 490.47 | 5999.58 | 11.79 | 12.23 |
| Ecuador | Andean Latin America | female | 53.86 | 399.84 | 1886.98 | 14046.38 | 7.42 | 7.44 |
| Egypt | North Africa and Middle East | female | 686.31 | 2335.12 | 24595.24 | 97249.29 | 3.40 | 3.95 |
| El Salvador | Central Latin America | female | 12.51 | 179.08 | 518.86 | 6576.02 | 14.32 | 12.67 |
| Equatorial Guinea | Central sub-Saharan Africa | female | 0.45 | 17.54 | 17.32 | 645.27 | 38.65 | 37.26 |
| Eritrea | Eastern Sub-Saharan Africa | female | 3.13 | 28.90 | 115.62 | 1017.80 | 9.25 | 8.80 |
| Estonia | Eastern Europe | female | 2.84 | 2.87 | 230.47 | 206.50 | 1.01 | 0.90 |
| Ethiopia | Eastern Sub-Saharan Africa | female | 33.62 | 145.46 | 1177.21 | 4962.68 | 4.33 | 4.22 |
| Micronesia (Federated States of) | Oceania | female | 0.40 | 2.70 | 12.77 | 90.93 | 6.74 | 7.12 |
| Fiji | Oceania | female | 5.28 | 57.52 | 177.01 | 1781.19 | 10.89 | 10.06 |
| Finland | Western Europe | female | 21.83 | 6.23 | 1054.44 | 720.34 | 0.29 | 0.68 |
| France | Western Europe | female | 641.97 | 668.71 | 14917.66 | 17747.27 | 1.04 | 1.19 |
| Gabon | Central sub-Saharan Africa | female | 5.55 | 42.37 | 172.28 | 1423.42 | 7.64 | 8.26 |
| Georgia | Central Asia | female | 28.41 | 75.93 | 1430.60 | 3379.95 | 2.67 | 2.36 |
| Germany | Western Europe | female | 2349.61 | 1306.96 | 69926.41 | 55180.55 | 0.56 | 0.79 |
| Ghana | Western sub-Saharan Africa | female | 39.13 | 295.25 | 1375.32 | 11249.36 | 7.55 | 8.18 |
| Greece | Western Europe | female | 110.70 | 92.19 | 4332.23 | 5753.49 | 0.83 | 1.33 |
| Greenland | High-income North America | female | 0.10 | 0.07 | 2.67 | 4.22 | 0.77 | 1.58 |
| Grenada | Caribbean | female | 2.30 | 6.62 | 59.23 | 212.27 | 2.88 | 3.58 |
| Guam | Oceania | female | 1.00 | 1.60 | 36.99 | 74.04 | 1.60 | 2.00 |
| Guatemala | Central Latin America | female | 10.23 | 315.73 | 524.15 | 11927.28 | 30.85 | 22.76 |
| Guinea | Western sub-Saharan Africa | female | 8.60 | 37.14 | 255.94 | 1173.99 | 4.32 | 4.59 |
| Guinea-Bissau | Western sub-Saharan Africa | female | 1.26 | 6.79 | 43.16 | 239.78 | 5.40 | 5.56 |
| Guyana | Caribbean | female | 14.47 | 42.66 | 502.69 | 1550.11 | 2.95 | 3.08 |
| Haiti | Caribbean | female | 21.79 | 76.90 | 719.70 | 3017.12 | 3.53 | 4.19 |
| Honduras | Central Latin America | female | 3.20 | 34.15 | 216.89 | 2278.66 | 10.67 | 10.51 |
| Hungary | Central Europe | female | 150.05 | 179.74 | 6500.37 | 9055.09 | 1.20 | 1.39 |
| Iceland | Western Europe | female | 0.37 | 0.24 | 16.24 | 24.34 | 0.65 | 1.50 |
| India | South Asia | female | 1818.56 | 19555.32 | 82230.50 | 853724.96 | 10.75 | 10.38 |
| Indonesia | Southeast Asia | female | 822.88 | 5262.91 | 30149.58 | 184920.76 | 6.40 | 6.13 |
| Iran (Islamic Republic of) | North Africa and Middle East | female | 258.22 | 1706.08 | 12862.96 | 82522.85 | 6.61 | 6.42 |
| Iraq | North Africa and Middle East | female | 309.47 | 1006.51 | 10614.30 | 44867.99 | 3.25 | 4.23 |
| Ireland | Western Europe | female | 23.53 | 13.43 | 612.03 | 764.32 | 0.57 | 1.25 |
| Israel | Western Europe | female | 77.28 | 231.37 | 2346.71 | 6390.68 | 2.99 | 2.72 |
| Italy | Western Europe | female | 1911.11 | 1589.85 | 50365.21 | 51886.34 | 0.83 | 1.03 |
| Jamaica | Caribbean | female | 32.40 | 166.90 | 851.25 | 4445.59 | 5.15 | 5.22 |
| Japan | High-income Asia Pacific | female | 597.09 | 533.03 | 27878.47 | 46492.95 | 0.89 | 1.67 |
| Jordan | North Africa and Middle East | female | 82.01 | 200.88 | 2381.83 | 7873.82 | 2.45 | 3.31 |
| Kazakhstan | Central Asia | female | 52.13 | 225.22 | 3728.38 | 13846.84 | 4.32 | 3.71 |
| Kenya | Eastern Sub-Saharan Africa | female | 15.38 | 107.24 | 508.59 | 3944.87 | 6.97 | 7.76 |
| Kiribati | Oceania | female | 0.35 | 1.23 | 11.84 | 42.75 | 3.47 | 3.61 |
| Kuwait | North Africa and Middle East | female | 14.75 | 30.88 | 668.45 | 3219.32 | 2.09 | 4.82 |
| Kyrgyzstan | Central Asia | female | 6.55 | 19.13 | 366.34 | 1435.23 | 2.92 | 3.92 |
| Lao People's Democratic Republic | Southeast Asia | female | 6.50 | 30.31 | 233.52 | 1237.62 | 4.66 | 5.30 |
| Latvia | Eastern Europe | female | 15.60 | 22.54 | 947.54 | 1068.97 | 1.45 | 1.13 |
| Lebanon | North Africa and Middle East | female | 27.41 | 68.37 | 1326.07 | 5094.36 | 2.49 | 3.84 |
| Lesotho | Southern sub-Saharan Africa | female | 7.50 | 49.99 | 204.76 | 1380.28 | 6.66 | 6.74 |
| Liberia | Western sub-Saharan Africa | female | 3.05 | 14.54 | 100.96 | 552.36 | 4.77 | 5.47 |
| Libya | North Africa and Middle East | female | 18.23 | 98.31 | 910.40 | 6800.44 | 5.39 | 7.47 |
| Lithuania | Eastern Europe | female | 12.44 | 11.62 | 889.64 | 844.25 | 0.93 | 0.95 |
| Luxembourg | Western Europe | female | 4.89 | 3.18 | 127.93 | 223.98 | 0.65 | 1.75 |
| North Macedonia | Central Europe | female | 27.97 | 101.19 | 1090.82 | 3696.09 | 3.62 | 3.39 |
| Madagascar | Eastern Sub-Saharan Africa | female | 5.79 | 26.34 | 200.03 | 1045.52 | 4.55 | 5.23 |
| Malawi | Eastern Sub-Saharan Africa | female | 6.09 | 20.12 | 203.61 | 727.79 | 3.31 | 3.57 |
| Malaysia | Southeast Asia | female | 215.18 | 247.05 | 8275.02 | 16500.56 | 1.15 | 1.99 |
| Maldives | Southeast Asia | female | 0.32 | 1.43 | 11.96 | 73.20 | 4.44 | 6.12 |
| Mali | Western sub-Saharan Africa | female | 9.54 | 48.15 | 311.78 | 1637.90 | 5.05 | 5.25 |
| Malta | Western Europe | female | 8.24 | 7.50 | 237.02 | 336.57 | 0.91 | 1.42 |
| Marshall Islands | Oceania | female | 0.10 | 0.78 | 4.02 | 34.33 | 7.39 | 8.53 |
| Mauritania | Western sub-Saharan Africa | female | 8.10 | 44.74 | 228.25 | 1324.51 | 5.52 | 5.80 |
| Mauritius | Southeast Asia | female | 15.55 | 118.54 | 560.70 | 3575.88 | 7.62 | 6.38 |
| Mexico | Central Latin America | female | 1859.53 | 4971.29 | 65582.35 | 181291.94 | 2.67 | 2.76 |
| Republic of Moldova | Eastern Europe | female | 15.17 | 17.81 | 1006.55 | 1727.64 | 1.17 | 1.72 |
| Mongolia | Central Asia | female | 0.98 | 5.98 | 59.73 | 544.05 | 6.13 | 9.11 |
| Montenegro | Central Europe | female | 5.83 | 10.74 | 290.40 | 615.13 | 1.84 | 2.12 |
| Morocco | North Africa and Middle East | female | 69.57 | 735.12 | 3160.88 | 36025.10 | 10.57 | 11.40 |
| Mozambique | Eastern Sub-Saharan Africa | female | 5.14 | 24.92 | 171.23 | 895.29 | 4.84 | 5.23 |
| Myanmar | Southeast Asia | female | 153.28 | 701.88 | 5324.43 | 25831.93 | 4.58 | 4.85 |
| Namibia | Southern sub-Saharan Africa | female | 8.21 | 40.98 | 233.90 | 1177.28 | 4.99 | 5.03 |
| Nepal | South Asia | female | 9.37 | 163.98 | 504.81 | 7852.74 | 17.50 | 15.56 |
| Netherlands | Western Europe | female | 352.94 | 197.06 | 8810.23 | 6626.24 | 0.56 | 0.75 |
| New Zealand | Australasia | female | 9.55 | 10.49 | 327.94 | 465.85 | 1.10 | 1.42 |
| Nicaragua | Central Latin America | female | 4.27 | 66.88 | 188.99 | 2635.23 | 15.65 | 13.94 |
| Niger | Western sub-Saharan Africa | female | 4.47 | 29.88 | 136.86 | 999.53 | 6.69 | 7.30 |
| Nigeria | Western sub-Saharan Africa | female | 189.95 | 1235.87 | 5304.94 | 39928.22 | 6.51 | 7.53 |
| Democratic People's Republic of Korea | East Asia | female | 55.01 | 215.33 | 2239.24 | 9766.45 | 3.91 | 4.36 |
| Northern Mariana Islands | Oceania | female | 0.22 | 0.73 | 9.55 | 31.95 | 3.29 | 3.35 |
| Norway | Western Europe | female | 26.60 | 13.20 | 1134.71 | 752.32 | 0.50 | 0.66 |
| Oman | North Africa and Middle East | female | 13.38 | 65.13 | 440.42 | 2618.99 | 4.87 | 5.95 |
| Pakistan | South Asia | female | 176.68 | 2868.15 | 6942.37 | 107191.82 | 16.23 | 15.44 |
| Palestine | North Africa and Middle East | female | 21.70 | 137.22 | 602.25 | 4374.52 | 6.32 | 7.26 |
| Panama | Central Latin America | female | 9.98 | 66.76 | 401.03 | 2672.34 | 6.69 | 6.66 |
| Papua New Guinea | Oceania | female | 4.73 | 28.59 | 188.77 | 1237.07 | 6.04 | 6.55 |
| Paraguay | Tropical Latin America | female | 9.71 | 87.60 | 323.35 | 2605.98 | 9.02 | 8.06 |
| Peru | Andean Latin America | female | 63.79 | 379.59 | 2431.69 | 15770.83 | 5.95 | 6.49 |
| Philippines | Southeast Asia | female | 273.98 | 1155.65 | 9413.12 | 41819.95 | 4.22 | 4.44 |
| Poland | Central Europe | female | 510.35 | 708.96 | 24126.98 | 38869.67 | 1.39 | 1.61 |
| Portugal | Western Europe | female | 150.40 | 160.28 | 4598.08 | 5407.76 | 1.07 | 1.18 |
| Puerto Rico | Caribbean | female | 47.64 | 77.52 | 1520.84 | 2560.28 | 1.63 | 1.68 |
| Qatar | North Africa and Middle East | female | 6.35 | 26.47 | 212.17 | 1740.32 | 4.17 | 8.20 |
| Romania | Central Europe | female | 106.52 | 157.58 | 5989.61 | 10765.13 | 1.48 | 1.80 |
| Russian Federation | Eastern Europe | female | 748.14 | 1272.47 | 43228.78 | 56317.19 | 1.70 | 1.30 |
| Rwanda | Eastern Sub-Saharan Africa | female | 11.43 | 37.22 | 364.69 | 1234.31 | 3.26 | 3.38 |
| Saint Lucia | Caribbean | female | 3.34 | 11.13 | 103.38 | 420.15 | 3.33 | 4.06 |
| Saint Vincent and the Grenadines | Caribbean | female | 3.10 | 8.70 | 82.70 | 275.69 | 2.81 | 3.33 |
| Samoa | Oceania | female | 0.72 | 2.41 | 23.34 | 83.66 | 3.36 | 3.58 |
| Sao Tome and Principe | Central sub-Saharan Africa | female | 0.06 | 0.39 | 3.36 | 25.66 | 6.17 | 7.64 |
| Saudi Arabia | North Africa and Middle East | female | 61.93 | 242.97 | 2580.08 | 21651.04 | 3.92 | 8.39 |
| Senegal | Western sub-Saharan Africa | female | 11.34 | 84.87 | 424.00 | 3090.44 | 7.49 | 7.29 |
| Serbia | Central Europe | female | 138.57 | 331.28 | 5563.83 | 12633.28 | 2.39 | 2.27 |
| Seychelles | Southeast Asia | female | 0.53 | 1.90 | 22.09 | 104.63 | 3.59 | 4.74 |
| Sierra Leone | Western sub-Saharan Africa | female | 3.79 | 22.99 | 102.96 | 701.41 | 6.06 | 6.81 |
| Singapore | High-income Asia Pacific | female | 35.38 | 14.40 | 1566.81 | 2490.84 | 0.41 | 1.59 |
| Slovakia | Central Europe | female | 82.35 | 70.13 | 3298.81 | 4188.08 | 0.85 | 1.27 |
| Slovenia | Central Europe | female | 26.31 | 26.42 | 1140.43 | 1467.67 | 1.00 | 1.29 |
| Solomon Islands | Oceania | female | 0.34 | 3.43 | 13.04 | 132.93 | 9.99 | 10.20 |
| Somalia | Eastern Sub-Saharan Africa | female | 2.21 | 8.59 | 79.12 | 316.03 | 3.88 | 3.99 |
| South Africa | Southern sub-Saharan Africa | female | 556.10 | 2618.77 | 16939.22 | 77810.89 | 4.71 | 4.59 |
| Republic of Korea | High-income Asia Pacific | female | 396.68 | 1299.45 | 17086.29 | 48817.75 | 3.28 | 2.86 |
| South Sudan | Eastern Sub-Saharan Africa | female | 5.64 | 14.52 | 170.01 | 543.30 | 2.58 | 3.20 |
| Spain | Western Europe | female | 693.31 | 508.47 | 21424.80 | 20927.87 | 0.73 | 0.98 |
| Sri Lanka | Southeast Asia | female | 43.96 | 696.00 | 1936.92 | 25063.36 | 15.83 | 12.94 |
| Sudan | North Africa and Middle East | female | 13.07 | 160.95 | 614.98 | 10391.53 | 12.31 | 16.90 |
| Suriname | Caribbean | female | 4.60 | 18.85 | 176.04 | 862.03 | 4.10 | 4.90 |
| Eswatini | Southern sub-Saharan Africa | female | 3.61 | 27.60 | 100.32 | 762.59 | 7.65 | 7.60 |
| Sweden | Western Europe | female | 60.20 | 28.66 | 1855.81 | 1022.95 | 0.48 | 0.55 |
| Switzerland | Western Europe | female | 143.23 | 64.69 | 3720.68 | 2750.41 | 0.45 | 0.74 |
| Syrian Arab Republic | North Africa and Middle East | female | 87.62 | 180.26 | 3772.27 | 11615.55 | 2.06 | 3.08 |
| Taiwan (Province of China) | East Asia | female | 302.09 | 969.61 | 9738.45 | 31046.47 | 3.21 | 3.19 |
| Tajikistan | Central Asia | female | 8.87 | 96.58 | 420.72 | 4668.67 | 10.89 | 11.10 |
| United Republic of Tanzania | Eastern Sub-Saharan Africa | female | 17.82 | 106.71 | 515.16 | 3452.34 | 5.99 | 6.70 |
| Thailand | Southeast Asia | female | 406.90 | 1705.37 | 14320.95 | 70997.43 | 4.19 | 4.96 |
| Bahamas | Caribbean | female | 5.39 | 9.98 | 179.31 | 428.77 | 1.85 | 2.39 |
| Gambia | Western sub-Saharan Africa | female | 0.73 | 9.36 | 23.95 | 295.77 | 12.89 | 12.35 |
| Timor-Leste | Southeast Asia | female | 0.34 | 3.44 | 12.62 | 135.72 | 10.15 | 10.75 |
| Togo | Western sub-Saharan Africa | female | 3.66 | 29.01 | 115.88 | 956.58 | 7.93 | 8.25 |
| Tonga | Oceania | female | 0.72 | 2.39 | 22.16 | 69.85 | 3.30 | 3.15 |
| Trinidad and Tobago | Caribbean | female | 82.92 | 148.89 | 2537.10 | 4658.30 | 1.80 | 1.84 |
| Tunisia | North Africa and Middle East | female | 36.46 | 180.86 | 2014.54 | 11699.17 | 4.96 | 5.81 |
| Turkey | North Africa and Middle East | female | 1138.11 | 2158.41 | 33807.96 | 80458.71 | 1.90 | 2.38 |
| Turkmenistan | Central Asia | female | 17.31 | 65.32 | 900.12 | 3623.45 | 3.77 | 4.03 |
| Uganda | Eastern Sub-Saharan Africa | female | 9.78 | 73.51 | 328.43 | 2759.72 | 7.52 | 8.40 |
| Ukraine | Eastern Europe | female | 254.82 | 169.66 | 18803.02 | 17220.81 | 0.67 | 0.92 |
| United Arab Emirates | North Africa and Middle East | female | 15.91 | 56.75 | 593.16 | 4230.80 | 3.57 | 7.13 |
| United Kingdom | Western Europe | female | 621.05 | 271.23 | 23568.03 | 24975.14 | 0.44 | 1.06 |
| United States of America | High-income North America | female | 2988.07 | 2019.76 | 115895.64 | 108493.20 | 0.68 | 0.94 |
| Uruguay | Southern Latin America | female | 25.73 | 36.60 | 583.86 | 1046.81 | 1.42 | 1.79 |
| Uzbekistan | Central Asia | female | 53.14 | 692.53 | 2561.07 | 29608.96 | 13.03 | 11.56 |
| Vanuatu | Oceania | female | 0.17 | 1.63 | 6.55 | 61.19 | 9.61 | 9.34 |
| Venezuela (Bolivarian Republic of) | Central Latin America | female | 240.03 | 905.20 | 9048.69 | 36108.75 | 3.77 | 3.99 |
| Viet nam | Southeast Asia | female | 231.52 | 1594.28 | 6310.57 | 50166.27 | 6.89 | 7.95 |
| United States Virgin Islands | Caribbean | female | 1.38 | 2.85 | 46.10 | 104.96 | 2.06 | 2.28 |
| Yemen | North Africa and Middle East | female | 7.10 | 112.05 | 316.64 | 6083.55 | 15.79 | 19.21 |
| Zambia | Eastern Sub-Saharan Africa | female | 7.95 | 48.35 | 275.22 | 1777.30 | 6.08 | 6.46 |
| Zimbabwe | Southern sub-Saharan Africa | female | 22.26 | 88.86 | 717.08 | 3088.87 | 3.99 | 4.31 |
| Monaco | Western Europe | female | 0.11 | 0.16 | 5.78 | 14.48 | 1.46 | 2.50 |
| San Marino | Western Europe | female | 0.14 | 0.20 | 5.09 | 10.29 | 1.44 | 2.02 |
| Saint Kitts and Nevis | Caribbean | female | 0.80 | 1.07 | 22.16 | 41.85 | 1.34 | 1.89 |
| Cook Islands | Oceania | female | 0.22 | 0.57 | 6.54 | 16.52 | 2.54 | 2.53 |
| Nauru | Oceania | female | 0.04 | 0.10 | 1.52 | 3.90 | 2.28 | 2.56 |
| Niue | Oceania | female | 0.03 | 0.07 | 0.81 | 1.98 | 2.09 | 2.46 |
| Palau | Oceania | female | 0.17 | 0.51 | 5.67 | 18.19 | 2.93 | 3.21 |
| Tokelau | Oceania | female | 0.04 | 0.04 | 1.00 | 1.09 | 1.01 | 1.09 |
| Tuvalu | Oceania | female | 0.04 | 0.20 | 1.25 | 6.37 | 5.16 | 5.12 |

**Supplementary Table 3. The number and its trend of age-standardized deaths and DALY rates type 2 diabetes burden attributed to ambient particulate matter pollution in 1990 and 2019, by countries and regions.**

| **Country** | **Region** | **Sex** | **Age-standardized Deaths Rate in 1990** | **Age-standardized Deaths Rate in 2019** | **Age-standardized DALYs Rate in 1990** | **Age-standardized DALYs Rate in 2019** |
| --- | --- | --- | --- | --- | --- | --- |
| Afghanistan | North Africa and Middle East | both | 0.60 | 2.32 | 21.57 | 92.54 |
| Albania | Central Europe | both | 0.30 | 0.43 | 19.44 | 43.95 |
| Algeria | North Africa and Middle East | both | 2.78 | 3.88 | 104.36 | 201.86 |
| American Samoa | Oceania | both | 2.89 | 4.24 | 96.90 | 159.88 |
| Andorra | Western Europe | both | 0.92 | 0.43 | 35.86 | 29.89 |
| Angola | Central sub-Saharan Africa | both | 0.77 | 3.72 | 23.56 | 117.01 |
| Antigua and Barbuda | Caribbean | both | 7.73 | 8.71 | 231.89 | 283.26 |
| Argentina | Southern Latin America | both | 2.22 | 2.17 | 67.32 | 87.05 |
| Armenia | Central Asia | both | 2.49 | 5.83 | 94.05 | 216.39 |
| Australia | Australasia | both | 0.62 | 0.40 | 20.25 | 19.18 |
| Austria | Western Europe | both | 2.06 | 1.23 | 63.96 | 54.51 |
| Azerbaijan | Central Asia | both | 1.04 | 4.03 | 48.54 | 169.38 |
| Bahrain | North Africa and Middle East | both | 17.14 | 30.34 | 455.17 | 770.18 |
| Bangladesh | South Asia | both | 0.89 | 3.15 | 24.63 | 90.83 |
| Barbados | Caribbean | both | 11.44 | 10.47 | 312.14 | 318.00 |
| Belarus | Eastern Europe | both | 0.73 | 0.32 | 51.43 | 40.53 |
| Belgium | Western Europe | both | 1.86 | 0.72 | 67.24 | 53.02 |
| Belize | Caribbean | both | 2.81 | 7.51 | 81.90 | 244.66 |
| Benin | Western sub-Saharan Africa | both | 0.61 | 1.76 | 17.62 | 54.43 |
| Bermuda | Caribbean | both | 2.28 | 0.87 | 68.81 | 40.21 |
| Bhutan | South Asia | both | 0.39 | 3.15 | 13.07 | 99.97 |
| Bolivia (Plurinational State of) | Andean Latin America | both | 3.47 | 5.84 | 101.93 | 172.58 |
| Bosnia and Herzegovina | Central Europe | both | 1.26 | 6.03 | 54.56 | 211.79 |
| Botswana | Southern sub-Saharan Africa | both | 2.59 | 11.46 | 67.93 | 300.19 |
| Brazil | Tropical Latin America | both | 2.14 | 2.42 | 76.26 | 90.46 |
| Brunei Darussalam | High-income Asia Pacific | both | 6.28 | 4.27 | 171.12 | 137.28 |
| Bulgaria | Central Europe | both | 2.26 | 2.06 | 95.30 | 109.03 |
| Burkina Faso | Western sub-Saharan Africa | both | 0.63 | 1.26 | 16.92 | 38.11 |
| Burundi | Eastern Sub-Saharan Africa | both | 0.80 | 0.91 | 21.80 | 26.40 |
| Cambodia | Southeast Asia | both | 0.60 | 1.49 | 17.60 | 54.68 |
| Cameroon | Western sub-Saharan Africa | both | 2.01 | 5.73 | 51.97 | 156.04 |
| Canada | High-income North America | both | 1.03 | 0.46 | 27.78 | 20.20 |
| Cabo Verde | Western sub-Saharan Africa | both | 0.39 | 5.63 | 18.91 | 187.02 |
| Central African Republic | Central sub-Saharan Africa | both | 1.09 | 1.68 | 33.35 | 56.00 |
| Chad | Western sub-Saharan Africa | both | 0.43 | 1.17 | 12.84 | 36.29 |
| Chile | Southern Latin America | both | 1.82 | 2.72 | 67.85 | 124.48 |
| China | East Asia | both | 0.65 | 1.74 | 32.64 | 89.70 |
| Colombia | Central Latin America | both | 1.91 | 1.96 | 90.68 | 133.83 |
| Comoros | Eastern Sub-Saharan Africa | both | 0.51 | 1.24 | 13.04 | 33.78 |
| Congo | Central sub-Saharan Africa | both | 2.49 | 6.46 | 71.80 | 194.89 |
| Costa Rica | Central Latin America | both | 1.16 | 1.61 | 61.69 | 127.47 |
| Côte d'Ivoire | Western sub-Saharan Africa | both | 1.22 | 2.96 | 33.41 | 87.34 |
| Croatia | Central Europe | both | 1.92 | 1.75 | 91.67 | 108.11 |
| Cuba | Caribbean | both | 2.90 | 1.46 | 134.63 | 127.76 |
| Cyprus | Western Europe | both | 9.44 | 3.86 | 189.20 | 113.96 |
| Czechia | Central Europe | both | 2.23 | 2.15 | 122.69 | 151.50 |
| Democratic Republic of the Congo | Central sub-Saharan Africa | both | 1.11 | 1.54 | 31.07 | 52.09 |
| Denmark | Western Europe | both | 1.38 | 0.94 | 44.87 | 35.94 |
| Djibouti | Eastern Sub-Saharan Africa | both | 1.46 | 6.77 | 39.80 | 185.43 |
| Dominica | Caribbean | both | 5.11 | 8.78 | 148.59 | 311.90 |
| Dominican Republic | Caribbean | both | 0.84 | 3.84 | 27.87 | 139.81 |
| Ecuador | Andean Latin America | both | 2.01 | 5.38 | 68.82 | 180.64 |
| Egypt | North Africa and Middle East | both | 4.83 | 8.12 | 148.38 | 293.51 |
| El Salvador | Central Latin America | both | 0.86 | 5.19 | 38.04 | 214.04 |
| Equatorial Guinea | Central sub-Saharan Africa | both | 0.93 | 10.44 | 28.65 | 294.69 |
| Eritrea | Eastern Sub-Saharan Africa | both | 0.92 | 2.91 | 27.07 | 83.41 |
| Estonia | Eastern Europe | both | 0.23 | 0.17 | 20.55 | 16.03 |
| Ethiopia | Eastern Sub-Saharan Africa | both | 0.63 | 1.29 | 17.31 | 34.01 |
| Micronesia (Federated States of) | Oceania | both | 2.14 | 9.03 | 61.88 | 262.32 |
| Fiji | Oceania | both | 4.04 | 18.21 | 111.26 | 486.81 |
| Finland | Western Europe | both | 0.45 | 0.09 | 26.43 | 13.61 |
| France | Western Europe | both | 1.20 | 0.75 | 34.74 | 29.01 |
| Gabon | Central sub-Saharan Africa | both | 4.03 | 13.16 | 113.59 | 379.37 |
| Georgia | Central Asia | both | 0.92 | 2.46 | 45.01 | 121.40 |
| Germany | Western Europe | both | 2.57 | 1.05 | 92.89 | 65.19 |
| Ghana | Western sub-Saharan Africa | both | 1.55 | 5.66 | 44.51 | 169.55 |
| Greece | Western Europe | both | 1.20 | 0.66 | 54.50 | 59.45 |
| Greenland | High-income North America | both | 0.69 | 0.38 | 19.65 | 19.41 |
| Grenada | Caribbean | both | 5.23 | 11.78 | 157.48 | 379.29 |
| Guam | Oceania | both | 2.75 | 1.58 | 83.64 | 78.77 |
| Guatemala | Central Latin America | both | 0.67 | 5.57 | 30.61 | 201.32 |
| Guinea | Western sub-Saharan Africa | both | 0.61 | 1.57 | 16.60 | 45.68 |
| Guinea-Bissau | Western sub-Saharan Africa | both | 1.02 | 2.32 | 28.59 | 67.23 |
| Guyana | Caribbean | both | 7.65 | 13.54 | 241.21 | 449.35 |
| Haiti | Caribbean | both | 1.14 | 1.91 | 34.63 | 68.17 |
| Honduras | Central Latin America | both | 0.37 | 1.29 | 25.12 | 80.53 |
| Hungary | Central Europe | both | 1.64 | 1.60 | 82.81 | 102.34 |
| Iceland | Western Europe | both | 0.24 | 0.12 | 12.04 | 11.05 |
| India | South Asia | both | 1.34 | 4.25 | 45.50 | 165.86 |
| Indonesia | Southeast Asia | both | 1.82 | 5.43 | 58.29 | 168.79 |
| Iran (Islamic Republic of) | North Africa and Middle East | both | 2.52 | 4.90 | 100.94 | 212.37 |
| Iraq | North Africa and Middle East | both | 8.25 | 10.48 | 257.22 | 369.95 |
| Ireland | Western Europe | both | 1.11 | 0.38 | 30.70 | 23.75 |
| Israel | Western Europe | both | 2.99 | 3.47 | 89.78 | 112.98 |
| Italy | Western Europe | both | 3.27 | 1.61 | 99.92 | 79.15 |
| Jamaica | Caribbean | both | 3.12 | 9.24 | 88.41 | 275.56 |
| Japan | High-income Asia Pacific | both | 0.69 | 0.24 | 37.48 | 38.65 |
| Jordan | North Africa and Middle East | both | 12.81 | 8.55 | 324.76 | 273.75 |
| Kazakhstan | Central Asia | both | 0.65 | 2.27 | 45.03 | 125.35 |
| Kenya | Eastern Sub-Saharan Africa | both | 0.66 | 1.81 | 17.92 | 52.69 |
| Kiribati | Oceania | both | 2.07 | 4.59 | 63.06 | 139.36 |
| Kuwait | North Africa and Middle East | both | 6.78 | 4.43 | 251.87 | 270.94 |
| Kyrgyzstan | Central Asia | both | 0.41 | 0.81 | 22.86 | 54.88 |
| Lao People's Democratic Republic | Southeast Asia | both | 0.69 | 1.60 | 22.52 | 58.48 |
| Latvia | Eastern Europe | both | 0.66 | 0.82 | 45.23 | 52.07 |
| Lebanon | North Africa and Middle East | both | 3.29 | 3.32 | 128.61 | 205.72 |
| Lesotho | Southern sub-Saharan Africa | both | 1.81 | 8.59 | 47.75 | 219.80 |
| Liberia | Western sub-Saharan Africa | both | 0.80 | 1.80 | 23.82 | 58.92 |
| Libya | North Africa and Middle East | both | 2.19 | 4.07 | 106.01 | 251.70 |
| Lithuania | Eastern Europe | both | 0.47 | 0.34 | 36.42 | 31.37 |
| Luxembourg | Western Europe | both | 1.44 | 0.52 | 42.09 | 50.00 |
| North Macedonia | Central Europe | both | 2.88 | 6.08 | 113.79 | 227.88 |
| Madagascar | Eastern Sub-Saharan Africa | both | 0.36 | 0.76 | 10.07 | 22.79 |
| Malawi | Eastern Sub-Saharan Africa | both | 0.50 | 0.98 | 13.52 | 29.96 |
| Malaysia | Southeast Asia | both | 4.55 | 1.94 | 163.02 | 119.19 |
| Maldives | Southeast Asia | both | 1.03 | 1.47 | 30.97 | 60.33 |
| Mali | Western sub-Saharan Africa | both | 0.56 | 1.27 | 15.59 | 37.45 |
| Malta | Western Europe | both | 3.27 | 1.43 | 97.60 | 75.97 |
| Marshall Islands | Oceania | both | 1.53 | 4.66 | 52.23 | 173.72 |
| Mauritania | Western sub-Saharan Africa | both | 1.87 | 4.42 | 47.89 | 114.35 |
| Mauritius | Southeast Asia | both | 4.44 | 14.28 | 155.40 | 430.04 |
| Mexico | Central Latin America | both | 8.62 | 9.18 | 286.77 | 323.11 |
| Republic of Moldova | Eastern Europe | both | 0.61 | 0.54 | 41.02 | 55.82 |
| Mongolia | Central Asia | both | 0.26 | 0.76 | 13.00 | 46.75 |
| Montenegro | Central Europe | both | 1.78 | 2.08 | 92.84 | 128.45 |
| Morocco | North Africa and Middle East | both | 1.11 | 4.63 | 46.52 | 208.26 |
| Mozambique | Eastern Sub-Saharan Africa | both | 0.29 | 0.89 | 7.88 | 26.00 |
| Myanmar | Southeast Asia | both | 1.59 | 3.95 | 47.39 | 124.47 |
| Namibia | Southern sub-Saharan Africa | both | 2.62 | 6.76 | 69.05 | 179.05 |
| Nepal | South Asia | both | 0.37 | 2.13 | 15.68 | 89.43 |
| Netherlands | Western Europe | both | 2.73 | 0.96 | 81.43 | 44.79 |
| New Zealand | Australasia | both | 0.49 | 0.28 | 17.11 | 13.63 |
| Nicaragua | Central Latin America | both | 0.72 | 3.54 | 28.26 | 125.68 |
| Niger | Western sub-Saharan Africa | both | 0.50 | 0.96 | 12.56 | 26.37 |
| Nigeria | Western sub-Saharan Africa | both | 1.33 | 4.18 | 33.83 | 104.56 |
| Democratic People's Republic of Korea | East Asia | both | 0.65 | 1.21 | 25.64 | 60.23 |
| Northern Mariana Islands | Oceania | both | 3.49 | 3.75 | 109.72 | 132.29 |
| Norway | Western Europe | both | 0.66 | 0.23 | 36.19 | 18.14 |
| Oman | North Africa and Middle East | both | 5.32 | 13.41 | 149.77 | 371.16 |
| Pakistan | South Asia | both | 0.99 | 6.35 | 33.96 | 198.63 |
| Palestine | North Africa and Middle East | both | 5.43 | 14.43 | 141.02 | 398.78 |
| Panama | Central Latin America | both | 1.32 | 3.06 | 58.12 | 136.01 |
| Papua New Guinea | Oceania | both | 1.23 | 2.56 | 36.55 | 79.05 |
| Paraguay | Tropical Latin America | both | 0.88 | 3.33 | 29.00 | 102.27 |
| Peru | Andean Latin America | both | 1.25 | 2.39 | 45.24 | 101.38 |
| Philippines | Southeast Asia | both | 2.78 | 3.51 | 77.17 | 113.08 |
| Poland | Central Europe | both | 1.91 | 1.79 | 100.46 | 121.60 |
| Portugal | Western Europe | both | 1.83 | 0.96 | 60.09 | 45.00 |
| Puerto Rico | Caribbean | both | 2.43 | 1.90 | 80.64 | 78.95 |
| Qatar | North Africa and Middle East | both | 26.96 | 29.68 | 612.07 | 722.09 |
| Romania | Central Europe | both | 0.75 | 0.79 | 44.67 | 63.70 |
| Russian Federation | Eastern Europe | both | 0.58 | 0.74 | 36.93 | 38.81 |
| Rwanda | Eastern Sub-Saharan Africa | both | 1.17 | 1.84 | 31.32 | 50.00 |
| Saint Lucia | Caribbean | both | 6.90 | 9.97 | 209.13 | 369.05 |
| Saint Vincent and the Grenadines | Caribbean | both | 6.97 | 12.82 | 196.97 | 413.60 |
| Samoa | Oceania | both | 2.34 | 3.83 | 69.72 | 124.13 |
| Sao Tome and Principe | Central sub-Saharan Africa | both | 0.32 | 1.33 | 13.54 | 60.39 |
| Saudi Arabia | North Africa and Middle East | both | 3.05 | 4.64 | 109.49 | 251.53 |
| Senegal | Western sub-Saharan Africa | both | 1.08 | 2.68 | 34.15 | 87.09 |
| Serbia | Central Europe | both | 2.46 | 3.69 | 100.06 | 166.21 |
| Seychelles | Southeast Asia | both | 1.63 | 3.43 | 76.09 | 188.11 |
| Sierra Leone | Western sub-Saharan Africa | both | 0.61 | 1.46 | 15.06 | 38.90 |
| Singapore | High-income Asia Pacific | both | 2.93 | 0.39 | 128.25 | 76.38 |
| Slovakia | Central Europe | both | 2.40 | 1.33 | 104.40 | 91.09 |
| Slovenia | Central Europe | both | 1.74 | 1.05 | 83.39 | 77.75 |
| Solomon Islands | Oceania | both | 0.64 | 2.62 | 20.88 | 85.95 |
| Somalia | Eastern Sub-Saharan Africa | both | 0.29 | 0.38 | 8.05 | 11.32 |
| South Africa | Southern sub-Saharan Africa | both | 5.21 | 11.41 | 148.70 | 313.84 |
| Republic of Korea | High-income Asia Pacific | both | 3.13 | 3.01 | 118.16 | 121.62 |
| South Sudan | Eastern Sub-Saharan Africa | both | 0.85 | 1.47 | 22.84 | 41.67 |
| Spain | Western Europe | both | 1.96 | 0.68 | 69.03 | 46.76 |
| Sri Lanka | Southeast Asia | both | 1.40 | 6.26 | 48.87 | 206.71 |
| Sudan | North Africa and Middle East | both | 0.34 | 2.24 | 15.40 | 122.32 |
| Suriname | Caribbean | both | 3.67 | 6.59 | 139.42 | 290.84 |
| Eswatini | Southern sub-Saharan Africa | both | 3.17 | 12.41 | 79.98 | 310.07 |
| Sweden | Western Europe | both | 0.69 | 0.24 | 26.58 | 11.95 |
| Switzerland | Western Europe | both | 2.04 | 0.56 | 65.44 | 35.41 |
| Syrian Arab Republic | North Africa and Middle East | both | 3.39 | 3.38 | 133.65 | 180.74 |
| Taiwan (Province of China) | East Asia | both | 4.02 | 4.84 | 124.13 | 168.29 |
| Tajikistan | Central Asia | both | 0.72 | 4.51 | 32.20 | 175.05 |
| United Republic of Tanzania | Eastern Sub-Saharan Africa | both | 0.52 | 1.36 | 12.68 | 37.12 |
| Thailand | Southeast Asia | both | 2.35 | 3.07 | 78.37 | 137.31 |
| Bahamas | Caribbean | both | 6.26 | 5.10 | 199.96 | 206.07 |
| Gambia | Western sub-Saharan Africa | both | 0.69 | 2.38 | 19.37 | 68.31 |
| Timor-Leste | Southeast Asia | both | 0.26 | 0.94 | 8.42 | 36.83 |
| Togo | Western sub-Saharan Africa | both | 0.83 | 2.03 | 21.95 | 56.26 |
| Tonga | Oceania | both | 2.46 | 6.20 | 69.63 | 184.50 |
| Trinidad and Tobago | Caribbean | both | 19.31 | 16.77 | 573.62 | 529.64 |
| Tunisia | North Africa and Middle East | both | 1.75 | 3.12 | 90.86 | 197.98 |
| Turkey | North Africa and Middle East | both | 6.11 | 4.51 | 172.98 | 172.00 |
| Turkmenistan | Central Asia | both | 1.66 | 3.04 | 80.14 | 155.01 |
| Uganda | Eastern Sub-Saharan Africa | both | 0.59 | 1.83 | 16.07 | 54.36 |
| Ukraine | Eastern Europe | both | 0.57 | 0.40 | 45.13 | 43.51 |
| United Arab Emirates | North Africa and Middle East | both | 17.35 | 12.77 | 433.46 | 430.73 |
| United Kingdom | Western Europe | both | 1.18 | 0.37 | 54.03 | 51.31 |
| United States of America | High-income North America | both | 1.57 | 0.74 | 72.66 | 45.80 |
| Uruguay | Southern Latin America | both | 1.21 | 1.17 | 29.01 | 42.09 |
| Uzbekistan | Central Asia | both | 0.99 | 6.67 | 46.57 | 245.07 |
| Vanuatu | Oceania | both | 0.73 | 2.30 | 24.57 | 78.57 |
| Venezuela (Bolivarian Republic of) | Central Latin America | both | 4.94 | 6.64 | 177.21 | 253.75 |
| Viet nam | Southeast Asia | both | 1.01 | 3.54 | 27.91 | 109.38 |
| United States Virgin Islands | Caribbean | both | 2.99 | 3.22 | 94.01 | 122.65 |
| Yemen | North Africa and Middle East | both | 0.33 | 1.77 | 13.46 | 84.92 |
| Zambia | Eastern Sub-Saharan Africa | both | 0.98 | 2.71 | 26.24 | 76.85 |
| Zimbabwe | Southern sub-Saharan Africa | both | 1.23 | 2.61 | 36.95 | 81.24 |
| Monaco | Western Europe | both | 0.26 | 0.33 | 17.69 | 37.59 |
| San Marino | Western Europe | both | 0.77 | 0.51 | 31.56 | 36.16 |
| Saint Kitts and Nevis | Caribbean | both | 3.68 | 3.93 | 109.40 | 136.63 |
| Cook Islands | Oceania | both | 4.20 | 4.72 | 111.25 | 142.11 |
| Nauru | Oceania | both | 2.99 | 5.38 | 83.05 | 153.59 |
| Niue | Oceania | both | 2.56 | 5.76 | 77.79 | 185.55 |
| Palau | Oceania | both | 3.67 | 5.51 | 109.04 | 172.82 |
| Tokelau | Oceania | both | 3.69 | 4.21 | 109.21 | 130.86 |
| Tuvalu | Oceania | both | 1.10 | 3.73 | 32.37 | 114.00 |
| Afghanistan | North Africa and Middle East | male | 0.49 | 1.52 | 19.19 | 75.37 |
| Albania | Central Europe | male | 0.37 | 0.49 | 23.36 | 50.25 |
| Algeria | North Africa and Middle East | male | 2.48 | 3.11 | 100.10 | 184.03 |
| American Samoa | Oceania | male | 3.58 | 4.64 | 118.74 | 174.39 |
| Andorra | Western Europe | male | 1.23 | 0.58 | 43.77 | 34.56 |
| Angola | Central sub-Saharan Africa | male | 1.18 | 5.68 | 34.45 | 170.50 |
| Antigua and Barbuda | Caribbean | male | 8.51 | 8.24 | 251.96 | 280.55 |
| Argentina | Southern Latin America | male | 2.63 | 2.72 | 77.35 | 101.79 |
| Armenia | Central Asia | male | 2.30 | 5.59 | 88.50 | 212.71 |
| Australia | Australasia | male | 0.74 | 0.50 | 23.51 | 22.60 |
| Austria | Western Europe | male | 2.08 | 1.52 | 68.11 | 63.54 |
| Azerbaijan | Central Asia | male | 1.22 | 4.08 | 54.14 | 169.04 |
| Bahrain | North Africa and Middle East | male | 18.44 | 29.77 | 486.85 | 774.07 |
| Bangladesh | South Asia | male | 1.07 | 3.16 | 29.28 | 95.57 |
| Barbados | Caribbean | male | 9.67 | 9.71 | 275.68 | 308.14 |
| Belarus | Eastern Europe | male | 0.71 | 0.39 | 51.07 | 43.01 |
| Belgium | Western Europe | male | 1.60 | 0.81 | 66.29 | 58.43 |
| Belize | Caribbean | male | 2.38 | 7.22 | 73.46 | 237.75 |
| Benin | Western sub-Saharan Africa | male | 0.79 | 2.09 | 22.42 | 64.51 |
| Bermuda | Caribbean | male | 2.55 | 1.11 | 80.39 | 48.00 |
| Bhutan | South Asia | male | 0.47 | 3.46 | 15.06 | 111.03 |
| Bolivia (Plurinational State of) | Andean Latin America | male | 3.02 | 5.19 | 95.99 | 168.68 |
| Bosnia and Herzegovina | Central Europe | male | 1.16 | 6.21 | 58.60 | 235.58 |
| Botswana | Southern sub-Saharan Africa | male | 3.13 | 13.22 | 82.61 | 343.10 |
| Brazil | Tropical Latin America | male | 1.99 | 2.54 | 78.82 | 99.39 |
| Brunei Darussalam | High-income Asia Pacific | male | 8.01 | 5.76 | 206.11 | 168.72 |
| Bulgaria | Central Europe | male | 2.33 | 2.32 | 102.81 | 123.35 |
| Burkina Faso | Western sub-Saharan Africa | male | 0.89 | 1.86 | 22.82 | 52.43 |
| Burundi | Eastern Sub-Saharan Africa | male | 1.12 | 1.20 | 30.23 | 34.16 |
| Cambodia | Southeast Asia | male | 0.72 | 1.82 | 21.43 | 68.44 |
| Cameroon | Western sub-Saharan Africa | male | 2.53 | 6.61 | 64.72 | 181.43 |
| Canada | High-income North America | male | 1.17 | 0.58 | 31.92 | 24.06 |
| Cabo Verde | Western sub-Saharan Africa | male | 0.47 | 5.64 | 23.15 | 196.43 |
| Central African Republic | Central sub-Saharan Africa | male | 1.79 | 2.63 | 52.49 | 83.84 |
| Chad | Western sub-Saharan Africa | male | 0.51 | 1.23 | 15.33 | 39.21 |
| Chile | Southern Latin America | male | 2.06 | 3.04 | 75.42 | 134.89 |
| China | East Asia | male | 0.72 | 2.05 | 36.09 | 101.57 |
| Colombia | Central Latin America | male | 1.72 | 1.94 | 96.10 | 146.85 |
| Comoros | Eastern Sub-Saharan Africa | male | 0.66 | 1.59 | 16.73 | 43.40 |
| Congo | Central sub-Saharan Africa | male | 3.95 | 8.05 | 110.02 | 238.59 |
| Costa Rica | Central Latin America | male | 1.05 | 1.71 | 67.83 | 147.52 |
| Côte d'Ivoire | Western sub-Saharan Africa | male | 1.59 | 3.38 | 42.63 | 99.00 |
| Croatia | Central Europe | male | 1.97 | 2.00 | 101.90 | 124.87 |
| Cuba | Caribbean | male | 2.13 | 1.34 | 130.89 | 143.05 |
| Cyprus | Western Europe | male | 7.52 | 4.04 | 178.18 | 127.24 |
| Czechia | Central Europe | male | 2.43 | 2.54 | 139.14 | 177.77 |
| Democratic Republic of the Congo | Central sub-Saharan Africa | male | 1.70 | 2.53 | 46.95 | 78.87 |
| Denmark | Western Europe | male | 1.67 | 1.22 | 53.33 | 42.57 |
| Djibouti | Eastern Sub-Saharan Africa | male | 1.92 | 8.31 | 50.57 | 222.56 |
| Dominica | Caribbean | male | 4.95 | 8.05 | 157.21 | 329.21 |
| Dominican Republic | Caribbean | male | 0.88 | 4.20 | 31.01 | 157.09 |
| Ecuador | Andean Latin America | male | 1.83 | 5.29 | 68.61 | 182.93 |
| Egypt | North Africa and Middle East | male | 4.23 | 7.01 | 131.91 | 274.62 |
| El Salvador | Central Latin America | male | 0.91 | 5.27 | 44.83 | 239.40 |
| Equatorial Guinea | Central sub-Saharan Africa | male | 1.58 | 14.47 | 46.21 | 393.70 |
| Eritrea | Eastern Sub-Saharan Africa | male | 1.35 | 3.80 | 38.59 | 108.80 |
| Estonia | Eastern Europe | male | 0.28 | 0.19 | 23.43 | 17.46 |
| Ethiopia | Eastern Sub-Saharan Africa | male | 0.85 | 1.71 | 22.92 | 43.40 |
| Micronesia (Federated States of) | Oceania | male | 2.46 | 9.93 | 71.93 | 295.67 |
| Fiji | Oceania | male | 4.99 | 21.52 | 134.09 | 552.45 |
| Finland | Western Europe | male | 0.42 | 0.12 | 27.32 | 14.54 |
| France | Western Europe | male | 1.33 | 0.95 | 40.31 | 34.73 |
| Gabon | Central sub-Saharan Africa | male | 7.11 | 19.29 | 188.00 | 529.17 |
| Georgia | Central Asia | male | 1.20 | 3.12 | 54.14 | 147.59 |
| Germany | Western Europe | male | 2.40 | 1.20 | 94.68 | 73.34 |
| Ghana | Western sub-Saharan Africa | male | 1.71 | 8.12 | 48.36 | 230.09 |
| Greece | Western Europe | male | 1.06 | 0.74 | 56.28 | 68.92 |
| Greenland | High-income North America | male | 0.73 | 0.50 | 23.18 | 24.95 |
| Grenada | Caribbean | male | 5.44 | 11.47 | 166.45 | 392.05 |
| Guam | Oceania | male | 2.07 | 1.55 | 71.50 | 80.27 |
| Guatemala | Central Latin America | male | 0.69 | 5.82 | 34.31 | 217.53 |
| Guinea | Western sub-Saharan Africa | male | 0.64 | 1.62 | 17.82 | 48.93 |
| Guinea-Bissau | Western sub-Saharan Africa | male | 1.37 | 2.66 | 37.94 | 78.08 |
| Guyana | Caribbean | male | 7.40 | 13.15 | 239.68 | 450.72 |
| Haiti | Caribbean | male | 0.87 | 1.42 | 29.04 | 59.37 |
| Honduras | Central Latin America | male | 0.44 | 1.46 | 31.46 | 96.46 |
| Hungary | Central Europe | male | 1.57 | 1.94 | 90.33 | 124.54 |
| Iceland | Western Europe | male | 0.28 | 0.18 | 13.53 | 13.09 |
| India | South Asia | male | 1.56 | 4.80 | 53.42 | 188.36 |
| Indonesia | Southeast Asia | male | 2.05 | 6.05 | 65.05 | 188.29 |
| Iran (Islamic Republic of) | North Africa and Middle East | male | 2.23 | 4.43 | 98.93 | 202.84 |
| Iraq | North Africa and Middle East | male | 8.21 | 10.99 | 258.97 | 377.48 |
| Ireland | Western Europe | male | 1.26 | 0.48 | 34.89 | 27.79 |
| Israel | Western Europe | male | 2.92 | 3.83 | 89.83 | 124.46 |
| Italy | Western Europe | male | 2.99 | 1.87 | 100.61 | 90.85 |
| Jamaica | Caribbean | male | 2.94 | 8.52 | 85.36 | 263.41 |
| Japan | High-income Asia Pacific | male | 0.79 | 0.31 | 46.80 | 48.26 |
| Jordan | North Africa and Middle East | male | 9.36 | 8.22 | 268.73 | 285.71 |
| Kazakhstan | Central Asia | male | 0.61 | 2.16 | 40.73 | 112.82 |
| Kenya | Eastern Sub-Saharan Africa | male | 0.91 | 2.75 | 24.24 | 76.35 |
| Kiribati | Oceania | male | 2.46 | 6.35 | 75.43 | 191.87 |
| Kuwait | North Africa and Middle East | male | 5.80 | 4.69 | 241.81 | 289.98 |
| Kyrgyzstan | Central Asia | male | 0.47 | 0.87 | 26.22 | 58.21 |
| Lao People's Democratic Republic | Southeast Asia | male | 0.76 | 1.69 | 25.20 | 64.72 |
| Latvia | Eastern Europe | male | 0.64 | 0.84 | 46.52 | 55.77 |
| Lebanon | North Africa and Middle East | male | 3.91 | 4.42 | 144.41 | 238.88 |
| Lesotho | Southern sub-Saharan Africa | male | 2.40 | 10.38 | 62.37 | 269.74 |
| Liberia | Western sub-Saharan Africa | male | 0.91 | 1.85 | 27.44 | 63.77 |
| Libya | North Africa and Middle East | male | 2.13 | 3.76 | 109.82 | 248.60 |
| Lithuania | Eastern Europe | male | 0.51 | 0.41 | 40.39 | 36.52 |
| Luxembourg | Western Europe | male | 1.44 | 0.59 | 44.29 | 56.45 |
| North Macedonia | Central Europe | male | 2.54 | 5.78 | 115.19 | 236.37 |
| Madagascar | Eastern Sub-Saharan Africa | male | 0.46 | 0.96 | 12.53 | 28.61 |
| Malawi | Eastern Sub-Saharan Africa | male | 0.67 | 1.59 | 17.84 | 45.76 |
| Malaysia | Southeast Asia | male | 4.02 | 1.83 | 154.53 | 117.69 |
| Maldives | Southeast Asia | male | 1.01 | 1.67 | 31.44 | 67.30 |
| Mali | Western sub-Saharan Africa | male | 0.59 | 1.17 | 16.48 | 36.09 |
| Malta | Western Europe | male | 3.00 | 1.68 | 95.26 | 79.73 |
| Marshall Islands | Oceania | male | 1.72 | 4.12 | 59.64 | 170.46 |
| Mauritania | Western sub-Saharan Africa | male | 2.09 | 3.83 | 52.87 | 99.89 |
| Mauritius | Southeast Asia | male | 4.91 | 16.17 | 176.20 | 488.83 |
| Mexico | Central Latin America | male | 8.06 | 10.19 | 285.74 | 365.71 |
| Republic of Moldova | Eastern Europe | male | 0.67 | 0.59 | 44.94 | 59.12 |
| Mongolia | Central Asia | male | 0.36 | 1.10 | 16.33 | 58.14 |
| Montenegro | Central Europe | male | 1.87 | 2.26 | 103.45 | 142.19 |
| Morocco | North Africa and Middle East | male | 1.09 | 4.09 | 48.73 | 196.00 |
| Mozambique | Eastern Sub-Saharan Africa | male | 0.41 | 1.48 | 10.79 | 41.14 |
| Myanmar | Southeast Asia | male | 1.92 | 5.30 | 55.18 | 158.74 |
| Namibia | Southern sub-Saharan Africa | male | 3.11 | 8.53 | 81.27 | 222.06 |
| Nepal | South Asia | male | 0.42 | 2.21 | 19.92 | 111.52 |
| Netherlands | Western Europe | male | 2.64 | 1.06 | 86.79 | 51.90 |
| New Zealand | Australasia | male | 0.57 | 0.34 | 18.78 | 15.54 |
| Nicaragua | Central Latin America | male | 0.96 | 4.18 | 35.50 | 150.70 |
| Niger | Western sub-Saharan Africa | male | 0.60 | 0.99 | 14.76 | 27.83 |
| Nigeria | Western sub-Saharan Africa | male | 1.73 | 4.94 | 42.02 | 120.43 |
| Democratic People's Republic of Korea | East Asia | male | 0.75 | 1.37 | 30.30 | 69.45 |
| Northern Mariana Islands | Oceania | male | 3.27 | 4.24 | 107.74 | 147.08 |
| Norway | Western Europe | male | 0.77 | 0.28 | 41.66 | 20.27 |
| Oman | North Africa and Middle East | male | 5.54 | 14.78 | 155.47 | 393.45 |
| Pakistan | South Asia | male | 1.16 | 6.24 | 40.50 | 205.41 |
| Palestine | North Africa and Middle East | male | 5.77 | 15.04 | 155.31 | 426.44 |
| Panama | Central Latin America | male | 1.21 | 3.09 | 62.41 | 146.54 |
| Papua New Guinea | Oceania | male | 1.89 | 3.68 | 53.64 | 108.70 |
| Paraguay | Tropical Latin America | male | 0.88 | 3.55 | 30.39 | 114.90 |
| Peru | Andean Latin America | male | 1.39 | 2.51 | 51.25 | 108.43 |
| Philippines | Southeast Asia | male | 3.09 | 3.95 | 92.85 | 128.41 |
| Poland | Central Europe | male | 1.85 | 2.12 | 105.97 | 143.55 |
| Portugal | Western Europe | male | 1.86 | 1.06 | 63.23 | 48.72 |
| Puerto Rico | Caribbean | male | 2.35 | 2.18 | 83.37 | 88.72 |
| Qatar | North Africa and Middle East | male | 27.34 | 26.10 | 616.72 | 672.11 |
| Romania | Central Europe | male | 0.83 | 0.93 | 51.55 | 73.61 |
| Russian Federation | Eastern Europe | male | 0.49 | 0.61 | 35.07 | 37.12 |
| Rwanda | Eastern Sub-Saharan Africa | male | 1.65 | 2.70 | 43.24 | 70.87 |
| Saint Lucia | Caribbean | male | 6.84 | 10.15 | 206.50 | 365.54 |
| Saint Vincent and the Grenadines | Caribbean | male | 5.91 | 12.04 | 181.00 | 407.14 |
| Samoa | Oceania | male | 3.07 | 4.30 | 89.15 | 138.68 |
| Sao Tome and Principe | Central sub-Saharan Africa | male | 0.48 | 1.86 | 17.65 | 75.85 |
| Saudi Arabia | North Africa and Middle East | male | 3.26 | 4.95 | 118.32 | 262.73 |
| Senegal | Western sub-Saharan Africa | male | 1.34 | 2.85 | 41.76 | 95.88 |
| Serbia | Central Europe | male | 2.34 | 3.82 | 107.86 | 187.08 |
| Seychelles | Southeast Asia | male | 1.67 | 3.26 | 83.76 | 192.33 |
| Sierra Leone | Western sub-Saharan Africa | male | 0.76 | 1.43 | 18.73 | 38.98 |
| Singapore | High-income Asia Pacific | male | 2.58 | 0.43 | 129.33 | 90.43 |
| Slovakia | Central Europe | male | 2.49 | 1.45 | 114.26 | 101.18 |
| Slovenia | Central Europe | male | 1.68 | 1.35 | 89.73 | 90.92 |
| Solomon Islands | Oceania | male | 0.72 | 2.99 | 23.78 | 100.86 |
| Somalia | Eastern Sub-Saharan Africa | male | 0.40 | 0.55 | 10.95 | 15.85 |
| South Africa | Southern sub-Saharan Africa | male | 5.56 | 12.12 | 159.87 | 330.24 |
| Republic of Korea | High-income Asia Pacific | male | 3.99 | 3.68 | 143.82 | 143.03 |
| South Sudan | Eastern Sub-Saharan Africa | male | 1.09 | 1.89 | 28.44 | 53.10 |
| Spain | Western Europe | male | 1.75 | 0.74 | 69.86 | 52.71 |
| Sri Lanka | Southeast Asia | male | 1.82 | 7.43 | 61.39 | 242.07 |
| Sudan | North Africa and Middle East | male | 0.36 | 2.30 | 17.28 | 131.87 |
| Suriname | Caribbean | male | 3.89 | 7.35 | 152.89 | 320.77 |
| Eswatini | Southern sub-Saharan Africa | male | 4.21 | 17.80 | 104.18 | 439.49 |
| Sweden | Western Europe | male | 0.80 | 0.29 | 30.61 | 13.98 |
| Switzerland | Western Europe | male | 2.09 | 0.67 | 70.74 | 40.21 |
| Syrian Arab Republic | North Africa and Middle East | male | 2.79 | 2.92 | 124.11 | 174.02 |
| Taiwan (Province of China) | East Asia | male | 3.62 | 5.31 | 119.44 | 190.19 |
| Tajikistan | Central Asia | male | 0.89 | 5.09 | 38.65 | 192.58 |
| United Republic of Tanzania | Eastern Sub-Saharan Africa | male | 0.68 | 1.85 | 16.50 | 49.11 |
| Thailand | Southeast Asia | male | 2.35 | 3.03 | 84.16 | 146.72 |
| Bahamas | Caribbean | male | 5.78 | 5.20 | 195.43 | 216.62 |
| Gambia | Western sub-Saharan Africa | male | 0.87 | 2.68 | 23.91 | 77.49 |
| Timor-Leste | Southeast Asia | male | 0.24 | 0.93 | 8.52 | 40.77 |
| Togo | Western sub-Saharan Africa | male | 1.02 | 2.51 | 26.89 | 69.96 |
| Tonga | Oceania | male | 2.25 | 6.99 | 65.02 | 206.55 |
| Trinidad and Tobago | Caribbean | male | 19.09 | 18.47 | 571.77 | 580.40 |
| Tunisia | North Africa and Middle East | male | 1.74 | 3.29 | 100.03 | 218.50 |
| Turkey | North Africa and Middle East | male | 5.32 | 4.13 | 162.50 | 170.30 |
| Turkmenistan | Central Asia | male | 1.78 | 3.17 | 82.24 | 155.84 |
| Uganda | Eastern Sub-Saharan Africa | male | 0.86 | 2.89 | 22.71 | 80.75 |
| Ukraine | Eastern Europe | male | 0.61 | 0.48 | 46.97 | 48.02 |
| United Arab Emirates | North Africa and Middle East | male | 15.45 | 13.21 | 406.86 | 441.28 |
| United Kingdom | Western Europe | male | 1.39 | 0.42 | 62.41 | 59.77 |
| United States of America | High-income North America | male | 1.65 | 0.89 | 79.22 | 53.79 |
| Uruguay | Southern Latin America | male | 1.34 | 1.41 | 32.27 | 51.38 |
| Uzbekistan | Central Asia | male | 1.21 | 7.21 | 56.52 | 265.90 |
| Vanuatu | Oceania | male | 0.80 | 2.41 | 28.02 | 87.04 |
| Venezuela (Bolivarian Republic of) | Central Latin America | male | 4.74 | 7.38 | 180.30 | 278.39 |
| Viet nam | Southeast Asia | male | 0.94 | 3.82 | 28.78 | 124.54 |
| United States Virgin Islands | Caribbean | male | 2.46 | 3.70 | 87.20 | 141.86 |
| Yemen | North Africa and Middle East | male | 0.35 | 1.63 | 15.08 | 85.11 |
| Zambia | Eastern Sub-Saharan Africa | male | 1.25 | 3.94 | 32.68 | 107.87 |
| Zimbabwe | Southern sub-Saharan Africa | male | 1.18 | 2.65 | 39.10 | 87.87 |
| Monaco | Western Europe | male | 0.34 | 0.47 | 20.39 | 42.92 |
| San Marino | Western Europe | male | 0.85 | 0.61 | 35.09 | 40.13 |
| Saint Kitts and Nevis | Caribbean | male | 3.53 | 4.31 | 108.95 | 152.13 |
| Cook Islands | Oceania | male | 4.37 | 4.88 | 115.55 | 150.76 |
| Nauru | Oceania | male | 3.10 | 5.40 | 89.86 | 161.46 |
| Niue | Oceania | male | 2.87 | 6.10 | 90.09 | 198.70 |
| Palau | Oceania | male | 3.45 | 5.52 | 107.01 | 179.39 |
| Tokelau | Oceania | male | 2.29 | 2.87 | 72.65 | 98.95 |
| Tuvalu | Oceania | male | 1.16 | 3.45 | 34.79 | 111.08 |
| Afghanistan | North Africa and Middle East | female | 0.72 | 3.06 | 23.92 | 108.40 |
| Albania | Central Europe | female | 0.25 | 0.38 | 15.79 | 38.13 |
| Algeria | North Africa and Middle East | female | 3.17 | 4.88 | 109.21 | 221.81 |
| American Samoa | Oceania | female | 2.24 | 3.88 | 74.47 | 146.12 |
| Andorra | Western Europe | female | 0.63 | 0.30 | 27.69 | 25.15 |
| Angola | Central sub-Saharan Africa | female | 0.41 | 2.30 | 13.01 | 74.43 |
| Antigua and Barbuda | Caribbean | female | 7.11 | 9.11 | 215.55 | 285.73 |
| Argentina | Southern Latin America | female | 1.90 | 1.76 | 59.03 | 74.90 |
| Armenia | Central Asia | female | 2.62 | 5.94 | 97.71 | 217.70 |
| Australia | Australasia | female | 0.53 | 0.32 | 17.67 | 16.08 |
| Austria | Western Europe | female | 1.98 | 0.99 | 59.69 | 46.43 |
| Azerbaijan | Central Asia | female | 0.93 | 3.97 | 44.45 | 169.21 |
| Bahrain | North Africa and Middle East | female | 15.88 | 30.89 | 420.94 | 761.95 |
| Bangladesh | South Asia | female | 0.65 | 3.12 | 18.81 | 85.36 |
| Barbados | Caribbean | female | 12.56 | 10.96 | 338.01 | 325.20 |
| Belarus | Eastern Europe | female | 0.73 | 0.29 | 51.28 | 38.92 |
| Belgium | Western Europe | female | 1.98 | 0.63 | 66.54 | 47.86 |
| Belize | Caribbean | female | 3.20 | 7.81 | 90.33 | 252.34 |
| Benin | Western sub-Saharan Africa | female | 0.45 | 1.50 | 13.21 | 45.74 |
| Bermuda | Caribbean | female | 2.06 | 0.67 | 59.43 | 33.60 |
| Bhutan | South Asia | female | 0.34 | 2.84 | 11.41 | 88.32 |
| Bolivia (Plurinational State of) | Andean Latin America | female | 3.85 | 6.39 | 107.19 | 175.85 |
| Bosnia and Herzegovina | Central Europe | female | 1.33 | 5.80 | 50.70 | 189.52 |
| Botswana | Southern sub-Saharan Africa | female | 2.20 | 10.27 | 56.40 | 268.42 |
| Brazil | Tropical Latin America | female | 2.25 | 2.29 | 73.64 | 82.68 |
| Brunei Darussalam | High-income Asia Pacific | female | 5.26 | 3.54 | 143.65 | 113.84 |
| Bulgaria | Central Europe | female | 2.18 | 1.84 | 88.31 | 96.42 |
| Burkina Faso | Western sub-Saharan Africa | female | 0.40 | 0.81 | 11.60 | 26.48 |
| Burundi | Eastern Sub-Saharan Africa | female | 0.54 | 0.63 | 14.90 | 18.21 |
| Cambodia | Southeast Asia | female | 0.51 | 1.27 | 14.76 | 44.67 |
| Cameroon | Western sub-Saharan Africa | female | 1.55 | 4.97 | 39.99 | 132.45 |
| Canada | High-income North America | female | 0.93 | 0.37 | 24.46 | 16.75 |
| Cabo Verde | Western sub-Saharan Africa | female | 0.33 | 5.49 | 15.94 | 177.44 |
| Central African Republic | Central sub-Saharan Africa | female | 0.55 | 1.01 | 17.40 | 33.60 |
| Chad | Western sub-Saharan Africa | female | 0.36 | 1.10 | 10.54 | 32.93 |
| Chile | Southern Latin America | female | 1.63 | 2.45 | 61.43 | 115.50 |
| China | East Asia | female | 0.63 | 1.55 | 29.61 | 79.05 |
| Colombia | Central Latin America | female | 2.07 | 1.97 | 85.49 | 122.73 |
| Comoros | Eastern Sub-Saharan Africa | female | 0.37 | 0.96 | 9.64 | 25.79 |
| Congo | Central sub-Saharan Africa | female | 1.45 | 5.17 | 41.96 | 155.64 |
| Costa Rica | Central Latin America | female | 1.25 | 1.51 | 55.86 | 109.96 |
| Côte d'Ivoire | Western sub-Saharan Africa | female | 0.83 | 2.54 | 22.93 | 74.74 |
| Croatia | Central Europe | female | 1.84 | 1.54 | 82.88 | 93.36 |
| Cuba | Caribbean | female | 3.62 | 1.56 | 138.19 | 113.70 |
| Cyprus | Western Europe | female | 11.33 | 3.59 | 199.88 | 100.75 |
| Czechia | Central Europe | female | 2.06 | 1.83 | 109.46 | 127.82 |
| Democratic Republic of the Congo | Central sub-Saharan Africa | female | 0.56 | 0.87 | 16.99 | 31.05 |
| Denmark | Western Europe | female | 1.14 | 0.71 | 37.41 | 29.82 |
| Djibouti | Eastern Sub-Saharan Africa | female | 1.08 | 5.21 | 29.37 | 143.38 |
| Dominica | Caribbean | female | 5.17 | 9.30 | 142.03 | 292.08 |
| Dominican Republic | Caribbean | female | 0.81 | 3.46 | 24.83 | 122.65 |
| Ecuador | Andean Latin America | female | 2.20 | 5.43 | 69.09 | 178.10 |
| Egypt | North Africa and Middle East | female | 5.43 | 10.10 | 164.71 | 322.28 |
| El Salvador | Central Latin America | female | 0.81 | 5.13 | 32.11 | 195.20 |
| Equatorial Guinea | Central sub-Saharan Africa | female | 0.47 | 7.80 | 15.02 | 225.03 |
| Eritrea | Eastern Sub-Saharan Africa | female | 0.65 | 2.36 | 19.06 | 65.12 |
| Estonia | Eastern Europe | female | 0.21 | 0.15 | 18.48 | 14.85 |
| Ethiopia | Eastern Sub-Saharan Africa | female | 0.40 | 0.87 | 11.41 | 24.12 |
| Micronesia (Federated States of) | Oceania | female | 1.85 | 8.21 | 51.93 | 230.54 |
| Fiji | Oceania | female | 3.23 | 16.08 | 89.62 | 434.59 |
| Finland | Western Europe | female | 0.45 | 0.07 | 24.98 | 12.70 |
| France | Western Europe | female | 1.10 | 0.59 | 30.08 | 24.09 |
| Gabon | Central sub-Saharan Africa | female | 1.98 | 8.68 | 55.81 | 251.14 |
| Georgia | Central Asia | female | 0.75 | 2.00 | 38.55 | 100.86 |
| Germany | Western Europe | female | 2.60 | 0.91 | 89.28 | 57.42 |
| Ghana | Western sub-Saharan Africa | female | 1.42 | 3.89 | 41.17 | 122.66 |
| Greece | Western Europe | female | 1.30 | 0.58 | 52.56 | 50.85 |
| Greenland | High-income North America | female | 0.65 | 0.25 | 15.84 | 13.03 |
| Grenada | Caribbean | female | 5.06 | 11.67 | 150.74 | 364.19 |
| Guam | Oceania | female | 3.34 | 1.61 | 95.33 | 77.28 |
| Guatemala | Central Latin America | female | 0.65 | 5.42 | 27.01 | 188.60 |
| Guinea | Western sub-Saharan Africa | female | 0.58 | 1.52 | 15.39 | 42.43 |
| Guinea-Bissau | Western sub-Saharan Africa | female | 0.69 | 2.05 | 19.87 | 58.21 |
| Guyana | Caribbean | female | 7.85 | 13.78 | 242.35 | 447.41 |
| Haiti | Caribbean | female | 1.38 | 2.34 | 39.94 | 76.00 |
| Honduras | Central Latin America | female | 0.31 | 1.13 | 19.04 | 66.38 |
| Hungary | Central Europe | female | 1.66 | 1.35 | 76.27 | 84.20 |
| Iceland | Western Europe | female | 0.21 | 0.06 | 10.70 | 9.14 |
| India | South Asia | female | 1.12 | 3.77 | 37.04 | 144.25 |
| Indonesia | Southeast Asia | female | 1.63 | 4.90 | 52.29 | 151.13 |
| Iran (Islamic Republic of) | North Africa and Middle East | female | 2.78 | 5.39 | 102.68 | 221.95 |
| Iraq | North Africa and Middle East | female | 8.28 | 9.95 | 255.74 | 362.54 |
| Ireland | Western Europe | female | 0.99 | 0.30 | 27.08 | 20.11 |
| Israel | Western Europe | female | 3.05 | 3.13 | 89.52 | 102.27 |
| Italy | Western Europe | female | 3.38 | 1.39 | 97.70 | 68.58 |
| Jamaica | Caribbean | female | 3.28 | 9.83 | 91.28 | 286.81 |
| Japan | High-income Asia Pacific | female | 0.62 | 0.17 | 29.41 | 29.60 |
| Jordan | North Africa and Middle East | female | 16.22 | 8.99 | 380.52 | 259.96 |
| Kazakhstan | Central Asia | female | 0.67 | 2.30 | 47.59 | 133.71 |
| Kenya | Eastern Sub-Saharan Africa | female | 0.44 | 1.11 | 11.89 | 32.29 |
| Kiribati | Oceania | female | 1.77 | 3.36 | 52.66 | 98.73 |
| Kuwait | North Africa and Middle East | female | 7.91 | 4.00 | 265.58 | 243.16 |
| Kyrgyzstan | Central Asia | female | 0.36 | 0.76 | 20.23 | 52.08 |
| Lao People's Democratic Republic | Southeast Asia | female | 0.64 | 1.52 | 20.24 | 52.69 |
| Latvia | Eastern Europe | female | 0.67 | 0.77 | 43.78 | 48.72 |
| Lebanon | North Africa and Middle East | female | 2.72 | 2.41 | 113.33 | 178.42 |
| Lesotho | Southern sub-Saharan Africa | female | 1.42 | 7.41 | 36.89 | 184.09 |
| Liberia | Western sub-Saharan Africa | female | 0.67 | 1.75 | 19.65 | 53.86 |
| Libya | North Africa and Middle East | female | 2.27 | 4.37 | 101.84 | 254.88 |
| Lithuania | Eastern Europe | female | 0.44 | 0.30 | 33.29 | 27.42 |
| Luxembourg | Western Europe | female | 1.41 | 0.46 | 39.83 | 43.82 |
| North Macedonia | Central Europe | female | 3.16 | 6.25 | 112.11 | 217.89 |
| Madagascar | Eastern Sub-Saharan Africa | female | 0.26 | 0.58 | 7.53 | 17.48 |
| Malawi | Eastern Sub-Saharan Africa | female | 0.35 | 0.58 | 9.76 | 17.78 |
| Malaysia | Southeast Asia | female | 5.03 | 2.06 | 170.66 | 120.53 |
| Maldives | Southeast Asia | female | 1.03 | 1.23 | 30.03 | 51.94 |
| Mali | Western sub-Saharan Africa | female | 0.53 | 1.37 | 14.76 | 38.90 |
| Malta | Western Europe | female | 3.45 | 1.24 | 98.93 | 72.52 |
| Marshall Islands | Oceania | female | 1.38 | 5.25 | 45.38 | 177.22 |
| Mauritania | Western sub-Saharan Africa | female | 1.70 | 5.03 | 43.58 | 129.08 |
| Mauritius | Southeast Asia | female | 4.01 | 12.60 | 136.31 | 375.36 |
| Mexico | Central Latin America | female | 9.16 | 8.28 | 287.64 | 285.12 |
| Republic of Moldova | Eastern Europe | female | 0.57 | 0.50 | 38.06 | 53.18 |
| Mongolia | Central Asia | female | 0.18 | 0.51 | 10.15 | 37.51 |
| Montenegro | Central Europe | female | 1.71 | 1.90 | 83.72 | 115.83 |
| Morocco | North Africa and Middle East | female | 1.13 | 5.17 | 44.44 | 220.41 |
| Mozambique | Eastern Sub-Saharan Africa | female | 0.20 | 0.49 | 5.33 | 14.27 |
| Myanmar | Southeast Asia | female | 1.32 | 2.98 | 40.74 | 97.79 |
| Namibia | Southern sub-Saharan Africa | female | 2.24 | 5.55 | 59.14 | 147.58 |
| Nepal | South Asia | female | 0.30 | 2.01 | 11.08 | 69.34 |
| Netherlands | Western Europe | female | 2.73 | 0.85 | 75.44 | 37.94 |
| New Zealand | Australasia | female | 0.43 | 0.22 | 15.85 | 11.93 |
| Nicaragua | Central Latin America | female | 0.55 | 3.03 | 22.06 | 105.05 |
| Niger | Western sub-Saharan Africa | female | 0.41 | 0.94 | 10.23 | 25.03 |
| Nigeria | Western sub-Saharan Africa | female | 1.01 | 3.48 | 25.66 | 90.03 |
| Democratic People's Republic of Korea | East Asia | female | 0.60 | 1.12 | 22.38 | 52.87 |
| Northern Mariana Islands | Oceania | female | 3.77 | 3.27 | 112.79 | 116.62 |
| Norway | Western Europe | female | 0.57 | 0.19 | 31.18 | 16.03 |
| Oman | North Africa and Middle East | female | 5.26 | 12.43 | 146.21 | 353.40 |
| Pakistan | South Asia | female | 0.77 | 6.47 | 26.06 | 191.46 |
| Palestine | North Africa and Middle East | female | 5.18 | 13.99 | 129.68 | 372.43 |
| Panama | Central Latin America | female | 1.41 | 3.01 | 53.67 | 125.66 |
| Papua New Guinea | Oceania | female | 0.57 | 1.38 | 18.51 | 46.34 |
| Paraguay | Tropical Latin America | female | 0.88 | 3.10 | 27.74 | 90.05 |
| Peru | Andean Latin America | female | 1.12 | 2.27 | 39.44 | 94.74 |
| Philippines | Southeast Asia | female | 2.49 | 3.09 | 61.97 | 98.38 |
| Poland | Central Europe | female | 1.90 | 1.50 | 94.58 | 101.85 |
| Portugal | Western Europe | female | 1.80 | 0.88 | 57.23 | 41.77 |
| Puerto Rico | Caribbean | female | 2.47 | 1.66 | 78.06 | 70.66 |
| Qatar | North Africa and Middle East | female | 27.12 | 42.14 | 622.68 | 880.83 |
| Romania | Central Europe | female | 0.68 | 0.67 | 38.71 | 54.91 |
| Russian Federation | Eastern Europe | female | 0.62 | 0.80 | 37.76 | 39.48 |
| Rwanda | Eastern Sub-Saharan Africa | female | 0.81 | 1.31 | 21.94 | 35.54 |
| Saint Lucia | Caribbean | female | 6.98 | 9.69 | 212.09 | 371.41 |
| Saint Vincent and the Grenadines | Caribbean | female | 7.75 | 13.54 | 209.62 | 419.45 |
| Samoa | Oceania | female | 1.70 | 3.42 | 51.29 | 110.03 |
| Sao Tome and Principe | Central sub-Saharan Africa | female | 0.21 | 0.86 | 10.02 | 46.24 |
| Saudi Arabia | North Africa and Middle East | female | 2.83 | 4.18 | 98.06 | 234.52 |
| Senegal | Western sub-Saharan Africa | female | 0.83 | 2.54 | 26.48 | 79.07 |
| Serbia | Central Europe | female | 2.51 | 3.51 | 92.14 | 146.19 |
| Seychelles | Southeast Asia | female | 1.58 | 3.46 | 69.68 | 182.67 |
| Sierra Leone | Western sub-Saharan Africa | female | 0.46 | 1.48 | 11.21 | 38.79 |
| Singapore | High-income Asia Pacific | female | 3.22 | 0.35 | 127.22 | 62.06 |
| Slovakia | Central Europe | female | 2.30 | 1.21 | 95.65 | 81.88 |
| Slovenia | Central Europe | female | 1.73 | 0.83 | 77.39 | 65.86 |
| Solomon Islands | Oceania | female | 0.54 | 2.24 | 17.29 | 70.50 |
| Somalia | Eastern Sub-Saharan Africa | female | 0.20 | 0.28 | 5.53 | 8.08 |
| South Africa | Southern sub-Saharan Africa | female | 4.93 | 10.88 | 139.94 | 301.19 |
| Republic of Korea | High-income Asia Pacific | female | 2.54 | 2.48 | 96.95 | 101.85 |
| South Sudan | Eastern Sub-Saharan Africa | female | 0.60 | 1.00 | 16.00 | 28.88 |
| Spain | Western Europe | female | 2.05 | 0.62 | 67.36 | 41.26 |
| Sri Lanka | Southeast Asia | female | 0.99 | 5.41 | 36.32 | 178.23 |
| Sudan | North Africa and Middle East | female | 0.33 | 2.17 | 13.37 | 111.12 |
| Suriname | Caribbean | female | 3.49 | 5.92 | 127.25 | 263.94 |
| Eswatini | Southern sub-Saharan Africa | female | 2.48 | 9.21 | 61.80 | 223.43 |
| Sweden | Western Europe | female | 0.60 | 0.19 | 23.02 | 10.00 |
| Switzerland | Western Europe | female | 1.96 | 0.47 | 60.21 | 30.90 |
| Syrian Arab Republic | North Africa and Middle East | female | 4.07 | 4.19 | 144.24 | 190.06 |
| Taiwan (Province of China) | East Asia | female | 4.47 | 4.39 | 130.35 | 147.39 |
| Tajikistan | Central Asia | female | 0.57 | 3.98 | 26.56 | 158.73 |
| United Republic of Tanzania | Eastern Sub-Saharan Africa | female | 0.38 | 0.96 | 9.23 | 26.52 |
| Thailand | Southeast Asia | female | 2.34 | 3.06 | 73.22 | 128.50 |
| Bahamas | Caribbean | female | 6.56 | 4.99 | 202.98 | 196.74 |
| Gambia | Western sub-Saharan Africa | female | 0.52 | 2.12 | 14.48 | 59.71 |
| Timor-Leste | Southeast Asia | female | 0.29 | 0.95 | 8.35 | 32.88 |
| Togo | Western sub-Saharan Africa | female | 0.68 | 1.72 | 17.58 | 46.15 |
| Tonga | Oceania | female | 2.67 | 5.60 | 74.30 | 165.49 |
| Trinidad and Tobago | Caribbean | female | 19.47 | 15.23 | 575.14 | 481.42 |
| Tunisia | North Africa and Middle East | female | 1.78 | 2.97 | 81.29 | 178.46 |
| Turkey | North Africa and Middle East | female | 6.71 | 4.77 | 181.46 | 172.42 |
| Turkmenistan | Central Asia | female | 1.56 | 2.94 | 77.90 | 154.21 |
| Uganda | Eastern Sub-Saharan Africa | female | 0.35 | 1.13 | 9.90 | 34.38 |
| Ukraine | Eastern Europe | female | 0.55 | 0.35 | 43.88 | 40.16 |
| United Arab Emirates | North Africa and Middle East | female | 19.71 | 11.86 | 466.47 | 401.82 |
| United Kingdom | Western Europe | female | 1.05 | 0.32 | 47.30 | 43.23 |
| United States of America | High-income North America | female | 1.52 | 0.61 | 67.17 | 38.75 |
| Uruguay | Southern Latin America | female | 1.10 | 0.99 | 26.23 | 34.70 |
| Uzbekistan | Central Asia | female | 0.82 | 6.23 | 38.40 | 227.77 |
| Vanuatu | Oceania | female | 0.65 | 2.18 | 20.42 | 69.48 |
| Venezuela (Bolivarian Republic of) | Central Latin America | female | 5.06 | 5.97 | 173.63 | 231.14 |
| Viet nam | Southeast Asia | female | 1.03 | 3.29 | 27.18 | 97.19 |
| United States Virgin Islands | Caribbean | female | 3.37 | 2.78 | 99.37 | 106.03 |
| Yemen | North Africa and Middle East | female | 0.32 | 1.92 | 12.10 | 84.76 |
| Zambia | Eastern Sub-Saharan Africa | female | 0.70 | 1.68 | 19.16 | 48.93 |
| Zimbabwe | Southern sub-Saharan Africa | female | 1.23 | 2.55 | 34.61 | 75.90 |
| Monaco | Western Europe | female | 0.21 | 0.23 | 15.47 | 32.74 |
| San Marino | Western Europe | female | 0.71 | 0.43 | 28.31 | 32.54 |
| Saint Kitts and Nevis | Caribbean | female | 3.78 | 3.52 | 109.49 | 121.16 |
| Cook Islands | Oceania | female | 4.00 | 4.54 | 106.37 | 133.58 |
| Nauru | Oceania | female | 2.86 | 5.39 | 75.48 | 146.61 |
| Niue | Oceania | female | 2.31 | 5.45 | 67.32 | 173.47 |
| Palau | Oceania | female | 3.85 | 5.43 | 110.77 | 163.72 |
| Tokelau | Oceania | female | 4.84 | 5.54 | 138.86 | 163.67 |
| Tuvalu | Oceania | female | 1.06 | 3.94 | 30.69 | 116.08 |

**Supplementary Table 4.The top three and the bottom three regions of type 2 diabetes burden attributed to ambient particulate matter pollution.**

| **Measure** | **sex** | **Top three regions** |  |  | **Bottom three regions** |  |  |
| --- | --- | --- | --- | --- | --- | --- | --- |
| 2019 ASR (per 100,000 people) |  |  |  |  |  |  |  |
| Age-standardized DALY rate |  |  |  |  |  |  |  |
|  | both | North Africa and Middle East(220.55) | Central Latin America(246.53) | Southern sub-Saharan Africa(280.44) | Australasia(18.31) | Eastern Europe(39.97) | Eastern Sub-Saharan Africa(40.18) |
|  | female | Central Latin America(219.21) | North Africa and Middle East(223.02) | Southern sub-Saharan Africa(266.01) | Australasia(15.42) | Eastern Sub-Saharan Africa(26.99) | High-income North America(36.41) |
|  | male | North Africa and Middle East(217.85) | Central Latin America(277.51) | Southern sub-Saharan Africa(299.26) | Australasia(21.49) | Eastern Europe(40.10) | High-income North America(50.56) |
| ASDR |  |  |  |  |  |  |  |
|  | both | North Africa and Middle East(5.28) | Central Latin America(6.34) | Southern sub-Saharan Africa(10.29) | Australasia(0.38) | Eastern Europe(0.63) | High-income North America(0.71) |
|  | female | North Africa and Middle East(5.65) | Central Latin America(5.75) | Southern sub-Saharan Africa(9.75) | Australasia(0.30) | High-income Asia Pacific(0.56) | High-income North America(0.58) |
|  | male | Oceania(5.50) | Central Latin America(7.00) | Southern sub-Saharan Africa(11.04) | Australasia(0.48) | Eastern Europe(0.57) | High-income North America(0.86) |
| 1990-2019 increase times |  |  |  |  |  |  |  |
| DALY (cases) |  |  |  |  |  |  |  |
|  | both | Oceania(6.18) | Western sub-Saharan Africa(6.60) | South Asia(9.39) | High-income North America(1.10) | Western Europe(1.12) | Eastern Europe(1.21) |
|  | female | Oceania(6.95) | Western sub-Saharan Africa(7.55) | South Asia(10.89) | High-income North America(0.95) | Western Europe(0.97) | Eastern Europe(1.17) |
|  | male | Central Asia(6.00) | East Asia(6.04) | South Asia(8.46) | High-income North America(1.26) | Eastern Europe(1.29) | Western Europe(1.32) |
| Death (case) |  |  |  |  |  |  |  |
|  | both | Western sub-Saharan Africa(6.23) | Central Asia(6.61) | South Asia(9.60) | Western Europe(0.84) | High-income North America(0.84) | Eastern Europe(1.40) |
|  | female | Western sub-Saharan Africa(6.79) | Oceania(7.05) | South Asia(11.41) | High-income North America(0.69) | Western Europe(0.72) | Eastern Europe(1.38) |
|  | male | East Asia(6.63) | Central Asia(6.77) | South Asia(8.43) | Western Europe(1.05) | High-income North America(1.05) | Eastern Europe(1.47) |
| EAPC |  |  |  |  |  |  |  |
| DALY |  |  |  |  |  |  |  |
|  | both | Western sub-Saharan Africa(4.10) | Central Asia(4.43) | South Asia(4.88) | High-income North America(-1.55) | Western Europe(-0.96) | Australasia(-0.75) |
|  | female | Western sub-Saharan Africa(4.34) | Central Asia(4.57) | South Asia(5.21) | High-income North America(-1.88) | Western Europe(-1.26) | Australasia(-0.84) |
|  | male | Western sub-Saharan Africa(4.00) | Central Asia(4.28) | South Asia(4.71) | High-income North America(-1.30) | Australasia(-0.73) | Western Europe(-0.67) |
| Death |  |  |  |  |  |  |  |
|  | both | Western sub-Saharan Africa(4.07) | South Asia(4.66) | Central Asia(4.84) | High-income North America(-3.47) | Western Europe(-2.79) | Australasia(-2.08) |
|  | female | Western sub-Saharan Africa(4.26) | Central Asia(4.94) | South Asia(4.95) | High-income North America(-4.05) | Western Europe(-3.34) | Australasia(-2.29) |
|  | male | Western sub-Saharan Africa(3.93) | South Asia(4.52) | Central Asia(4.70) | High-income North America(-2.98) | Western Europe(-2.17) | Australasia(-2.05) |

**Supplementary Table 5.The top three and the bottom three countries of type 2 diabetes burden attributed to ambient particulate matter pollution.**

| **Measure** | **sex** | **Top three countries** |  |  | **Bottom three countries** |  |  |
| --- | --- | --- | --- | --- | --- | --- | --- |
| 2019 ASR (per 100,000 people) |  |  |  |  |  |  |  |
| Age-standardized DALY rate |  |  |  |  |  |  |  |
|  | both | Trinidad and Tobago(529.64) | Qatar(722.09) | Bahrain(770.18) | Iceland(11.05) | Somalia(11.32) | Sweden(11.95) |
|  | female | Trinidad and Tobago(481.42) | Bahrain(761.95) | Qatar(880.83) | Somalia(8.08) | Iceland(9.14) | Sweden(10.00) |
|  | male | Trinidad and Tobago(580.40) | Qatar(672.11) | Bahrain(774.07) | Iceland(13.09) | Sweden(13.98) | Finland(14.54) |
| ASDR |  |  |  |  |  |  |  |
|  | both | Fiji(18.21) | Qatar(29.68) | Bahrain(30.34) | Finland(0.09) | Iceland(0.12) | Estonia(0.17) |
|  | female | Fiji(16.08) | Bahrain(30.89) | Qatar(42.14) | Iceland(0.06) | Finland(0.07) | Estonia(0.15) |
|  | male | Fiji(21.52) | Qatar(26.10) | Bahrain(29.77) | Finland(0.12) | Iceland(0.18) | Estonia(0.19) |
| 1990-2019 increase times |  |  |  |  |  |  |  |
| DALY (cases) |  |  |  |  |  |  |  |
|  | both | Guatemala(19.55) | Djibouti(19.56) | Equatorial Guinea(24.63) | Sweden(0.60) | Norway(0.70) | Finland(0.77) |
|  | female | Cabo Verde(20.46) | Guatemala(22.76) | Equatorial Guinea(37.26) | Sweden(0.55) | Norway(0.66) | Finland(0.68) |
|  | male | Bhutan(17.18) | Equatorial Guinea(19.54) | Djibouti(19.59) | Sweden(0.66) | Norway(0.74) | Finland(0.89) |
| Death (case) |  |  |  |  |  |  |  |
|  | both | Equatorial Guinea(23.93) | Cabo Verde(25.41) | Guatemala(26.92) | Finland(0.39) | United Kingdom(0.47) | Singapore(0.49) |
|  | female | Cabo Verde(30.36) | Guatemala(30.85) | Equatorial Guinea(38.65) | Finland(0.29) | Singapore(0.41) | United Kingdom(0.44) |
|  | male | Cabo Verde(20.50) | Bhutan(21.03) | Guatemala(23.46) | United Kingdom(0.52) | Norway(0.56) | Finland(0.59) |
| EAPC |  |  |  |  |  |  |  |
| DALY |  |  |  |  |  |  |  |
|  | both | Sudan(8.01) | Bhutan(8.50) | Equatorial Guinea(9.99) | Norway(-3.00) | Sweden(-2.57) | Bermuda(-2.51) |
|  | female | Bhutan(8.44) | Cabo Verde(8.59) | Equatorial Guinea(11.68) | Norway(-3.02) | Bermuda(-2.88) | Cyprus(-2.85) |
|  | male | Sudan(7.83) | Bhutan(8.45) | Equatorial Guinea(9.03) | Norway(-3.03) | Sweden(-2.55) | Bermuda(-2.20) |
| Death |  |  |  |  |  |  |  |
|  | both | Bhutan(8.76) | Cabo Verde(8.84) | Equatorial Guinea(10.35) | Singapore(-7.25) | Finland(-5.75) | Switzerland(-4.90) |
|  | female | Bhutan(8.75) | Cabo Verde(9.65) | Equatorial Guinea(12.15) | Singapore(-7.92) | Finland(-6.71) | Switzerland(-5.43) |
|  | male | Bosnia and Herzegovina(7.65) | Bhutan(8.54) | Equatorial Guinea(9.33) | Singapore(-6.46) | Finland(-4.65) | United Kingdom(-4.55) |

**Supplementary Table 6. Age distribution of DALY rate for type 2 diabetes burden attributed to ambient particulate matter pollution in different countries in 2019.**

| **2019 DALY rate** | **25-29 years** | **30-34 years** | **35-39 years** | **40-44 years** | **45-49 years** | **50-54 years** | **55-59 years** | **60-64 years** | **65-69 years** | **70-74 years** | **75-79 years** | **80-84** | **85+ years** |
| --- | --- | --- | --- | --- | --- | --- | --- | --- | --- | --- | --- | --- | --- |
| Afghanistan | 11.95 | 21.40 | 34.25 | 58.81 | 113.52 | 193.15 | 287.34 | 386.94 | 448.93 | 486.78 | 490.68 | 465.95 | 432.07 |
| Albania | 5.81 | 12.49 | 20.95 | 32.73 | 53.54 | 78.98 | 111.72 | 157.26 | 203.85 | 249.67 | 274.36 | 280.08 | 250.26 |
| Algeria | 24.68 | 45.16 | 73.59 | 121.48 | 220.43 | 366.39 | 542.34 | 767.48 | 946.81 | 1078.02 | 1207.36 | 1274.30 | 1692.58 |
| American Samoa | 26.37 | 49.12 | 85.25 | 148.81 | 239.72 | 363.87 | 481.24 | 573.22 | 683.02 | 748.59 | 779.26 | 787.88 | 790.31 |
| Andorra | 5.06 | 8.38 | 12.82 | 19.31 | 33.00 | 49.99 | 72.56 | 102.48 | 134.28 | 169.16 | 196.31 | 216.92 | 231.65 |
| Angola | 12.10 | 23.26 | 38.97 | 65.38 | 135.15 | 247.28 | 333.86 | 470.44 | 518.48 | 643.31 | 687.09 | 764.06 | 700.62 |
| Antigua and Barbuda | 33.12 | 56.25 | 91.19 | 143.33 | 284.75 | 493.50 | 744.36 | 1074.84 | 1288.62 | 1624.09 | 1878.06 | 2229.06 | 2026.07 |
| Argentina | 7.34 | 15.16 | 26.30 | 44.55 | 90.99 | 150.12 | 236.95 | 344.51 | 448.09 | 516.30 | 565.06 | 558.98 | 529.97 |
| Armenia | 18.84 | 36.67 | 61.76 | 106.91 | 224.27 | 367.91 | 606.93 | 895.80 | 1194.37 | 1421.99 | 1393.66 | 1201.57 | 821.58 |
| Australia | 1.31 | 3.48 | 6.56 | 11.71 | 21.04 | 31.74 | 44.69 | 61.40 | 83.16 | 108.29 | 134.44 | 164.04 | 189.79 |
| Austria | 4.97 | 9.50 | 15.76 | 25.64 | 48.47 | 80.85 | 127.82 | 194.76 | 263.27 | 323.08 | 380.46 | 465.86 | 515.48 |
| Azerbaijan | 18.88 | 34.21 | 55.34 | 92.55 | 199.02 | 333.85 | 523.41 | 739.00 | 930.58 | 939.84 | 903.58 | 762.93 | 664.21 |
| Bahamas | 34.38 | 63.43 | 99.61 | 149.97 | 271.16 | 428.28 | 543.01 | 741.66 | 942.11 | 1042.06 | 1194.60 | 1250.79 | 1147.10 |
| Bahrain | 47.87 | 71.94 | 114.07 | 212.99 | 459.07 | 883.69 | 1632.95 | 2644.78 | 3850.88 | 5435.12 | 6658.84 | 7145.78 | 8062.15 |
| Bangladesh | 12.88 | 21.91 | 34.40 | 62.02 | 116.45 | 167.80 | 192.61 | 271.71 | 363.61 | 392.54 | 502.75 | 911.84 | 1149.04 |
| Barbados | 42.13 | 78.08 | 115.42 | 181.56 | 338.94 | 536.08 | 775.15 | 1074.76 | 1399.46 | 1800.36 | 2254.77 | 2480.96 | 2643.89 |
| Belarus | 6.68 | 15.24 | 25.31 | 37.40 | 60.70 | 85.73 | 114.78 | 146.36 | 173.90 | 188.01 | 197.76 | 205.35 | 211.53 |
| Belgium | 8.03 | 13.61 | 21.53 | 33.29 | 58.32 | 89.91 | 134.20 | 188.60 | 243.28 | 295.84 | 331.68 | 373.18 | 421.02 |
| Belize | 35.05 | 66.06 | 90.47 | 145.15 | 304.33 | 522.65 | 747.10 | 933.13 | 1134.50 | 1317.68 | 1378.33 | 1374.28 | 1202.83 |
| Benin | 5.61 | 9.48 | 12.55 | 27.41 | 56.09 | 97.02 | 133.21 | 197.86 | 284.09 | 310.90 | 384.02 | 411.01 | 369.50 |
| Bermuda | 5.98 | 10.08 | 15.98 | 25.63 | 47.37 | 75.61 | 105.29 | 139.65 | 177.52 | 217.25 | 253.72 | 287.42 | 292.45 |
| Bhutan | 11.14 | 20.59 | 33.01 | 58.66 | 106.48 | 167.81 | 241.27 | 358.32 | 445.15 | 549.70 | 699.98 | 807.70 | 837.06 |
| Bolivia (Plurinational State of) | 11.47 | 21.77 | 38.81 | 75.10 | 160.49 | 287.44 | 467.06 | 674.47 | 889.50 | 1107.89 | 1242.61 | 1212.42 | 1011.90 |
| Bosnia and Herzegovina | 16.55 | 33.78 | 56.11 | 93.03 | 177.97 | 298.56 | 489.13 | 766.41 | 1069.45 | 1437.57 | 1670.68 | 1763.99 | 1444.04 |
| Botswana | 16.84 | 27.14 | 52.72 | 113.79 | 244.64 | 465.10 | 776.21 | 1377.07 | 1683.89 | 1963.63 | 2090.41 | 1864.53 | 1734.89 |
| Brazil | 9.29 | 17.96 | 31.31 | 54.06 | 99.12 | 159.90 | 238.20 | 336.34 | 431.97 | 520.69 | 588.53 | 620.20 | 609.15 |
| Brunei Darussalam | 11.64 | 24.43 | 42.82 | 74.96 | 142.61 | 209.63 | 347.10 | 508.43 | 695.59 | 842.77 | 952.24 | 1008.06 | 935.48 |
| Bulgaria | 12.31 | 25.86 | 42.01 | 66.01 | 131.55 | 206.70 | 296.67 | 423.18 | 527.67 | 622.46 | 689.37 | 641.52 | 556.00 |
| Burkina Faso | 3.95 | 7.65 | 9.24 | 22.39 | 44.68 | 76.07 | 104.07 | 139.10 | 184.30 | 186.29 | 244.34 | 283.46 | 270.49 |
| Burundi | 2.34 | 4.60 | 7.93 | 13.45 | 28.71 | 51.43 | 72.05 | 106.82 | 119.73 | 153.27 | 168.39 | 181.51 | 164.93 |
| Cabo Verde | 17.34 | 29.25 | 43.62 | 89.07 | 181.77 | 294.54 | 430.41 | 629.49 | 932.03 | 1091.88 | 1393.02 | 1655.65 | 1599.22 |
| Cambodia | 6.65 | 12.32 | 20.01 | 33.95 | 61.87 | 102.06 | 150.37 | 206.46 | 259.65 | 310.31 | 342.35 | 344.20 | 329.16 |
| Cameroon | 15.33 | 26.14 | 32.68 | 81.85 | 163.64 | 280.39 | 377.79 | 567.33 | 828.30 | 888.09 | 1100.35 | 1180.66 | 1030.25 |
| Canada | 0.75 | 2.50 | 5.24 | 9.70 | 20.58 | 32.64 | 49.57 | 74.54 | 99.32 | 120.82 | 139.07 | 156.95 | 176.47 |
| Central African Republic | 5.73 | 11.21 | 19.98 | 35.68 | 80.17 | 144.25 | 187.21 | 236.31 | 241.34 | 272.89 | 269.63 | 265.88 | 228.30 |
| Chad | 3.62 | 5.98 | 8.28 | 17.94 | 37.05 | 65.33 | 88.85 | 134.50 | 195.40 | 211.10 | 254.27 | 263.33 | 229.48 |
| Chile | 8.76 | 18.21 | 33.01 | 58.06 | 110.89 | 194.00 | 315.61 | 478.27 | 635.54 | 743.67 | 859.27 | 924.23 | 1016.98 |
| China | 20.90 | 35.30 | 54.72 | 80.46 | 117.63 | 166.89 | 227.40 | 297.61 | 379.21 | 472.65 | 515.54 | 535.45 | 537.66 |
| Colombia | 24.45 | 40.39 | 63.54 | 100.37 | 162.46 | 249.56 | 362.71 | 503.20 | 616.67 | 712.12 | 775.79 | 784.34 | 753.09 |
| Comoros | 3.02 | 5.82 | 10.07 | 16.74 | 34.61 | 60.34 | 85.36 | 130.99 | 149.64 | 201.31 | 229.23 | 263.25 | 247.23 |
| Congo | 17.90 | 34.56 | 62.38 | 107.31 | 224.70 | 406.15 | 569.20 | 772.46 | 861.13 | 1061.74 | 1168.70 | 1254.19 | 1262.15 |
| Cook Islands | 16.66 | 33.12 | 61.21 | 115.71 | 191.94 | 303.84 | 409.00 | 510.08 | 636.22 | 725.56 | 762.31 | 812.69 | 869.49 |
| Costa Rica | 23.65 | 38.64 | 61.09 | 97.74 | 161.83 | 243.81 | 341.48 | 470.54 | 574.23 | 659.56 | 740.06 | 758.37 | 743.93 |
| Croatia | 10.60 | 22.94 | 40.49 | 66.01 | 114.34 | 179.92 | 273.78 | 390.36 | 512.95 | 635.46 | 714.35 | 789.73 | 776.85 |
| Cuba | 26.41 | 42.94 | 66.69 | 102.18 | 170.03 | 259.16 | 353.50 | 453.21 | 557.06 | 645.55 | 721.77 | 728.43 | 689.49 |
| Cyprus | 8.46 | 14.07 | 22.52 | 35.02 | 61.87 | 111.64 | 210.19 | 360.87 | 534.04 | 717.53 | 920.54 | 1324.15 | 1609.41 |
| Czechia | 14.97 | 31.76 | 56.45 | 92.77 | 164.45 | 261.01 | 397.14 | 569.50 | 724.52 | 857.52 | 945.09 | 1052.32 | 1084.46 |
| Côte d'Ivoire | 8.70 | 14.69 | 19.03 | 44.81 | 90.45 | 154.01 | 210.24 | 316.13 | 458.27 | 495.29 | 623.65 | 678.33 | 597.25 |
| Democratic People's Republic of Korea | 12.46 | 20.00 | 30.50 | 46.93 | 82.01 | 121.97 | 172.77 | 229.99 | 284.96 | 315.34 | 323.20 | 285.08 | 247.00 |
| Democratic Republic of the Congo | 5.62 | 10.80 | 18.16 | 30.79 | 64.04 | 116.43 | 154.34 | 208.69 | 226.81 | 274.88 | 293.01 | 318.38 | 297.73 |
| Denmark | 5.35 | 9.32 | 13.72 | 20.11 | 38.88 | 62.46 | 86.64 | 121.69 | 156.17 | 197.84 | 239.74 | 281.20 | 323.19 |
| Djibouti | 15.70 | 30.42 | 52.31 | 89.57 | 186.77 | 337.58 | 475.64 | 727.27 | 835.08 | 1114.48 | 1258.18 | 1412.04 | 1310.76 |
| Dominica | 46.78 | 81.49 | 130.61 | 204.66 | 373.97 | 584.22 | 813.36 | 1086.21 | 1441.54 | 1699.56 | 1958.00 | 2123.27 | 2035.39 |
| Dominican Republic | 28.05 | 48.24 | 71.35 | 113.63 | 191.62 | 304.78 | 389.19 | 543.18 | 605.66 | 701.19 | 703.04 | 711.57 | 693.11 |
| Ecuador | 19.48 | 35.64 | 57.96 | 96.74 | 185.62 | 308.49 | 485.73 | 674.63 | 855.07 | 1018.90 | 1168.28 | 1242.58 | 1449.40 |
| Egypt | 29.16 | 53.58 | 84.63 | 145.95 | 321.13 | 612.11 | 918.48 | 1303.53 | 1531.41 | 1566.14 | 1527.42 | 1515.36 | 1495.09 |
| El Salvador | 29.71 | 57.46 | 93.94 | 155.04 | 292.83 | 443.56 | 648.49 | 849.48 | 995.09 | 1087.21 | 1156.64 | 1098.71 | 927.83 |
| Equatorial Guinea | 27.70 | 53.49 | 90.44 | 150.54 | 305.28 | 544.81 | 770.90 | 1108.14 | 1316.04 | 1676.24 | 1975.49 | 2241.23 | 2234.20 |
| Eritrea | 7.44 | 15.13 | 27.13 | 46.88 | 102.66 | 175.96 | 243.73 | 343.36 | 369.71 | 453.05 | 490.22 | 515.08 | 479.05 |
| Estonia | 2.37 | 5.10 | 8.99 | 13.54 | 24.33 | 34.43 | 45.01 | 58.42 | 70.63 | 79.29 | 83.06 | 81.83 | 76.48 |
| Eswatini | 18.22 | 29.11 | 58.89 | 134.94 | 298.15 | 535.61 | 837.87 | 1428.01 | 1707.43 | 1900.83 | 2011.71 | 1810.87 | 1687.48 |
| Ethiopia | 2.54 | 5.26 | 9.12 | 14.99 | 32.16 | 58.10 | 84.03 | 128.94 | 153.03 | 213.99 | 248.12 | 272.80 | 254.46 |
| Fiji | 38.15 | 75.90 | 152.64 | 315.33 | 585.75 | 1037.02 | 1485.82 | 1986.93 | 2388.63 | 2681.70 | 2545.73 | 2642.33 | 2720.91 |
| Finland | 3.42 | 5.46 | 7.96 | 11.51 | 18.88 | 26.44 | 35.55 | 46.28 | 57.37 | 67.64 | 74.82 | 79.78 | 80.29 |
| France | 3.18 | 5.98 | 9.54 | 15.09 | 29.21 | 44.82 | 68.72 | 98.14 | 130.45 | 165.22 | 197.79 | 238.16 | 301.53 |
| Gabon | 31.46 | 63.09 | 110.99 | 191.58 | 410.79 | 766.54 | 1090.92 | 1545.23 | 1755.91 | 2140.08 | 2298.90 | 2441.49 | 2430.41 |
| Gambia | 5.74 | 9.93 | 13.83 | 31.37 | 67.11 | 119.56 | 167.82 | 249.89 | 369.85 | 391.52 | 485.04 | 521.90 | 488.44 |
| Georgia | 13.39 | 25.90 | 43.67 | 72.52 | 154.63 | 256.39 | 367.51 | 507.58 | 609.06 | 690.26 | 699.60 | 578.08 | 386.57 |
| Germany | 11.47 | 18.45 | 27.87 | 42.08 | 72.22 | 110.58 | 160.96 | 225.52 | 286.24 | 349.94 | 408.95 | 474.78 | 565.09 |
| Ghana | 15.60 | 29.30 | 37.69 | 92.58 | 178.04 | 310.12 | 432.95 | 631.12 | 923.06 | 944.57 | 1156.81 | 1179.36 | 1059.54 |
| Greece | 10.04 | 16.38 | 24.96 | 38.72 | 66.38 | 107.47 | 160.30 | 220.56 | 282.68 | 330.30 | 370.06 | 371.39 | 351.30 |
| Greenland | 1.01 | 3.44 | 6.42 | 11.99 | 22.80 | 36.12 | 54.07 | 76.50 | 95.78 | 114.09 | 118.91 | 118.60 | 108.66 |
| Grenada | 44.96 | 86.98 | 140.20 | 221.24 | 415.43 | 658.19 | 958.00 | 1377.30 | 1919.11 | 2219.59 | 2444.66 | 2576.90 | 2407.65 |
| Guam | 17.27 | 29.71 | 49.56 | 78.65 | 117.78 | 187.96 | 231.37 | 280.37 | 330.11 | 341.68 | 369.22 | 357.07 | 355.86 |
| Guatemala | 25.73 | 53.80 | 96.92 | 163.86 | 299.38 | 461.19 | 620.50 | 783.86 | 911.47 | 924.65 | 907.13 | 957.06 | 1081.96 |
| Guinea | 4.73 | 7.28 | 10.21 | 22.36 | 45.79 | 82.79 | 112.42 | 171.45 | 246.92 | 264.04 | 316.37 | 334.96 | 286.73 |
| Guinea-Bissau | 7.14 | 11.71 | 16.64 | 38.94 | 82.59 | 133.92 | 179.36 | 255.42 | 355.37 | 358.75 | 416.55 | 436.97 | 382.88 |
| Guyana | 58.52 | 107.28 | 164.04 | 290.30 | 586.29 | 990.62 | 1402.75 | 1825.91 | 2104.20 | 2307.04 | 2376.83 | 2252.88 | 2120.97 |
| Haiti | 8.85 | 15.64 | 24.93 | 42.29 | 85.69 | 141.17 | 200.51 | 268.95 | 323.51 | 370.69 | 390.12 | 391.49 | 324.59 |
| Honduras | 14.07 | 23.41 | 38.14 | 63.61 | 110.23 | 171.68 | 239.01 | 309.71 | 365.66 | 404.35 | 422.76 | 402.47 | 372.81 |
| Hungary | 10.28 | 22.56 | 39.62 | 65.41 | 122.13 | 195.30 | 285.83 | 385.06 | 481.09 | 564.04 | 638.92 | 650.84 | 585.39 |
| Iceland | 2.18 | 3.53 | 5.38 | 8.02 | 12.98 | 19.33 | 27.63 | 38.20 | 48.89 | 60.00 | 69.03 | 75.40 | 75.60 |
| India | 21.25 | 40.21 | 65.98 | 115.60 | 203.45 | 330.26 | 463.43 | 635.72 | 739.67 | 832.70 | 965.64 | 1100.53 | 1043.87 |
| Indonesia | 17.34 | 36.75 | 68.98 | 131.30 | 251.87 | 390.18 | 550.38 | 685.71 | 750.11 | 788.71 | 834.03 | 793.40 | 763.03 |
| Iran (Islamic Republic of) | 20.36 | 37.93 | 64.51 | 108.37 | 204.20 | 363.36 | 573.76 | 834.22 | 1059.25 | 1251.20 | 1442.60 | 1447.22 | 1347.62 |
| Iraq | 48.23 | 81.34 | 129.50 | 210.32 | 410.72 | 687.40 | 1057.97 | 1475.31 | 1796.56 | 2082.16 | 2221.89 | 2181.04 | 2184.11 |
| Ireland | 3.41 | 5.90 | 9.32 | 14.45 | 24.33 | 37.14 | 54.94 | 76.96 | 103.46 | 136.25 | 169.46 | 193.28 | 218.65 |
| Israel | 11.77 | 19.11 | 29.61 | 47.16 | 93.51 | 167.54 | 260.91 | 378.27 | 518.81 | 672.96 | 852.54 | 992.54 | 1220.37 |
| Italy | 7.68 | 14.42 | 24.05 | 39.17 | 72.22 | 122.33 | 193.96 | 285.92 | 378.03 | 465.66 | 545.43 | 623.31 | 726.19 |
| Jamaica | 27.62 | 52.80 | 80.36 | 141.94 | 277.84 | 471.18 | 701.42 | 981.19 | 1311.59 | 1688.62 | 1980.63 | 2035.49 | 1893.11 |
| Japan | 6.37 | 12.72 | 22.42 | 36.29 | 57.84 | 81.16 | 106.93 | 135.43 | 162.78 | 186.64 | 200.80 | 208.62 | 210.88 |
| Jordan | 26.35 | 45.33 | 69.09 | 114.79 | 226.43 | 418.92 | 670.56 | 1021.12 | 1426.55 | 1710.74 | 1967.00 | 2091.18 | 2130.11 |
| Kazakhstan | 16.97 | 30.28 | 49.47 | 78.19 | 144.65 | 226.79 | 351.16 | 500.45 | 643.10 | 717.54 | 756.92 | 715.85 | 548.87 |
| Kenya | 4.76 | 10.12 | 17.85 | 29.50 | 62.32 | 109.44 | 146.28 | 202.95 | 227.29 | 299.92 | 329.73 | 355.31 | 323.73 |
| Kiribati | 18.47 | 36.10 | 67.72 | 146.84 | 259.95 | 384.31 | 477.46 | 528.88 | 596.21 | 592.77 | 461.88 | 424.97 | 515.90 |
| Kuwait | 42.74 | 72.63 | 110.80 | 169.15 | 282.59 | 459.02 | 689.51 | 975.50 | 1259.53 | 1577.82 | 1723.16 | 1864.35 | 1935.42 |
| Kyrgyzstan | 9.85 | 17.38 | 28.49 | 45.52 | 79.27 | 118.07 | 174.92 | 225.35 | 270.81 | 252.65 | 241.02 | 211.70 | 208.15 |
| Lao People's Democratic Republic | 8.05 | 14.80 | 23.41 | 39.10 | 67.70 | 108.36 | 158.82 | 220.33 | 274.15 | 325.66 | 357.45 | 365.61 | 348.64 |
| Latvia | 8.50 | 18.40 | 28.98 | 42.46 | 73.44 | 110.15 | 142.89 | 186.90 | 229.79 | 263.72 | 273.24 | 283.53 | 265.73 |
| Lebanon | 26.46 | 47.19 | 75.48 | 122.79 | 223.73 | 379.97 | 549.77 | 745.31 | 1019.66 | 1225.33 | 1303.15 | 1265.58 | 1246.69 |
| Lesotho | 12.50 | 20.51 | 40.97 | 93.49 | 209.16 | 386.56 | 615.77 | 1070.07 | 1229.31 | 1329.18 | 1362.21 | 1177.98 | 1089.20 |
| Liberia | 6.53 | 10.49 | 14.77 | 30.23 | 60.77 | 102.92 | 141.72 | 214.63 | 304.54 | 333.01 | 410.97 | 449.73 | 423.15 |
| Libya | 30.54 | 56.75 | 92.94 | 154.34 | 294.73 | 489.65 | 725.61 | 1033.46 | 1267.50 | 1365.63 | 1447.29 | 1340.63 | 1301.30 |
| Lithuania | 4.78 | 10.90 | 18.65 | 27.53 | 50.71 | 68.15 | 88.54 | 116.10 | 137.49 | 149.55 | 152.49 | 150.38 | 141.18 |
| Luxembourg | 6.09 | 10.56 | 16.86 | 26.92 | 49.41 | 82.77 | 130.24 | 190.16 | 242.81 | 290.63 | 327.00 | 351.67 | 365.11 |
| Madagascar | 2.37 | 4.48 | 7.69 | 12.69 | 25.35 | 43.58 | 61.83 | 91.30 | 101.99 | 129.55 | 140.19 | 155.31 | 149.83 |
| Malawi | 2.98 | 5.73 | 9.38 | 15.59 | 33.29 | 59.31 | 80.83 | 117.57 | 135.83 | 176.57 | 194.45 | 204.12 | 174.59 |
| Malaysia | 17.92 | 32.61 | 51.50 | 79.39 | 135.10 | 212.60 | 310.98 | 433.83 | 566.78 | 693.15 | 742.30 | 734.30 | 720.24 |
| Maldives | 7.29 | 13.48 | 21.38 | 33.46 | 56.97 | 90.78 | 137.45 | 207.22 | 287.81 | 373.04 | 425.93 | 480.35 | 503.28 |
| Mali | 3.88 | 6.09 | 8.87 | 18.37 | 37.63 | 65.65 | 88.71 | 135.39 | 197.44 | 220.51 | 267.20 | 290.72 | 250.69 |
| Malta | 10.97 | 18.41 | 28.10 | 42.83 | 76.26 | 119.42 | 182.97 | 265.14 | 352.05 | 438.82 | 522.10 | 593.61 | 626.77 |
| Marshall Islands | 29.78 | 56.77 | 100.80 | 176.69 | 279.76 | 421.40 | 534.80 | 629.68 | 726.00 | 776.58 | 759.47 | 761.75 | 746.05 |
| Mauritania | 9.67 | 15.28 | 22.15 | 47.32 | 98.39 | 177.67 | 250.44 | 403.63 | 601.53 | 676.74 | 892.38 | 1017.02 | 957.69 |
| Mauritius | 33.40 | 73.64 | 137.74 | 229.60 | 492.20 | 768.58 | 1171.90 | 1741.53 | 2215.01 | 2589.62 | 2731.41 | 2505.23 | 2283.16 |
| Mexico | 35.88 | 70.09 | 127.37 | 229.06 | 412.91 | 663.58 | 952.41 | 1252.59 | 1521.25 | 1687.67 | 1817.84 | 1833.90 | 1702.40 |
| Micronesia (Federated States of) | 32.59 | 70.08 | 112.14 | 208.65 | 404.69 | 663.58 | 855.00 | 998.29 | 1125.44 | 1228.30 | 1212.29 | 1200.90 | 1183.11 |
| Monaco | 7.75 | 12.64 | 18.64 | 27.69 | 44.88 | 67.14 | 95.58 | 129.65 | 165.08 | 202.15 | 229.63 | 248.69 | 253.53 |
| Mongolia | 8.85 | 18.41 | 27.96 | 43.14 | 80.37 | 114.52 | 137.38 | 163.49 | 201.50 | 207.99 | 203.94 | 197.65 | 196.46 |
| Montenegro | 12.94 | 27.16 | 46.94 | 75.40 | 139.44 | 223.73 | 341.49 | 492.11 | 640.76 | 747.91 | 820.26 | 841.39 | 773.30 |
| Morocco | 23.76 | 42.95 | 69.51 | 116.14 | 220.60 | 381.27 | 584.24 | 837.23 | 1043.40 | 1210.16 | 1269.50 | 1250.95 | 1189.78 |
| Mozambique | 2.50 | 5.08 | 8.95 | 15.18 | 32.08 | 56.82 | 77.54 | 109.82 | 116.37 | 134.69 | 139.36 | 152.52 | 152.60 |
| Myanmar | 16.06 | 37.06 | 55.50 | 82.72 | 134.78 | 244.46 | 336.05 | 454.15 | 574.28 | 668.07 | 784.45 | 760.11 | 790.02 |
| Namibia | 9.70 | 15.85 | 29.41 | 60.62 | 134.56 | 260.45 | 442.57 | 800.60 | 1006.96 | 1202.86 | 1323.95 | 1201.95 | 1104.34 |
| Nauru | 18.78 | 39.52 | 74.35 | 144.08 | 236.00 | 368.93 | 475.14 | 568.83 | 665.44 | 736.99 | 719.70 | 703.59 | 701.25 |
| Nepal | 14.79 | 26.39 | 43.07 | 61.46 | 110.90 | 165.92 | 245.28 | 301.62 | 348.16 | 415.19 | 541.39 | 685.99 | 795.87 |
| Netherlands | 6.03 | 10.56 | 16.72 | 25.99 | 47.59 | 73.00 | 106.89 | 148.22 | 195.32 | 247.33 | 296.26 | 355.42 | 454.89 |
| New Zealand | 1.42 | 3.29 | 5.54 | 9.40 | 16.20 | 24.78 | 34.46 | 44.98 | 56.71 | 71.07 | 90.98 | 106.54 | 115.86 |
| Nicaragua | 14.05 | 23.69 | 41.21 | 74.21 | 140.52 | 233.67 | 352.27 | 497.36 | 621.96 | 726.59 | 790.75 | 751.66 | 689.39 |
| Niger | 2.43 | 4.05 | 5.41 | 12.23 | 25.50 | 45.88 | 62.16 | 95.94 | 140.43 | 153.95 | 193.94 | 207.39 | 183.69 |
| Nigeria | 7.19 | 14.45 | 17.98 | 42.50 | 87.10 | 154.54 | 218.78 | 345.60 | 506.64 | 631.39 | 898.49 | 1012.56 | 974.63 |
| Niue | 26.60 | 48.56 | 84.65 | 153.03 | 253.04 | 409.07 | 552.69 | 691.06 | 840.53 | 939.68 | 951.19 | 949.06 | 947.32 |
| North Macedonia | 13.46 | 29.58 | 54.96 | 94.59 | 195.20 | 336.93 | 558.39 | 844.08 | 1172.71 | 1470.78 | 1829.54 | 1812.63 | 1578.07 |
| Northern Mariana Islands | 18.96 | 35.04 | 60.98 | 103.74 | 187.50 | 280.02 | 388.45 | 473.56 | 587.45 | 687.02 | 716.24 | 711.49 | 714.61 |
| Norway | 3.30 | 5.75 | 9.04 | 13.78 | 22.58 | 32.51 | 44.54 | 60.19 | 76.53 | 94.68 | 111.89 | 124.38 | 146.42 |
| Oman | 22.02 | 38.91 | 65.47 | 112.74 | 258.83 | 505.03 | 853.32 | 1373.83 | 2060.52 | 2482.82 | 2846.22 | 3270.92 | 2965.68 |
| Pakistan | 26.93 | 48.61 | 75.04 | 141.47 | 240.52 | 376.70 | 519.66 | 763.60 | 885.94 | 1037.52 | 1216.06 | 1324.29 | 1302.58 |
| Palau | 23.69 | 48.65 | 86.56 | 164.06 | 262.85 | 400.30 | 515.55 | 623.61 | 747.34 | 816.91 | 826.47 | 861.79 | 870.41 |
| Palestine | 26.32 | 43.40 | 73.48 | 127.62 | 275.44 | 522.60 | 998.96 | 1465.43 | 2101.19 | 2740.82 | 3215.06 | 3250.20 | 3125.15 |
| Panama | 17.98 | 30.09 | 49.23 | 84.02 | 153.40 | 259.63 | 375.85 | 518.70 | 640.12 | 745.91 | 841.51 | 880.42 | 821.69 |
| Papua New Guinea | 12.33 | 25.29 | 42.38 | 75.52 | 113.27 | 178.07 | 242.68 | 304.87 | 361.98 | 369.60 | 371.47 | 347.93 | 310.09 |
| Paraguay | 7.90 | 15.46 | 26.28 | 51.29 | 105.76 | 187.39 | 279.98 | 404.66 | 506.33 | 611.54 | 685.03 | 691.02 | 594.65 |
| Peru | 11.56 | 20.86 | 34.29 | 59.51 | 109.32 | 179.91 | 278.47 | 382.34 | 493.27 | 590.55 | 662.79 | 653.99 | 611.81 |
| Philippines | 14.63 | 27.76 | 45.31 | 75.88 | 136.95 | 222.06 | 326.38 | 438.64 | 554.38 | 613.23 | 635.78 | 633.56 | 595.22 |
| Poland | 13.41 | 30.38 | 55.79 | 91.64 | 161.61 | 236.17 | 330.03 | 446.60 | 557.51 | 646.15 | 706.29 | 730.92 | 714.74 |
| Portugal | 6.51 | 10.29 | 15.59 | 23.73 | 40.82 | 66.66 | 102.44 | 152.49 | 204.31 | 267.35 | 323.48 | 380.49 | 420.67 |
| Puerto Rico | 10.42 | 18.63 | 31.14 | 50.58 | 100.96 | 159.21 | 219.17 | 288.83 | 349.42 | 415.84 | 469.56 | 513.48 | 508.55 |
| Qatar | 32.57 | 59.80 | 105.49 | 190.09 | 442.63 | 873.08 | 1438.39 | 2121.56 | 2947.81 | 4332.81 | 6114.32 | 9145.93 | 10541.40 |
| Republic of Korea | 14.30 | 28.03 | 47.08 | 77.09 | 133.89 | 204.24 | 289.34 | 394.77 | 522.39 | 687.98 | 855.10 | 983.56 | 1055.72 |
| Republic of Moldova | 8.80 | 18.55 | 31.52 | 48.10 | 81.50 | 119.74 | 165.84 | 216.54 | 265.95 | 269.74 | 259.00 | 237.39 | 236.42 |
| Romania | 7.82 | 16.68 | 28.45 | 45.81 | 81.40 | 125.01 | 182.36 | 243.31 | 307.32 | 347.15 | 360.37 | 351.30 | 300.64 |
| Russian Federation | 4.04 | 8.80 | 15.51 | 24.44 | 43.77 | 65.93 | 98.14 | 146.63 | 198.40 | 230.50 | 256.18 | 245.90 | 206.24 |
| Rwanda | 4.21 | 8.45 | 14.31 | 23.39 | 49.48 | 89.61 | 128.01 | 193.10 | 226.68 | 299.00 | 341.94 | 391.92 | 362.43 |
| Saint Kitts and Nevis | 13.81 | 23.92 | 39.85 | 70.40 | 147.58 | 260.83 | 384.36 | 526.66 | 675.31 | 790.18 | 871.27 | 892.64 | 804.49 |
| Saint Lucia | 55.54 | 99.01 | 152.46 | 254.18 | 459.79 | 691.18 | 1000.31 | 1342.48 | 1654.89 | 1939.24 | 2127.44 | 2350.72 | 2663.03 |
| Saint Vincent and the Grenadines | 57.94 | 101.54 | 159.89 | 268.61 | 543.57 | 861.23 | 1117.41 | 1439.50 | 1840.62 | 2144.97 | 2500.97 | 2789.24 | 2682.37 |
| Samoa | 16.35 | 31.68 | 57.41 | 107.19 | 177.43 | 279.26 | 373.27 | 459.96 | 560.30 | 612.58 | 620.69 | 623.30 | 616.84 |
| San Marino | 6.34 | 10.27 | 15.41 | 22.86 | 38.43 | 59.30 | 87.23 | 124.01 | 162.77 | 204.17 | 238.65 | 266.56 | 292.27 |
| Sao Tome and Principe | 5.98 | 10.36 | 15.43 | 29.05 | 61.99 | 105.37 | 153.88 | 217.43 | 317.59 | 356.65 | 412.72 | 428.65 | 423.87 |
| Saudi Arabia | 41.01 | 70.93 | 110.83 | 178.16 | 308.58 | 494.54 | 732.47 | 943.55 | 1155.04 | 1322.65 | 1400.99 | 1432.80 | 1447.25 |
| Senegal | 9.05 | 14.85 | 20.59 | 42.68 | 84.02 | 146.35 | 208.02 | 317.07 | 458.86 | 510.87 | 620.77 | 675.67 | 622.27 |
| Serbia | 11.98 | 26.97 | 49.35 | 84.20 | 166.81 | 281.88 | 424.78 | 615.30 | 841.46 | 963.31 | 1127.30 | 1301.20 | 1204.87 |
| Seychelles | 22.52 | 43.27 | 72.33 | 123.26 | 212.68 | 330.46 | 478.62 | 679.95 | 904.30 | 1065.16 | 1241.38 | 1257.04 | 1231.03 |
| Sierra Leone | 3.94 | 6.15 | 8.38 | 19.05 | 39.38 | 69.53 | 91.85 | 141.61 | 204.53 | 219.22 | 278.95 | 311.02 | 269.82 |
| Singapore | 14.94 | 27.11 | 46.32 | 70.49 | 113.75 | 155.14 | 211.84 | 271.34 | 328.69 | 372.14 | 390.81 | 399.05 | 385.64 |
| Slovakia | 8.71 | 19.43 | 35.11 | 57.92 | 106.70 | 166.49 | 244.31 | 351.65 | 445.67 | 521.54 | 563.12 | 577.70 | 503.34 |
| Slovenia | 8.57 | 18.49 | 33.07 | 52.98 | 87.71 | 133.83 | 195.51 | 282.19 | 365.25 | 439.26 | 518.84 | 539.22 | 493.07 |
| Solomon Islands | 15.86 | 33.69 | 60.08 | 106.94 | 159.61 | 226.21 | 276.22 | 305.44 | 333.52 | 339.70 | 328.18 | 280.61 | 257.36 |
| Somalia | 1.05 | 2.05 | 3.66 | 6.41 | 13.86 | 24.71 | 33.37 | 47.33 | 50.98 | 62.28 | 65.28 | 66.77 | 58.62 |
| South Africa | 25.44 | 41.20 | 66.82 | 120.91 | 236.98 | 460.72 | 786.01 | 1417.33 | 1789.21 | 1943.06 | 2026.38 | 2191.85 | 2141.77 |
| South Sudan | 3.67 | 6.83 | 11.83 | 19.32 | 39.96 | 72.57 | 105.08 | 160.06 | 189.83 | 254.04 | 295.30 | 327.77 | 297.06 |
| Spain | 7.14 | 11.59 | 17.81 | 27.19 | 45.91 | 73.82 | 113.24 | 161.96 | 211.88 | 263.45 | 311.04 | 355.59 | 435.98 |
| Sri Lanka | 19.44 | 36.33 | 60.48 | 103.33 | 190.22 | 317.42 | 467.10 | 695.93 | 988.08 | 1332.91 | 1484.61 | 1671.70 | 1847.09 |
| Sudan | 16.11 | 28.83 | 46.01 | 74.45 | 138.13 | 234.44 | 345.62 | 485.68 | 597.16 | 687.16 | 721.54 | 715.12 | 653.27 |
| Suriname | 47.52 | 81.32 | 133.61 | 206.42 | 375.25 | 611.66 | 890.04 | 1138.70 | 1341.80 | 1447.22 | 1569.22 | 1532.85 | 1346.41 |
| Sweden | 1.88 | 3.41 | 5.17 | 7.70 | 13.35 | 19.72 | 28.05 | 39.78 | 52.07 | 63.70 | 77.85 | 91.73 | 112.75 |
| Switzerland | 5.26 | 8.94 | 13.95 | 21.61 | 36.51 | 56.80 | 84.65 | 121.13 | 158.66 | 196.86 | 232.42 | 273.93 | 329.75 |
| Syrian Arab Republic | 26.82 | 45.05 | 67.70 | 105.50 | 190.29 | 320.18 | 475.61 | 697.94 | 872.73 | 1010.45 | 1100.94 | 1186.13 | 1264.01 |
| Taiwan (Province of China) | 13.44 | 26.46 | 47.89 | 84.75 | 175.88 | 280.21 | 405.24 | 583.76 | 782.79 | 1038.77 | 1228.52 | 1359.82 | 1291.94 |
| Tajikistan | 16.95 | 33.02 | 53.89 | 89.43 | 205.16 | 346.35 | 573.53 | 821.30 | 1040.23 | 987.91 | 857.15 | 645.66 | 439.19 |
| Thailand | 20.35 | 39.18 | 63.79 | 99.05 | 158.65 | 242.73 | 337.95 | 487.66 | 666.92 | 821.21 | 865.41 | 859.06 | 743.35 |
| Timor-Leste | 4.31 | 7.91 | 12.81 | 21.27 | 38.80 | 65.08 | 97.15 | 137.16 | 175.19 | 213.73 | 242.94 | 252.73 | 245.46 |
| Togo | 4.96 | 8.48 | 11.24 | 27.88 | 60.31 | 103.14 | 137.85 | 203.69 | 302.48 | 311.53 | 397.24 | 434.35 | 372.89 |
| Tokelau | 17.30 | 33.71 | 58.74 | 117.44 | 179.49 | 302.51 | 400.99 | 463.12 | 583.42 | 622.28 | 684.05 | 666.27 | 684.18 |
| Tonga | 18.84 | 36.71 | 66.39 | 123.73 | 232.10 | 394.86 | 570.48 | 724.60 | 892.95 | 988.15 | 981.94 | 967.34 | 935.99 |
| Trinidad and Tobago | 40.76 | 81.22 | 142.47 | 284.30 | 602.20 | 1058.08 | 1559.83 | 2207.49 | 2635.28 | 2996.30 | 3220.07 | 3105.77 | 2706.82 |
| Tunisia | 25.71 | 46.31 | 73.03 | 116.90 | 209.47 | 349.69 | 542.69 | 767.96 | 976.55 | 1127.30 | 1221.37 | 1262.55 | 1197.05 |
| Turkey | 16.07 | 29.29 | 49.07 | 82.99 | 154.58 | 269.32 | 423.37 | 634.44 | 855.63 | 1091.23 | 1270.46 | 1287.11 | 1217.01 |
| Turkmenistan | 23.10 | 41.43 | 67.05 | 109.83 | 239.28 | 365.77 | 554.31 | 732.91 | 750.22 | 700.23 | 582.41 | 461.18 | 401.54 |
| Tuvalu | 14.74 | 30.01 | 55.04 | 102.51 | 169.19 | 263.23 | 346.61 | 416.71 | 500.25 | 552.45 | 551.67 | 564.73 | 562.77 |
| Uganda | 5.34 | 10.30 | 17.24 | 27.92 | 57.95 | 101.85 | 142.18 | 208.38 | 243.65 | 321.25 | 367.89 | 400.32 | 349.23 |
| Ukraine | 9.11 | 18.47 | 29.93 | 43.54 | 72.90 | 96.46 | 125.00 | 157.55 | 183.87 | 187.37 | 188.48 | 189.55 | 195.53 |
| United Arab Emirates | 25.51 | 46.90 | 76.10 | 136.06 | 337.62 | 689.44 | 1208.87 | 1830.74 | 2346.31 | 2496.25 | 2890.99 | 3256.39 | 3160.28 |
| United Kingdom | 18.59 | 30.17 | 43.14 | 58.13 | 79.96 | 101.95 | 126.34 | 154.07 | 184.95 | 222.15 | 250.07 | 274.65 | 308.94 |
| United Republic of Tanzania | 3.23 | 6.47 | 10.95 | 17.94 | 37.45 | 67.43 | 95.94 | 145.94 | 167.42 | 222.09 | 249.62 | 280.43 | 257.78 |
| United States of America | 3.99 | 9.42 | 18.03 | 32.36 | 60.61 | 91.40 | 131.79 | 180.62 | 214.29 | 240.90 | 254.15 | 263.25 | 260.41 |
| United States Virgin Islands | 14.96 | 26.62 | 44.95 | 77.37 | 153.78 | 250.66 | 343.61 | 450.92 | 557.98 | 642.17 | 742.04 | 800.62 | 766.82 |
| Uruguay | 2.44 | 6.37 | 11.05 | 19.67 | 38.42 | 65.57 | 108.69 | 165.06 | 209.81 | 263.76 | 297.43 | 316.30 | 290.02 |
| Uzbekistan | 19.64 | 36.26 | 57.53 | 98.18 | 259.80 | 486.32 | 819.69 | 1333.01 | 1459.48 | 1376.58 | 1139.68 | 845.68 | 574.15 |
| Vanuatu | 10.56 | 21.18 | 38.84 | 72.30 | 120.52 | 187.74 | 241.76 | 292.34 | 347.03 | 370.23 | 362.60 | 363.88 | 358.72 |
| Venezuela (Bolivarian Republic of) | 35.61 | 55.55 | 90.48 | 152.24 | 276.95 | 459.69 | 712.05 | 1001.95 | 1263.11 | 1462.77 | 1583.87 | 1568.85 | 1229.98 |
| Viet Nam | 9.18 | 17.94 | 31.58 | 56.10 | 98.48 | 154.45 | 231.69 | 390.82 | 564.52 | 736.17 | 805.56 | 843.27 | 878.71 |
| Yemen | 9.39 | 17.59 | 29.33 | 49.78 | 95.26 | 162.61 | 241.52 | 340.94 | 420.16 | 482.04 | 504.28 | 504.48 | 458.34 |
| Zambia | 7.11 | 14.14 | 24.96 | 42.05 | 88.31 | 157.46 | 210.11 | 298.88 | 335.38 | 430.22 | 475.08 | 533.32 | 516.21 |
| Zimbabwe | 7.09 | 10.18 | 17.13 | 32.62 | 70.12 | 150.58 | 249.49 | 388.28 | 434.98 | 470.55 | 475.99 | 438.65 | 435.97 |

**Supplementary Table 7. Age distribution of deaths rate for type 2 diabetes burden attributed to ambient particulate matter pollution in different countries in 2019.**

| **2019 Deahts rate** | **25-29 years** | **30-34 years** | **35-39 years** | **40-44 years** | **45-49 years** | **50-54 years** | **55-59 years** | **60-64 years** | **65-69 years** | **70-74 years** | **75-79 years** | **80-84** | **85+ years** |
| --- | --- | --- | --- | --- | --- | --- | --- | --- | --- | --- | --- | --- | --- |
| Afghanistan | 0.05 | 0.11 | 0.21 | 0.50 | 1.37 | 2.87 | 5.13 | 8.31 | 11.34 | 14.98 | 18.80 | 22.53 | 29.11 |
| Albania | 0.01 | 0.02 | 0.02 | 0.03 | 0.11 | 0.19 | 0.38 | 0.91 | 1.72 | 3.24 | 5.03 | 7.07 | 7.84 |
| Algeria | 0.04 | 0.10 | 0.20 | 0.48 | 1.32 | 2.76 | 4.80 | 8.92 | 13.40 | 19.56 | 31.74 | 48.08 | 118.12 |
| American Samoa | 0.08 | 0.24 | 0.54 | 1.31 | 2.71 | 5.26 | 8.55 | 12.23 | 18.23 | 25.14 | 34.15 | 46.40 | 71.11 |
| Andorra | 0.00 | 0.01 | 0.01 | 0.02 | 0.11 | 0.19 | 0.36 | 0.70 | 1.25 | 2.33 | 4.14 | 7.20 | 14.83 |
| Angola | 0.05 | 0.16 | 0.32 | 0.64 | 1.82 | 4.22 | 6.57 | 11.42 | 14.43 | 23.02 | 31.34 | 47.63 | 64.62 |
| Antigua and Barbuda | 0.14 | 0.29 | 0.54 | 0.93 | 2.80 | 6.28 | 11.85 | 21.97 | 31.64 | 53.14 | 82.26 | 137.46 | 185.69 |
| Argentina | 0.03 | 0.07 | 0.13 | 0.25 | 0.85 | 1.69 | 3.37 | 6.11 | 9.99 | 14.35 | 20.81 | 27.20 | 39.58 |
| Armenia | 0.05 | 0.14 | 0.23 | 0.53 | 2.05 | 4.09 | 9.45 | 18.37 | 32.15 | 50.03 | 61.96 | 66.16 | 52.93 |
| Australia | 0.00 | 0.01 | 0.02 | 0.04 | 0.12 | 0.21 | 0.35 | 0.61 | 1.06 | 1.87 | 3.47 | 6.70 | 14.62 |
| Austria | 0.01 | 0.02 | 0.04 | 0.07 | 0.25 | 0.51 | 0.98 | 2.04 | 3.71 | 6.12 | 10.69 | 21.69 | 43.16 |
| Azerbaijan | 0.06 | 0.14 | 0.21 | 0.42 | 1.83 | 3.88 | 8.26 | 15.23 | 24.48 | 29.51 | 34.98 | 34.35 | 37.22 |
| Bahamas | 0.18 | 0.45 | 0.76 | 1.21 | 2.86 | 5.41 | 7.53 | 13.53 | 22.18 | 30.14 | 46.68 | 65.11 | 90.49 |
| Bahrain | 0.28 | 0.34 | 0.59 | 1.60 | 4.53 | 10.23 | 22.93 | 48.51 | 98.99 | 200.09 | 334.59 | 482.08 | 781.93 |
| Bangladesh | 0.05 | 0.10 | 0.16 | 0.48 | 1.40 | 2.32 | 2.54 | 4.64 | 8.13 | 10.20 | 19.00 | 58.94 | 116.35 |
| Barbados | 0.23 | 0.59 | 0.87 | 1.54 | 3.82 | 7.12 | 12.54 | 21.76 | 35.73 | 60.68 | 103.76 | 154.32 | 258.69 |
| Belarus | 0.01 | 0.04 | 0.05 | 0.05 | 0.20 | 0.32 | 0.57 | 0.97 | 1.46 | 1.77 | 2.39 | 3.58 | 6.49 |
| Belgium | 0.00 | 0.02 | 0.03 | 0.05 | 0.19 | 0.34 | 0.66 | 1.21 | 2.03 | 3.51 | 5.81 | 11.33 | 26.37 |
| Belize | 0.26 | 0.63 | 0.82 | 1.52 | 4.31 | 8.96 | 15.31 | 22.56 | 33.51 | 48.82 | 64.55 | 83.58 | 109.41 |
| Benin | 0.04 | 0.08 | 0.08 | 0.31 | 0.83 | 1.71 | 2.55 | 4.48 | 7.92 | 9.90 | 16.65 | 24.00 | 32.35 |
| Bermuda | 0.01 | 0.03 | 0.05 | 0.11 | 0.34 | 0.71 | 1.19 | 1.95 | 3.12 | 5.02 | 7.96 | 12.70 | 21.40 |
| Bhutan | 0.03 | 0.08 | 0.12 | 0.34 | 0.99 | 1.98 | 3.52 | 7.03 | 10.81 | 17.74 | 31.64 | 49.91 | 78.01 |
| Bolivia (Plurinational State of) | 0.07 | 0.16 | 0.31 | 0.78 | 2.23 | 4.84 | 9.53 | 16.50 | 26.40 | 41.41 | 59.91 | 75.42 | 86.30 |
| Bosnia and Herzegovina | 0.08 | 0.16 | 0.22 | 0.40 | 1.22 | 2.52 | 5.30 | 11.35 | 22.45 | 44.78 | 72.44 | 105.19 | 115.26 |
| Botswana | 0.12 | 0.21 | 0.53 | 1.54 | 4.11 | 9.26 | 17.92 | 39.23 | 56.80 | 81.94 | 110.96 | 125.75 | 169.27 |
| Brazil | 0.05 | 0.12 | 0.21 | 0.42 | 1.00 | 1.94 | 3.50 | 6.11 | 9.73 | 15.12 | 22.74 | 32.22 | 50.23 |
| Brunei Darussalam | 0.07 | 0.17 | 0.31 | 0.64 | 1.71 | 2.67 | 5.20 | 9.21 | 17.25 | 28.44 | 43.95 | 63.90 | 83.89 |
| Bulgaria | 0.04 | 0.09 | 0.11 | 0.16 | 0.90 | 1.77 | 3.08 | 5.92 | 9.29 | 14.69 | 22.52 | 25.99 | 28.22 |
| Burkina Faso | 0.03 | 0.07 | 0.07 | 0.29 | 0.74 | 1.48 | 2.25 | 3.40 | 5.30 | 5.83 | 10.63 | 17.08 | 24.80 |
| Burundi | 0.01 | 0.04 | 0.07 | 0.15 | 0.44 | 0.98 | 1.57 | 2.82 | 3.59 | 5.78 | 8.04 | 11.58 | 15.47 |
| Cabo Verde | 0.08 | 0.14 | 0.16 | 0.69 | 2.14 | 4.04 | 6.48 | 11.10 | 21.48 | 29.83 | 54.56 | 92.95 | 138.19 |
| Cambodia | 0.04 | 0.10 | 0.16 | 0.33 | 0.79 | 1.59 | 2.77 | 4.50 | 6.49 | 9.25 | 13.01 | 17.07 | 23.98 |
| Cameroon | 0.15 | 0.28 | 0.31 | 1.18 | 2.88 | 5.74 | 8.48 | 14.97 | 26.49 | 32.66 | 53.62 | 76.90 | 98.50 |
| Canada | 0.00 | 0.01 | 0.02 | 0.03 | 0.15 | 0.28 | 0.48 | 0.92 | 1.55 | 2.44 | 3.99 | 6.92 | 14.96 |
| Central African Republic | 0.03 | 0.08 | 0.18 | 0.41 | 1.28 | 2.81 | 4.16 | 6.21 | 7.18 | 10.09 | 12.47 | 16.13 | 19.50 |
| Chad | 0.03 | 0.05 | 0.06 | 0.21 | 0.56 | 1.18 | 1.74 | 3.16 | 5.65 | 6.92 | 11.06 | 15.14 | 18.98 |
| Chile | 0.02 | 0.05 | 0.10 | 0.22 | 0.61 | 1.34 | 2.61 | 5.17 | 9.61 | 15.35 | 26.49 | 41.34 | 79.24 |
| China | 0.03 | 0.07 | 0.14 | 0.26 | 0.55 | 1.09 | 2.01 | 3.60 | 6.39 | 11.63 | 17.52 | 25.30 | 39.12 |
| Colombia | 0.07 | 0.15 | 0.22 | 0.38 | 0.77 | 1.50 | 2.78 | 5.07 | 7.85 | 12.52 | 19.03 | 25.83 | 39.24 |
| Comoros | 0.02 | 0.04 | 0.09 | 0.18 | 0.51 | 1.10 | 1.81 | 3.45 | 4.52 | 7.77 | 11.25 | 17.39 | 24.59 |
| Congo | 0.08 | 0.24 | 0.55 | 1.14 | 3.19 | 7.13 | 11.64 | 19.12 | 24.64 | 38.71 | 54.88 | 79.54 | 119.72 |
| Cook Islands | 0.08 | 0.24 | 0.56 | 1.42 | 2.84 | 5.48 | 8.68 | 12.80 | 19.50 | 27.61 | 37.39 | 54.24 | 89.57 |
| Costa Rica | 0.05 | 0.10 | 0.15 | 0.29 | 0.68 | 1.21 | 1.97 | 3.74 | 5.81 | 9.30 | 15.98 | 22.78 | 37.85 |
| Croatia | 0.01 | 0.03 | 0.04 | 0.07 | 0.30 | 0.68 | 1.55 | 3.10 | 5.79 | 10.85 | 17.94 | 30.85 | 46.23 |
| Cuba | 0.04 | 0.09 | 0.14 | 0.23 | 0.60 | 1.23 | 2.24 | 3.82 | 6.29 | 9.57 | 14.95 | 18.70 | 26.30 |
| Cyprus | 0.01 | 0.02 | 0.05 | 0.08 | 0.23 | 0.59 | 1.49 | 3.66 | 8.25 | 16.70 | 33.27 | 79.33 | 151.00 |
| Czechia | 0.01 | 0.02 | 0.04 | 0.07 | 0.39 | 0.81 | 1.70 | 3.63 | 6.73 | 12.33 | 20.72 | 37.97 | 65.96 |
| Côte d'Ivoire | 0.07 | 0.13 | 0.13 | 0.55 | 1.42 | 2.84 | 4.22 | 7.52 | 13.36 | 16.46 | 28.16 | 41.38 | 53.59 |
| Democratic People's Republic of Korea | 0.04 | 0.08 | 0.13 | 0.25 | 0.75 | 1.39 | 2.45 | 4.04 | 6.12 | 8.30 | 11.02 | 11.63 | 13.93 |
| Democratic Republic of the Congo | 0.02 | 0.07 | 0.14 | 0.30 | 0.85 | 1.96 | 2.93 | 4.82 | 5.91 | 9.20 | 12.56 | 18.70 | 26.13 |
| Denmark | 0.01 | 0.03 | 0.04 | 0.07 | 0.31 | 0.65 | 1.02 | 1.82 | 2.87 | 4.85 | 8.36 | 14.51 | 29.77 |
| Djibouti | 0.08 | 0.23 | 0.47 | 0.99 | 2.82 | 6.30 | 10.21 | 19.20 | 25.29 | 42.96 | 62.09 | 93.82 | 128.15 |
| Dominica | 0.26 | 0.54 | 0.94 | 1.58 | 3.76 | 6.93 | 11.65 | 19.71 | 35.35 | 54.16 | 83.83 | 123.76 | 181.35 |
| Dominican Republic | 0.24 | 0.47 | 0.71 | 1.30 | 2.62 | 5.05 | 7.33 | 12.80 | 16.60 | 24.00 | 29.24 | 38.26 | 55.78 |
| Ecuador | 0.14 | 0.31 | 0.54 | 1.02 | 2.47 | 4.72 | 8.68 | 13.69 | 19.97 | 29.17 | 44.81 | 65.10 | 127.32 |
| Egypt | 0.14 | 0.31 | 0.53 | 1.20 | 4.07 | 9.92 | 17.44 | 30.13 | 41.61 | 50.09 | 60.03 | 78.65 | 117.69 |
| El Salvador | 0.16 | 0.43 | 0.75 | 1.41 | 3.56 | 6.16 | 10.91 | 16.92 | 23.73 | 32.29 | 45.11 | 54.98 | 67.01 |
| Equatorial Guinea | 0.14 | 0.40 | 0.79 | 1.55 | 4.16 | 9.20 | 15.15 | 26.83 | 37.54 | 61.78 | 95.97 | 148.59 | 223.71 |
| Eritrea | 0.04 | 0.12 | 0.27 | 0.57 | 1.69 | 3.48 | 5.59 | 9.45 | 11.64 | 17.86 | 24.51 | 34.16 | 45.97 |
| Estonia | 0.00 | 0.00 | 0.01 | 0.02 | 0.12 | 0.19 | 0.28 | 0.51 | 0.81 | 1.19 | 1.61 | 1.96 | 2.36 |
| Eswatini | 0.18 | 0.30 | 0.75 | 2.19 | 5.78 | 11.95 | 21.28 | 43.56 | 61.59 | 84.05 | 113.09 | 130.84 | 175.34 |
| Ethiopia | 0.01 | 0.04 | 0.08 | 0.16 | 0.49 | 1.10 | 1.85 | 3.46 | 4.73 | 8.46 | 12.55 | 18.37 | 25.29 |
| Fiji | 0.28 | 0.72 | 1.82 | 4.75 | 10.51 | 22.24 | 37.15 | 58.71 | 84.24 | 116.27 | 138.55 | 192.07 | 284.34 |
| Finland | 0.00 | 0.01 | 0.01 | 0.01 | 0.06 | 0.08 | 0.12 | 0.19 | 0.30 | 0.48 | 0.79 | 1.41 | 2.58 |
| France | 0.01 | 0.02 | 0.03 | 0.05 | 0.21 | 0.37 | 0.71 | 1.25 | 2.09 | 3.52 | 6.08 | 11.51 | 28.51 |
| Gabon | 0.17 | 0.50 | 1.06 | 2.18 | 6.14 | 14.01 | 23.01 | 39.55 | 52.27 | 81.09 | 112.50 | 161.09 | 243.41 |
| Gambia | 0.04 | 0.08 | 0.09 | 0.37 | 1.04 | 2.23 | 3.45 | 6.08 | 11.14 | 13.40 | 22.32 | 32.23 | 44.26 |
| Georgia | 0.02 | 0.06 | 0.11 | 0.22 | 1.17 | 2.50 | 4.66 | 8.81 | 13.55 | 20.28 | 26.60 | 25.20 | 15.75 |
| Germany | 0.00 | 0.02 | 0.03 | 0.07 | 0.28 | 0.53 | 0.93 | 1.70 | 2.72 | 4.70 | 8.61 | 16.92 | 39.89 |
| Ghana | 0.12 | 0.27 | 0.28 | 1.21 | 2.85 | 5.88 | 9.07 | 15.45 | 27.86 | 32.03 | 52.77 | 70.73 | 93.09 |
| Greece | 0.00 | 0.02 | 0.03 | 0.06 | 0.18 | 0.40 | 0.83 | 1.47 | 2.64 | 4.17 | 7.14 | 9.82 | 13.46 |
| Greenland | 0.00 | 0.03 | 0.04 | 0.08 | 0.20 | 0.37 | 0.64 | 1.05 | 1.59 | 2.45 | 3.45 | 4.72 | 6.34 |
| Grenada | 0.24 | 0.66 | 1.13 | 1.91 | 4.72 | 8.82 | 15.72 | 29.19 | 53.46 | 77.29 | 110.89 | 157.85 | 229.65 |
| Guam | 0.03 | 0.11 | 0.28 | 0.60 | 1.13 | 2.52 | 3.49 | 4.99 | 6.98 | 8.15 | 11.90 | 14.95 | 25.75 |
| Guatemala | 0.18 | 0.52 | 1.07 | 2.05 | 4.48 | 7.87 | 12.02 | 17.68 | 24.52 | 29.44 | 35.90 | 52.39 | 92.91 |
| Guinea | 0.04 | 0.07 | 0.09 | 0.29 | 0.75 | 1.61 | 2.39 | 4.33 | 7.56 | 9.22 | 14.50 | 20.46 | 26.32 |
| Guinea-Bissau | 0.06 | 0.11 | 0.16 | 0.55 | 1.48 | 2.75 | 4.08 | 6.77 | 11.38 | 13.14 | 19.86 | 27.70 | 35.17 |
| Guyana | 0.43 | 0.97 | 1.53 | 3.26 | 8.35 | 16.86 | 28.54 | 45.05 | 62.54 | 84.80 | 110.71 | 134.65 | 191.75 |
| Haiti | 0.04 | 0.10 | 0.17 | 0.36 | 1.06 | 2.14 | 3.67 | 6.07 | 8.91 | 12.79 | 17.06 | 22.27 | 24.58 |
| Honduras | 0.04 | 0.08 | 0.15 | 0.32 | 0.79 | 1.54 | 2.59 | 4.06 | 5.91 | 8.43 | 11.51 | 13.43 | 16.64 |
| Hungary | 0.01 | 0.04 | 0.06 | 0.11 | 0.56 | 1.19 | 2.32 | 3.92 | 6.34 | 10.17 | 17.23 | 24.41 | 29.84 |
| Iceland | 0.00 | 0.00 | 0.00 | 0.01 | 0.03 | 0.05 | 0.10 | 0.20 | 0.34 | 0.62 | 1.15 | 2.03 | 3.69 |
| India | 0.06 | 0.16 | 0.24 | 0.63 | 1.66 | 3.67 | 6.35 | 11.48 | 16.43 | 23.97 | 39.10 | 62.53 | 85.44 |
| Indonesia | 0.16 | 0.40 | 0.85 | 1.94 | 4.55 | 8.18 | 13.37 | 19.19 | 24.01 | 29.88 | 40.13 | 49.01 | 68.94 |
| Iran (Islamic Republic of) | 0.05 | 0.10 | 0.19 | 0.42 | 1.25 | 3.13 | 6.46 | 12.45 | 20.08 | 31.51 | 51.97 | 69.84 | 96.29 |
| Iraq | 0.26 | 0.47 | 0.89 | 1.80 | 4.86 | 9.69 | 18.14 | 30.89 | 45.61 | 67.58 | 94.00 | 120.67 | 186.99 |
| Ireland | 0.00 | 0.01 | 0.01 | 0.02 | 0.06 | 0.11 | 0.21 | 0.39 | 0.77 | 1.71 | 3.73 | 6.90 | 14.78 |
| Israel | 0.01 | 0.04 | 0.08 | 0.18 | 0.69 | 1.69 | 3.13 | 5.49 | 9.86 | 17.50 | 32.59 | 55.27 | 124.22 |
| Italy | 0.00 | 0.02 | 0.03 | 0.07 | 0.27 | 0.63 | 1.27 | 2.49 | 4.58 | 8.15 | 14.64 | 26.45 | 58.46 |
| Jamaica | 0.13 | 0.37 | 0.56 | 1.26 | 3.29 | 6.83 | 12.39 | 21.46 | 36.42 | 61.26 | 95.13 | 129.45 | 197.16 |
| Japan | 0.00 | 0.01 | 0.02 | 0.04 | 0.14 | 0.22 | 0.34 | 0.51 | 0.76 | 1.21 | 1.91 | 3.18 | 7.49 |
| Jordan | 0.06 | 0.13 | 0.23 | 0.60 | 1.90 | 4.77 | 9.56 | 19.13 | 35.55 | 55.22 | 85.80 | 124.00 | 195.16 |
| Kazakhstan | 0.04 | 0.07 | 0.11 | 0.17 | 0.74 | 1.46 | 3.49 | 6.99 | 12.25 | 17.73 | 24.81 | 29.37 | 23.07 |
| Kenya | 0.02 | 0.08 | 0.17 | 0.33 | 0.94 | 2.03 | 3.12 | 5.25 | 6.76 | 11.45 | 16.15 | 23.44 | 31.70 |
| Kiribati | 0.17 | 0.40 | 0.90 | 2.47 | 5.15 | 8.79 | 12.53 | 16.04 | 21.68 | 26.11 | 24.39 | 29.14 | 52.86 |
| Kuwait | 0.04 | 0.10 | 0.14 | 0.29 | 0.83 | 1.88 | 3.78 | 7.60 | 14.49 | 28.65 | 44.63 | 70.99 | 127.01 |
| Kyrgyzstan | 0.03 | 0.05 | 0.09 | 0.18 | 0.55 | 1.03 | 2.21 | 3.56 | 5.39 | 5.09 | 5.39 | 4.50 | 6.63 |
| Lao People's Democratic Republic | 0.06 | 0.13 | 0.21 | 0.42 | 0.90 | 1.73 | 2.98 | 4.88 | 6.89 | 9.66 | 13.54 | 18.31 | 25.99 |
| Latvia | 0.04 | 0.10 | 0.13 | 0.17 | 0.47 | 0.84 | 1.19 | 2.06 | 3.41 | 5.31 | 7.14 | 10.39 | 14.11 |
| Lebanon | 0.04 | 0.08 | 0.15 | 0.35 | 1.05 | 2.43 | 3.94 | 6.58 | 14.41 | 24.52 | 34.70 | 43.09 | 66.50 |
| Lesotho | 0.11 | 0.18 | 0.46 | 1.41 | 3.89 | 8.43 | 15.58 | 32.90 | 44.65 | 59.01 | 76.34 | 84.00 | 111.48 |
| Liberia | 0.05 | 0.08 | 0.09 | 0.31 | 0.84 | 1.70 | 2.51 | 4.57 | 7.98 | 9.87 | 16.75 | 24.93 | 35.19 |
| Libya | 0.04 | 0.12 | 0.24 | 0.57 | 1.83 | 3.69 | 6.70 | 13.22 | 20.48 | 26.94 | 38.45 | 42.29 | 65.19 |
| Lithuania | 0.01 | 0.03 | 0.05 | 0.05 | 0.31 | 0.39 | 0.58 | 1.10 | 1.66 | 2.26 | 2.87 | 3.59 | 4.50 |
| Luxembourg | 0.00 | 0.01 | 0.02 | 0.03 | 0.11 | 0.21 | 0.40 | 0.84 | 1.44 | 2.69 | 5.02 | 8.90 | 17.86 |
| Madagascar | 0.01 | 0.03 | 0.07 | 0.13 | 0.36 | 0.77 | 1.27 | 2.31 | 2.94 | 4.73 | 6.45 | 9.62 | 13.87 |
| Malawi | 0.01 | 0.04 | 0.07 | 0.15 | 0.47 | 1.06 | 1.65 | 2.92 | 3.87 | 6.45 | 9.08 | 12.75 | 16.22 |
| Malaysia | 0.05 | 0.13 | 0.21 | 0.36 | 0.93 | 1.90 | 3.41 | 5.82 | 8.80 | 13.01 | 17.05 | 21.00 | 32.27 |
| Maldives | 0.02 | 0.06 | 0.10 | 0.19 | 0.46 | 0.94 | 1.75 | 3.36 | 5.51 | 8.87 | 13.22 | 21.29 | 35.55 |
| Mali | 0.03 | 0.05 | 0.07 | 0.22 | 0.58 | 1.21 | 1.77 | 3.25 | 5.82 | 7.53 | 12.04 | 17.57 | 22.02 |
| Malta | 0.02 | 0.05 | 0.07 | 0.12 | 0.38 | 0.65 | 1.26 | 2.40 | 4.30 | 7.55 | 13.80 | 24.37 | 45.36 |
| Marshall Islands | 0.12 | 0.33 | 0.78 | 1.88 | 3.73 | 7.05 | 10.60 | 14.80 | 20.71 | 27.33 | 33.61 | 44.82 | 63.43 |
| Mauritania | 0.08 | 0.13 | 0.19 | 0.59 | 1.56 | 3.36 | 5.22 | 10.22 | 18.68 | 24.46 | 43.40 | 66.65 | 95.08 |
| Mauritius | 0.22 | 0.65 | 1.43 | 2.69 | 7.89 | 14.22 | 25.23 | 44.85 | 67.73 | 97.44 | 131.12 | 154.23 | 211.39 |
| Mexico | 0.19 | 0.51 | 1.09 | 2.39 | 5.25 | 10.04 | 17.15 | 27.00 | 40.21 | 55.88 | 79.62 | 107.70 | 149.83 |
| Micronesia (Federated States of) | 0.27 | 0.76 | 1.34 | 3.07 | 7.43 | 14.47 | 21.49 | 29.14 | 38.96 | 52.17 | 65.23 | 85.42 | 121.44 |
| Monaco | 0.01 | 0.02 | 0.02 | 0.02 | 0.09 | 0.16 | 0.29 | 0.54 | 0.95 | 1.77 | 3.21 | 5.71 | 11.16 |
| Mongolia | 0.04 | 0.12 | 0.17 | 0.28 | 0.85 | 1.40 | 1.76 | 2.36 | 3.76 | 4.33 | 4.75 | 5.44 | 7.68 |
| Montenegro | 0.02 | 0.05 | 0.07 | 0.10 | 0.56 | 1.21 | 2.57 | 5.11 | 9.10 | 14.20 | 21.82 | 31.05 | 38.36 |
| Morocco | 0.04 | 0.09 | 0.19 | 0.51 | 1.69 | 3.95 | 7.64 | 14.09 | 21.60 | 32.17 | 43.45 | 55.46 | 77.06 |
| Mozambique | 0.01 | 0.04 | 0.09 | 0.19 | 0.54 | 1.15 | 1.80 | 3.05 | 3.65 | 5.15 | 6.67 | 9.89 | 15.28 |
| Myanmar | 0.14 | 0.42 | 0.64 | 1.03 | 1.98 | 4.58 | 7.23 | 11.41 | 16.57 | 22.47 | 34.64 | 43.28 | 70.93 |
| Namibia | 0.04 | 0.08 | 0.21 | 0.65 | 1.97 | 4.70 | 9.50 | 21.83 | 32.90 | 49.13 | 69.27 | 79.90 | 107.78 |
| Nauru | 0.16 | 0.43 | 0.98 | 2.32 | 4.42 | 8.10 | 12.03 | 16.83 | 23.58 | 32.10 | 39.44 | 49.86 | 73.54 |
| Nepal | 0.03 | 0.08 | 0.15 | 0.16 | 0.71 | 1.37 | 2.92 | 4.13 | 5.58 | 9.15 | 18.92 | 36.23 | 66.38 |
| Netherlands | 0.00 | 0.02 | 0.03 | 0.05 | 0.25 | 0.45 | 0.80 | 1.37 | 2.33 | 4.13 | 7.58 | 15.12 | 39.28 |
| New Zealand | 0.00 | 0.02 | 0.02 | 0.04 | 0.11 | 0.22 | 0.38 | 0.60 | 0.88 | 1.40 | 2.55 | 4.07 | 7.57 |
| Nicaragua | 0.05 | 0.10 | 0.21 | 0.52 | 1.43 | 3.02 | 5.76 | 10.30 | 16.22 | 24.56 | 35.22 | 43.13 | 55.94 |
| Niger | 0.02 | 0.04 | 0.04 | 0.16 | 0.42 | 0.90 | 1.33 | 2.46 | 4.41 | 5.59 | 9.31 | 13.16 | 17.40 |
| Nigeria | 0.06 | 0.14 | 0.14 | 0.53 | 1.38 | 2.90 | 4.53 | 8.61 | 15.34 | 22.95 | 44.44 | 66.69 | 97.85 |
| Niue | 0.12 | 0.29 | 0.65 | 1.63 | 3.41 | 7.01 | 11.38 | 17.16 | 25.74 | 36.04 | 46.58 | 61.63 | 92.45 |
| North Macedonia | 0.02 | 0.05 | 0.12 | 0.24 | 1.20 | 2.69 | 6.01 | 12.06 | 23.83 | 42.34 | 77.15 | 101.06 | 114.80 |
| Northern Mariana Islands | 0.05 | 0.16 | 0.39 | 0.96 | 2.34 | 4.38 | 7.26 | 10.45 | 15.82 | 23.46 | 31.34 | 41.93 | 64.37 |
| Norway | 0.00 | 0.01 | 0.01 | 0.02 | 0.08 | 0.13 | 0.20 | 0.35 | 0.58 | 1.00 | 2.01 | 3.55 | 9.40 |
| Oman | 0.02 | 0.06 | 0.18 | 0.52 | 2.47 | 6.47 | 13.48 | 28.54 | 58.10 | 89.05 | 136.42 | 217.04 | 294.78 |
| Pakistan | 0.19 | 0.39 | 0.57 | 1.47 | 3.09 | 5.84 | 9.43 | 17.80 | 24.52 | 36.61 | 57.36 | 84.10 | 123.79 |
| Palau | 0.14 | 0.41 | 0.89 | 2.21 | 4.21 | 7.65 | 11.43 | 16.25 | 23.60 | 31.75 | 40.98 | 57.54 | 85.86 |
| Palestine | 0.12 | 0.18 | 0.38 | 0.87 | 2.87 | 6.90 | 17.72 | 31.03 | 58.10 | 100.90 | 158.47 | 212.61 | 302.97 |
| Panama | 0.05 | 0.12 | 0.21 | 0.47 | 1.18 | 2.63 | 4.53 | 7.84 | 12.11 | 18.66 | 29.40 | 42.60 | 63.71 |
| Papua New Guinea | 0.09 | 0.24 | 0.48 | 1.07 | 1.88 | 3.59 | 5.79 | 8.63 | 12.30 | 15.20 | 19.41 | 23.79 | 31.20 |
| Paraguay | 0.06 | 0.13 | 0.23 | 0.55 | 1.41 | 2.99 | 5.28 | 9.45 | 14.38 | 22.10 | 32.35 | 42.98 | 56.71 |
| Peru | 0.06 | 0.13 | 0.23 | 0.48 | 1.15 | 2.25 | 4.26 | 6.82 | 10.50 | 15.52 | 22.69 | 28.13 | 39.59 |
| Philippines | 0.14 | 0.31 | 0.53 | 1.02 | 2.20 | 4.17 | 7.12 | 11.09 | 16.37 | 21.46 | 28.07 | 36.41 | 51.86 |
| Poland | 0.02 | 0.05 | 0.09 | 0.19 | 0.80 | 1.37 | 2.36 | 4.13 | 6.84 | 10.98 | 17.10 | 25.83 | 39.84 |
| Portugal | 0.01 | 0.02 | 0.03 | 0.06 | 0.16 | 0.33 | 0.60 | 1.28 | 2.42 | 5.03 | 9.40 | 17.39 | 33.17 |
| Puerto Rico | 0.02 | 0.07 | 0.15 | 0.28 | 0.92 | 1.74 | 2.86 | 4.73 | 7.13 | 11.26 | 17.22 | 26.20 | 43.22 |
| Qatar | 0.04 | 0.09 | 0.28 | 0.51 | 1.69 | 4.68 | 10.89 | 24.69 | 56.07 | 138.10 | 295.65 | 647.15 | 1058.27 |
| Republic of Korea | 0.03 | 0.09 | 0.16 | 0.33 | 0.90 | 1.65 | 2.69 | 4.46 | 7.94 | 15.84 | 30.15 | 51.69 | 91.63 |
| Republic of Moldova | 0.02 | 0.04 | 0.07 | 0.09 | 0.32 | 0.56 | 1.12 | 2.08 | 3.61 | 3.96 | 4.00 | 3.39 | 5.65 |
| Romania | 0.02 | 0.04 | 0.05 | 0.09 | 0.35 | 0.68 | 1.38 | 2.30 | 3.92 | 5.77 | 7.88 | 10.00 | 9.23 |
| Russian Federation | 0.01 | 0.02 | 0.03 | 0.05 | 0.24 | 0.45 | 0.93 | 2.04 | 3.84 | 5.71 | 8.44 | 9.93 | 9.85 |
| Rwanda | 0.02 | 0.07 | 0.13 | 0.25 | 0.75 | 1.67 | 2.76 | 5.07 | 6.85 | 11.50 | 16.85 | 26.08 | 35.66 |
| Saint Kitts and Nevis | 0.04 | 0.10 | 0.19 | 0.45 | 1.52 | 3.54 | 6.42 | 10.92 | 17.64 | 26.12 | 37.47 | 50.79 | 67.82 |
| Saint Lucia | 0.30 | 0.69 | 1.07 | 2.12 | 4.86 | 8.16 | 14.51 | 24.12 | 37.43 | 57.21 | 82.72 | 127.73 | 238.43 |
| Saint Vincent and the Grenadines | 0.40 | 0.83 | 1.40 | 2.76 | 7.36 | 13.43 | 19.64 | 30.63 | 49.82 | 73.57 | 114.72 | 174.90 | 247.80 |
| Samoa | 0.08 | 0.22 | 0.51 | 1.29 | 2.61 | 5.03 | 7.93 | 11.56 | 17.14 | 23.00 | 29.73 | 39.86 | 59.74 |
| San Marino | 0.01 | 0.01 | 0.02 | 0.02 | 0.10 | 0.19 | 0.36 | 0.74 | 1.36 | 2.58 | 4.77 | 8.66 | 18.68 |
| Sao Tome and Principe | 0.01 | 0.03 | 0.02 | 0.14 | 0.60 | 1.26 | 2.03 | 3.31 | 6.39 | 7.87 | 12.30 | 16.78 | 25.40 |
| Saudi Arabia | 0.11 | 0.23 | 0.43 | 0.95 | 2.31 | 4.61 | 8.57 | 12.69 | 19.16 | 28.29 | 39.40 | 55.23 | 90.58 |
| Senegal | 0.05 | 0.10 | 0.11 | 0.42 | 1.12 | 2.35 | 3.62 | 6.65 | 12.04 | 15.38 | 25.55 | 37.97 | 52.18 |
| Serbia | 0.01 | 0.05 | 0.09 | 0.18 | 0.87 | 2.01 | 3.84 | 7.57 | 14.96 | 22.02 | 38.24 | 66.29 | 82.70 |
| Seychelles | 0.07 | 0.18 | 0.31 | 0.70 | 1.64 | 3.01 | 5.01 | 8.74 | 13.88 | 19.12 | 32.99 | 45.01 | 69.74 |
| Sierra Leone | 0.04 | 0.06 | 0.08 | 0.27 | 0.69 | 1.43 | 2.06 | 3.79 | 6.61 | 8.14 | 13.68 | 20.33 | 26.15 |
| Singapore | 0.01 | 0.02 | 0.02 | 0.05 | 0.14 | 0.24 | 0.42 | 0.73 | 1.25 | 2.21 | 3.69 | 5.94 | 11.06 |
| Slovakia | 0.00 | 0.02 | 0.03 | 0.07 | 0.43 | 0.86 | 1.65 | 3.48 | 5.86 | 9.41 | 14.13 | 20.42 | 22.67 |
| Slovenia | 0.00 | 0.02 | 0.03 | 0.05 | 0.19 | 0.42 | 0.83 | 1.93 | 3.53 | 6.29 | 12.45 | 18.59 | 26.00 |
| Solomon Islands | 0.16 | 0.42 | 0.87 | 1.82 | 3.10 | 5.05 | 7.05 | 8.95 | 11.45 | 13.96 | 16.80 | 17.97 | 22.78 |
| Somalia | 0.01 | 0.02 | 0.04 | 0.08 | 0.23 | 0.50 | 0.77 | 1.30 | 1.60 | 2.44 | 3.22 | 4.33 | 5.44 |
| South Africa | 0.21 | 0.36 | 0.64 | 1.38 | 3.21 | 7.69 | 15.75 | 36.90 | 56.14 | 73.96 | 98.58 | 144.44 | 212.29 |
| South Sudan | 0.02 | 0.04 | 0.10 | 0.18 | 0.55 | 1.26 | 2.12 | 4.02 | 5.51 | 9.48 | 14.13 | 20.98 | 28.81 |
| Spain | 0.00 | 0.01 | 0.01 | 0.02 | 0.09 | 0.19 | 0.39 | 0.72 | 1.32 | 2.70 | 5.67 | 11.53 | 31.14 |
| Sri Lanka | 0.11 | 0.23 | 0.41 | 0.83 | 2.00 | 4.10 | 6.94 | 12.73 | 21.97 | 38.44 | 55.77 | 90.16 | 163.64 |
| Sudan | 0.03 | 0.07 | 0.13 | 0.29 | 0.93 | 2.11 | 3.77 | 6.94 | 10.47 | 15.52 | 20.92 | 26.84 | 34.39 |
| Suriname | 0.24 | 0.49 | 0.93 | 1.51 | 3.53 | 7.12 | 13.41 | 21.22 | 30.88 | 41.01 | 58.53 | 73.13 | 91.08 |
| Sweden | 0.00 | 0.01 | 0.01 | 0.02 | 0.07 | 0.12 | 0.19 | 0.36 | 0.61 | 1.01 | 1.99 | 3.78 | 9.55 |
| Switzerland | 0.00 | 0.01 | 0.02 | 0.03 | 0.11 | 0.20 | 0.38 | 0.76 | 1.33 | 2.36 | 4.56 | 9.56 | 24.49 |
| Syrian Arab Republic | 0.09 | 0.15 | 0.20 | 0.37 | 1.09 | 2.44 | 4.30 | 8.76 | 13.45 | 20.14 | 29.93 | 47.05 | 78.32 |
| Taiwan (Province of China) | 0.02 | 0.10 | 0.22 | 0.46 | 1.68 | 3.15 | 5.29 | 9.69 | 16.51 | 30.40 | 49.84 | 77.62 | 116.86 |
| Tajikistan | 0.07 | 0.18 | 0.28 | 0.53 | 2.29 | 4.77 | 10.75 | 19.75 | 31.61 | 35.48 | 36.54 | 30.44 | 20.78 |
| Thailand | 0.13 | 0.31 | 0.54 | 0.91 | 1.71 | 3.08 | 4.86 | 8.73 | 14.55 | 21.77 | 27.75 | 34.84 | 42.18 |
| Timor-Leste | 0.02 | 0.05 | 0.09 | 0.17 | 0.42 | 0.90 | 1.61 | 2.74 | 4.00 | 5.81 | 8.38 | 11.44 | 16.37 |
| Togo | 0.04 | 0.08 | 0.09 | 0.37 | 1.03 | 2.06 | 3.02 | 5.26 | 9.58 | 11.29 | 19.18 | 27.89 | 35.09 |
| Tokelau | 0.10 | 0.26 | 0.55 | 1.53 | 2.72 | 5.74 | 8.88 | 11.83 | 18.25 | 23.66 | 33.93 | 43.50 | 72.86 |
| Tonga | 0.09 | 0.25 | 0.58 | 1.44 | 3.51 | 7.42 | 12.88 | 19.42 | 29.00 | 39.39 | 49.52 | 64.88 | 97.57 |
| Trinidad and Tobago | 0.19 | 0.55 | 1.06 | 2.85 | 7.88 | 16.83 | 30.07 | 53.49 | 77.85 | 111.55 | 155.25 | 194.45 | 255.44 |
| Tunisia | 0.03 | 0.08 | 0.14 | 0.32 | 0.94 | 2.05 | 4.24 | 7.88 | 13.19 | 20.37 | 30.74 | 44.43 | 62.31 |
| Turkey | 0.03 | 0.07 | 0.16 | 0.39 | 1.11 | 2.58 | 5.07 | 9.97 | 17.54 | 30.69 | 49.48 | 67.11 | 96.66 |
| Turkmenistan | 0.08 | 0.18 | 0.29 | 0.60 | 2.69 | 4.96 | 9.95 | 16.39 | 18.92 | 19.76 | 17.09 | 12.17 | 11.79 |
| Tuvalu | 0.09 | 0.26 | 0.60 | 1.41 | 2.79 | 5.18 | 7.96 | 11.19 | 16.21 | 22.05 | 27.86 | 38.03 | 56.05 |
| Uganda | 0.02 | 0.07 | 0.14 | 0.28 | 0.81 | 1.78 | 2.86 | 5.13 | 6.91 | 11.76 | 17.37 | 25.36 | 32.59 |
| Ukraine | 0.03 | 0.06 | 0.09 | 0.12 | 0.43 | 0.59 | 0.91 | 1.44 | 2.04 | 2.07 | 2.38 | 3.07 | 5.62 |
| United Arab Emirates | 0.02 | 0.10 | 0.21 | 0.67 | 3.32 | 8.88 | 18.79 | 34.80 | 56.00 | 72.86 | 118.49 | 188.69 | 262.51 |
| United Kingdom | 0.01 | 0.02 | 0.03 | 0.05 | 0.16 | 0.22 | 0.33 | 0.54 | 0.84 | 1.53 | 2.84 | 5.59 | 14.49 |
| United Republic of Tanzania | 0.02 | 0.05 | 0.11 | 0.21 | 0.58 | 1.28 | 2.11 | 3.91 | 5.12 | 8.60 | 12.28 | 18.52 | 25.53 |
| United States of America | 0.01 | 0.05 | 0.08 | 0.14 | 0.46 | 0.80 | 1.28 | 2.04 | 2.84 | 4.21 | 6.05 | 9.13 | 15.70 |
| United States Virgin Islands | 0.04 | 0.11 | 0.23 | 0.53 | 1.61 | 3.22 | 5.19 | 8.38 | 12.96 | 18.82 | 29.45 | 43.32 | 62.68 |
| Uruguay | 0.02 | 0.05 | 0.07 | 0.13 | 0.34 | 0.72 | 1.53 | 2.94 | 4.53 | 7.63 | 11.64 | 17.26 | 24.41 |
| Uzbekistan | 0.07 | 0.16 | 0.22 | 0.45 | 3.01 | 7.38 | 16.42 | 35.12 | 45.65 | 51.43 | 50.15 | 41.59 | 26.39 |
| Vanuatu | 0.05 | 0.15 | 0.35 | 0.89 | 1.83 | 3.46 | 5.18 | 7.34 | 10.47 | 13.52 | 16.52 | 22.05 | 31.27 |
| Venezuela (Bolivarian Republic of) | 0.19 | 0.31 | 0.58 | 1.18 | 2.82 | 5.81 | 11.31 | 19.61 | 30.90 | 45.86 | 65.43 | 85.17 | 93.82 |
| Viet Nam | 0.05 | 0.13 | 0.26 | 0.58 | 1.27 | 2.31 | 4.01 | 8.74 | 15.11 | 24.13 | 33.14 | 46.35 | 75.71 |
| Yemen | 0.02 | 0.05 | 0.10 | 0.26 | 0.82 | 1.83 | 3.27 | 5.75 | 8.44 | 11.99 | 15.76 | 20.44 | 25.72 |
| Zambia | 0.04 | 0.12 | 0.26 | 0.52 | 1.43 | 3.09 | 4.67 | 7.95 | 10.14 | 16.35 | 22.83 | 34.59 | 49.43 |
| Zimbabwe | 0.04 | 0.05 | 0.11 | 0.31 | 0.92 | 2.65 | 5.30 | 10.14 | 13.18 | 17.35 | 21.86 | 25.18 | 37.96 |

**Supplementary Table 8. Comparative Analysis of Age-Standardized type 2 diabetes mellitus death and DALY Rates attributed to ambient Particulate Matter Pollution by Countries in Relation to Sex, 1990 vs. 2019。**

|  | **Male** |  |  |  |  |  | **Female** |  |  |  |  |  |
| --- | --- | --- | --- | --- | --- | --- | --- | --- | --- | --- | --- | --- |
|  | ASDR (per 100000) No.(95%UI) |  |  | Age-standardized DALY rate (per 100000) No.(95%UI) |  |  | ASDR (per 100000) No.(95%UI) |  |  | Age-standardized DALY rate (per 100000) No.(95%UI) |  |  |
| Countiries | 1990 | 2019 | eapc | 1990 | 2019 | eapc | 1990 | 2019 | eapc | 1990 | 2019 | eapc |
| Afghanistan | 0.49(0.09,1.32) | 1.52(0.57,2.98) | 4.74(3.93,5.56) | 19.19(3.51,49.98) | 75.37(29.09,140.04) | 5.46(4.69,6.24) | 0.72(0.12,2.08) | 3.06(0.89,6.71) | 5.98(5.14,6.83) | 23.92(4.11,67.75) | 108.40(37.44,216.35) | 6.11(5.31,6.91) |
| Albania | 0.37(0.17,0.67) | 0.49(0.29,0.76) | 0.81(0.27,1.35) | 23.36(10.04,42.25) | 50.25(28.63,76.38) | 2.88(2.66,3.11) | 0.25(0.11,0.45) | 0.38(0.22,0.61) | 1.68(1.16,2.19) | 15.79(6.75,29.43) | 38.13(20.94,59.77) | 3.62(3.34,3.90) |
| Algeria | 2.48(1.42,3.84) | 3.11(1.98,4.44) | 0.94(0.79,1.08) | 100.10(60.59,145.72) | 184.03(115.50,263.75) | 2.13(2.08,2.18) | 3.17(1.64,5.34) | 4.88(3.11,7.47) | 1.97(1.79,2.15) | 109.21(61.66,167.68) | 221.81(142.18,314.83) | 2.58(2.46,2.69) |
| American Samoa | 3.58(0.92,9.77) | 4.64(1.54,10.45) | 0.58(0.24,0.93) | 118.74(29.84,314.14) | 174.39(58.38,386.63) | 0.98(0.72,1.25) | 2.24(0.50,6.27) | 3.88(1.43,8.84) | 1.42(1.15,1.69) | 74.47(16.69,207.21) | 146.12(53.15,329.31) | 1.86(1.62,2.11) |
| Andorra | 1.23(0.40,2.28) | 0.58(0.27,0.98) | -2.34(-2.63,-2.06) | 43.77(14.36,79.91) | 34.56(15.19,59.22) | -0.60(-0.87,-0.34) | 0.63(0.19,1.19) | 0.30(0.13,0.53) | -2.28(-2.43,-2.13) | 27.69(9.12,49.95) | 25.15(11.22,43.67) | -0.13(-0.31,0.05) |
| Angola | 1.18(0.27,3.11) | 5.68(2.76,9.47) | 5.74(5.29,6.20) | 34.45(8.06,89.10) | 170.50(80.69,286.60) | 5.89(5.42,6.36) | 0.41(0.09,1.01) | 2.30(1.05,4.09) | 6.20(5.84,6.57) | 13.01(3.05,33.52) | 74.43(34.06,129.49) | 6.31(5.91,6.71) |
| Antigua and Barbuda | 8.51(2.36,15.04) | 8.24(3.38,13.13) | -0.33(-0.66,0.00) | 251.96(70.99,441.96) | 280.55(111.06,461.33) | 0.13(-0.06,0.32) | 7.11(1.84,12.71) | 9.11(3.66,14.54) | 0.23(-0.04,0.49) | 215.55(55.36,387.77) | 285.73(114.04,461.62) | 0.49(0.29,0.70) |
| Argentina | 2.63(0.93,4.62) | 2.72(1.53,4.09) | -0.50(-0.79,-0.20) | 77.35(27.40,136.38) | 101.79(58.77,152.57) | 0.44(0.22,0.66) | 1.90(0.63,3.50) | 1.76(1.00,2.62) | -0.74(-0.95,-0.53) | 59.03(19.87,109.32) | 74.90(42.46,113.74) | 0.45(0.29,0.62) |
| Armenia | 2.30(1.15,3.50) | 5.59(3.77,7.72) | 2.58(1.96,3.21) | 88.50(43.86,135.09) | 212.71(141.49,289.14) | 2.89(2.42,3.35) | 2.62(1.21,4.13) | 5.94(3.97,8.07) | 2.35(1.56,3.14) | 97.71(45.01,155.57) | 217.70(143.61,297.19) | 2.62(2.07,3.19) |
| Australia | 0.74(0.07,1.75) | 0.50(0.12,1.04) | -1.92(-2.23,-1.61) | 23.51(2.09,56.93) | 22.60(5.01,47.07) | -0.59(-0.80,-0.38) | 0.53(0.05,1.28) | 0.32(0.08,0.64) | -2.15(-2.35,-1.95) | 17.67(1.81,43.02) | 16.08(3.51,33.34) | -0.68(-0.88,-0.48) |
| Austria | 2.08(1.10,3.09) | 1.52(0.92,2.23) | -0.62(-1.27,0.03) | 68.11(35.59,104.42) | 63.54(36.16,98.55) | 0.02(-0.49,0.54) | 1.98(1.05,2.93) | 0.99(0.60,1.46) | -2.02(-2.55,-1.49) | 59.69(30.97,91.10) | 46.43(26.36,71.05) | -0.77(-1.11,-0.43) |
| Azerbaijan | 1.22(0.51,2.11) | 4.08(2.38,6.05) | 3.92(3.49,4.34) | 54.14(21.28,93.41) | 169.04(99.23,245.06) | 3.79(3.49,4.10) | 0.93(0.36,1.70) | 3.97(2.22,6.10) | 4.85(4.20,5.50) | 44.45(17.18,83.18) | 169.21(96.10,255.40) | 4.70(4.25,5.16) |
| Bahamas | 5.78(1.46,10.40) | 5.20(1.62,9.18) | -0.91(-1.17,-0.66) | 195.43(47.51,355.16) | 216.62(64.73,390.69) | -0.03(-0.21,0.15) | 6.56(1.67,11.72) | 4.99(1.53,8.94) | -1.46(-1.74,-1.18) | 202.98(50.36,372.85) | 196.74(58.65,350.55) | -0.50(-0.70,-0.30) |
| Bahrain | 18.44(12.42,25.09) | 29.77(20.24,41.76) | 2.02(1.44,2.61) | 486.85(336.25,652.40) | 774.07(530.39,1070.38) | 1.69(1.28,2.10) | 15.88(10.90,22.21) | 30.89(20.76,41.73) | 2.65(2.09,3.21) | 420.94(292.01,569.13) | 761.95(533.00,1024.64) | 2.14(1.75,2.54) |
| Bangladesh | 1.07(0.27,2.45) | 3.16(1.77,4.89) | 3.30(2.68,3.92) | 29.28(7.24,68.34) | 95.57(53.99,147.48) | 3.96(3.66,4.26) | 0.65(0.16,1.58) | 3.12(1.67,4.84) | 5.45(5.00,5.91) | 18.81(4.86,45.33) | 85.36(47.19,131.79) | 5.34(5.13,5.56) |
| Barbados | 9.67(3.54,15.32) | 9.71(4.58,14.86) | -1.01(-1.37,-0.65) | 275.68(98.31,444.02) | 308.14(143.54,477.40) | -0.35(-0.60,-0.09) | 12.56(4.38,19.64) | 10.96(5.44,16.56) | -0.66(-0.80,-0.53) | 338.01(119.36,536.88) | 325.20(151.72,500.14) | -0.36(-0.49,-0.23) |
| Belarus | 0.71(0.39,1.03) | 0.39(0.24,0.58) | -3.03(-3.38,-2.68) | 51.07(27.28,78.62) | 43.01(24.50,66.52) | -0.94(-1.25,-0.63) | 0.73(0.41,1.08) | 0.29(0.17,0.44) | -4.64(-5.20,-4.08) | 51.28(27.17,80.11) | 38.92(21.60,61.25) | -1.29(-1.60,-0.99) |
| Belgium | 1.60(0.83,2.41) | 0.81(0.49,1.17) | -2.53(-2.85,-2.20) | 66.29(32.52,105.86) | 58.43(32.53,91.66) | -0.47(-0.73,-0.22) | 1.98(1.02,2.94) | 0.63(0.38,0.92) | -4.11(-4.38,-3.84) | 66.54(33.48,103.63) | 47.86(26.66,74.97) | -1.37(-1.58,-1.16) |
| Belize | 2.38(0.47,5.15) | 7.22(2.57,11.83) | 3.12(2.23,4.02) | 73.46(14.78,159.02) | 237.75(85.82,382.57) | 3.35(2.64,4.07) | 3.20(0.57,7.50) | 7.81(2.53,13.01) | 2.94(2.23,3.66) | 90.33(16.15,211.82) | 252.34(82.96,428.32) | 3.21(2.60,3.82) |
| Benin | 0.79(0.20,1.98) | 2.09(0.78,4.05) | 3.32(2.99,3.65) | 22.42(5.36,55.62) | 64.51(25.19,123.70) | 3.62(3.31,3.93) | 0.45(0.11,1.13) | 1.50(0.58,2.90) | 4.30(4.03,4.56) | 13.21(3.25,33.04) | 45.74(18.06,87.79) | 4.41(4.13,4.69) |
| Bermuda | 2.55(0.48,6.02) | 1.11(0.16,2.37) | -3.13(-3.32,-2.94) | 80.39(14.66,190.13) | 48.00(6.98,103.49) | -2.20(-2.43,-1.97) | 2.06(0.41,5.02) | 0.67(0.12,1.46) | -4.83(-5.21,-4.44) | 59.43(11.26,142.02) | 33.60(5.96,71.50) | -2.88(-3.25,-2.51) |
| Bhutan | 0.47(0.10,1.17) | 3.46(1.73,5.69) | 8.54(8.05,9.04) | 15.06(3.24,38.78) | 111.03(57.60,177.10) | 8.45(7.99,8.91) | 0.34(0.07,0.88) | 2.84(1.46,4.63) | 8.75(8.36,9.14) | 11.41(2.37,29.42) | 88.32(45.72,141.81) | 8.44(8.05,8.83) |
| Bolivia (Plurinational State of) | 3.02(1.10,5.65) | 5.19(2.82,8.13) | 1.70(1.50,1.89) | 95.99(35.54,175.91) | 168.68(97.83,250.39) | 1.77(1.58,1.95) | 3.85(1.28,7.63) | 6.39(3.40,10.04) | 1.48(1.13,1.82) | 107.19(36.26,207.59) | 175.85(96.80,269.73) | 1.44(1.09,1.78) |
| Bosnia and Herzegovina | 1.16(0.56,1.94) | 6.21(3.96,8.77) | 7.65(6.57,8.73) | 58.60(26.47,99.53) | 235.58(152.28,324.77) | 6.03(5.29,6.77) | 1.33(0.60,2.34) | 5.80(3.36,8.32) | 6.66(5.68,7.64) | 50.70(22.62,89.85) | 189.52(118.18,267.48) | 5.74(5.02,6.47) |
| Botswana | 3.13(1.34,5.76) | 13.22(7.45,20.73) | 4.59(3.67,5.50) | 82.61(35.42,147.08) | 343.10(195.17,523.00) | 4.58(3.68,5.48) | 2.20(0.93,4.18) | 10.27(5.48,16.69) | 5.70(5.11,6.29) | 56.40(24.02,103.88) | 268.42(147.65,429.66) | 5.86(5.34,6.39) |
| Brazil | 1.99(0.81,3.61) | 2.54(1.55,3.90) | 0.82(0.46,1.17) | 78.82(32.51,140.00) | 99.39(58.03,152.44) | 0.84(0.56,1.12) | 2.25(0.91,3.97) | 2.29(1.36,3.48) | -0.10(-0.43,0.24) | 73.64(29.64,131.34) | 82.68(47.41,127.97) | 0.32(0.04,0.60) |
| Brunei Darussalam | 8.01(2.19,17.04) | 5.76(1.56,11.02) | -0.03(-0.87,0.81) | 206.11(56.79,437.50) | 168.72(43.55,329.83) | 0.11(-0.52,0.75) | 5.26(1.50,11.36) | 3.54(0.90,6.96) | -0.78(-1.18,-0.38) | 143.65(39.69,304.07) | 113.84(29.59,227.41) | -0.32(-0.77,0.12) |
| Bulgaria | 2.33(1.29,3.41) | 2.32(1.47,3.30) | -0.87(-1.34,-0.40) | 102.81(55.29,156.22) | 123.35(74.05,176.02) | 0.17(-0.11,0.45) | 2.18(1.14,3.29) | 1.84(1.16,2.58) | -1.53(-2.01,-1.04) | 88.31(43.55,137.18) | 96.42(59.31,138.73) | -0.24(-0.54,0.07) |
| Burkina Faso | 0.89(0.16,2.52) | 1.86(0.53,4.33) | 2.87(2.70,3.03) | 22.82(4.13,65.56) | 52.43(14.86,118.60) | 3.18(3.02,3.34) | 0.40(0.07,1.19) | 0.81(0.23,1.88) | 2.73(2.56,2.90) | 11.60(2.01,34.56) | 26.48(7.51,62.16) | 3.23(3.06,3.39) |
| Burundi | 1.12(0.24,2.99) | 1.20(0.33,2.80) | 0.03(-0.22,0.28) | 30.23(6.37,80.99) | 34.16(9.41,78.04) | 0.17(-0.11,0.45) | 0.54(0.10,1.49) | 0.63(0.17,1.45) | 0.25(0.02,0.49) | 14.90(2.83,40.18) | 18.21(4.83,41.46) | 0.41(0.15,0.66) |
| Cabo Verde | 0.47(0.17,0.96) | 5.64(3.69,7.89) | 7.45(6.44,8.47) | 23.15(8.13,46.13) | 196.43(128.16,273.78) | 7.14(6.54,7.75) | 0.33(0.11,0.69) | 5.49(3.37,7.80) | 9.65(8.64,10.67) | 15.94(5.34,33.43) | 177.44(111.00,252.09) | 8.59(7.92,9.26) |
| Cambodia | 0.72(0.18,1.76) | 1.82(0.79,3.28) | 3.65(3.42,3.89) | 21.43(5.49,52.83) | 68.44(29.75,120.64) | 4.52(4.30,4.74) | 0.51(0.12,1.32) | 1.27(0.54,2.33) | 3.47(3.26,3.68) | 14.76(3.54,38.20) | 44.67(19.51,81.08) | 4.13(3.96,4.30) |
| Cameroon | 2.53(0.89,5.07) | 6.61(3.46,10.86) | 3.23(2.95,3.51) | 64.72(23.82,127.97) | 181.43(97.59,293.26) | 3.46(3.18,3.75) | 1.55(0.54,3.19) | 4.97(2.32,8.07) | 4.27(4.08,4.47) | 39.99(14.01,81.38) | 132.45(65.92,211.09) | 4.32(4.11,4.53) |
| Canada | 1.17(0.29,2.35) | 0.58(0.23,1.06) | -3.53(-4.40,-2.65) | 31.92(7.53,63.85) | 24.06(9.07,46.44) | -1.78(-2.29,-1.28) | 0.93(0.23,1.88) | 0.37(0.15,0.68) | -4.22(-5.03,-3.41) | 24.46(5.60,49.58) | 16.75(6.43,32.86) | -2.22(-2.68,-1.76) |
| Central African Republic | 1.79(0.37,4.58) | 2.63(0.70,5.96) | 1.36(1.22,1.51) | 52.49(10.95,136.63) | 83.84(22.44,193.32) | 1.64(1.49,1.80) | 0.55(0.11,1.44) | 1.01(0.25,2.41) | 2.21(2.01,2.42) | 17.40(3.36,44.04) | 33.60(8.56,78.41) | 2.41(2.23,2.59) |
| Chad | 0.51(0.09,1.48) | 1.23(0.34,2.83) | 3.08(2.79,3.37) | 15.33(2.60,44.58) | 39.21(10.76,88.23) | 3.35(3.10,3.60) | 0.36(0.06,1.06) | 1.10(0.27,2.68) | 4.30(4.13,4.47) | 10.54(1.68,32.21) | 32.93(8.42,77.73) | 4.33(4.17,4.50) |
| Chile | 2.06(0.96,3.28) | 3.04(2.11,4.04) | 1.30(0.97,1.64) | 75.42(33.33,122.24) | 134.89(87.73,188.21) | 1.99(1.77,2.20) | 1.63(0.72,2.75) | 2.45(1.65,3.27) | 1.24(0.97,1.51) | 61.43(25.64,107.54) | 115.50(72.29,163.37) | 2.20(1.94,2.47) |
| China | 0.72(0.31,1.24) | 2.05(1.41,2.75) | 4.04(3.66,4.42) | 36.09(15.53,63.85) | 101.57(67.77,138.44) | 3.87(3.50,4.25) | 0.63(0.28,1.17) | 1.55(1.03,2.14) | 3.41(2.90,3.93) | 29.61(12.88,54.84) | 79.05(51.77,109.80) | 3.75(3.29,4.21) |
| Colombia | 1.72(0.79,2.94) | 1.94(1.21,2.83) | -0.61(-1.21,0.00) | 96.10(43.15,169.04) | 146.85(91.89,213.90) | 0.95(0.60,1.30) | 2.07(0.89,3.66) | 1.97(1.21,2.88) | -1.14(-1.63,-0.64) | 85.49(35.83,156.27) | 122.73(76.89,180.06) | 0.64(0.29,0.99) |
| Comoros | 0.66(0.19,1.55) | 1.59(0.68,3.01) | 2.89(2.75,3.04) | 16.73(4.74,38.78) | 43.40(18.29,82.61) | 3.14(3.00,3.29) | 0.37(0.11,0.85) | 0.96(0.40,1.84) | 3.19(3.03,3.36) | 9.64(2.87,22.29) | 25.79(10.81,48.71) | 3.31(3.14,3.47) |
| Congo | 3.95(1.18,8.79) | 8.05(4.14,12.68) | 2.19(1.83,2.56) | 110.02(33.91,238.10) | 238.59(126.19,370.03) | 2.45(2.09,2.81) | 1.45(0.43,3.15) | 5.17(2.47,8.50) | 4.59(4.41,4.77) | 41.96(12.76,90.04) | 155.64(75.60,252.29) | 4.71(4.53,4.89) |
| Cook Islands | 4.37(1.34,11.38) | 4.88(0.77,12.21) | -0.11(-0.46,0.24) | 115.55(35.79,301.37) | 150.76(24.07,370.33) | 0.53(0.25,0.81) | 4.00(1.12,10.97) | 4.54(0.95,11.00) | 0.20(-0.03,0.43) | 106.37(30.33,289.94) | 133.58(29.39,320.22) | 0.57(0.38,0.76) |
| Costa Rica | 1.05(0.43,1.89) | 1.71(1.02,2.49) | 0.96(0.53,1.39) | 67.83(26.51,127.72) | 147.52(86.30,221.62) | 2.58(2.38,2.77) | 1.25(0.48,2.38) | 1.51(0.93,2.18) | -0.19(-0.72,0.34) | 55.86(21.31,108.48) | 109.96(65.56,166.14) | 1.98(1.65,2.30) |
| Croatia | 1.97(1.09,2.81) | 2.00(1.28,2.79) | -0.33(-0.68,0.02) | 101.90(52.09,153.13) | 124.87(76.96,179.52) | 0.44(0.23,0.65) | 1.84(0.95,2.74) | 1.54(0.97,2.18) | -1.22(-1.54,-0.90) | 82.88(40.00,126.08) | 93.36(56.52,135.07) | 0.05(-0.10,0.21) |
| Cuba | 2.13(0.83,3.47) | 1.34(0.66,2.11) | -1.77(-2.60,-0.94) | 130.89(48.16,230.53) | 143.05(68.04,237.29) | 0.21(-0.10,0.52) | 3.62(1.33,6.18) | 1.56(0.79,2.47) | -3.12(-3.77,-2.46) | 138.19(48.81,240.00) | 113.70(54.37,190.50) | -0.81(-1.19,-0.43) |
| Cyprus | 7.52(3.31,12.53) | 4.04(2.52,5.78) | -2.65(-3.27,-2.02) | 178.18(75.97,295.33) | 127.24(77.00,191.60) | -1.74(-2.17,-1.30) | 11.33(4.63,19.30) | 3.59(2.09,5.25) | -4.33(-4.51,-4.15) | 199.88(83.31,332.37) | 100.75(60.20,150.38) | -2.85(-3.07,-2.63) |
| Czechia | 2.43(1.37,3.36) | 2.54(1.62,3.61) | 2.16(1.23,3.09) | 139.14(74.71,209.64) | 177.77(108.23,261.49) | 1.33(1.02,1.64) | 2.06(1.15,2.87) | 1.83(1.14,2.56) | 1.42(0.53,2.32) | 109.46(59.31,163.36) | 127.82(75.61,190.74) | 0.97(0.64,1.29) |
| Côte d'Ivoire | 1.59(0.43,3.62) | 3.38(1.51,6.07) | 2.12(1.57,2.66) | 42.63(11.67,95.83) | 99.00(44.42,176.84) | 2.43(1.93,2.94) | 0.83(0.22,1.90) | 2.54(1.09,4.59) | 4.14(3.70,4.59) | 22.93(6.34,54.18) | 74.74(32.01,131.87) | 4.33(3.92,4.75) |
| Democratic People's Republic of Korea | 0.75(0.25,1.59) | 1.37(0.73,2.20) | 2.29(2.17,2.42) | 30.30(10.68,61.10) | 69.45(36.25,109.67) | 3.03(2.88,3.19) | 0.60(0.20,1.34) | 1.12(0.60,1.86) | 2.37(2.23,2.51) | 22.38(7.89,48.80) | 52.87(28.29,87.70) | 3.17(3.00,3.33) |
| Democratic Republic of the Congo | 1.70(0.42,4.28) | 2.53(0.90,4.89) | 0.84(0.14,1.55) | 46.95(11.56,117.52) | 78.87(27.86,155.54) | 1.32(0.63,2.01) | 0.56(0.13,1.38) | 0.87(0.32,1.78) | 1.10(0.45,1.76) | 16.99(4.01,41.70) | 31.05(11.24,62.16) | 1.71(1.04,2.39) |
| Denmark | 1.67(0.73,2.70) | 1.22(0.65,1.92) | -0.83(-1.62,-0.03) | 53.33(23.23,86.48) | 42.57(21.98,68.78) | -0.59(-1.15,-0.02) | 1.14(0.51,1.83) | 0.71(0.38,1.14) | -1.42(-2.08,-0.74) | 37.41(16.15,61.39) | 29.82(15.18,48.94) | -0.80(-1.18,-0.42) |
| Djibouti | 1.92(0.56,4.39) | 8.31(4.61,13.12) | 5.70(5.07,6.33) | 50.57(14.81,111.93) | 222.56(125.46,339.37) | 5.74(5.12,6.38) | 1.08(0.30,2.37) | 5.21(2.65,8.03) | 6.30(5.66,6.95) | 29.37(8.51,64.30) | 143.38(72.74,218.62) | 6.30(5.65,6.95) |
| Dominica | 4.95(1.62,9.55) | 8.05(3.49,12.98) | 1.05(0.69,1.42) | 157.21(51.67,305.52) | 329.21(138.70,538.61) | 2.10(1.77,2.42) | 5.17(1.60,10.59) | 9.30(3.95,15.41) | 1.68(1.26,2.10) | 142.03(42.82,293.20) | 292.08(121.31,478.52) | 2.08(1.68,2.49) |
| Dominican Republic | 0.88(0.23,2.02) | 4.20(1.68,7.58) | 6.39(5.92,6.87) | 31.01(8.03,73.98) | 157.09(60.31,271.85) | 6.44(6.08,6.80) | 0.81(0.20,2.00) | 3.46(1.34,6.31) | 6.09(5.61,6.57) | 24.83(6.06,61.84) | 122.65(46.46,217.62) | 6.39(5.98,6.80) |
| Ecuador | 1.83(0.75,3.22) | 5.29(3.18,7.73) | 3.88(3.44,4.33) | 68.61(28.32,119.64) | 182.93(113.84,259.46) | 3.55(3.14,3.96) | 2.20(0.82,4.07) | 5.43(3.20,8.00) | 3.52(3.03,4.01) | 69.09(26.35,129.33) | 178.10(105.78,254.18) | 3.58(3.12,4.04) |
| Egypt | 4.23(2.95,5.52) | 7.01(4.54,10.07) | 1.84(1.71,1.98) | 131.91(92.83,174.00) | 274.62(190.39,373.67) | 2.69(2.60,2.79) | 5.43(3.70,7.37) | 10.10(6.48,14.73) | 2.54(2.35,2.74) | 164.71(113.87,223.02) | 322.28(218.81,440.21) | 2.60(2.44,2.76) |
| El Salvador | 0.91(0.31,1.82) | 5.27(3.03,8.13) | 6.27(5.68,6.87) | 44.83(15.68,92.55) | 239.40(140.15,363.82) | 6.17(5.66,6.69) | 0.81(0.28,1.69) | 5.13(2.81,8.17) | 6.75(6.31,7.20) | 32.11(11.42,67.52) | 195.20(110.11,302.83) | 6.53(6.17,6.88) |
| Equatorial Guinea | 1.58(0.29,4.54) | 14.47(8.68,21.55) | 9.33(8.86,9.80) | 46.21(8.51,130.27) | 393.70(227.75,571.77) | 9.03(8.59,9.48) | 0.47(0.08,1.29) | 7.80(4.31,12.35) | 12.15(11.29,13.02) | 15.02(2.68,41.25) | 225.03(125.30,333.11) | 11.68(10.89,12.47) |
| Eritrea | 1.35(0.30,3.65) | 3.80(1.46,7.57) | 3.17(2.71,3.64) | 38.59(9.30,100.60) | 108.80(42.62,208.88) | 3.20(2.77,3.64) | 0.65(0.12,1.81) | 2.36(0.86,4.62) | 4.27(3.84,4.71) | 19.06(3.90,52.57) | 65.12(24.57,127.67) | 4.11(3.75,4.48) |
| Estonia | 0.28(0.10,0.54) | 0.19(0.06,0.38) | -0.85(-2.12,0.43) | 23.43(8.04,47.22) | 17.46(5.11,35.56) | -0.68(-1.67,0.33) | 0.21(0.07,0.42) | 0.15(0.05,0.31) | -1.11(-2.51,0.32) | 18.48(6.01,38.00) | 14.85(5.05,29.35) | -0.52(-1.49,0.45) |
| Eswatini | 4.21(1.65,7.87) | 17.80(9.16,29.23) | 5.10(4.46,5.73) | 104.18(41.91,194.85) | 439.49(227.45,702.24) | 5.15(4.47,5.82) | 2.48(0.98,4.86) | 9.21(4.10,16.09) | 5.04(4.38,5.71) | 61.80(25.27,119.62) | 223.43(106.73,387.36) | 4.91(4.28,5.55) |
| Ethiopia | 0.85(0.17,2.28) | 1.71(0.73,3.18) | 2.75(2.25,3.25) | 22.92(4.62,61.17) | 43.40(19.14,80.13) | 2.42(1.96,2.89) | 0.40(0.08,1.16) | 0.87(0.39,1.68) | 2.54(2.20,2.87) | 11.41(2.44,32.48) | 24.12(11.13,45.41) | 2.40(2.09,2.71) |
| Fiji | 4.99(1.05,14.95) | 21.52(5.65,47.34) | 4.66(3.67,5.66) | 134.09(28.08,407.52) | 552.45(148.75,1196.34) | 4.59(3.69,5.51) | 3.23(0.66,10.20) | 16.08(3.83,37.74) | 5.28(4.26,6.31) | 89.62(18.44,279.89) | 434.59(105.62,1013.68) | 5.25(4.27,6.23) |
| Finland | 0.42(0.05,0.94) | 0.12(0.02,0.27) | -4.65(-5.19,-4.11) | 27.32(2.99,63.48) | 14.54(2.47,35.72) | -2.19(-2.76,-1.62) | 0.45(0.05,1.01) | 0.07(0.01,0.17) | -6.71(-7.03,-6.39) | 24.98(2.95,59.23) | 12.70(2.14,30.90) | -2.35(-2.96,-1.73) |
| France | 1.33(0.63,2.06) | 0.95(0.57,1.42) | -0.98(-1.74,-0.21) | 40.31(18.62,64.31) | 34.73(19.87,54.25) | -0.50(-0.96,-0.04) | 1.10(0.53,1.74) | 0.59(0.34,0.90) | -2.15(-2.82,-1.48) | 30.08(14.20,48.23) | 24.09(13.76,37.73) | -0.66(-1.01,-0.30) |
| Gabon | 7.11(2.51,14.60) | 19.29(11.91,27.64) | 3.30(2.94,3.66) | 188.00(68.74,378.65) | 529.17(332.44,741.12) | 3.47(3.10,3.84) | 1.98(0.67,4.01) | 8.68(5.23,12.79) | 5.18(4.36,6.01) | 55.81(20.17,110.72) | 251.14(152.86,366.68) | 5.29(4.51,6.08) |
| Gambia | 0.87(0.20,2.24) | 2.68(1.10,4.93) | 3.74(3.53,3.96) | 23.91(5.70,59.01) | 77.49(32.58,139.74) | 4.02(3.84,4.19) | 0.52(0.12,1.37) | 2.12(0.85,4.10) | 4.99(4.81,5.18) | 14.48(3.37,36.86) | 59.71(24.53,112.44) | 5.04(4.87,5.20) |
| Georgia | 1.20(0.55,2.06) | 3.12(1.93,4.55) | 4.29(3.77,4.82) | 54.14(23.29,94.28) | 147.59(87.21,219.03) | 4.09(3.75,4.44) | 0.75(0.30,1.32) | 2.00(1.14,3.01) | 4.45(3.78,5.11) | 38.55(15.25,71.36) | 100.86(55.96,156.40) | 4.06(3.64,4.49) |
| Germany | 2.40(1.22,3.59) | 1.20(0.73,1.78) | -2.68(-2.94,-2.41) | 94.68(46.01,146.69) | 73.34(41.82,115.18) | -1.18(-1.39,-0.98) | 2.60(1.31,3.89) | 0.91(0.55,1.36) | -3.89(-4.17,-3.62) | 89.28(43.71,137.93) | 57.42(32.37,90.60) | -1.77(-2.08,-1.47) |
| Ghana | 1.71(0.67,3.20) | 8.12(4.69,12.25) | 6.39(5.91,6.87) | 48.36(19.55,92.10) | 230.09(130.34,340.64) | 6.40(5.93,6.88) | 1.42(0.53,2.86) | 3.89(2.19,6.08) | 3.54(3.28,3.79) | 41.17(15.41,82.00) | 122.66(66.55,183.52) | 4.10(3.89,4.31) |
| Greece | 1.06(0.55,1.59) | 0.74(0.47,1.04) | -0.32(-0.79,0.15) | 56.28(27.33,90.60) | 68.92(39.25,106.54) | 0.93(0.63,1.23) | 1.30(0.66,2.01) | 0.58(0.36,0.81) | -2.11(-2.54,-1.68) | 52.56(25.27,83.15) | 50.85(28.26,78.88) | 0.02(-0.23,0.27) |
| Greenland | 0.73(0.12,2.04) | 0.50(0.04,1.44) | -1.38(-1.63,-1.13) | 23.18(3.81,63.89) | 24.95(1.80,74.10) | 0.14(-0.18,0.46) | 0.65(0.12,1.88) | 0.25(0.02,0.74) | -4.59(-5.18,-3.99) | 15.84(2.88,44.83) | 13.03(1.20,38.22) | -1.73(-2.25,-1.21) |
| Grenada | 5.44(1.36,10.93) | 11.47(5.12,17.61) | 2.01(1.13,2.90) | 166.45(42.04,340.30) | 392.05(169.55,614.55) | 2.44(1.86,3.02) | 5.06(1.13,11.07) | 11.67(5.07,17.89) | 2.35(1.82,2.88) | 150.74(34.48,330.16) | 364.19(154.55,570.69) | 2.48(2.01,2.95) |
| Guam | 2.07(0.29,5.18) | 1.55(0.61,2.82) | -0.78(-1.42,-0.14) | 71.50(10.11,181.30) | 80.27(30.41,146.56) | 0.61(0.00,1.22) | 3.34(0.51,8.64) | 1.61(0.63,3.02) | -2.99(-3.93,-2.05) | 95.33(15.04,244.79) | 77.28(30.41,138.30) | -0.88(-1.70,-0.05) |
| Guatemala | 0.69(0.21,1.53) | 5.82(3.02,9.41) | 6.51(5.72,7.31) | 34.31(9.61,76.90) | 217.53(115.75,346.77) | 6.07(5.50,6.65) | 0.65(0.19,1.42) | 5.42(2.82,9.19) | 6.43(5.73,7.13) | 27.01(7.64,61.06) | 188.60(97.71,312.43) | 6.28(5.82,6.75) |
| Guinea | 0.64(0.15,1.73) | 1.62(0.57,3.37) | 3.44(3.15,3.72) | 17.82(4.02,47.51) | 48.93(17.40,100.47) | 3.71(3.47,3.95) | 0.58(0.12,1.61) | 1.52(0.50,3.31) | 3.69(3.54,3.84) | 15.39(3.33,43.72) | 42.43(14.30,90.39) | 3.81(3.69,3.94) |
| Guinea-Bissau | 1.37(0.29,3.51) | 2.66(0.96,5.35) | 2.24(2.04,2.44) | 37.94(8.08,99.78) | 78.08(28.92,158.44) | 2.46(2.29,2.63) | 0.69(0.14,1.96) | 2.05(0.70,4.36) | 3.89(3.70,4.09) | 19.87(4.27,54.53) | 58.21(20.53,124.55) | 3.88(3.71,4.05) |
| Guyana | 7.40(1.62,13.75) | 13.15(5.19,21.69) | 1.80(1.17,2.43) | 239.68(55.58,451.91) | 450.72(184.86,741.31) | 2.05(1.48,2.61) | 7.85(1.57,16.06) | 13.78(5.44,22.69) | 1.64(0.97,2.32) | 242.35(49.03,499.28) | 447.41(181.50,728.47) | 1.89(1.28,2.49) |
| Haiti | 0.87(0.20,2.22) | 1.42(0.49,3.14) | 2.01(1.81,2.21) | 29.04(7.34,73.71) | 59.37(20.54,128.15) | 2.77(2.59,2.94) | 1.38(0.31,3.84) | 2.34(0.76,5.63) | 2.08(1.89,2.27) | 39.94(8.97,108.61) | 76.00(25.55,173.78) | 2.45(2.29,2.62) |
| Honduras | 0.44(0.12,1.04) | 1.46(0.70,2.54) | 4.64(4.33,4.94) | 31.46(9.06,73.87) | 96.46(46.25,165.76) | 4.26(3.94,4.59) | 0.31(0.08,0.72) | 1.13(0.54,1.98) | 4.85(4.27,5.44) | 19.04(5.33,45.88) | 66.38(32.00,118.27) | 4.52(4.15,4.89) |
| Hungary | 1.57(0.86,2.34) | 1.94(1.23,2.73) | 1.03(0.54,1.53) | 90.33(46.79,142.58) | 124.54(74.29,180.25) | 1.15(0.71,1.59) | 1.66(0.84,2.52) | 1.35(0.87,1.89) | -0.63(-0.98,-0.29) | 76.27(36.79,122.29) | 84.20(50.03,123.57) | 0.38(0.13,0.64) |
| Iceland | 0.28(0.02,0.74) | 0.18(0.03,0.41) | -1.63(-2.06,-1.20) | 13.53(1.05,36.17) | 13.09(2.21,31.57) | 0.02(-0.29,0.33) | 0.21(0.02,0.55) | 0.06(0.01,0.14) | -4.43(-4.90,-3.95) | 10.70(0.90,28.73) | 9.14(1.51,22.32) | -0.59(-0.87,-0.31) |
| India | 1.56(0.62,2.82) | 4.80(3.16,6.65) | 4.33(4.11,4.55) | 53.42(22.83,97.85) | 188.36(122.61,260.96) | 4.57(4.44,4.70) | 1.12(0.49,2.14) | 3.77(2.41,5.26) | 4.44(4.18,4.69) | 37.04(16.64,68.92) | 144.25(95.30,200.48) | 4.91(4.76,5.06) |
| Indonesia | 2.05(0.84,3.78) | 6.05(3.72,9.04) | 3.69(3.55,3.83) | 65.05(27.76,119.88) | 188.29(113.90,274.87) | 3.54(3.40,3.67) | 1.63(0.69,3.03) | 4.90(2.93,7.29) | 3.76(3.57,3.95) | 52.29(22.59,96.01) | 151.13(89.28,223.61) | 3.50(3.31,3.70) |
| Iran (Islamic Republic of) | 2.23(1.56,3.00) | 4.43(3.19,5.73) | 2.83(2.57,3.10) | 98.93(68.06,136.22) | 202.84(142.65,272.15) | 2.91(2.75,3.07) | 2.78(1.88,3.77) | 5.39(3.72,6.94) | 2.57(2.40,2.73) | 102.68(71.08,138.35) | 221.95(156.30,304.32) | 2.95(2.83,3.07) |
| Iraq | 8.21(5.12,12.01) | 10.99(7.53,14.68) | 0.94(0.85,1.02) | 258.97(165.42,366.16) | 377.48(262.41,497.26) | 1.26(1.14,1.37) | 8.28(4.72,12.33) | 9.95(6.79,13.75) | 0.66(0.43,0.88) | 255.74(149.95,373.83) | 362.54(248.26,489.09) | 1.21(0.94,1.48) |
| Ireland | 1.26(0.37,2.27) | 0.48(0.21,0.83) | -3.67(-4.15,-3.19) | 34.89(10.38,65.58) | 27.79(11.57,51.01) | -0.99(-1.27,-0.71) | 0.99(0.29,1.80) | 0.30(0.13,0.51) | -4.19(-4.54,-3.84) | 27.08(7.86,50.32) | 20.11(8.35,36.85) | -1.07(-1.29,-0.84) |
| Israel | 2.92(1.58,4.28) | 3.83(2.60,5.08) | -0.23(-1.34,0.90) | 89.83(49.44,135.23) | 124.46(80.64,171.82) | 0.35(-0.42,1.12) | 3.05(1.72,4.50) | 3.13(2.05,4.24) | -1.00(-2.05,0.07) | 89.52(49.80,132.97) | 102.27(67.36,143.01) | -0.28(-0.98,0.44) |
| Italy | 2.99(1.66,4.33) | 1.87(1.21,2.60) | -1.51(-1.72,-1.29) | 100.61(56.35,147.19) | 90.85(55.61,135.14) | -0.05(-0.49,0.39) | 3.38(1.85,4.86) | 1.39(0.88,1.95) | -3.08(-3.19,-2.97) | 97.70(54.95,142.94) | 68.58(42.06,102.82) | -1.13(-1.38,-0.89) |
| Jamaica | 2.94(1.11,5.55) | 8.52(4.81,12.73) | 3.62(2.85,4.40) | 85.36(31.92,162.43) | 263.41(150.91,399.98) | 3.72(3.02,4.43) | 3.28(1.21,6.69) | 9.83(5.44,15.12) | 3.22(2.42,4.02) | 91.28(32.98,186.22) | 286.81(161.17,435.18) | 3.46(2.70,4.23) |
| Japan | 0.79(0.24,1.37) | 0.31(0.18,0.46) | -3.22(-3.72,-2.73) | 46.80(13.56,83.67) | 48.26(26.58,76.60) | -0.13(-0.52,0.25) | 0.62(0.19,1.06) | 0.17(0.10,0.27) | -4.39(-5.08,-3.69) | 29.41(8.44,52.50) | 29.60(16.24,47.84) | -0.46(-0.94,0.02) |
| Jordan | 9.36(6.20,13.05) | 8.22(5.58,11.37) | -0.44(-0.63,-0.24) | 268.73(181.77,365.16) | 285.71(196.63,390.80) | 0.16(-0.03,0.36) | 16.22(10.99,22.19) | 8.99(6.14,12.43) | -2.59(-3.31,-1.86) | 380.52(261.23,514.04) | 259.96(176.37,355.57) | -1.81(-2.37,-1.24) |
| Kazakhstan | 0.61(0.27,1.01) | 2.16(1.33,3.12) | 3.35(2.52,4.19) | 40.73(17.18,71.55) | 112.82(65.29,170.93) | 3.23(2.86,3.61) | 0.67(0.27,1.19) | 2.30(1.39,3.40) | 3.09(2.19,4.00) | 47.59(18.39,89.14) | 133.71(77.25,200.37) | 3.46(3.06,3.87) |
| Kenya | 0.91(0.36,1.84) | 2.75(1.41,4.55) | 4.39(3.95,4.82) | 24.24(9.89,47.34) | 76.35(38.77,126.28) | 4.55(4.10,5.01) | 0.44(0.18,0.85) | 1.11(0.53,1.92) | 3.97(3.70,4.25) | 11.89(4.97,23.15) | 32.29(15.81,56.03) | 4.17(3.90,4.44) |
| Kiribati | 2.46(0.52,6.88) | 6.35(1.63,15.77) | 2.07(1.25,2.90) | 75.43(16.08,211.71) | 191.87(51.39,483.71) | 2.06(1.29,2.84) | 1.77(0.40,5.20) | 3.36(0.82,8.98) | 1.09(0.56,1.63) | 52.66(12.08,155.91) | 98.73(24.54,264.93) | 1.09(0.59,1.59) |
| Kuwait | 5.80(4.16,7.56) | 4.69(3.18,6.47) | -1.25(-2.11,-0.39) | 241.81(165.99,328.75) | 289.98(189.04,413.17) | 0.26(-0.22,0.74) | 7.91(5.68,10.31) | 4.00(2.67,5.40) | -2.20(-2.80,-1.60) | 265.58(187.74,352.65) | 243.16(157.78,344.75) | -0.39(-0.80,0.02) |
| Kyrgyzstan | 0.47(0.18,0.89) | 0.87(0.49,1.29) | 1.00(0.50,1.51) | 26.22(9.74,50.10) | 58.21(32.75,90.40) | 2.11(1.80,2.43) | 0.36(0.13,0.71) | 0.76(0.42,1.18) | 1.41(0.88,1.94) | 20.23(6.94,42.07) | 52.08(27.60,83.78) | 2.63(2.29,2.96) |
| Lao People's Democratic Republic | 0.76(0.19,1.90) | 1.69(0.72,3.16) | 2.71(2.52,2.90) | 25.20(6.23,62.65) | 64.72(27.97,115.19) | 3.27(3.09,3.46) | 0.64(0.16,1.69) | 1.52(0.67,2.81) | 2.82(2.61,3.02) | 20.24(5.04,52.95) | 52.69(24.16,93.73) | 3.21(3.02,3.40) |
| Latvia | 0.64(0.33,0.98) | 0.84(0.47,1.29) | 1.24(0.43,2.05) | 46.52(23.58,74.97) | 55.77(30.79,86.00) | 0.89(0.28,1.49) | 0.67(0.34,1.04) | 0.77(0.43,1.24) | 0.74(-0.04,1.52) | 43.78(20.86,71.14) | 48.72(27.21,75.93) | 0.62(0.06,1.18) |
| Lebanon | 3.91(2.51,5.69) | 4.42(2.56,6.67) | 0.83(0.51,1.16) | 144.41(91.68,207.24) | 238.88(152.58,340.20) | 1.97(1.78,2.17) | 2.72(1.63,3.98) | 2.41(1.53,3.63) | -0.67(-0.81,-0.54) | 113.33(68.41,163.83) | 178.42(111.27,254.51) | 1.44(1.28,1.60) |
| Lesotho | 2.40(0.89,4.81) | 10.38(5.16,17.88) | 5.49(5.18,5.79) | 62.37(22.96,122.78) | 269.74(134.19,461.92) | 5.59(5.28,5.91) | 1.42(0.53,2.88) | 7.41(3.27,13.66) | 7.16(6.63,7.70) | 36.89(13.98,74.31) | 184.09(86.07,330.26) | 6.93(6.44,7.42) |
| Liberia | 0.91(0.23,2.23) | 1.85(0.70,3.66) | 3.48(3.08,3.87) | 27.44(7.39,66.79) | 63.77(25.08,125.20) | 4.05(3.61,4.49) | 0.67(0.18,1.68) | 1.75(0.67,3.48) | 4.56(4.09,5.02) | 19.65(5.43,49.85) | 53.86(21.73,108.58) | 4.81(4.33,5.29) |
| Libya | 2.13(1.16,3.22) | 3.76(2.19,6.01) | 2.00(1.74,2.27) | 109.82(62.46,157.79) | 248.60(156.98,358.20) | 2.69(2.37,3.02) | 2.27(1.24,3.69) | 4.37(2.80,6.35) | 2.48(2.16,2.81) | 101.84(57.60,153.42) | 254.88(165.48,359.25) | 3.04(2.70,3.38) |
| Lithuania | 0.51(0.23,0.83) | 0.41(0.23,0.67) | -0.48(-1.02,0.07) | 40.39(17.98,68.74) | 36.52(18.85,61.13) | -0.09(-0.62,0.44) | 0.44(0.19,0.71) | 0.30(0.16,0.48) | -1.50(-1.96,-1.04) | 33.29(14.12,56.29) | 27.42(14.51,45.29) | -0.40(-0.87,0.07) |
| Luxembourg | 1.44(0.60,2.36) | 0.59(0.33,0.92) | -2.94(-3.33,-2.55) | 44.29(18.02,75.21) | 56.45(28.47,95.52) | 1.21(0.90,1.52) | 1.41(0.60,2.31) | 0.46(0.25,0.73) | -3.70(-4.07,-3.33) | 39.83(16.07,67.65) | 43.82(22.47,73.11) | 0.48(0.11,0.85) |
| Madagascar | 0.46(0.13,1.05) | 0.96(0.38,1.96) | 2.97(2.78,3.16) | 12.53(3.51,28.08) | 28.61(11.22,57.25) | 3.30(3.12,3.48) | 0.26(0.08,0.62) | 0.58(0.24,1.20) | 3.03(2.87,3.19) | 7.53(2.28,17.44) | 17.48(7.37,34.87) | 3.24(3.09,3.40) |
| Malawi | 0.67(0.15,1.73) | 1.59(0.59,3.17) | 3.62(3.30,3.95) | 17.84(4.12,45.86) | 45.76(17.01,91.47) | 3.88(3.56,4.21) | 0.35(0.08,0.92) | 0.58(0.22,1.20) | 2.27(2.00,2.54) | 9.76(2.02,25.25) | 17.78(6.76,35.59) | 2.66(2.41,2.92) |
| Malaysia | 4.02(2.07,5.84) | 1.83(1.10,2.67) | -3.79(-4.45,-3.13) | 154.53(80.28,224.69) | 117.69(70.68,172.83) | -1.17(-1.44,-0.91) | 5.03(2.58,7.55) | 2.06(1.22,3.27) | -3.60(-4.09,-3.11) | 170.66(85.59,249.51) | 120.53(71.50,179.73) | -1.27(-1.45,-1.10) |
| Maldives | 1.01(0.28,2.37) | 1.67(0.89,2.76) | 1.28(0.89,1.68) | 31.44(8.71,74.37) | 67.30(33.28,110.51) | 2.31(1.91,2.71) | 1.03(0.27,2.65) | 1.23(0.64,2.11) | -0.12(-0.57,0.32) | 30.03(7.69,77.78) | 51.94(25.58,87.15) | 1.46(0.98,1.93) |
| Mali | 0.59(0.10,1.67) | 1.17(0.34,2.59) | 2.67(2.58,2.76) | 16.48(2.88,47.74) | 36.09(10.04,80.43) | 3.03(2.93,3.13) | 0.53(0.09,1.53) | 1.37(0.39,3.26) | 3.60(3.40,3.81) | 14.76(2.63,43.46) | 38.90(11.04,89.83) | 3.65(3.49,3.81) |
| Malta | 3.00(1.28,4.97) | 1.68(0.98,2.41) | -1.67(-2.09,-1.23) | 95.26(41.41,160.31) | 79.73(45.06,123.15) | -0.35(-0.74,0.04) | 3.45(1.48,5.61) | 1.24(0.71,1.82) | -3.46(-3.77,-3.14) | 98.93(42.42,167.28) | 72.52(40.32,114.25) | -1.03(-1.27,-0.78) |
| Marshall Islands | 1.72(0.30,5.41) | 4.12(1.03,10.03) | 2.82(2.41,3.23) | 59.64(10.16,184.05) | 170.46(43.05,408.94) | 3.36(2.92,3.80) | 1.38(0.25,4.54) | 5.25(1.28,13.33) | 4.17(3.48,4.85) | 45.38(8.11,145.94) | 177.22(44.18,450.61) | 4.26(3.61,4.92) |
| Mauritania | 2.09(0.73,4.28) | 3.83(2.00,6.48) | 1.69(1.47,1.91) | 52.87(18.02,107.00) | 99.89(53.61,161.04) | 1.78(1.56,2.01) | 1.70(0.58,3.80) | 5.03(2.52,8.38) | 3.61(3.39,3.83) | 43.58(15.27,96.29) | 129.08(65.69,211.65) | 3.56(3.37,3.75) |
| Mauritius | 4.91(2.26,7.76) | 16.17(7.83,25.41) | 6.27(5.11,7.44) | 176.20(79.54,278.26) | 488.83(235.67,753.99) | 5.19(4.26,6.12) | 4.01(1.72,6.51) | 12.60(6.12,19.55) | 5.76(4.68,6.86) | 136.31(56.87,222.11) | 375.36(181.55,579.26) | 4.69(3.89,5.50) |
| Mexico | 8.06(4.39,11.90) | 10.19(6.69,14.44) | 0.38(0.14,0.63) | 285.74(157.66,427.94) | 365.71(239.48,509.06) | 0.55(0.32,0.79) | 9.16(5.10,13.75) | 8.28(5.44,11.71) | -0.83(-1.10,-0.57) | 287.64(156.85,438.76) | 285.12(183.94,399.49) | -0.49(-0.72,-0.26) |
| Micronesia (Federated States of) | 2.46(0.42,7.20) | 9.93(2.34,24.83) | 4.76(4.24,5.28) | 71.93(12.76,213.98) | 295.67(69.99,718.10) | 4.86(4.32,5.40) | 1.85(0.35,5.75) | 8.21(1.77,21.32) | 5.14(4.64,5.64) | 51.93(9.98,159.47) | 230.54(51.64,605.30) | 5.18(4.67,5.68) |
| Monaco | 0.34(0.04,0.75) | 0.47(0.24,0.72) | 1.68(0.63,2.74) | 20.39(1.98,44.56) | 42.92(20.90,70.78) | 3.14(2.27,4.03) | 0.21(0.02,0.45) | 0.23(0.12,0.37) | 0.98(0.10,1.87) | 15.47(1.59,34.25) | 32.74(15.83,55.62) | 3.09(2.31,3.88) |
| Mongolia | 0.36(0.14,0.67) | 1.10(0.67,1.62) | 3.78(3.57,3.99) | 16.33(6.24,30.63) | 58.14(34.63,83.11) | 4.77(4.63,4.91) | 0.18(0.07,0.36) | 0.51(0.30,0.79) | 3.68(3.43,3.93) | 10.15(3.83,19.57) | 37.51(21.83,56.57) | 4.89(4.73,5.05) |
| Montenegro | 1.87(1.08,2.75) | 2.26(1.45,3.16) | 0.91(0.73,1.09) | 103.45(55.75,157.18) | 142.19(83.95,208.15) | 1.17(1.03,1.30) | 1.71(0.90,2.61) | 1.90(1.17,2.73) | 0.66(0.41,0.91) | 83.72(42.89,131.85) | 115.83(67.68,169.17) | 1.22(1.08,1.36) |
| Morocco | 1.09(0.49,1.88) | 4.09(2.62,5.67) | 4.65(4.45,4.85) | 48.73(21.86,79.53) | 196.00(128.70,267.96) | 5.01(4.88,5.13) | 1.13(0.49,2.19) | 5.17(3.43,7.33) | 5.98(5.55,6.41) | 44.44(19.62,79.26) | 220.41(146.33,306.75) | 6.05(5.70,6.41) |
| Mozambique | 0.41(0.07,1.15) | 1.48(0.50,3.09) | 5.58(5.23,5.94) | 10.79(1.92,31.05) | 41.14(14.20,86.91) | 5.81(5.44,6.17) | 0.20(0.03,0.56) | 0.49(0.16,1.06) | 3.94(3.53,4.34) | 5.33(0.90,15.34) | 14.27(4.78,30.50) | 4.20(3.81,4.59) |
| Myanmar | 1.92(0.57,4.25) | 5.30(2.87,8.20) | 4.11(3.79,4.42) | 55.18(16.56,123.45) | 158.74(85.95,247.25) | 4.23(3.92,4.54) | 1.32(0.39,3.13) | 2.98(1.61,4.66) | 3.20(2.92,3.48) | 40.74(12.29,97.67) | 97.79(53.17,154.17) | 3.42(3.14,3.70) |
| Namibia | 3.11(1.30,5.80) | 8.53(4.45,13.33) | 3.40(2.97,3.83) | 81.27(34.36,146.79) | 222.06(117.17,341.86) | 3.44(3.02,3.86) | 2.24(0.94,4.19) | 5.55(2.69,9.25) | 2.92(2.47,3.37) | 59.14(25.19,108.79) | 147.58(72.51,238.06) | 2.99(2.60,3.37) |
| Nauru | 3.10(0.57,10.41) | 5.40(0.93,14.20) | 0.86(0.49,1.25) | 89.86(16.77,293.02) | 161.46(27.79,438.69) | 0.98(0.61,1.36) | 2.86(0.49,9.79) | 5.39(1.00,15.55) | 1.23(0.82,1.63) | 75.48(13.04,260.81) | 146.61(27.22,422.49) | 1.34(0.94,1.74) |
| Nepal | 0.42(0.09,1.04) | 2.21(1.13,3.54) | 6.58(6.15,7.02) | 19.92(4.37,49.29) | 111.52(58.38,175.10) | 6.59(6.35,6.84) | 0.30(0.06,0.81) | 2.01(0.98,3.28) | 7.48(7.07,7.89) | 11.08(2.43,29.02) | 69.34(35.02,117.50) | 7.00(6.71,7.28) |
| Netherlands | 2.64(1.31,3.98) | 1.06(0.64,1.55) | -3.45(-3.91,-2.99) | 86.79(42.09,135.70) | 51.90(30.18,80.23) | -1.97(-2.20,-1.75) | 2.73(1.34,4.14) | 0.85(0.50,1.26) | -4.38(-4.77,-3.99) | 75.44(36.48,116.25) | 37.94(21.51,59.01) | -2.57(-2.72,-2.43) |
| New Zealand | 0.57(0.03,1.49) | 0.34(0.05,0.75) | -3.12(-3.75,-2.49) | 18.78(1.11,48.76) | 15.54(2.45,35.90) | -1.81(-2.20,-1.41) | 0.43(0.03,1.11) | 0.22(0.04,0.51) | -3.33(-3.85,-2.81) | 15.85(1.13,40.29) | 11.93(1.88,27.47) | -2.02(-2.41,-1.62) |
| Nicaragua | 0.96(0.27,2.19) | 4.18(2.03,7.05) | 5.11(4.80,5.42) | 35.50(10.36,84.20) | 150.70(71.22,250.09) | 5.14(4.86,5.41) | 0.55(0.15,1.26) | 3.03(1.45,5.23) | 5.79(4.97,6.61) | 22.06(6.04,52.35) | 105.05(50.70,180.60) | 5.42(5.00,5.85) |
| Niger | 0.60(0.09,1.89) | 0.99(0.20,2.66) | 1.88(1.67,2.09) | 14.76(2.16,46.57) | 27.83(5.79,76.90) | 2.27(2.08,2.45) | 0.41(0.06,1.34) | 0.94(0.18,2.78) | 2.88(2.58,3.19) | 10.23(1.45,33.57) | 25.03(4.94,73.46) | 3.06(2.81,3.30) |
| Nigeria | 1.73(0.64,3.65) | 4.94(2.83,7.82) | 3.94(3.81,4.06) | 42.02(15.63,88.15) | 120.43(69.63,188.75) | 3.91(3.80,4.02) | 1.01(0.37,2.16) | 3.48(1.95,5.56) | 4.61(4.40,4.82) | 25.66(9.41,54.11) | 90.03(49.10,141.81) | 4.52(4.29,4.76) |
| Niue | 2.87(0.92,7.07) | 6.10(1.04,15.13) | 2.17(1.70,2.64) | 90.09(27.92,216.83) | 198.70(32.46,482.59) | 2.30(1.88,2.72) | 2.31(0.68,5.92) | 5.45(1.06,13.96) | 2.57(2.19,2.95) | 67.32(19.73,166.76) | 173.47(32.90,428.13) | 2.91(2.57,3.25) |
| North Macedonia | 2.54(1.54,3.73) | 5.78(3.84,8.09) | 3.11(2.45,3.77) | 115.19(63.81,172.74) | 236.37(156.66,324.71) | 2.63(2.21,3.05) | 3.16(1.69,4.82) | 6.25(4.05,8.70) | 2.92(2.42,3.43) | 112.11(59.15,170.73) | 217.89(140.38,300.02) | 2.65(2.30,2.99) |
| Northern Mariana Islands | 3.27(0.93,7.21) | 4.24(1.96,7.37) | 1.14(0.73,1.54) | 107.74(30.45,239.50) | 147.08(66.40,255.54) | 1.19(0.87,1.51) | 3.77(1.02,9.14) | 3.27(1.52,5.87) | -0.46(-0.80,-0.12) | 112.79(31.46,269.26) | 116.62(54.59,203.29) | 0.06(-0.25,0.38) |
| Norway | 0.77(0.20,1.47) | 0.28(0.09,0.54) | -3.73(-4.33,-3.14) | 41.66(10.96,82.14) | 20.27(6.36,40.84) | -3.03(-3.40,-2.67) | 0.57(0.16,1.10) | 0.19(0.06,0.38) | -3.85(-4.37,-3.33) | 31.18(8.29,60.78) | 16.03(5.02,32.40) | -3.02(-3.30,-2.74) |
| Oman | 5.54(2.88,8.94) | 14.78(10.26,19.96) | 3.52(3.02,4.03) | 155.47(78.84,246.32) | 393.45(276.63,521.65) | 3.04(2.48,3.61) | 5.26(2.49,8.96) | 12.43(8.67,16.85) | 2.66(2.01,3.31) | 146.21(70.73,241.97) | 353.40(244.84,469.97) | 2.57(1.89,3.25) |
| Pakistan | 1.16(0.31,2.63) | 6.24(3.52,9.38) | 6.53(6.20,6.85) | 40.50(11.50,90.86) | 205.41(119.07,307.66) | 6.33(6.04,6.61) | 0.77(0.20,1.79) | 6.47(3.39,10.31) | 8.27(7.90,8.64) | 26.06(6.87,58.85) | 191.46(106.79,290.69) | 7.85(7.54,8.16) |
| Palau | 3.45(0.02,10.01) | 5.52(0.02,15.15) | 2.35(1.57,3.13) | 107.01(0.63,308.95) | 179.39(0.49,489.63) | 2.53(1.76,3.31) | 3.85(0.03,11.96) | 5.43(0.02,14.75) | 1.99(1.23,2.77) | 110.77(0.72,335.60) | 163.72(0.57,438.01) | 2.20(1.43,2.97) |
| Palestine | 5.77(2.68,9.61) | 15.04(10.45,19.85) | 2.88(2.58,3.17) | 155.31(73.66,253.65) | 426.44(294.53,565.67) | 3.03(2.75,3.32) | 5.18(2.30,9.16) | 13.99(9.62,18.64) | 2.99(2.69,3.28) | 129.68(58.28,224.15) | 372.43(263.08,495.67) | 3.26(2.98,3.55) |
| Panama | 1.21(0.46,2.32) | 3.09(1.70,4.89) | 3.24(2.70,3.79) | 62.41(22.15,123.42) | 146.54(77.16,227.60) | 3.35(2.92,3.79) | 1.41(0.51,2.83) | 3.01(1.61,4.76) | 2.79(2.22,3.37) | 53.67(18.93,108.65) | 125.66(67.12,201.57) | 3.17(2.76,3.59) |
| Papua New Guinea | 1.89(0.31,6.07) | 3.68(0.80,10.25) | 2.10(1.97,2.23) | 53.64(8.45,181.67) | 108.70(23.01,295.14) | 2.27(2.13,2.41) | 0.57(0.09,1.90) | 1.38(0.31,3.96) | 2.99(2.86,3.12) | 18.51(2.93,61.04) | 46.34(10.59,129.07) | 3.12(2.98,3.25) |
| Paraguay | 0.88(0.33,1.73) | 3.55(1.76,6.00) | 5.03(4.38,5.68) | 30.39(11.18,61.09) | 114.90(59.45,188.90) | 4.53(4.01,5.04) | 0.88(0.32,1.84) | 3.10(1.52,5.28) | 4.42(3.74,5.11) | 27.74(10.29,57.26) | 90.05(45.33,152.73) | 4.06(3.54,4.58) |
| Peru | 1.39(0.59,2.35) | 2.51(1.52,3.82) | 2.53(2.14,2.92) | 51.25(22.11,86.41) | 108.43(68.89,153.74) | 2.83(2.51,3.16) | 1.12(0.46,2.07) | 2.27(1.36,3.39) | 2.39(1.97,2.82) | 39.44(16.37,71.69) | 94.74(58.31,134.36) | 2.95(2.60,3.30) |
| Philippines | 3.09(1.58,5.06) | 3.95(2.34,5.98) | 0.10(-0.29,0.50) | 92.85(49.20,150.36) | 128.41(77.05,190.75) | 0.34(0.01,0.68) | 2.49(1.22,4.18) | 3.09(1.89,4.76) | 0.16(-0.12,0.44) | 61.97(31.36,106.23) | 98.38(60.46,150.03) | 0.97(0.71,1.22) |
| Poland | 1.85(1.08,2.63) | 2.12(1.42,2.92) | 0.84(0.51,1.17) | 105.97(58.77,156.91) | 143.55(92.16,199.18) | 1.25(0.93,1.57) | 1.90(1.11,2.77) | 1.50(0.95,2.07) | -0.71(-1.03,-0.40) | 94.58(51.24,142.87) | 101.85(64.63,145.51) | 0.40(0.07,0.74) |
| Portugal | 1.86(0.53,3.56) | 1.06(0.51,1.78) | -2.44(-3.11,-1.76) | 63.23(18.50,123.69) | 48.72(22.33,85.64) | -1.11(-1.65,-0.56) | 1.80(0.51,3.44) | 0.88(0.42,1.49) | -3.09(-3.75,-2.43) | 57.23(16.16,114.00) | 41.77(18.96,74.76) | -1.43(-1.95,-0.90) |
| Puerto Rico | 2.35(0.01,7.21) | 2.18(0.42,4.41) | -1.03(-1.36,-0.69) | 83.37(0.40,255.91) | 88.72(17.70,183.22) | -0.45(-0.78,-0.13) | 2.47(0.01,7.46) | 1.66(0.31,3.42) | -2.25(-2.64,-1.86) | 78.06(0.43,234.73) | 70.66(13.94,143.21) | -1.09(-1.44,-0.73) |
| Qatar | 27.34(18.92,36.85) | 26.10(17.75,36.58) | 0.14(-0.37,0.66) | 616.72(432.27,823.47) | 672.11(455.80,920.73) | 0.44(0.04,0.84) | 27.12(18.26,37.71) | 42.14(28.23,58.48) | 2.41(1.66,3.18) | 622.68(431.73,831.39) | 880.83(609.30,1201.95) | 1.72(1.06,2.39) |
| Republic of Korea | 3.99(2.13,5.51) | 3.68(2.53,4.80) | -1.01(-1.79,-0.23) | 143.82(74.02,205.87) | 143.03(96.62,197.69) | -0.68(-1.13,-0.22) | 2.54(1.31,3.66) | 2.48(1.59,3.31) | -1.14(-2.10,-0.18) | 96.95(50.05,146.58) | 101.85(66.86,142.35) | -0.66(-1.19,-0.12) |
| Republic of Moldova | 0.67(0.29,1.10) | 0.59(0.33,0.91) | -0.64(-1.08,-0.20) | 44.94(19.30,75.89) | 59.12(30.07,95.95) | 1.22(0.74,1.70) | 0.57(0.24,1.02) | 0.50(0.27,0.77) | -0.71(-1.19,-0.23) | 38.06(15.33,69.71) | 53.18(26.34,86.06) | 1.66(1.17,2.15) |
| Romania | 0.83(0.40,1.28) | 0.93(0.59,1.32) | 0.40(0.11,0.69) | 51.55(23.54,87.14) | 73.61(44.23,108.92) | 1.21(1.03,1.39) | 0.68(0.32,1.11) | 0.67(0.43,0.98) | -0.04(-0.27,0.20) | 38.71(16.48,66.35) | 54.91(32.71,82.31) | 1.22(1.07,1.37) |
| Russian Federation | 0.49(0.22,0.80) | 0.61(0.31,1.00) | 0.77(0.15,1.39) | 35.07(15.25,58.98) | 37.12(18.15,61.30) | 0.29(0.11,0.47) | 0.62(0.28,1.01) | 0.80(0.38,1.30) | 0.71(0.04,1.38) | 37.76(16.32,63.14) | 39.48(19.36,64.42) | 0.26(0.09,0.42) |
| Rwanda | 1.65(0.38,4.13) | 2.70(0.98,5.33) | 1.16(0.79,1.52) | 43.24(9.96,107.45) | 70.87(26.04,139.93) | 1.09(0.69,1.50) | 0.81(0.18,2.12) | 1.31(0.48,2.57) | 1.39(1.02,1.77) | 21.94(5.01,55.86) | 35.54(13.84,68.25) | 1.40(1.03,1.78) |
| Saint Kitts and Nevis | 3.53(0.82,7.93) | 4.31(1.64,7.68) | 0.05(-0.24,0.34) | 108.95(25.19,243.86) | 152.13(58.96,272.81) | 0.46(0.19,0.73) | 3.78(0.82,8.78) | 3.52(1.38,6.25) | 0.01(-0.27,0.28) | 109.49(24.43,249.11) | 121.16(47.38,216.49) | 0.22(-0.03,0.47) |
| Saint Lucia | 6.84(2.06,13.35) | 10.15(4.84,15.72) | 0.41(0.04,0.79) | 206.50(62.56,398.97) | 365.54(162.02,570.72) | 1.42(1.14,1.70) | 6.98(1.88,14.73) | 9.69(4.22,15.06) | -0.33(-0.85,0.19) | 212.09(56.81,441.33) | 371.41(158.08,587.62) | 0.94(0.56,1.31) |
| Saint Vincent and the Grenadines | 5.91(1.55,11.58) | 12.04(5.14,18.90) | 1.61(1.13,2.08) | 181.00(47.11,353.42) | 407.14(169.03,658.30) | 2.08(1.66,2.50) | 7.75(1.85,16.54) | 13.54(5.47,21.34) | 1.21(0.77,1.66) | 209.62(48.94,453.50) | 419.45(170.35,680.04) | 1.64(1.21,2.09) |
| Samoa | 3.07(0.67,8.38) | 4.30(1.13,10.30) | 0.66(0.43,0.89) | 89.15(19.94,249.93) | 138.68(36.76,330.06) | 1.02(0.80,1.25) | 1.70(0.37,5.02) | 3.42(0.83,8.66) | 2.01(1.67,2.35) | 51.29(11.61,153.53) | 110.03(26.22,277.69) | 2.26(1.97,2.55) |
| San Marino | 0.85(0.19,1.65) | 0.61(0.22,1.11) | -1.38(-1.64,-1.13) | 35.09(7.85,67.78) | 40.13(14.71,73.10) | 0.22(-0.06,0.50) | 0.71(0.15,1.37) | 0.43(0.14,0.81) | -1.69(-1.93,-1.44) | 28.31(6.25,55.79) | 32.54(11.82,58.60) | 0.29(0.03,0.56) |
| Sao Tome and Principe | 0.48(0.15,1.05) | 1.86(0.89,3.20) | 5.37(5.09,5.65) | 17.65(5.67,39.69) | 75.85(35.16,131.45) | 5.67(5.44,5.91) | 0.21(0.07,0.46) | 0.86(0.37,1.52) | 5.34(5.12,5.56) | 10.02(3.15,22.87) | 46.24(20.04,82.69) | 5.86(5.63,6.10) |
| Saudi Arabia | 3.26(1.68,5.59) | 4.95(3.43,6.61) | 0.16(-0.56,0.88) | 118.32(61.83,196.06) | 262.73(176.44,356.39) | 1.84(1.31,2.38) | 2.83(1.32,4.86) | 4.18(2.83,6.10) | -0.16(-1.04,0.72) | 98.06(47.69,164.47) | 234.52(159.71,321.37) | 1.86(1.14,2.59) |
| Senegal | 1.34(0.36,3.08) | 2.85(1.22,5.39) | 2.30(1.96,2.65) | 41.76(12.18,93.86) | 95.88(43.25,177.04) | 2.75(2.46,3.04) | 0.83(0.22,2.01) | 2.54(1.03,4.86) | 3.79(3.44,4.13) | 26.48(7.29,63.17) | 79.07(32.52,150.63) | 3.79(3.47,4.11) |
| Serbia | 2.34(1.27,3.70) | 3.82(2.53,5.32) | 2.13(1.76,2.51) | 107.86(57.79,169.04) | 187.08(121.77,258.12) | 2.04(1.78,2.31) | 2.51(1.26,4.02) | 3.51(2.29,4.91) | 1.46(1.11,1.82) | 92.14(46.56,148.05) | 146.19(91.76,204.47) | 1.75(1.51,1.99) |
| Seychelles | 1.67(0.84,2.69) | 3.26(1.69,5.03) | 1.75(1.37,2.14) | 83.76(41.06,135.79) | 192.33(98.95,306.83) | 2.45(2.13,2.78) | 1.58(0.73,2.56) | 3.46(1.71,5.34) | 2.50(2.17,2.82) | 69.68(31.71,113.47) | 182.67(92.18,288.74) | 3.10(2.75,3.46) |
| Sierra Leone | 0.76(0.19,1.86) | 1.43(0.51,2.91) | 2.46(2.28,2.64) | 18.73(4.51,46.70) | 38.98(14.48,78.73) | 2.86(2.65,3.06) | 0.46(0.11,1.16) | 1.48(0.53,3.17) | 4.73(4.51,4.96) | 11.21(2.78,28.15) | 38.79(14.06,82.68) | 4.98(4.75,5.22) |
| Singapore | 2.58(0.96,4.03) | 0.43(0.26,0.60) | -6.46(-7.37,-5.54) | 129.33(44.67,213.74) | 90.43(48.61,145.10) | -1.45(-1.71,-1.20) | 3.22(1.18,5.07) | 0.35(0.21,0.50) | -7.92(-8.98,-6.84) | 127.22(43.42,209.58) | 62.06(33.44,98.25) | -2.44(-2.67,-2.22) |
| Slovakia | 2.49(1.44,3.50) | 1.45(0.88,2.07) | -1.69(-1.80,-1.57) | 114.26(65.03,168.68) | 101.18(61.85,149.04) | -0.46(-0.56,-0.37) | 2.30(1.26,3.28) | 1.21(0.74,1.71) | -2.20(-2.32,-2.09) | 95.65(52.17,141.60) | 81.88(49.25,120.23) | -0.60(-0.66,-0.54) |
| Slovenia | 1.68(0.93,2.51) | 1.35(0.86,1.98) | -2.97(-3.98,-1.96) | 89.73(47.33,136.89) | 90.92(53.53,135.00) | -1.10(-1.60,-0.59) | 1.73(0.93,2.60) | 0.83(0.50,1.22) | -4.93(-5.90,-3.95) | 77.39(39.62,118.70) | 65.86(39.34,97.39) | -1.64(-2.05,-1.23) |
| Solomon Islands | 0.72(0.12,2.46) | 2.99(0.75,7.90) | 4.77(4.63,4.92) | 23.78(3.83,77.33) | 100.86(25.38,263.27) | 4.91(4.78,5.03) | 0.54(0.08,1.75) | 2.24(0.55,6.11) | 4.70(4.46,4.94) | 17.29(2.82,57.84) | 70.50(17.32,192.97) | 4.69(4.48,4.91) |
| Somalia | 0.40(0.05,1.35) | 0.55(0.09,1.74) | 1.50(1.18,1.82) | 10.95(1.47,35.20) | 15.85(2.49,47.32) | 1.66(1.34,1.99) | 0.20(0.02,0.63) | 0.28(0.04,0.87) | 1.85(1.50,2.20) | 5.53(0.70,17.87) | 8.08(1.18,25.31) | 1.85(1.52,2.18) |
| South Africa | 5.56(3.77,7.75) | 12.12(8.31,16.08) | 3.16(2.72,3.59) | 159.87(105.95,219.22) | 330.24(223.14,439.97) | 2.91(2.55,3.28) | 4.93(3.21,6.91) | 10.88(7.35,14.27) | 3.35(2.89,3.80) | 139.94(91.01,197.87) | 301.19(198.75,399.26) | 3.20(2.82,3.58) |
| South Sudan | 1.09(0.30,2.51) | 1.89(0.69,3.80) | 2.23(2.10,2.36) | 28.44(7.93,66.66) | 53.10(20.09,103.85) | 2.47(2.34,2.59) | 0.60(0.16,1.37) | 1.00(0.36,1.97) | 2.19(2.03,2.36) | 16.00(4.37,38.38) | 28.88(10.52,56.39) | 2.43(2.27,2.58) |
| Spain | 1.75(0.65,3.06) | 0.74(0.42,1.15) | -2.98(-3.38,-2.57) | 69.86(25.24,128.08) | 52.71(26.77,87.13) | -1.48(-1.98,-0.98) | 2.05(0.78,3.62) | 0.62(0.33,0.98) | -4.36(-4.69,-4.03) | 67.36(24.26,124.20) | 41.26(21.12,69.27) | -1.70(-2.02,-1.37) |
| Sri Lanka | 1.82(0.72,3.48) | 7.43(4.04,11.65) | 6.14(5.25,7.03) | 61.39(23.93,119.52) | 242.07(137.67,368.68) | 5.69(4.96,6.42) | 0.99(0.37,1.99) | 5.41(2.79,8.84) | 6.55(5.79,7.32) | 36.32(13.45,73.26) | 178.23(94.17,283.88) | 6.09(5.48,6.71) |
| Sudan | 0.36(0.10,0.85) | 2.30(1.13,4.04) | 7.23(6.87,7.58) | 17.28(4.63,39.13) | 131.87(72.85,204.43) | 7.83(7.60,8.05) | 0.33(0.08,0.84) | 2.17(1.07,3.70) | 7.50(7.15,7.85) | 13.37(3.52,33.26) | 111.12(60.40,178.13) | 8.23(7.96,8.51) |
| Suriname | 3.89(1.20,6.85) | 7.35(3.46,11.52) | 2.20(1.83,2.57) | 152.89(48.04,275.00) | 320.77(153.97,508.47) | 2.73(2.47,2.99) | 3.49(1.00,6.65) | 5.92(2.83,9.36) | 1.99(1.70,2.28) | 127.25(35.51,249.75) | 263.94(120.57,427.13) | 2.76(2.54,2.97) |
| Sweden | 0.80(0.13,1.67) | 0.29(0.07,0.65) | -3.47(-4.08,-2.85) | 30.61(5.20,66.28) | 13.98(3.08,31.92) | -2.55(-3.08,-2.00) | 0.60(0.10,1.23) | 0.19(0.05,0.43) | -3.84(-4.38,-3.29) | 23.02(3.94,49.26) | 10.00(2.24,23.28) | -2.66(-3.18,-2.14) |
| Switzerland | 2.09(0.96,3.31) | 0.67(0.37,1.06) | -4.30(-4.67,-3.92) | 70.74(31.79,115.01) | 40.21(20.98,66.60) | -2.11(-2.34,-1.87) | 1.96(0.87,3.10) | 0.47(0.26,0.74) | -5.43(-5.74,-5.13) | 60.21(26.83,98.13) | 30.90(15.82,52.10) | -2.35(-2.59,-2.11) |
| Syrian Arab Republic | 2.79(1.74,4.02) | 2.92(1.93,4.27) | -0.51(-0.83,-0.19) | 124.11(80.69,172.84) | 174.02(110.76,245.67) | 0.78(0.61,0.95) | 4.07(2.44,6.02) | 4.19(2.84,6.01) | -0.72(-1.13,-0.31) | 144.24(93.41,205.05) | 190.06(124.23,269.49) | 0.39(0.14,0.64) |
| Taiwan (Province of China) | 3.62(1.98,5.57) | 5.31(3.56,7.50) | 0.30(-0.48,1.08) | 119.44(63.23,188.95) | 190.19(126.24,265.73) | 0.81(0.19,1.44) | 4.47(2.14,7.22) | 4.39(2.86,6.18) | -1.35(-2.21,-0.49) | 130.35(62.15,216.22) | 147.39(96.95,206.97) | -0.54(-1.17,0.10) |
| Tajikistan | 0.89(0.27,1.86) | 5.09(2.59,7.99) | 6.66(6.10,7.21) | 38.65(11.93,79.95) | 192.58(100.52,293.68) | 5.92(5.48,6.36) | 0.57(0.18,1.22) | 3.98(1.81,6.47) | 7.63(7.08,8.18) | 26.56(8.13,57.98) | 158.73(77.82,250.64) | 6.88(6.46,7.29) |
| Thailand | 2.35(1.09,4.03) | 3.03(1.93,4.46) | 0.05(-0.33,0.44) | 84.16(39.91,141.32) | 146.72(94.60,202.91) | 1.14(0.85,1.44) | 2.34(1.03,4.14) | 3.06(1.92,4.47) | 0.11(-0.22,0.45) | 73.22(32.99,127.74) | 128.50(82.81,181.30) | 1.12(0.78,1.47) |
| Timor-Leste | 0.24(0.05,0.68) | 0.93(0.36,1.89) | 6.06(5.31,6.82) | 8.52(1.74,25.23) | 40.77(15.96,82.01) | 6.65(5.95,7.35) | 0.29(0.06,0.83) | 0.95(0.35,1.93) | 5.32(4.62,6.02) | 8.35(1.69,24.42) | 32.88(12.36,67.34) | 5.85(5.18,6.52) |
| Togo | 1.02(0.30,2.35) | 2.51(1.09,4.62) | 2.97(2.59,3.35) | 26.89(8.01,60.45) | 69.96(29.96,125.94) | 3.16(2.80,3.52) | 0.68(0.20,1.52) | 1.72(0.73,3.14) | 3.22(2.94,3.51) | 17.58(5.31,38.98) | 46.15(20.13,84.50) | 3.29(2.99,3.60) |
| Tokelau | 2.29(0.02,7.68) | 2.87(0.01,8.54) | 0.38(0.12,0.64) | 72.65(0.81,238.46) | 98.95(0.26,282.16) | 0.67(0.41,0.92) | 4.84(0.05,16.59) | 5.54(0.02,15.57) | -0.03(-0.29,0.24) | 138.86(1.52,467.90) | 163.67(0.47,463.91) | 0.08(-0.18,0.34) |
| Tonga | 2.25(0.51,6.26) | 6.99(1.88,15.62) | 3.85(3.24,4.46) | 65.02(15.42,180.79) | 206.55(56.96,454.20) | 3.93(3.41,4.44) | 2.67(0.60,7.66) | 5.60(1.38,13.59) | 2.38(2.18,2.58) | 74.30(16.02,213.55) | 165.49(39.91,398.54) | 2.51(2.32,2.69) |
| Trinidad and Tobago | 19.09(4.61,30.98) | 18.47(7.65,30.58) | -0.68(-0.94,-0.41) | 571.77(141.23,949.90) | 580.40(241.85,926.95) | -0.48(-0.72,-0.25) | 19.47(4.75,31.96) | 15.23(6.42,24.42) | -1.35(-1.58,-1.11) | 575.14(142.75,957.06) | 481.42(200.95,756.88) | -1.14(-1.38,-0.91) |
| Tunisia | 1.74(1.03,2.51) | 3.29(2.04,4.99) | 2.36(2.15,2.57) | 100.03(58.58,149.00) | 218.50(139.12,309.34) | 2.92(2.56,3.28) | 1.78(1.01,3.01) | 2.97(1.87,4.50) | 1.92(1.64,2.21) | 81.29(47.18,121.42) | 178.46(115.70,255.46) | 2.69(2.43,2.95) |
| Turkey | 5.32(3.32,7.75) | 4.13(2.71,5.81) | -0.54(-0.82,-0.27) | 162.50(106.79,228.17) | 170.30(113.81,233.37) | 0.83(0.51,1.16) | 6.71(4.23,9.45) | 4.77(3.08,6.56) | -0.98(-1.34,-0.63) | 181.46(115.58,252.21) | 172.42(114.27,238.38) | 0.45(0.05,0.85) |
| Turkmenistan | 1.78(0.80,2.70) | 3.17(1.86,4.72) | 1.15(0.71,1.59) | 82.24(35.85,127.14) | 155.84(92.80,226.84) | 1.71(1.42,1.99) | 1.56(0.68,2.38) | 2.94(1.75,4.40) | 1.49(1.05,1.94) | 77.90(33.39,119.85) | 154.21(92.51,223.86) | 2.07(1.78,2.36) |
| Tuvalu | 1.16(0.24,3.62) | 3.45(0.83,9.01) | 3.24(2.83,3.65) | 34.79(7.12,107.06) | 111.08(28.36,289.37) | 3.54(3.17,3.90) | 1.06(0.21,3.38) | 3.94(0.93,10.37) | 4.02(3.58,4.46) | 30.69(6.11,97.44) | 116.08(28.76,307.43) | 4.12(3.74,4.51) |
| Uganda | 0.86(0.17,2.43) | 2.89(1.19,5.69) | 4.71(4.46,4.96) | 22.71(4.73,63.93) | 80.75(33.93,153.98) | 4.93(4.68,5.19) | 0.35(0.07,0.95) | 1.13(0.48,2.16) | 4.72(4.48,4.97) | 9.90(2.11,25.86) | 34.38(15.02,64.57) | 5.05(4.81,5.29) |
| Ukraine | 0.61(0.31,0.92) | 0.48(0.26,0.74) | -1.73(-2.09,-1.38) | 46.97(23.02,75.82) | 48.02(25.15,76.07) | -0.16(-0.44,0.12) | 0.55(0.27,0.86) | 0.35(0.19,0.56) | -2.49(-2.86,-2.12) | 43.88(20.04,71.86) | 40.16(20.53,64.85) | -0.49(-0.77,-0.20) |
| United Arab Emirates | 15.45(9.98,22.20) | 13.21(8.51,19.13) | -0.80(-1.20,-0.40) | 406.86(272.73,558.90) | 441.28(307.53,610.72) | 0.13(-0.12,0.39) | 19.71(12.41,28.71) | 11.86(7.90,16.95) | -1.21(-2.60,0.19) | 466.47(315.14,646.21) | 401.82(277.04,556.95) | -0.14(-1.06,0.78) |
| United Kingdom | 1.39(0.62,2.22) | 0.42(0.23,0.67) | -4.55(-4.72,-4.37) | 62.41(26.40,102.91) | 59.77(30.21,99.81) | 0.00(-0.21,0.21) | 1.05(0.47,1.67) | 0.32(0.18,0.50) | -4.38(-4.51,-4.24) | 47.30(20.00,77.13) | 43.23(21.77,71.30) | -0.18(-0.34,-0.03) |
| United Republic of Tanzania | 0.68(0.19,1.59) | 1.85(0.79,3.54) | 3.85(3.63,4.07) | 16.50(4.62,38.53) | 49.11(21.30,91.51) | 4.30(4.06,4.54) | 0.38(0.11,0.90) | 0.96(0.44,1.76) | 3.79(3.57,4.00) | 9.23(2.59,21.39) | 26.52(12.03,47.78) | 4.37(4.14,4.60) |
| United States of America | 1.65(0.61,2.87) | 0.89(0.41,1.59) | -2.92(-3.66,-2.18) | 79.22(27.88,140.95) | 53.79(23.27,96.41) | -1.26(-1.66,-0.85) | 1.52(0.57,2.61) | 0.61(0.27,1.08) | -4.01(-4.74,-3.28) | 67.17(23.92,118.36) | 38.75(17.01,69.54) | -1.84(-2.18,-1.49) |
| United States Virgin Islands | 2.46(0.28,6.14) | 3.70(1.59,6.33) | 1.52(0.99,2.04) | 87.20(9.75,218.85) | 141.86(59.92,241.12) | 1.66(1.22,2.11) | 3.37(0.42,8.51) | 2.78(1.20,4.74) | -1.09(-1.45,-0.72) | 99.37(12.03,246.79) | 106.03(44.96,179.70) | -0.22(-0.59,0.15) |
| Uruguay | 1.34(0.32,2.83) | 1.41(0.63,2.40) | 0.22(0.01,0.43) | 32.27(7.57,66.78) | 51.38(22.90,87.46) | 1.52(1.07,1.98) | 1.10(0.25,2.43) | 0.99(0.43,1.69) | -0.45(-0.71,-0.19) | 26.23(6.06,56.82) | 34.70(15.19,59.97) | 0.89(0.50,1.29) |
| Uzbekistan | 1.21(0.48,1.99) | 7.21(4.65,10.21) | 5.93(5.23,6.65) | 56.52(22.90,95.17) | 265.90(167.60,378.40) | 5.24(4.74,5.74) | 0.82(0.30,1.43) | 6.23(3.97,8.88) | 6.92(6.15,7.70) | 38.40(13.66,68.39) | 227.77(144.29,323.02) | 6.21(5.67,6.74) |
| Vanuatu | 0.80(0.15,2.27) | 2.41(0.63,5.87) | 3.83(3.69,3.98) | 28.02(5.32,78.18) | 87.04(22.94,212.56) | 3.87(3.74,3.99) | 0.65(0.13,1.93) | 2.18(0.53,5.48) | 4.06(3.85,4.27) | 20.42(4.07,60.26) | 69.48(17.57,171.64) | 4.07(3.87,4.27) |
| Venezuela (Bolivarian Republic of) | 4.74(2.18,7.42) | 7.38(4.35,10.84) | 1.44(1.16,1.73) | 180.30(81.51,287.00) | 278.39(167.98,395.79) | 1.54(1.35,1.73) | 5.06(2.22,8.03) | 5.97(3.62,8.79) | 0.38(0.12,0.64) | 173.63(77.87,276.17) | 231.14(140.63,335.72) | 0.93(0.75,1.12) |
| Viet Nam | 0.94(0.31,2.00) | 3.82(2.19,5.64) | 5.69(5.26,6.12) | 28.78(9.74,61.74) | 124.54(71.57,182.99) | 5.84(5.44,6.23) | 1.03(0.34,2.28) | 3.29(1.78,5.09) | 4.49(4.25,4.72) | 27.18(8.90,59.82) | 97.19(54.28,146.76) | 4.95(4.67,5.22) |
| Yemen | 0.35(0.08,0.87) | 1.63(0.78,2.71) | 6.17(5.87,6.48) | 15.08(3.27,37.41) | 85.11(42.88,134.65) | 6.79(6.51,7.07) | 0.32(0.06,0.87) | 1.92(0.81,3.53) | 7.31(6.99,7.64) | 12.10(2.50,31.02) | 84.76(39.56,144.70) | 7.71(7.38,8.04) |
| Zambia | 1.25(0.37,2.67) | 3.94(1.84,6.88) | 4.08(3.92,4.24) | 32.68(9.91,70.60) | 107.87(50.61,186.26) | 4.23(4.06,4.40) | 0.70(0.22,1.58) | 1.68(0.78,2.96) | 3.18(2.90,3.47) | 19.16(5.83,42.94) | 48.93(22.68,85.74) | 3.36(3.07,3.65) |
| Zimbabwe | 1.18(0.45,2.21) | 2.65(1.09,4.82) | 2.18(1.41,2.95) | 39.10(14.99,72.83) | 87.87(38.15,160.47) | 2.35(1.67,3.03) | 1.23(0.48,2.30) | 2.55(1.08,4.65) | 3.43(2.94,3.91) | 34.61(13.74,64.10) | 75.90(32.61,139.12) | 3.39(3.02,3.76) |

**Supplementary Table 9. Comparative Analysis of Age-standardized of type 2 diabetes mellitus death and DALY Rates attributed to ambient Particulate Matter Pollution by Countries in Relation to Sex and SDI, 1990 vs. 2019**

| **Country** | **Region** | **Sex** | **SDI_2019** | **Age-standardized Deaths Rate 1990** | **Age-standardized Deaths Rate 2019** | **Age-standardized DALYs Rate 1990** | **Age-standardized DALYs Rate 2019** |
| --- | --- | --- | --- | --- | --- | --- | --- |
| Afghanistan | North Africa and Middle East | both | 0.343 | 0.60 | 2.32 | 21.57 | 92.54 |
| Albania | Central Europe | both | 0.681 | 0.30 | 0.43 | 19.44 | 43.95 |
| Algeria | North Africa and Middle East | both | 0.652 | 2.78 | 3.88 | 104.36 | 201.86 |
| American Samoa | Oceania | both | 0.712 | 2.89 | 4.24 | 96.90 | 159.88 |
| Andorra | Western Europe | both | 0.894 | 0.92 | 0.43 | 35.86 | 29.89 |
| Angola | Central sub-Saharan Africa | both | 0.47 | 0.77 | 3.72 | 23.56 | 117.01 |
| Antigua and Barbuda | Caribbean | both | 0.743 | 7.73 | 8.71 | 231.89 | 283.26 |
| Argentina | Southern Latin America | both | 0.708 | 2.22 | 2.17 | 67.32 | 87.05 |
| Armenia | Central Asia | both | 0.689 | 2.49 | 5.83 | 94.05 | 216.39 |
| Australia | Australasia | both | 0.839 | 0.62 | 0.40 | 20.25 | 19.18 |
| Austria | Western Europe | both | 0.849 | 2.06 | 1.23 | 63.96 | 54.51 |
| Azerbaijan | Central Asia | both | 0.683 | 1.04 | 4.03 | 48.54 | 169.38 |
| Bahrain | North Africa and Middle East | both | 0.751 | 17.14 | 30.34 | 455.17 | 770.18 |
| Bangladesh | South Asia | both | 0.483 | 0.89 | 3.15 | 24.63 | 90.83 |
| Barbados | Caribbean | both | 0.742 | 11.44 | 10.47 | 312.14 | 318.00 |
| Belarus | Eastern Europe | both | 0.745 | 0.73 | 0.32 | 51.43 | 40.53 |
| Belgium | Western Europe | both | 0.851 | 1.86 | 0.72 | 67.24 | 53.02 |
| Belize | Caribbean | both | 0.603 | 2.81 | 7.51 | 81.90 | 244.66 |
| Benin | Western sub-Saharan Africa | both | 0.352 | 0.61 | 1.76 | 17.62 | 54.43 |
| Bermuda | Caribbean | both | 0.813 | 2.28 | 0.87 | 68.81 | 40.21 |
| Bhutan | South Asia | both | 0.455 | 0.39 | 3.15 | 13.07 | 99.97 |
| Bolivia (Plurinational State of) | Andean Latin America | both | 0.566 | 3.47 | 5.84 | 101.93 | 172.58 |
| Bosnia and Herzegovina | Central Europe | both | 0.718 | 1.26 | 6.03 | 54.56 | 211.79 |
| Botswana | Southern sub-Saharan Africa | both | 0.634 | 2.59 | 11.46 | 67.93 | 300.19 |
| Brazil | Tropical Latin America | both | 0.64 | 2.14 | 2.42 | 76.26 | 90.46 |
| Brunei Darussalam | High-income Asia Pacific | both | 0.823 | 6.28 | 4.27 | 171.12 | 137.28 |
| Bulgaria | Central Europe | both | 0.764 | 2.26 | 2.06 | 95.30 | 109.03 |
| Burkina Faso | Western sub-Saharan Africa | both | 0.257 | 0.63 | 1.26 | 16.92 | 38.11 |
| Burundi | Eastern Sub-Saharan Africa | both | 0.284 | 0.80 | 0.91 | 21.80 | 26.40 |
| Cambodia | Southeast Asia | both | 0.469 | 0.60 | 1.49 | 17.60 | 54.68 |
| Cameroon | Western sub-Saharan Africa | both | 0.49 | 2.01 | 5.73 | 51.97 | 156.04 |
| Canada | High-income North America | both | 0.873 | 1.03 | 0.46 | 27.78 | 20.20 |
| Cabo Verde | Western sub-Saharan Africa | both | 0.525 | 0.39 | 5.63 | 18.91 | 187.02 |
| Central African Republic | Central sub-Saharan Africa | both | 0.274 | 1.09 | 1.68 | 33.35 | 56.00 |
| Chad | Western sub-Saharan Africa | both | 0.238 | 0.43 | 1.17 | 12.84 | 36.29 |
| Chile | Southern Latin America | both | 0.759 | 1.82 | 2.72 | 67.85 | 124.48 |
| China | East Asia | both | 0.686 | 0.65 | 1.74 | 32.64 | 89.70 |
| Colombia | Central Latin America | both | 0.633 | 1.91 | 1.96 | 90.68 | 133.83 |
| Comoros | Eastern Sub-Saharan Africa | both | 0.455 | 0.51 | 1.24 | 13.04 | 33.78 |
| Congo | Central sub-Saharan Africa | both | 0.568 | 2.49 | 6.46 | 71.80 | 194.89 |
| Costa Rica | Central Latin America | both | 0.68 | 1.16 | 1.61 | 61.69 | 127.47 |
| Côte d'Ivoire | Western sub-Saharan Africa | both | 0.408 | 1.22 | 2.96 | 33.41 | 87.34 |
| Croatia | Central Europe | both | 0.794 | 1.92 | 1.75 | 91.67 | 108.11 |
| Cuba | Caribbean | both | 0.668 | 2.90 | 1.46 | 134.63 | 127.76 |
| Cyprus | Western Europe | both | 0.841 | 9.44 | 3.86 | 189.20 | 113.96 |
| Czechia | Central Europe | both | 0.557 | 2.23 | 2.15 | 122.69 | 151.50 |
| Democratic Republic of the Congo | Central sub-Saharan Africa | both | 0.568 | 1.11 | 1.54 | 31.07 | 52.09 |
| Denmark | Western Europe | both | 0.89 | 1.38 | 0.94 | 44.87 | 35.94 |
| Djibouti | Eastern Sub-Saharan Africa | both | 0.459 | 1.46 | 6.77 | 39.80 | 185.43 |
| Dominica | Caribbean | both | 0.729 | 5.11 | 8.78 | 148.59 | 311.90 |
| Dominican Republic | Caribbean | both | 0.592 | 0.84 | 3.84 | 27.87 | 139.81 |
| Ecuador | Andean Latin America | both | 0.64 | 2.01 | 5.38 | 68.82 | 180.64 |
| Egypt | North Africa and Middle East | both | 0.658 | 4.83 | 8.12 | 148.38 | 293.51 |
| El Salvador | Central Latin America | both | 0.573 | 0.86 | 5.19 | 38.04 | 214.04 |
| Equatorial Guinea | Central sub-Saharan Africa | both | 0.685 | 0.93 | 10.44 | 28.65 | 294.69 |
| Eritrea | Eastern Sub-Saharan Africa | both | 0.396 | 0.92 | 2.91 | 27.07 | 83.41 |
| Estonia | Eastern Europe | both | 0.835 | 0.23 | 0.17 | 20.55 | 16.03 |
| Ethiopia | Eastern Sub-Saharan Africa | both | 0.343 | 0.63 | 1.29 | 17.31 | 34.01 |
| Micronesia (Federated States of) | Oceania | both | 0.58 | 2.14 | 9.03 | 61.88 | 262.32 |
| Fiji | Oceania | both | 0.664 | 4.04 | 18.21 | 111.26 | 486.81 |
| Finland | Western Europe | both | 0.856 | 0.45 | 0.09 | 26.43 | 13.61 |
| France | Western Europe | both | 0.834 | 1.20 | 0.75 | 34.74 | 29.01 |
| Gabon | Central sub-Saharan Africa | both | 0.656 | 4.03 | 13.16 | 113.59 | 379.37 |
| Georgia | Central Asia | both | 0.702 | 0.92 | 2.46 | 45.01 | 121.40 |
| Germany | Western Europe | both | 0.898 | 2.57 | 1.05 | 92.89 | 65.19 |
| Ghana | Western sub-Saharan Africa | both | 0.557 | 1.55 | 5.66 | 44.51 | 169.55 |
| Greece | Western Europe | both | 0.794 | 1.20 | 0.66 | 54.50 | 59.45 |
| Greenland | High-income North America | both | 0.761 | 0.69 | 0.38 | 19.65 | 19.41 |
| Grenada | Caribbean | both | 0.669 | 5.23 | 11.78 | 157.48 | 379.29 |
| Guam | Oceania | both | 0.813 | 2.75 | 1.58 | 83.64 | 78.77 |
| Guatemala | Central Latin America | both | 0.526 | 0.67 | 5.57 | 30.61 | 201.32 |
| Guinea | Western sub-Saharan Africa | both | 0.325 | 0.61 | 1.57 | 16.60 | 45.68 |
| Guinea-Bissau | Western sub-Saharan Africa | both | 0.355 | 1.02 | 2.32 | 28.59 | 67.23 |
| Guyana | Caribbean | both | 0.618 | 7.65 | 13.54 | 241.21 | 449.35 |
| Haiti | Caribbean | both | 0.432 | 1.14 | 1.91 | 34.63 | 68.17 |
| Honduras | Central Latin America | both | 0.496 | 0.37 | 1.29 | 25.12 | 80.53 |
| Hungary | Central Europe | both | 0.791 | 1.64 | 1.60 | 82.81 | 102.34 |
| Iceland | Western Europe | both | 0.869 | 0.24 | 0.12 | 12.04 | 11.05 |
| India | South Asia | both | 0.566 | 1.34 | 4.25 | 45.50 | 165.86 |
| Indonesia | Southeast Asia | both | 0.66 | 1.82 | 5.43 | 58.29 | 168.79 |
| Iran (Islamic Republic of) | North Africa and Middle East | both | 0.67 | 2.52 | 4.90 | 100.94 | 212.37 |
| Iraq | North Africa and Middle East | both | 0.671 | 8.25 | 10.48 | 257.22 | 369.95 |
| Ireland | Western Europe | both | 0.867 | 1.11 | 0.38 | 30.70 | 23.75 |
| Israel | Western Europe | both | 0.803 | 2.99 | 3.47 | 89.78 | 112.98 |
| Italy | Western Europe | both | 0.801 | 3.27 | 1.61 | 99.92 | 79.15 |
| Jamaica | Caribbean | both | 0.684 | 3.12 | 9.24 | 88.41 | 275.56 |
| Japan | High-income Asia Pacific | both | 0.87 | 0.69 | 0.24 | 37.48 | 38.65 |
| Jordan | North Africa and Middle East | both | 0.731 | 12.81 | 8.55 | 324.76 | 273.75 |
| Kazakhstan | Central Asia | both | 0.723 | 0.65 | 2.27 | 45.03 | 125.35 |
| Kenya | Eastern Sub-Saharan Africa | both | 0.508 | 0.66 | 1.81 | 17.92 | 52.69 |
| Kiribati | Oceania | both | 0.527 | 2.07 | 4.59 | 63.06 | 139.36 |
| Kuwait | North Africa and Middle East | both | 0.851 | 6.78 | 4.43 | 251.87 | 270.94 |
| Kyrgyzstan | Central Asia | both | 0.596 | 0.41 | 0.81 | 22.86 | 54.88 |
| Lao People's Democratic Republic | Southeast Asia | both | 0.49 | 0.69 | 1.60 | 22.52 | 58.48 |
| Latvia | Eastern Europe | both | 0.82 | 0.66 | 0.82 | 45.23 | 52.07 |
| Lebanon | North Africa and Middle East | both | 0.708 | 3.29 | 3.32 | 128.61 | 205.72 |
| Lesotho | Southern sub-Saharan Africa | both | 0.507 | 1.81 | 8.59 | 47.75 | 219.80 |
| Liberia | Western sub-Saharan Africa | both | 0.37 | 0.80 | 1.80 | 23.82 | 58.92 |
| Libya | North Africa and Middle East | both | 0.709 | 2.19 | 4.07 | 106.01 | 251.70 |
| Lithuania | Eastern Europe | both | 0.843 | 0.47 | 0.34 | 36.42 | 31.37 |
| Luxembourg | Western Europe | both | 0.895 | 1.44 | 0.52 | 42.09 | 50.00 |
| North Macedonia | Central Europe | both | 0.744 | 2.88 | 6.08 | 113.79 | 227.88 |
| Madagascar | Eastern Sub-Saharan Africa | both | 0.396 | 0.36 | 0.76 | 10.07 | 22.79 |
| Malawi | Eastern Sub-Saharan Africa | both | 0.384 | 0.50 | 0.98 | 13.52 | 29.96 |
| Malaysia | Southeast Asia | both | 0.737 | 4.55 | 1.94 | 163.02 | 119.19 |
| Maldives | Southeast Asia | both | 0.562 | 1.03 | 1.47 | 30.97 | 60.33 |
| Mali | Western sub-Saharan Africa | both | 0.263 | 0.56 | 1.27 | 15.59 | 37.45 |
| Malta | Western Europe | both | 0.801 | 3.27 | 1.43 | 97.60 | 75.97 |
| Marshall Islands | Oceania | both | 0.544 | 1.53 | 4.66 | 52.23 | 173.72 |
| Mauritania | Western sub-Saharan Africa | both | 0.496 | 1.87 | 4.42 | 47.89 | 114.35 |
| Mauritius | Southeast Asia | both | 0.705 | 4.44 | 14.28 | 155.40 | 430.04 |
| Mexico | Central Latin America | both | 0.649 | 8.62 | 9.18 | 286.77 | 323.11 |
| Republic of Moldova | Eastern Europe | both | 0.696 | 0.61 | 0.54 | 41.02 | 55.82 |
| Mongolia | Central Asia | both | 0.606 | 0.26 | 0.76 | 13.00 | 46.75 |
| Montenegro | Central Europe | both | 0.791 | 1.78 | 2.08 | 92.84 | 128.45 |
| Morocco | North Africa and Middle East | both | 0.548 | 1.11 | 4.63 | 46.52 | 208.26 |
| Mozambique | Eastern Sub-Saharan Africa | both | 0.307 | 0.29 | 0.89 | 7.88 | 26.00 |
| Myanmar | Southeast Asia | both | 0.521 | 1.59 | 3.95 | 47.39 | 124.47 |
| Namibia | Southern sub-Saharan Africa | both | 0.612 | 2.62 | 6.76 | 69.05 | 179.05 |
| Nepal | South Asia | both | 0.422 | 0.37 | 2.13 | 15.68 | 89.43 |
| Netherlands | Western Europe | both | 0.883 | 2.73 | 0.96 | 81.43 | 44.79 |
| New Zealand | Australasia | both | 0.84 | 0.49 | 0.28 | 17.11 | 13.63 |
| Nicaragua | Central Latin America | both | 0.517 | 0.72 | 3.54 | 28.26 | 125.68 |
| Niger | Western sub-Saharan Africa | both | 0.162 | 0.50 | 0.96 | 12.56 | 26.37 |
| Nigeria | Western sub-Saharan Africa | both | 0.515 | 1.33 | 4.18 | 33.83 | 104.56 |
| Democratic People's Republic of Korea | East Asia | both | 0.558 | 0.65 | 1.21 | 25.64 | 60.23 |
| Northern Mariana Islands | Oceania | both | 0.771 | 3.49 | 3.75 | 109.72 | 132.29 |
| Norway | Western Europe | both | 0.913 | 0.66 | 0.23 | 36.19 | 18.14 |
| Oman | North Africa and Middle East | both | 0.783 | 5.32 | 13.41 | 149.77 | 371.16 |
| Pakistan | South Asia | both | 0.449 | 0.99 | 6.35 | 33.96 | 198.63 |
| Palestine | North Africa and Middle East | both | 0.588 | 5.43 | 14.43 | 141.02 | 398.78 |
| Panama | Central Latin America | both | 0.686 | 1.32 | 3.06 | 58.12 | 136.01 |
| Papua New Guinea | Oceania | both | 0.394 | 1.23 | 2.56 | 36.55 | 79.05 |
| Paraguay | Tropical Latin America | both | 0.638 | 0.88 | 3.33 | 29.00 | 102.27 |
| Peru | Andean Latin America | both | 0.648 | 1.25 | 2.39 | 45.24 | 101.38 |
| Philippines | Southeast Asia | both | 0.623 | 2.78 | 3.51 | 77.17 | 113.08 |
| Poland | Central Europe | both | 0.802 | 1.91 | 1.79 | 100.46 | 121.60 |
| Portugal | Western Europe | both | 0.743 | 1.83 | 0.96 | 60.09 | 45.00 |
| Puerto Rico | Caribbean | both | 0.814 | 2.43 | 1.90 | 80.64 | 78.95 |
| Qatar | North Africa and Middle East | both | 0.83 | 26.96 | 29.68 | 612.07 | 722.09 |
| Romania | Central Europe | both | 0.76 | 0.75 | 0.79 | 44.67 | 63.70 |
| Russian Federation | Eastern Europe | both | 0.805 | 0.58 | 0.74 | 36.93 | 38.81 |
| Rwanda | Eastern Sub-Saharan Africa | both | 0.429 | 1.17 | 1.84 | 31.32 | 50.00 |
| Saint Lucia | Caribbean | both | 0.67 | 6.90 | 9.97 | 209.13 | 369.05 |
| Saint Vincent and the Grenadines | Caribbean | both | 0.627 | 6.97 | 12.82 | 196.97 | 413.60 |
| Samoa | Oceania | both | 0.641 | 2.34 | 3.83 | 69.72 | 124.13 |
| Sao Tome and Principe | Central sub-Saharan Africa | both | 0.67 | 0.32 | 1.33 | 13.54 | 60.39 |
| Saudi Arabia | North Africa and Middle East | both | 0.805 | 3.05 | 4.64 | 109.49 | 251.53 |
| Senegal | Western sub-Saharan Africa | both | 0.389 | 1.08 | 2.68 | 34.15 | 87.09 |
| Serbia | Central Europe | both | 0.767 | 2.46 | 3.69 | 100.06 | 166.21 |
| Seychelles | Southeast Asia | both | 0.724 | 1.63 | 3.43 | 76.09 | 188.11 |
| Sierra Leone | Western sub-Saharan Africa | both | 0.347 | 0.61 | 1.46 | 15.06 | 38.90 |
| Singapore | High-income Asia Pacific | both | 0.861 | 2.93 | 0.39 | 128.25 | 76.38 |
| Slovakia | Central Europe | both | 0.812 | 2.40 | 1.33 | 104.40 | 91.09 |
| Slovenia | Central Europe | both | 0.84 | 1.74 | 1.05 | 83.39 | 77.75 |
| Solomon Islands | Oceania | both | 0.407 | 0.64 | 2.62 | 20.88 | 85.95 |
| Somalia | Eastern Sub-Saharan Africa | both | 0.081 | 0.29 | 0.38 | 8.05 | 11.32 |
| South Africa | Southern sub-Saharan Africa | both | 0.678 | 5.21 | 11.41 | 148.70 | 313.84 |
| Republic of Korea | High-income Asia Pacific | both | 0.46 | 3.13 | 3.01 | 118.16 | 121.62 |
| South Sudan | Eastern Sub-Saharan Africa | both | 0.363 | 0.85 | 1.47 | 22.84 | 41.67 |
| Spain | Western Europe | both | 0.767 | 1.96 | 0.68 | 69.03 | 46.76 |
| Sri Lanka | Southeast Asia | both | 0.69 | 1.40 | 6.26 | 48.87 | 206.71 |
| Sudan | North Africa and Middle East | both | 0.515 | 0.34 | 2.24 | 15.40 | 122.32 |
| Suriname | Caribbean | both | 0.636 | 3.67 | 6.59 | 139.42 | 290.84 |
| Eswatini | Southern sub-Saharan Africa | both | 0.78 | 3.17 | 12.41 | 79.98 | 310.07 |
| Sweden | Western Europe | both | 0.872 | 0.69 | 0.24 | 26.58 | 11.95 |
| Switzerland | Western Europe | both | 0.929 | 2.04 | 0.56 | 65.44 | 35.41 |
| Syrian Arab Republic | North Africa and Middle East | both | 0.636 | 3.39 | 3.38 | 133.65 | 180.74 |
| Taiwan (Province of China) | East Asia | both | 0.515 | 4.02 | 4.84 | 124.13 | 168.29 |
| Tajikistan | Central Asia | both | 0.539 | 0.72 | 4.51 | 32.20 | 175.05 |
| United Republic of Tanzania | Eastern Sub-Saharan Africa | both | 0.902 | 0.52 | 1.36 | 12.68 | 37.12 |
| Thailand | Southeast Asia | both | 0.687 | 2.35 | 3.07 | 78.37 | 137.31 |
| Bahamas | Caribbean | both | 0.877 | 6.26 | 5.10 | 199.96 | 206.07 |
| Gambia | Western sub-Saharan Africa | both | 0.902 | 0.69 | 2.38 | 19.37 | 68.31 |
| Timor-Leste | Southeast Asia | both | 0.514 | 0.26 | 0.94 | 8.42 | 36.83 |
| Togo | Western sub-Saharan Africa | both | 0.417 | 0.83 | 2.03 | 21.95 | 56.26 |
| Tonga | Oceania | both | 0.636 | 2.46 | 6.20 | 69.63 | 184.50 |
| Trinidad and Tobago | Caribbean | both | 0.757 | 19.31 | 16.77 | 573.62 | 529.64 |
| Tunisia | North Africa and Middle East | both | 0.672 | 1.75 | 3.12 | 90.86 | 197.98 |
| Turkey | North Africa and Middle East | both | 0.748 | 6.11 | 4.51 | 172.98 | 172.00 |
| Turkmenistan | Central Asia | both | 0.67 | 1.66 | 3.04 | 80.14 | 155.01 |
| Uganda | Eastern Sub-Saharan Africa | both | 0.404 | 0.59 | 1.83 | 16.07 | 54.36 |
| Ukraine | Eastern Europe | both | 0.736 | 0.57 | 0.40 | 45.13 | 43.51 |
| United Arab Emirates | North Africa and Middle East | both | 0.88 | 17.35 | 12.77 | 433.46 | 430.73 |
| United Kingdom | Western Europe | both | 0.902 | 1.18 | 0.37 | 54.03 | 51.31 |
| United States of America | High-income North America | both | 0.618 | 1.57 | 0.74 | 72.66 | 45.80 |
| Uruguay | Southern Latin America | both | 0.697 | 1.21 | 1.17 | 29.01 | 42.09 |
| Uzbekistan | Central Asia | both | 0.631 | 0.99 | 6.67 | 46.57 | 245.07 |
| Vanuatu | Oceania | both | 0.485 | 0.73 | 2.30 | 24.57 | 78.57 |
| Venezuela (Bolivarian Republic of) | Central Latin America | both | 0.412 | 4.94 | 6.64 | 177.21 | 253.75 |
| Viet nam | Southeast Asia | both | 0.902 | 1.01 | 3.54 | 27.91 | 109.38 |
| United States Virgin Islands | Caribbean | both | 0.877 | 2.99 | 3.22 | 94.01 | 122.65 |
| Yemen | North Africa and Middle East | both | 0.412 | 0.33 | 1.77 | 13.46 | 84.92 |
| Zambia | Eastern Sub-Saharan Africa | both | 0.505 | 0.98 | 2.71 | 26.24 | 76.85 |
| Zimbabwe | Southern sub-Saharan Africa | both | 0.476 | 1.23 | 2.61 | 36.95 | 81.24 |
| Monaco | Western Europe | both | 0.902 | 0.26 | 0.33 | 17.69 | 37.59 |
| San Marino | Western Europe | both | 0.884 | 0.77 | 0.51 | 31.56 | 36.16 |
| Saint Kitts and Nevis | Caribbean | both | 0.746 | 3.68 | 3.93 | 109.40 | 136.63 |
| Cook Islands | Oceania | both | 0.764 | 4.20 | 4.72 | 111.25 | 142.11 |
| Nauru | Oceania | both | 0.618 | 2.99 | 5.38 | 83.05 | 153.59 |
| Niue | Oceania | both | 0.711 | 2.56 | 5.76 | 77.79 | 185.55 |
| Palau | Oceania | both | 0.738 | 3.67 | 5.51 | 109.04 | 172.82 |
| Tokelau | Oceania | both | 0.626 | 3.69 | 4.21 | 109.21 | 130.86 |
| Tuvalu | Oceania | both | 0.589 | 1.10 | 3.73 | 32.37 | 114.00 |
| Afghanistan | North Africa and Middle East | male | 0.343 | 0.49 | 1.52 | 19.19 | 75.37 |
| Albania | Central Europe | male | 0.681 | 0.37 | 0.49 | 23.36 | 50.25 |
| Algeria | North Africa and Middle East | male | 0.652 | 2.48 | 3.11 | 100.10 | 184.03 |
| American Samoa | Oceania | male | 0.712 | 3.58 | 4.64 | 118.74 | 174.39 |
| Andorra | Western Europe | male | 0.894 | 1.23 | 0.58 | 43.77 | 34.56 |
| Angola | Central sub-Saharan Africa | male | 0.47 | 1.18 | 5.68 | 34.45 | 170.50 |
| Antigua and Barbuda | Caribbean | male | 0.743 | 8.51 | 8.24 | 251.96 | 280.55 |
| Argentina | Southern Latin America | male | 0.708 | 2.63 | 2.72 | 77.35 | 101.79 |
| Armenia | Central Asia | male | 0.689 | 2.30 | 5.59 | 88.50 | 212.71 |
| Australia | Australasia | male | 0.839 | 0.74 | 0.50 | 23.51 | 22.60 |
| Austria | Western Europe | male | 0.849 | 2.08 | 1.52 | 68.11 | 63.54 |
| Azerbaijan | Central Asia | male | 0.683 | 1.22 | 4.08 | 54.14 | 169.04 |
| Bahrain | North Africa and Middle East | male | 0.751 | 18.44 | 29.77 | 486.85 | 774.07 |
| Bangladesh | South Asia | male | 0.483 | 1.07 | 3.16 | 29.28 | 95.57 |
| Barbados | Caribbean | male | 0.742 | 9.67 | 9.71 | 275.68 | 308.14 |
| Belarus | Eastern Europe | male | 0.745 | 0.71 | 0.39 | 51.07 | 43.01 |
| Belgium | Western Europe | male | 0.851 | 1.60 | 0.81 | 66.29 | 58.43 |
| Belize | Caribbean | male | 0.603 | 2.38 | 7.22 | 73.46 | 237.75 |
| Benin | Western sub-Saharan Africa | male | 0.352 | 0.79 | 2.09 | 22.42 | 64.51 |
| Bermuda | Caribbean | male | 0.813 | 2.55 | 1.11 | 80.39 | 48.00 |
| Bhutan | South Asia | male | 0.455 | 0.47 | 3.46 | 15.06 | 111.03 |
| Bolivia (Plurinational State of) | Andean Latin America | male | 0.566 | 3.02 | 5.19 | 95.99 | 168.68 |
| Bosnia and Herzegovina | Central Europe | male | 0.718 | 1.16 | 6.21 | 58.60 | 235.58 |
| Botswana | Southern sub-Saharan Africa | male | 0.634 | 3.13 | 13.22 | 82.61 | 343.10 |
| Brazil | Tropical Latin America | male | 0.64 | 1.99 | 2.54 | 78.82 | 99.39 |
| Brunei Darussalam | High-income Asia Pacific | male | 0.823 | 8.01 | 5.76 | 206.11 | 168.72 |
| Bulgaria | Central Europe | male | 0.764 | 2.33 | 2.32 | 102.81 | 123.35 |
| Burkina Faso | Western sub-Saharan Africa | male | 0.257 | 0.89 | 1.86 | 22.82 | 52.43 |
| Burundi | Eastern Sub-Saharan Africa | male | 0.284 | 1.12 | 1.20 | 30.23 | 34.16 |
| Cambodia | Southeast Asia | male | 0.469 | 0.72 | 1.82 | 21.43 | 68.44 |
| Cameroon | Western sub-Saharan Africa | male | 0.49 | 2.53 | 6.61 | 64.72 | 181.43 |
| Canada | High-income North America | male | 0.873 | 1.17 | 0.58 | 31.92 | 24.06 |
| Cabo Verde | Western sub-Saharan Africa | male | 0.525 | 0.47 | 5.64 | 23.15 | 196.43 |
| Central African Republic | Central sub-Saharan Africa | male | 0.274 | 1.79 | 2.63 | 52.49 | 83.84 |
| Chad | Western sub-Saharan Africa | male | 0.238 | 0.51 | 1.23 | 15.33 | 39.21 |
| Chile | Southern Latin America | male | 0.759 | 2.06 | 3.04 | 75.42 | 134.89 |
| China | East Asia | male | 0.686 | 0.72 | 2.05 | 36.09 | 101.57 |
| Colombia | Central Latin America | male | 0.633 | 1.72 | 1.94 | 96.10 | 146.85 |
| Comoros | Eastern Sub-Saharan Africa | male | 0.455 | 0.66 | 1.59 | 16.73 | 43.40 |
| Congo | Central sub-Saharan Africa | male | 0.568 | 3.95 | 8.05 | 110.02 | 238.59 |
| Costa Rica | Central Latin America | male | 0.68 | 1.05 | 1.71 | 67.83 | 147.52 |
| Côte d'Ivoire | Western sub-Saharan Africa | male | 0.408 | 1.59 | 3.38 | 42.63 | 99.00 |
| Croatia | Central Europe | male | 0.794 | 1.97 | 2.00 | 101.90 | 124.87 |
| Cuba | Caribbean | male | 0.668 | 2.13 | 1.34 | 130.89 | 143.05 |
| Cyprus | Western Europe | male | 0.841 | 7.52 | 4.04 | 178.18 | 127.24 |
| Czechia | Central Europe | male | 0.557 | 2.43 | 2.54 | 139.14 | 177.77 |
| Democratic Republic of the Congo | Central sub-Saharan Africa | male | 0.568 | 1.70 | 2.53 | 46.95 | 78.87 |
| Denmark | Western Europe | male | 0.89 | 1.67 | 1.22 | 53.33 | 42.57 |
| Djibouti | Eastern Sub-Saharan Africa | male | 0.459 | 1.92 | 8.31 | 50.57 | 222.56 |
| Dominica | Caribbean | male | 0.729 | 4.95 | 8.05 | 157.21 | 329.21 |
| Dominican Republic | Caribbean | male | 0.592 | 0.88 | 4.20 | 31.01 | 157.09 |
| Ecuador | Andean Latin America | male | 0.64 | 1.83 | 5.29 | 68.61 | 182.93 |
| Egypt | North Africa and Middle East | male | 0.658 | 4.23 | 7.01 | 131.91 | 274.62 |
| El Salvador | Central Latin America | male | 0.573 | 0.91 | 5.27 | 44.83 | 239.40 |
| Equatorial Guinea | Central sub-Saharan Africa | male | 0.685 | 1.58 | 14.47 | 46.21 | 393.70 |
| Eritrea | Eastern Sub-Saharan Africa | male | 0.396 | 1.35 | 3.80 | 38.59 | 108.80 |
| Estonia | Eastern Europe | male | 0.835 | 0.28 | 0.19 | 23.43 | 17.46 |
| Ethiopia | Eastern Sub-Saharan Africa | male | 0.343 | 0.85 | 1.71 | 22.92 | 43.40 |
| Micronesia (Federated States of) | Oceania | male | 0.58 | 2.46 | 9.93 | 71.93 | 295.67 |
| Fiji | Oceania | male | 0.664 | 4.99 | 21.52 | 134.09 | 552.45 |
| Finland | Western Europe | male | 0.856 | 0.42 | 0.12 | 27.32 | 14.54 |
| France | Western Europe | male | 0.834 | 1.33 | 0.95 | 40.31 | 34.73 |
| Gabon | Central sub-Saharan Africa | male | 0.656 | 7.11 | 19.29 | 188.00 | 529.17 |
| Georgia | Central Asia | male | 0.702 | 1.20 | 3.12 | 54.14 | 147.59 |
| Germany | Western Europe | male | 0.898 | 2.40 | 1.20 | 94.68 | 73.34 |
| Ghana | Western sub-Saharan Africa | male | 0.557 | 1.71 | 8.12 | 48.36 | 230.09 |
| Greece | Western Europe | male | 0.794 | 1.06 | 0.74 | 56.28 | 68.92 |
| Greenland | High-income North America | male | 0.761 | 0.73 | 0.50 | 23.18 | 24.95 |
| Grenada | Caribbean | male | 0.669 | 5.44 | 11.47 | 166.45 | 392.05 |
| Guam | Oceania | male | 0.813 | 2.07 | 1.55 | 71.50 | 80.27 |
| Guatemala | Central Latin America | male | 0.526 | 0.69 | 5.82 | 34.31 | 217.53 |
| Guinea | Western sub-Saharan Africa | male | 0.325 | 0.64 | 1.62 | 17.82 | 48.93 |
| Guinea-Bissau | Western sub-Saharan Africa | male | 0.355 | 1.37 | 2.66 | 37.94 | 78.08 |
| Guyana | Caribbean | male | 0.618 | 7.40 | 13.15 | 239.68 | 450.72 |
| Haiti | Caribbean | male | 0.432 | 0.87 | 1.42 | 29.04 | 59.37 |
| Honduras | Central Latin America | male | 0.496 | 0.44 | 1.46 | 31.46 | 96.46 |
| Hungary | Central Europe | male | 0.791 | 1.57 | 1.94 | 90.33 | 124.54 |
| Iceland | Western Europe | male | 0.869 | 0.28 | 0.18 | 13.53 | 13.09 |
| India | South Asia | male | 0.566 | 1.56 | 4.80 | 53.42 | 188.36 |
| Indonesia | Southeast Asia | male | 0.66 | 2.05 | 6.05 | 65.05 | 188.29 |
| Iran (Islamic Republic of) | North Africa and Middle East | male | 0.67 | 2.23 | 4.43 | 98.93 | 202.84 |
| Iraq | North Africa and Middle East | male | 0.671 | 8.21 | 10.99 | 258.97 | 377.48 |
| Ireland | Western Europe | male | 0.867 | 1.26 | 0.48 | 34.89 | 27.79 |
| Israel | Western Europe | male | 0.803 | 2.92 | 3.83 | 89.83 | 124.46 |
| Italy | Western Europe | male | 0.801 | 2.99 | 1.87 | 100.61 | 90.85 |
| Jamaica | Caribbean | male | 0.684 | 2.94 | 8.52 | 85.36 | 263.41 |
| Japan | High-income Asia Pacific | male | 0.87 | 0.79 | 0.31 | 46.80 | 48.26 |
| Jordan | North Africa and Middle East | male | 0.731 | 9.36 | 8.22 | 268.73 | 285.71 |
| Kazakhstan | Central Asia | male | 0.723 | 0.61 | 2.16 | 40.73 | 112.82 |
| Kenya | Eastern Sub-Saharan Africa | male | 0.508 | 0.91 | 2.75 | 24.24 | 76.35 |
| Kiribati | Oceania | male | 0.527 | 2.46 | 6.35 | 75.43 | 191.87 |
| Kuwait | North Africa and Middle East | male | 0.851 | 5.80 | 4.69 | 241.81 | 289.98 |
| Kyrgyzstan | Central Asia | male | 0.596 | 0.47 | 0.87 | 26.22 | 58.21 |
| Lao People's Democratic Republic | Southeast Asia | male | 0.49 | 0.76 | 1.69 | 25.20 | 64.72 |
| Latvia | Eastern Europe | male | 0.82 | 0.64 | 0.84 | 46.52 | 55.77 |
| Lebanon | North Africa and Middle East | male | 0.708 | 3.91 | 4.42 | 144.41 | 238.88 |
| Lesotho | Southern sub-Saharan Africa | male | 0.507 | 2.40 | 10.38 | 62.37 | 269.74 |
| Liberia | Western sub-Saharan Africa | male | 0.37 | 0.91 | 1.85 | 27.44 | 63.77 |
| Libya | North Africa and Middle East | male | 0.709 | 2.13 | 3.76 | 109.82 | 248.60 |
| Lithuania | Eastern Europe | male | 0.843 | 0.51 | 0.41 | 40.39 | 36.52 |
| Luxembourg | Western Europe | male | 0.895 | 1.44 | 0.59 | 44.29 | 56.45 |
| North Macedonia | Central Europe | male | 0.744 | 2.54 | 5.78 | 115.19 | 236.37 |
| Madagascar | Eastern Sub-Saharan Africa | male | 0.396 | 0.46 | 0.96 | 12.53 | 28.61 |
| Malawi | Eastern Sub-Saharan Africa | male | 0.384 | 0.67 | 1.59 | 17.84 | 45.76 |
| Malaysia | Southeast Asia | male | 0.737 | 4.02 | 1.83 | 154.53 | 117.69 |
| Maldives | Southeast Asia | male | 0.562 | 1.01 | 1.67 | 31.44 | 67.30 |
| Mali | Western sub-Saharan Africa | male | 0.263 | 0.59 | 1.17 | 16.48 | 36.09 |
| Malta | Western Europe | male | 0.801 | 3.00 | 1.68 | 95.26 | 79.73 |
| Marshall Islands | Oceania | male | 0.544 | 1.72 | 4.12 | 59.64 | 170.46 |
| Mauritania | Western sub-Saharan Africa | male | 0.496 | 2.09 | 3.83 | 52.87 | 99.89 |
| Mauritius | Southeast Asia | male | 0.705 | 4.91 | 16.17 | 176.20 | 488.83 |
| Mexico | Central Latin America | male | 0.649 | 8.06 | 10.19 | 285.74 | 365.71 |
| Republic of Moldova | Eastern Europe | male | 0.696 | 0.67 | 0.59 | 44.94 | 59.12 |
| Mongolia | Central Asia | male | 0.606 | 0.36 | 1.10 | 16.33 | 58.14 |
| Montenegro | Central Europe | male | 0.791 | 1.87 | 2.26 | 103.45 | 142.19 |
| Morocco | North Africa and Middle East | male | 0.548 | 1.09 | 4.09 | 48.73 | 196.00 |
| Mozambique | Eastern Sub-Saharan Africa | male | 0.307 | 0.41 | 1.48 | 10.79 | 41.14 |
| Myanmar | Southeast Asia | male | 0.521 | 1.92 | 5.30 | 55.18 | 158.74 |
| Namibia | Southern sub-Saharan Africa | male | 0.612 | 3.11 | 8.53 | 81.27 | 222.06 |
| Nepal | South Asia | male | 0.422 | 0.42 | 2.21 | 19.92 | 111.52 |
| Netherlands | Western Europe | male | 0.883 | 2.64 | 1.06 | 86.79 | 51.90 |
| New Zealand | Australasia | male | 0.84 | 0.57 | 0.34 | 18.78 | 15.54 |
| Nicaragua | Central Latin America | male | 0.517 | 0.96 | 4.18 | 35.50 | 150.70 |
| Niger | Western sub-Saharan Africa | male | 0.162 | 0.60 | 0.99 | 14.76 | 27.83 |
| Nigeria | Western sub-Saharan Africa | male | 0.515 | 1.73 | 4.94 | 42.02 | 120.43 |
| Democratic People's Republic of Korea | East Asia | male | 0.558 | 0.75 | 1.37 | 30.30 | 69.45 |
| Northern Mariana Islands | Oceania | male | 0.771 | 3.27 | 4.24 | 107.74 | 147.08 |
| Norway | Western Europe | male | 0.913 | 0.77 | 0.28 | 41.66 | 20.27 |
| Oman | North Africa and Middle East | male | 0.783 | 5.54 | 14.78 | 155.47 | 393.45 |
| Pakistan | South Asia | male | 0.449 | 1.16 | 6.24 | 40.50 | 205.41 |
| Palestine | North Africa and Middle East | male | 0.588 | 5.77 | 15.04 | 155.31 | 426.44 |
| Panama | Central Latin America | male | 0.686 | 1.21 | 3.09 | 62.41 | 146.54 |
| Papua New Guinea | Oceania | male | 0.394 | 1.89 | 3.68 | 53.64 | 108.70 |
| Paraguay | Tropical Latin America | male | 0.638 | 0.88 | 3.55 | 30.39 | 114.90 |
| Peru | Andean Latin America | male | 0.648 | 1.39 | 2.51 | 51.25 | 108.43 |
| Philippines | Southeast Asia | male | 0.623 | 3.09 | 3.95 | 92.85 | 128.41 |
| Poland | Central Europe | male | 0.802 | 1.85 | 2.12 | 105.97 | 143.55 |
| Portugal | Western Europe | male | 0.743 | 1.86 | 1.06 | 63.23 | 48.72 |
| Puerto Rico | Caribbean | male | 0.814 | 2.35 | 2.18 | 83.37 | 88.72 |
| Qatar | North Africa and Middle East | male | 0.83 | 27.34 | 26.10 | 616.72 | 672.11 |
| Romania | Central Europe | male | 0.76 | 0.83 | 0.93 | 51.55 | 73.61 |
| Russian Federation | Eastern Europe | male | 0.805 | 0.49 | 0.61 | 35.07 | 37.12 |
| Rwanda | Eastern Sub-Saharan Africa | male | 0.429 | 1.65 | 2.70 | 43.24 | 70.87 |
| Saint Lucia | Caribbean | male | 0.67 | 6.84 | 10.15 | 206.50 | 365.54 |
| Saint Vincent and the Grenadines | Caribbean | male | 0.627 | 5.91 | 12.04 | 181.00 | 407.14 |
| Samoa | Oceania | male | 0.641 | 3.07 | 4.30 | 89.15 | 138.68 |
| Sao Tome and Principe | Central sub-Saharan Africa | male | 0.67 | 0.48 | 1.86 | 17.65 | 75.85 |
| Saudi Arabia | North Africa and Middle East | male | 0.805 | 3.26 | 4.95 | 118.32 | 262.73 |
| Senegal | Western sub-Saharan Africa | male | 0.389 | 1.34 | 2.85 | 41.76 | 95.88 |
| Serbia | Central Europe | male | 0.767 | 2.34 | 3.82 | 107.86 | 187.08 |
| Seychelles | Southeast Asia | male | 0.724 | 1.67 | 3.26 | 83.76 | 192.33 |
| Sierra Leone | Western sub-Saharan Africa | male | 0.347 | 0.76 | 1.43 | 18.73 | 38.98 |
| Singapore | High-income Asia Pacific | male | 0.861 | 2.58 | 0.43 | 129.33 | 90.43 |
| Slovakia | Central Europe | male | 0.812 | 2.49 | 1.45 | 114.26 | 101.18 |
| Slovenia | Central Europe | male | 0.84 | 1.68 | 1.35 | 89.73 | 90.92 |
| Solomon Islands | Oceania | male | 0.407 | 0.72 | 2.99 | 23.78 | 100.86 |
| Somalia | Eastern Sub-Saharan Africa | male | 0.081 | 0.40 | 0.55 | 10.95 | 15.85 |
| South Africa | Southern sub-Saharan Africa | male | 0.678 | 5.56 | 12.12 | 159.87 | 330.24 |
| Republic of Korea | High-income Asia Pacific | male | 0.46 | 3.99 | 3.68 | 143.82 | 143.03 |
| South Sudan | Eastern Sub-Saharan Africa | male | 0.363 | 1.09 | 1.89 | 28.44 | 53.10 |
| Spain | Western Europe | male | 0.767 | 1.75 | 0.74 | 69.86 | 52.71 |
| Sri Lanka | Southeast Asia | male | 0.69 | 1.82 | 7.43 | 61.39 | 242.07 |
| Sudan | North Africa and Middle East | male | 0.515 | 0.36 | 2.30 | 17.28 | 131.87 |
| Suriname | Caribbean | male | 0.636 | 3.89 | 7.35 | 152.89 | 320.77 |
| Eswatini | Southern sub-Saharan Africa | male | 0.78 | 4.21 | 17.80 | 104.18 | 439.49 |
| Sweden | Western Europe | male | 0.872 | 0.80 | 0.29 | 30.61 | 13.98 |
| Switzerland | Western Europe | male | 0.929 | 2.09 | 0.67 | 70.74 | 40.21 |
| Syrian Arab Republic | North Africa and Middle East | male | 0.636 | 2.79 | 2.92 | 124.11 | 174.02 |
| Taiwan (Province of China) | East Asia | male | 0.515 | 3.62 | 5.31 | 119.44 | 190.19 |
| Tajikistan | Central Asia | male | 0.539 | 0.89 | 5.09 | 38.65 | 192.58 |
| United Republic of Tanzania | Eastern Sub-Saharan Africa | male | 0.902 | 0.68 | 1.85 | 16.50 | 49.11 |
| Thailand | Southeast Asia | male | 0.687 | 2.35 | 3.03 | 84.16 | 146.72 |
| Bahamas | Caribbean | male | 0.877 | 5.78 | 5.20 | 195.43 | 216.62 |
| Gambia | Western sub-Saharan Africa | male | 0.902 | 0.87 | 2.68 | 23.91 | 77.49 |
| Timor-Leste | Southeast Asia | male | 0.514 | 0.24 | 0.93 | 8.52 | 40.77 |
| Togo | Western sub-Saharan Africa | male | 0.417 | 1.02 | 2.51 | 26.89 | 69.96 |
| Tonga | Oceania | male | 0.636 | 2.25 | 6.99 | 65.02 | 206.55 |
| Trinidad and Tobago | Caribbean | male | 0.757 | 19.09 | 18.47 | 571.77 | 580.40 |
| Tunisia | North Africa and Middle East | male | 0.672 | 1.74 | 3.29 | 100.03 | 218.50 |
| Turkey | North Africa and Middle East | male | 0.748 | 5.32 | 4.13 | 162.50 | 170.30 |
| Turkmenistan | Central Asia | male | 0.67 | 1.78 | 3.17 | 82.24 | 155.84 |
| Uganda | Eastern Sub-Saharan Africa | male | 0.404 | 0.86 | 2.89 | 22.71 | 80.75 |
| Ukraine | Eastern Europe | male | 0.736 | 0.61 | 0.48 | 46.97 | 48.02 |
| United Arab Emirates | North Africa and Middle East | male | 0.88 | 15.45 | 13.21 | 406.86 | 441.28 |
| United Kingdom | Western Europe | male | 0.902 | 1.39 | 0.42 | 62.41 | 59.77 |
| United States of America | High-income North America | male | 0.618 | 1.65 | 0.89 | 79.22 | 53.79 |
| Uruguay | Southern Latin America | male | 0.697 | 1.34 | 1.41 | 32.27 | 51.38 |
| Uzbekistan | Central Asia | male | 0.631 | 1.21 | 7.21 | 56.52 | 265.90 |
| Vanuatu | Oceania | male | 0.485 | 0.80 | 2.41 | 28.02 | 87.04 |
| Venezuela (Bolivarian Republic of) | Central Latin America | male | 0.412 | 4.74 | 7.38 | 180.30 | 278.39 |
| Viet nam | Southeast Asia | male | 0.902 | 0.94 | 3.82 | 28.78 | 124.54 |
| United States Virgin Islands | Caribbean | male | 0.877 | 2.46 | 3.70 | 87.20 | 141.86 |
| Yemen | North Africa and Middle East | male | 0.412 | 0.35 | 1.63 | 15.08 | 85.11 |
| Zambia | Eastern Sub-Saharan Africa | male | 0.505 | 1.25 | 3.94 | 32.68 | 107.87 |
| Zimbabwe | Southern sub-Saharan Africa | male | 0.476 | 1.18 | 2.65 | 39.10 | 87.87 |
| Monaco | Western Europe | male | 0.902 | 0.34 | 0.47 | 20.39 | 42.92 |
| San Marino | Western Europe | male | 0.884 | 0.85 | 0.61 | 35.09 | 40.13 |
| Saint Kitts and Nevis | Caribbean | male | 0.746 | 3.53 | 4.31 | 108.95 | 152.13 |
| Cook Islands | Oceania | male | 0.764 | 4.37 | 4.88 | 115.55 | 150.76 |
| Nauru | Oceania | male | 0.618 | 3.10 | 5.40 | 89.86 | 161.46 |
| Niue | Oceania | male | 0.711 | 2.87 | 6.10 | 90.09 | 198.70 |
| Palau | Oceania | male | 0.738 | 3.45 | 5.52 | 107.01 | 179.39 |
| Tokelau | Oceania | male | 0.626 | 2.29 | 2.87 | 72.65 | 98.95 |
| Tuvalu | Oceania | male | 0.589 | 1.16 | 3.45 | 34.79 | 111.08 |
| Afghanistan | North Africa and Middle East | female | 0.343 | 0.72 | 3.06 | 23.92 | 108.40 |
| Albania | Central Europe | female | 0.681 | 0.25 | 0.38 | 15.79 | 38.13 |
| Algeria | North Africa and Middle East | female | 0.652 | 3.17 | 4.88 | 109.21 | 221.81 |
| American Samoa | Oceania | female | 0.712 | 2.24 | 3.88 | 74.47 | 146.12 |
| Andorra | Western Europe | female | 0.894 | 0.63 | 0.30 | 27.69 | 25.15 |
| Angola | Central sub-Saharan Africa | female | 0.47 | 0.41 | 2.30 | 13.01 | 74.43 |
| Antigua and Barbuda | Caribbean | female | 0.743 | 7.11 | 9.11 | 215.55 | 285.73 |
| Argentina | Southern Latin America | female | 0.708 | 1.90 | 1.76 | 59.03 | 74.90 |
| Armenia | Central Asia | female | 0.689 | 2.62 | 5.94 | 97.71 | 217.70 |
| Australia | Australasia | female | 0.839 | 0.53 | 0.32 | 17.67 | 16.08 |
| Austria | Western Europe | female | 0.849 | 1.98 | 0.99 | 59.69 | 46.43 |
| Azerbaijan | Central Asia | female | 0.683 | 0.93 | 3.97 | 44.45 | 169.21 |
| Bahrain | North Africa and Middle East | female | 0.751 | 15.88 | 30.89 | 420.94 | 761.95 |
| Bangladesh | South Asia | female | 0.483 | 0.65 | 3.12 | 18.81 | 85.36 |
| Barbados | Caribbean | female | 0.742 | 12.56 | 10.96 | 338.01 | 325.20 |
| Belarus | Eastern Europe | female | 0.745 | 0.73 | 0.29 | 51.28 | 38.92 |
| Belgium | Western Europe | female | 0.851 | 1.98 | 0.63 | 66.54 | 47.86 |
| Belize | Caribbean | female | 0.603 | 3.20 | 7.81 | 90.33 | 252.34 |
| Benin | Western sub-Saharan Africa | female | 0.352 | 0.45 | 1.50 | 13.21 | 45.74 |
| Bermuda | Caribbean | female | 0.813 | 2.06 | 0.67 | 59.43 | 33.60 |
| Bhutan | South Asia | female | 0.455 | 0.34 | 2.84 | 11.41 | 88.32 |
| Bolivia (Plurinational State of) | Andean Latin America | female | 0.566 | 3.85 | 6.39 | 107.19 | 175.85 |
| Bosnia and Herzegovina | Central Europe | female | 0.718 | 1.33 | 5.80 | 50.70 | 189.52 |
| Botswana | Southern sub-Saharan Africa | female | 0.634 | 2.20 | 10.27 | 56.40 | 268.42 |
| Brazil | Tropical Latin America | female | 0.64 | 2.25 | 2.29 | 73.64 | 82.68 |
| Brunei Darussalam | High-income Asia Pacific | female | 0.823 | 5.26 | 3.54 | 143.65 | 113.84 |
| Bulgaria | Central Europe | female | 0.764 | 2.18 | 1.84 | 88.31 | 96.42 |
| Burkina Faso | Western sub-Saharan Africa | female | 0.257 | 0.40 | 0.81 | 11.60 | 26.48 |
| Burundi | Eastern Sub-Saharan Africa | female | 0.284 | 0.54 | 0.63 | 14.90 | 18.21 |
| Cambodia | Southeast Asia | female | 0.469 | 0.51 | 1.27 | 14.76 | 44.67 |
| Cameroon | Western sub-Saharan Africa | female | 0.49 | 1.55 | 4.97 | 39.99 | 132.45 |
| Canada | High-income North America | female | 0.873 | 0.93 | 0.37 | 24.46 | 16.75 |
| Cabo Verde | Western sub-Saharan Africa | female | 0.525 | 0.33 | 5.49 | 15.94 | 177.44 |
| Central African Republic | Central sub-Saharan Africa | female | 0.274 | 0.55 | 1.01 | 17.40 | 33.60 |
| Chad | Western sub-Saharan Africa | female | 0.238 | 0.36 | 1.10 | 10.54 | 32.93 |
| Chile | Southern Latin America | female | 0.759 | 1.63 | 2.45 | 61.43 | 115.50 |
| China | East Asia | female | 0.686 | 0.63 | 1.55 | 29.61 | 79.05 |
| Colombia | Central Latin America | female | 0.633 | 2.07 | 1.97 | 85.49 | 122.73 |
| Comoros | Eastern Sub-Saharan Africa | female | 0.455 | 0.37 | 0.96 | 9.64 | 25.79 |
| Congo | Central sub-Saharan Africa | female | 0.568 | 1.45 | 5.17 | 41.96 | 155.64 |
| Costa Rica | Central Latin America | female | 0.68 | 1.25 | 1.51 | 55.86 | 109.96 |
| Côte d'Ivoire | Western sub-Saharan Africa | female | 0.408 | 0.83 | 2.54 | 22.93 | 74.74 |
| Croatia | Central Europe | female | 0.794 | 1.84 | 1.54 | 82.88 | 93.36 |
| Cuba | Caribbean | female | 0.668 | 3.62 | 1.56 | 138.19 | 113.70 |
| Cyprus | Western Europe | female | 0.841 | 11.33 | 3.59 | 199.88 | 100.75 |
| Czechia | Central Europe | female | 0.557 | 2.06 | 1.83 | 109.46 | 127.82 |
| Democratic Republic of the Congo | Central sub-Saharan Africa | female | 0.568 | 0.56 | 0.87 | 16.99 | 31.05 |
| Denmark | Western Europe | female | 0.89 | 1.14 | 0.71 | 37.41 | 29.82 |
| Djibouti | Eastern Sub-Saharan Africa | female | 0.459 | 1.08 | 5.21 | 29.37 | 143.38 |
| Dominica | Caribbean | female | 0.729 | 5.17 | 9.30 | 142.03 | 292.08 |
| Dominican Republic | Caribbean | female | 0.592 | 0.81 | 3.46 | 24.83 | 122.65 |
| Ecuador | Andean Latin America | female | 0.64 | 2.20 | 5.43 | 69.09 | 178.10 |
| Egypt | North Africa and Middle East | female | 0.658 | 5.43 | 10.10 | 164.71 | 322.28 |
| El Salvador | Central Latin America | female | 0.573 | 0.81 | 5.13 | 32.11 | 195.20 |
| Equatorial Guinea | Central sub-Saharan Africa | female | 0.685 | 0.47 | 7.80 | 15.02 | 225.03 |
| Eritrea | Eastern Sub-Saharan Africa | female | 0.396 | 0.65 | 2.36 | 19.06 | 65.12 |
| Estonia | Eastern Europe | female | 0.835 | 0.21 | 0.15 | 18.48 | 14.85 |
| Ethiopia | Eastern Sub-Saharan Africa | female | 0.343 | 0.40 | 0.87 | 11.41 | 24.12 |
| Micronesia (Federated States of) | Oceania | female | 0.58 | 1.85 | 8.21 | 51.93 | 230.54 |
| Fiji | Oceania | female | 0.664 | 3.23 | 16.08 | 89.62 | 434.59 |
| Finland | Western Europe | female | 0.856 | 0.45 | 0.07 | 24.98 | 12.70 |
| France | Western Europe | female | 0.834 | 1.10 | 0.59 | 30.08 | 24.09 |
| Gabon | Central sub-Saharan Africa | female | 0.656 | 1.98 | 8.68 | 55.81 | 251.14 |
| Georgia | Central Asia | female | 0.702 | 0.75 | 2.00 | 38.55 | 100.86 |
| Germany | Western Europe | female | 0.898 | 2.60 | 0.91 | 89.28 | 57.42 |
| Ghana | Western sub-Saharan Africa | female | 0.557 | 1.42 | 3.89 | 41.17 | 122.66 |
| Greece | Western Europe | female | 0.794 | 1.30 | 0.58 | 52.56 | 50.85 |
| Greenland | High-income North America | female | 0.761 | 0.65 | 0.25 | 15.84 | 13.03 |
| Grenada | Caribbean | female | 0.669 | 5.06 | 11.67 | 150.74 | 364.19 |
| Guam | Oceania | female | 0.813 | 3.34 | 1.61 | 95.33 | 77.28 |
| Guatemala | Central Latin America | female | 0.526 | 0.65 | 5.42 | 27.01 | 188.60 |
| Guinea | Western sub-Saharan Africa | female | 0.325 | 0.58 | 1.52 | 15.39 | 42.43 |
| Guinea-Bissau | Western sub-Saharan Africa | female | 0.355 | 0.69 | 2.05 | 19.87 | 58.21 |
| Guyana | Caribbean | female | 0.618 | 7.85 | 13.78 | 242.35 | 447.41 |
| Haiti | Caribbean | female | 0.432 | 1.38 | 2.34 | 39.94 | 76.00 |
| Honduras | Central Latin America | female | 0.496 | 0.31 | 1.13 | 19.04 | 66.38 |
| Hungary | Central Europe | female | 0.791 | 1.66 | 1.35 | 76.27 | 84.20 |
| Iceland | Western Europe | female | 0.869 | 0.21 | 0.06 | 10.70 | 9.14 |
| India | South Asia | female | 0.566 | 1.12 | 3.77 | 37.04 | 144.25 |
| Indonesia | Southeast Asia | female | 0.66 | 1.63 | 4.90 | 52.29 | 151.13 |
| Iran (Islamic Republic of) | North Africa and Middle East | female | 0.67 | 2.78 | 5.39 | 102.68 | 221.95 |
| Iraq | North Africa and Middle East | female | 0.671 | 8.28 | 9.95 | 255.74 | 362.54 |
| Ireland | Western Europe | female | 0.867 | 0.99 | 0.30 | 27.08 | 20.11 |
| Israel | Western Europe | female | 0.803 | 3.05 | 3.13 | 89.52 | 102.27 |
| Italy | Western Europe | female | 0.801 | 3.38 | 1.39 | 97.70 | 68.58 |
| Jamaica | Caribbean | female | 0.684 | 3.28 | 9.83 | 91.28 | 286.81 |
| Japan | High-income Asia Pacific | female | 0.87 | 0.62 | 0.17 | 29.41 | 29.60 |
| Jordan | North Africa and Middle East | female | 0.731 | 16.22 | 8.99 | 380.52 | 259.96 |
| Kazakhstan | Central Asia | female | 0.723 | 0.67 | 2.30 | 47.59 | 133.71 |
| Kenya | Eastern Sub-Saharan Africa | female | 0.508 | 0.44 | 1.11 | 11.89 | 32.29 |
| Kiribati | Oceania | female | 0.527 | 1.77 | 3.36 | 52.66 | 98.73 |
| Kuwait | North Africa and Middle East | female | 0.851 | 7.91 | 4.00 | 265.58 | 243.16 |
| Kyrgyzstan | Central Asia | female | 0.596 | 0.36 | 0.76 | 20.23 | 52.08 |
| Lao People's Democratic Republic | Southeast Asia | female | 0.49 | 0.64 | 1.52 | 20.24 | 52.69 |
| Latvia | Eastern Europe | female | 0.82 | 0.67 | 0.77 | 43.78 | 48.72 |
| Lebanon | North Africa and Middle East | female | 0.708 | 2.72 | 2.41 | 113.33 | 178.42 |
| Lesotho | Southern sub-Saharan Africa | female | 0.507 | 1.42 | 7.41 | 36.89 | 184.09 |
| Liberia | Western sub-Saharan Africa | female | 0.37 | 0.67 | 1.75 | 19.65 | 53.86 |
| Libya | North Africa and Middle East | female | 0.709 | 2.27 | 4.37 | 101.84 | 254.88 |
| Lithuania | Eastern Europe | female | 0.843 | 0.44 | 0.30 | 33.29 | 27.42 |
| Luxembourg | Western Europe | female | 0.895 | 1.41 | 0.46 | 39.83 | 43.82 |
| North Macedonia | Central Europe | female | 0.744 | 3.16 | 6.25 | 112.11 | 217.89 |
| Madagascar | Eastern Sub-Saharan Africa | female | 0.396 | 0.26 | 0.58 | 7.53 | 17.48 |
| Malawi | Eastern Sub-Saharan Africa | female | 0.384 | 0.35 | 0.58 | 9.76 | 17.78 |
| Malaysia | Southeast Asia | female | 0.737 | 5.03 | 2.06 | 170.66 | 120.53 |
| Maldives | Southeast Asia | female | 0.562 | 1.03 | 1.23 | 30.03 | 51.94 |
| Mali | Western sub-Saharan Africa | female | 0.263 | 0.53 | 1.37 | 14.76 | 38.90 |
| Malta | Western Europe | female | 0.801 | 3.45 | 1.24 | 98.93 | 72.52 |
| Marshall Islands | Oceania | female | 0.544 | 1.38 | 5.25 | 45.38 | 177.22 |
| Mauritania | Western sub-Saharan Africa | female | 0.496 | 1.70 | 5.03 | 43.58 | 129.08 |
| Mauritius | Southeast Asia | female | 0.705 | 4.01 | 12.60 | 136.31 | 375.36 |
| Mexico | Central Latin America | female | 0.649 | 9.16 | 8.28 | 287.64 | 285.12 |
| Republic of Moldova | Eastern Europe | female | 0.696 | 0.57 | 0.50 | 38.06 | 53.18 |
| Mongolia | Central Asia | female | 0.606 | 0.18 | 0.51 | 10.15 | 37.51 |
| Montenegro | Central Europe | female | 0.791 | 1.71 | 1.90 | 83.72 | 115.83 |
| Morocco | North Africa and Middle East | female | 0.548 | 1.13 | 5.17 | 44.44 | 220.41 |
| Mozambique | Eastern Sub-Saharan Africa | female | 0.307 | 0.20 | 0.49 | 5.33 | 14.27 |
| Myanmar | Southeast Asia | female | 0.521 | 1.32 | 2.98 | 40.74 | 97.79 |
| Namibia | Southern sub-Saharan Africa | female | 0.612 | 2.24 | 5.55 | 59.14 | 147.58 |
| Nepal | South Asia | female | 0.422 | 0.30 | 2.01 | 11.08 | 69.34 |
| Netherlands | Western Europe | female | 0.883 | 2.73 | 0.85 | 75.44 | 37.94 |
| New Zealand | Australasia | female | 0.84 | 0.43 | 0.22 | 15.85 | 11.93 |
| Nicaragua | Central Latin America | female | 0.517 | 0.55 | 3.03 | 22.06 | 105.05 |
| Niger | Western sub-Saharan Africa | female | 0.162 | 0.41 | 0.94 | 10.23 | 25.03 |
| Nigeria | Western sub-Saharan Africa | female | 0.515 | 1.01 | 3.48 | 25.66 | 90.03 |
| Democratic People's Republic of Korea | East Asia | female | 0.558 | 0.60 | 1.12 | 22.38 | 52.87 |
| Northern Mariana Islands | Oceania | female | 0.771 | 3.77 | 3.27 | 112.79 | 116.62 |
| Norway | Western Europe | female | 0.913 | 0.57 | 0.19 | 31.18 | 16.03 |
| Oman | North Africa and Middle East | female | 0.783 | 5.26 | 12.43 | 146.21 | 353.40 |
| Pakistan | South Asia | female | 0.449 | 0.77 | 6.47 | 26.06 | 191.46 |
| Palestine | North Africa and Middle East | female | 0.588 | 5.18 | 13.99 | 129.68 | 372.43 |
| Panama | Central Latin America | female | 0.686 | 1.41 | 3.01 | 53.67 | 125.66 |
| Papua New Guinea | Oceania | female | 0.394 | 0.57 | 1.38 | 18.51 | 46.34 |
| Paraguay | Tropical Latin America | female | 0.638 | 0.88 | 3.10 | 27.74 | 90.05 |
| Peru | Andean Latin America | female | 0.648 | 1.12 | 2.27 | 39.44 | 94.74 |
| Philippines | Southeast Asia | female | 0.623 | 2.49 | 3.09 | 61.97 | 98.38 |
| Poland | Central Europe | female | 0.802 | 1.90 | 1.50 | 94.58 | 101.85 |
| Portugal | Western Europe | female | 0.743 | 1.80 | 0.88 | 57.23 | 41.77 |
| Puerto Rico | Caribbean | female | 0.814 | 2.47 | 1.66 | 78.06 | 70.66 |
| Qatar | North Africa and Middle East | female | 0.83 | 27.12 | 42.14 | 622.68 | 880.83 |
| Romania | Central Europe | female | 0.76 | 0.68 | 0.67 | 38.71 | 54.91 |
| Russian Federation | Eastern Europe | female | 0.805 | 0.62 | 0.80 | 37.76 | 39.48 |
| Rwanda | Eastern Sub-Saharan Africa | female | 0.429 | 0.81 | 1.31 | 21.94 | 35.54 |
| Saint Lucia | Caribbean | female | 0.67 | 6.98 | 9.69 | 212.09 | 371.41 |
| Saint Vincent and the Grenadines | Caribbean | female | 0.627 | 7.75 | 13.54 | 209.62 | 419.45 |
| Samoa | Oceania | female | 0.641 | 1.70 | 3.42 | 51.29 | 110.03 |
| Sao Tome and Principe | Central sub-Saharan Africa | female | 0.67 | 0.21 | 0.86 | 10.02 | 46.24 |
| Saudi Arabia | North Africa and Middle East | female | 0.805 | 2.83 | 4.18 | 98.06 | 234.52 |
| Senegal | Western sub-Saharan Africa | female | 0.389 | 0.83 | 2.54 | 26.48 | 79.07 |
| Serbia | Central Europe | female | 0.767 | 2.51 | 3.51 | 92.14 | 146.19 |
| Seychelles | Southeast Asia | female | 0.724 | 1.58 | 3.46 | 69.68 | 182.67 |
| Sierra Leone | Western sub-Saharan Africa | female | 0.347 | 0.46 | 1.48 | 11.21 | 38.79 |
| Singapore | High-income Asia Pacific | female | 0.861 | 3.22 | 0.35 | 127.22 | 62.06 |
| Slovakia | Central Europe | female | 0.812 | 2.30 | 1.21 | 95.65 | 81.88 |
| Slovenia | Central Europe | female | 0.84 | 1.73 | 0.83 | 77.39 | 65.86 |
| Solomon Islands | Oceania | female | 0.407 | 0.54 | 2.24 | 17.29 | 70.50 |
| Somalia | Eastern Sub-Saharan Africa | female | 0.081 | 0.20 | 0.28 | 5.53 | 8.08 |
| South Africa | Southern sub-Saharan Africa | female | 0.678 | 4.93 | 10.88 | 139.94 | 301.19 |
| Republic of Korea | High-income Asia Pacific | female | 0.46 | 2.54 | 2.48 | 96.95 | 101.85 |
| South Sudan | Eastern Sub-Saharan Africa | female | 0.363 | 0.60 | 1.00 | 16.00 | 28.88 |
| Spain | Western Europe | female | 0.767 | 2.05 | 0.62 | 67.36 | 41.26 |
| Sri Lanka | Southeast Asia | female | 0.69 | 0.99 | 5.41 | 36.32 | 178.23 |
| Sudan | North Africa and Middle East | female | 0.515 | 0.33 | 2.17 | 13.37 | 111.12 |
| Suriname | Caribbean | female | 0.636 | 3.49 | 5.92 | 127.25 | 263.94 |
| Eswatini | Southern sub-Saharan Africa | female | 0.78 | 2.48 | 9.21 | 61.80 | 223.43 |
| Sweden | Western Europe | female | 0.872 | 0.60 | 0.19 | 23.02 | 10.00 |
| Switzerland | Western Europe | female | 0.929 | 1.96 | 0.47 | 60.21 | 30.90 |
| Syrian Arab Republic | North Africa and Middle East | female | 0.636 | 4.07 | 4.19 | 144.24 | 190.06 |
| Taiwan (Province of China) | East Asia | female | 0.515 | 4.47 | 4.39 | 130.35 | 147.39 |
| Tajikistan | Central Asia | female | 0.539 | 0.57 | 3.98 | 26.56 | 158.73 |
| United Republic of Tanzania | Eastern Sub-Saharan Africa | female | 0.902 | 0.38 | 0.96 | 9.23 | 26.52 |
| Thailand | Southeast Asia | female | 0.687 | 2.34 | 3.06 | 73.22 | 128.50 |
| Bahamas | Caribbean | female | 0.877 | 6.56 | 4.99 | 202.98 | 196.74 |
| Gambia | Western sub-Saharan Africa | female | 0.902 | 0.52 | 2.12 | 14.48 | 59.71 |
| Timor-Leste | Southeast Asia | female | 0.514 | 0.29 | 0.95 | 8.35 | 32.88 |
| Togo | Western sub-Saharan Africa | female | 0.417 | 0.68 | 1.72 | 17.58 | 46.15 |
| Tonga | Oceania | female | 0.636 | 2.67 | 5.60 | 74.30 | 165.49 |
| Trinidad and Tobago | Caribbean | female | 0.757 | 19.47 | 15.23 | 575.14 | 481.42 |
| Tunisia | North Africa and Middle East | female | 0.672 | 1.78 | 2.97 | 81.29 | 178.46 |
| Turkey | North Africa and Middle East | female | 0.748 | 6.71 | 4.77 | 181.46 | 172.42 |
| Turkmenistan | Central Asia | female | 0.67 | 1.56 | 2.94 | 77.90 | 154.21 |
| Uganda | Eastern Sub-Saharan Africa | female | 0.404 | 0.35 | 1.13 | 9.90 | 34.38 |
| Ukraine | Eastern Europe | female | 0.736 | 0.55 | 0.35 | 43.88 | 40.16 |
| United Arab Emirates | North Africa and Middle East | female | 0.88 | 19.71 | 11.86 | 466.47 | 401.82 |
| United Kingdom | Western Europe | female | 0.902 | 1.05 | 0.32 | 47.30 | 43.23 |
| United States of America | High-income North America | female | 0.618 | 1.52 | 0.61 | 67.17 | 38.75 |
| Uruguay | Southern Latin America | female | 0.697 | 1.10 | 0.99 | 26.23 | 34.70 |
| Uzbekistan | Central Asia | female | 0.631 | 0.82 | 6.23 | 38.40 | 227.77 |
| Vanuatu | Oceania | female | 0.485 | 0.65 | 2.18 | 20.42 | 69.48 |
| Venezuela (Bolivarian Republic of) | Central Latin America | female | 0.412 | 5.06 | 5.97 | 173.63 | 231.14 |
| Viet nam | Southeast Asia | female | 0.902 | 1.03 | 3.29 | 27.18 | 97.19 |
| United States Virgin Islands | Caribbean | female | 0.877 | 3.37 | 2.78 | 99.37 | 106.03 |
| Yemen | North Africa and Middle East | female | 0.412 | 0.32 | 1.92 | 12.10 | 84.76 |
| Zambia | Eastern Sub-Saharan Africa | female | 0.505 | 0.70 | 1.68 | 19.16 | 48.93 |
| Zimbabwe | Southern sub-Saharan Africa | female | 0.476 | 1.23 | 2.55 | 34.61 | 75.90 |
| Monaco | Western Europe | female | 0.902 | 0.21 | 0.23 | 15.47 | 32.74 |
| San Marino | Western Europe | female | 0.884 | 0.71 | 0.43 | 28.31 | 32.54 |
| Saint Kitts and Nevis | Caribbean | female | 0.746 | 3.78 | 3.52 | 109.49 | 121.16 |
| Cook Islands | Oceania | female | 0.764 | 4.00 | 4.54 | 106.37 | 133.58 |
| Nauru | Oceania | female | 0.618 | 2.86 | 5.39 | 75.48 | 146.61 |
| Niue | Oceania | female | 0.711 | 2.31 | 5.45 | 67.32 | 173.47 |
| Palau | Oceania | female | 0.738 | 3.85 | 5.43 | 110.77 | 163.72 |
| Tokelau | Oceania | female | 0.626 | 4.84 | 5.54 | 138.86 | 163.67 |
| Tuvalu | Oceania | female | 0.589 | 1.06 | 3.94 | 30.69 | 116.08 |
